# Supplementary material for: Quantum theory of the nonlinear Hall effect
Source: Nat Commun. 2021 Aug 19;12:5038. doi: 10.1038/s41467-021-25273-4 (PMC8377135; doi:10.1038/s41467-021-25273-4)
Supplement: Supplementary file 1 — Supplementary Information [file 41467_2021_25273_MOESM1_ESM.pdf]

Supplementary Information
for
"Quantum theory of the nonlinear Hall effect"

Z. Z. Du,[1,2] C. M. Wang,[3,1,2] Hai-Peng Sun,[1,2] Hai-Zhou Lu,[1,2,*] and X. C. Xie[4,5,6]

[1] *Shenzhen Institute for Quantum Science and Engineering and Department of Physics, Southern University of Science and Technology (SUSTech), Shenzhen 518055, China*

[2] *Shenzhen Key Laboratory of Quantum Science and Engineering, Shenzhen 518055, China*

[3] *Department of Physics, Shanghai Normal University, Shanghai 200234, China*

[4] *International Center for Quantum Materials, School of Physics, Peking University, Beijing 100871, China*

[5] *CAS Center for Excellence in Topological Quantum Computation, University of Chinese Academy of Sciences, Beijing 100190, China*

[6] *Beijing Academy of Quantum Information Sciences, West Building 3, No.10, Xibeiwang East Road, Haidian District, Beijing 100193, China*

(Dated: July 27, 2021)

CONTENTS

Supplementary Note 1. The difference between the nonlinear Hall effect and nonlinear optics 3

Supplementary Note 2. Diagrammatics: I. General formalism 3
- A. Feynman rules 4
- B. Generic formula of the quadratic conductivity 5
- C. Diagrammatic correspondence of the generic formula 9
- D. Generic two-band model with disorder 10

Supplementary Note 3. Diagrammatics: II. Triangular diagrams 12
- A. Intrinsic contribution 13
- B. Side-jump contribution 14
  - 1. Anomalous velocity induced side-jump contribution 14
  - 2. Anomalous distribution induced side-jump contribution 16
- C. Skew-scattering contribution 19
  - 1. Intrinsic skew-scattering contribution 19
  - 2. Extrinsic skew-scattering contribution 26

Supplementary Note 4. Diagrammatics: III. Two-photon diagrams 28
- A. Intrinsic contribution 29
- B. Side-jump contribution 29
  - 1. Anomalous velocity induced side-jump contribution 29
  - 2. Anomalous distribution induced side-jump contribution 31
- C. Skew-scattering contribution 32
  - 1. Intrinsic skew-scattering contribution 32
  - 2. Extrinsic skew-scattering contribution 34

Supplementary Note 5. Diagrammatics: IV. Remarks on the methodology 35
- A. Role of the multi-photon processes 35
- B. Results of the original intrinsic triangular diagrams 35

Supplementary Note 6. Diagrammatics: V. General aspects of the diagrammatic theory 40
- A. Intrinsic triangular diagrams 40
- B. Side-jump triangular diagrams 40
- C. Skew-scattering triangular diagrams 42

* Corresponding author: luhz@sustech.edu.cn

D. Two-photon diagrams 43

Supplementary Note 7. Physical quantities and useful identities 44
A. Intrinsic related quantities 44
B. Side-jump related quantities 44
C. Skew-scattering related quantities 45
D. Identities about the Green's functions 47

Supplementary Note 8. 2D tilted Dirac model 48
A. Born approximation 48
1. Self-energy 48
2. Vertex correction 49
3. Edge corrections 51
B. Nonlinear Hall conductivity 54
1. Nonlinear Hall conductivity of the 2D tilted Dirac model 54

Supplementary Note 9. Symmetry aspects of the nonlinear response 54
A. $\mathcal{I}$-symmetry 55
B. $\mathcal{M}$-symmetry 56
C. $\mathcal{C}_n$-symmetry 56
1. $\mathcal{C}_{2n}$-symmetry 57
2. $\mathcal{C}_{2n+1}$-symmetry 58
D. 32 Point groups 58

References 59

Supplementary Note 1. THE DIFFERENCE BETWEEN THE NONLINEAR HALL EFFECT AND NONLINEAR OPTICS

The nonlinear Hall effect describes the transverse nonlinear current response to a longitudinal driving electric field, which has been theoretically predicted on the basis of the Boltzmann transport theory that includes the Berry curvature in momentum space [1]. The nonlinear means that the I-V relation is quadratic. That is, the response current is proportional to E^2 instead of E. The nonlinear Hall effect can exist in both *dc* and *ac* measurements. For a *dc* driving field, the response current is also a direct current, which shows no difference from the linear Hall effect other than the quadratic I-V relation. In the *ac* measurement, the response current has both zero- and double-frequency components because of its E^2 dependence. In order to remove the background signal, the double-frequency component of the Hall conductance is usually measured in experiments [2, 3].

Due to the nonlinear property, the quantum theory of the nonlinear Hall effect must be a nonlinear response theory. As the nonlinear response of the nonlinear optics has been well established for decades, a natural question is why we need to build up a new one for the nonlinear Hall effect. The answer to this question has two main aspects:

1. The scales and physics. For nonlinear optical effects, such as the photovoltaic effect and second-harmonic generation, the frequency of the input light is about 10^{14} Hz. This frequency is chosen such that $\hbar\omega$ is about the same magnitude with the material's band gaps Δ_i, so as to trigger the resonance hopping of the electron from one band to the other. For the nonlinear Hall effect, however, the frequency of the driving *ac* electric field is about $10-1000$ Hz, which is too low to induce any resonance hopping between bands. In theories of optics, the high-frequency approximation and inter-band transition are applied thus the results cannot be applied directly to the nonlinear Hall effect, especially when the frequency approaches zero. A proper justification of the theoretical approaches and approximations should begin with an estimation of scales and magnitudes. The nonlinear Hall effect and the photovoltaic effect have totally different scales, magnitudes, and measurements, as shown below.

TABLE 1. Comparison between the nonlinear Hall effect and the photovoltaic effect.

	Frequency (Hz)	Strength of electric field (V/m)	Input	Output
Nonlinear Hall effect	10-1000	1000	Electric current	Transverse voltage
Photovoltaic effect	10^{14}	10^9	Laser	Longitudinal current

2. The disorder effects. Because of the low frequency driving *ac* electric field, the nonlinear Hall effect is a transport effect, which is only determined by the properties of the electrons near the Fermi surface. Consequently, disorder effects are very important in the nonlinear Hall responses. This is very different from nonlinear optical effects, in which disorder effects are only important when the band broadening or resonance hopping between the impurity bands are involved. The importance of disorder effects, including the side-jump and skew-scattering contributions, has been stressed in previous investigations about nonlinear Hall effect within semiclassical formalism [4–6], and new side-jump contribution without semiclassical correspondence has also been discovered [5]. Thus, for a systematic investigation of the nonlinear Hall effect, a full quantum theory that includes all of the disorder-induced contributions is required.

In the following sections, we establish a quantum theory of the nonlinear Hall effect that includes all of the known (intrinsic, side-jump and skew-scattering) contributions.

Supplementary Note 2. DIAGRAMMATICS: I. GENERAL FORMALISM

In general, the response current of a system in the presence of driving electric filed can be written as

$$\begin{aligned} J_a(t) &= \int_{-\infty}^{t} dt'\Pi_{ab}(t-t')E_b(t') + \int_{-\infty}^{t} dt' \int_{-\infty}^{t'} dt''\Xi_{abc}(t'-t'')E_b(t')E_c(t'') + \cdots \\ &= \int_{-\infty}^{t} dt'\Pi_{ab}(t-t')E_b(t') + \frac{1}{2}\int_{-\infty}^{t} dt' \int_{-\infty}^{t} dt''\Xi_{abc}(t-t',t-t'')E_b(t')E_c(t'') + \cdots , \end{aligned} \quad (1)$$

where Π_{ab} and Ξ_{abc} refer to the linear and quadratic response tensors with $\{a,b,c\} \in \{x,y,z\}$, respectively. For a driving electric field $E_a(t) = \mathcal{E}_a e^{-i\omega_a t}$ with the amplitude vector $\mathcal{E}_a$ and frequency ω_a, the above expression can be rewritten after the Fourier transformation as

$$J_a(t) = \Pi_{ab}(\omega_b)\mathcal{E}_b e^{-i\omega_b t} + \frac{1}{2}\Xi_{abc}(\omega_b,\omega_c)\mathcal{E}_b\mathcal{E}_c e^{-i(\omega_b+\omega_c)t}, \quad (2)$$

where

$$\Pi_{ab}(\omega_b) = \int_0^\infty dt \Pi_{ab}(t) e^{i\omega_b t}, \qquad \Xi_{abc}(\omega_b, \omega_c) = \int_0^\infty dt \int_0^\infty dt' \Xi_{abc}(t, t') e^{i\omega_b t} e^{i\omega_c t}. \tag{3}$$

In the nonlinear Hall measurements, the time dependence of the electric field is $E_a(t) = \mathrm{Re}\{\mathcal{E}_a e^{-i\omega_a t}\} = \mathcal{E}_a \cos(\omega_a t)$. Thus, the response current becomes

$$\begin{aligned} J_a(t) = &\frac{1}{2}\mathrm{Re}\Big[\Pi_{ab}(\omega_b)\mathcal{E}_b e^{-i\omega_b t} + \Pi_{ab}(-\omega_b)\mathcal{E}_b e^{i\omega_b t} + \frac{1}{4}\Xi_{abc}(\omega_b, -\omega_c)\mathcal{E}_b\mathcal{E}_c e^{-i(\omega_b - \omega_c)t} \\ &+ \frac{1}{4}\Xi_{abc}(-\omega_b, \omega_c)\mathcal{E}_b\mathcal{E}_c e^{i(\omega_b - \omega_c)t} + \frac{1}{4}\Xi_{abc}(\omega_b, \omega_c)\mathcal{E}_b\mathcal{E}_c e^{-i(\omega_b + \omega_c)t} + \frac{1}{4}\Xi_{abc}(-\omega_b, -\omega_c)\mathcal{E}_b\mathcal{E}_c e^{i(\omega_b + \omega_c)t}\Big] \\ = &\sigma_{ab}(\omega_b)\mathcal{E}_b \cos(\omega_b t) + \tilde{\sigma}_{ab}(\omega_b)\mathcal{E}_b \sin(\omega_b t) + \xi_{abc}\mathcal{E}_b\mathcal{E}_c \cos[(\omega_b - \omega_c)t] + \tilde{\xi}_{abc}\mathcal{E}_b\mathcal{E}_c \sin[(\omega_b - \omega_c)t] \\ &+ \chi_{abc}\mathcal{E}_b\mathcal{E}_c \cos[(\omega_b + \omega_c)t] + \tilde{\chi}_{abc}\mathcal{E}_b\mathcal{E}_c \sin[(\omega_b + \omega_c)t], \end{aligned} \tag{4}$$

where

$$\sigma_{ab}(\omega_b) = \frac{1}{2}\mathrm{Re}\big[\Pi_{ab}(\omega_b) + \Pi_{ab}(-\omega_b)\big], \qquad \tilde{\sigma}_{ab}(\omega_b) = \frac{1}{2}\mathrm{Im}\big[\Pi_{ab}(\omega_b) - \Pi_{ab}(-\omega_b)\big], \tag{5}$$

$$\xi_{abc}(\omega_b, \omega_c) = \frac{1}{8}\mathrm{Re}\big[\Xi_{abc}(\omega_b, -\omega_c) + \Xi_{abc}(-\omega_b, \omega_c)\big], \qquad \tilde{\xi}_{abc}(\omega_b, \omega_c) = \frac{1}{8}\mathrm{Im}\big[\Xi_{abc}(\omega_b, -\omega_c) - \Xi_{abc}(-\omega_b, \omega_c)\big], \tag{6}$$

$$\chi_{abc}(\omega_b, \omega_c) = \frac{1}{8}\mathrm{Re}\big[\Xi_{abc}(\omega_b, \omega_c) + \Xi_{abc}(-\omega_b, -\omega_c)\big], \qquad \tilde{\chi}_{abc}(\omega_b, \omega_c) = \frac{1}{8}\mathrm{Im}\big[\Xi_{abc}(\omega_b, \omega_c) - \Xi_{abc}(-\omega_b, -\omega_c)\big], \tag{7}$$

represent the in-phase (dissipative) and out-of-phase (reactive) linear and quadratic responses to the driving electric field. Only the in-phase responses can survive in the *dc* limit.

A. Feynman rules

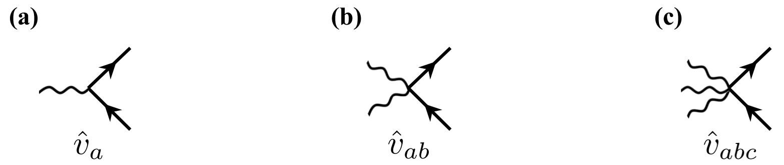

FIG. 1. Vertexes including the multi-photon processes.

Consider a generic system coupled with an electromagnetic field via the vector potential gauge, we have

$$\hat{\mathcal{H}} = \hat{\mathcal{H}}_0(\boldsymbol{k} + e\boldsymbol{A}(t)/\hbar) \equiv \hat{\mathcal{H}}_0(\boldsymbol{k}) + \hat{\mathcal{H}}_c, \tag{8}$$

where $-e$ is the electric charge of the electron. Without loss of generality, the electric field is taken as $E_a(t) = \mathcal{E}_a e^{-i\omega_a t}$, thus the vector potential $A_a(t) = -i\mathcal{E}_a e^{-i\omega_a t}/\omega_a = -iE_a(t)/\omega_a$ and $\hat{\mathcal{H}}_c$ contains the interaction between the total electric field and the particles of the system as

$$\hat{\mathcal{H}}_c = \frac{e}{i}\frac{\hat{v}_a}{\omega_a}E_a + \frac{1}{2!}\Big(\frac{e}{i}\Big)^2\frac{\hat{v}_{ab}}{\omega_a\omega_b}E_aE_b + \frac{1}{3!}\Big(\frac{e}{i}\Big)^3\frac{\hat{v}_{abc}}{\omega_a\omega_b\omega_c}E_aE_bE_c + \cdots, \tag{9}$$

where the velocity operator $\hat{v}_a$ and its tensor generalizations $\hat{v}_{ab}$ and $\hat{v}_{abc}$ read

$$\hat{v}_a = \frac{1}{\hbar}\partial^a_{\boldsymbol{k}}\hat{\mathcal{H}}_0, \qquad \hat{v}_{ab} = \frac{1}{\hbar^2}\partial^b_{\boldsymbol{k}}\partial^a_{\boldsymbol{k}}\hat{\mathcal{H}}_0, \qquad \hat{v}_{abc} = \frac{1}{\hbar^3}\partial^c_{\boldsymbol{k}}\partial^b_{\boldsymbol{k}}\partial^a_{\boldsymbol{k}}\hat{\mathcal{H}}_0. \tag{10}$$

On the other hand, the conductivity tensor of the system is determined by the ensemble average of the current operator $\hat{\boldsymbol{J}}$, which can be obtained as

$$\hat{J}_a = -e\hat{v}_a - i\Big(\frac{e}{i}\Big)^2\frac{\hat{v}_{ab}}{\omega_b}E_b - \frac{i}{2!}\Big(\frac{e}{i}\Big)^3\frac{\hat{v}_{abc}}{\omega_b\omega_c}E_bE_c - \cdots. \tag{11}$$

The above result indicates that the output vertices have different coefficients with the input vertices. A detailed derivation of the Feynman rules can be found in [7]. In order to correctly compute the second order nonlinear conductivities, we expand the Hamiltonian and the current operator up to third order, which includes the vertex shown in Supplementary Fig. 1. It can be shown that Supplementary Eq. (10) is consistent with the velocity operators generated by the covariant derivative in the eigenstate basis [7].

B. Generic formula of the quadratic conductivity

To construct a generic nonlinear response formula, we consider a generic Hamiltonian for a noninteracting multi-band system that can be formally diagonalized as

$$\hat{\mathcal{H}}_0(\boldsymbol{k}) \Rightarrow \sum_{\eta} \varepsilon_{\boldsymbol{k}}^{\eta} |u_{\boldsymbol{k}}^{\eta}\rangle\langle u_{\boldsymbol{k}}^{\eta}|, \tag{12}$$

where η is the label of band, $\varepsilon_{\boldsymbol{k}}^{\eta}$ and $|u_{\boldsymbol{k}}^{\eta}\rangle$ are the eigenenergy and eigenstate of the η-band.

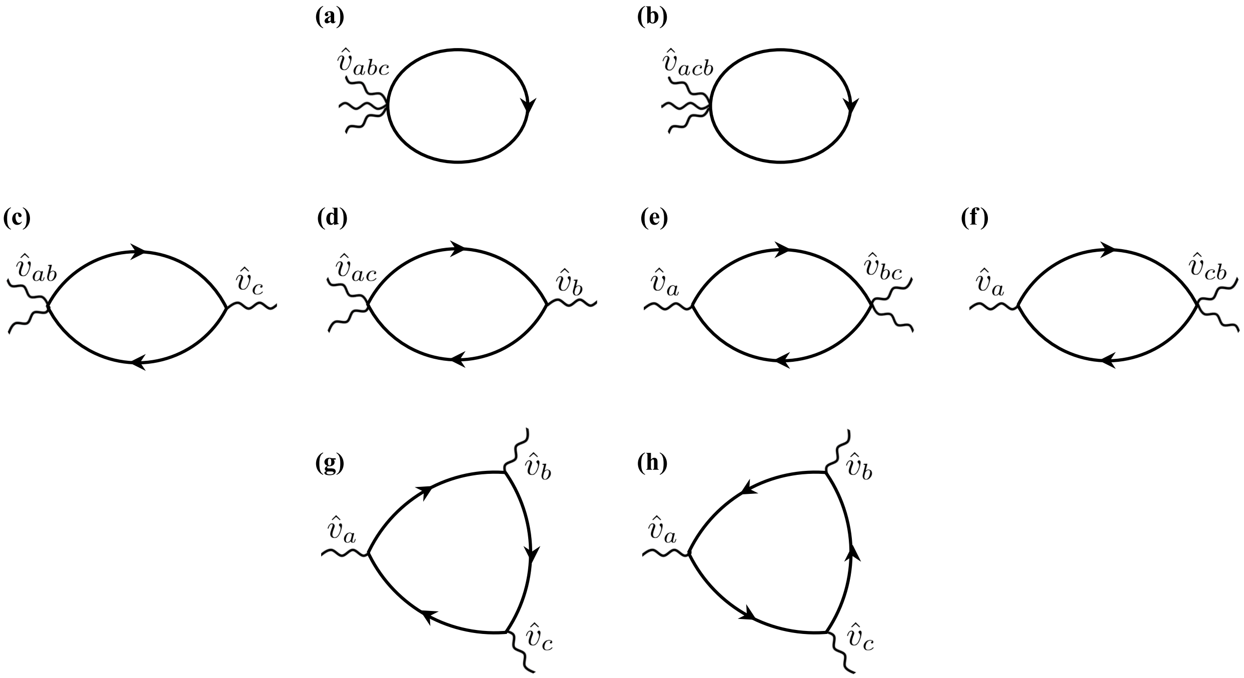

FIG. 2. Feynman diagrams of the quadratic conductivity.

According to the Feynman rules in the vector potential gauge, there are eight Feynman diagrams for the quadratic conductivity as shown in Supplementary Fig. 2. The diagrams are in pairs, which insures the $b \leftrightarrow c$ symmetry of the final results. Denoting the contribution of the diagrams Supplementary Fig. 2 (a) and (b) as $\mathcal{D}_{(a)}(\omega_b, \omega_c)$ and $\mathcal{D}_{(b)}(\omega_b, \omega_c)$, we have

$$\mathcal{D}_{(a)}(\omega_b, \omega_c) + \mathcal{D}_{(b)}(\omega_b, \omega_c) = \frac{ie^3}{2\omega_b\omega_c}\frac{1}{\beta}\int [dk] \sum_n \mathrm{Tr}[\hat{v}_{abc}\hat{\mathcal{G}}(i\omega_n)] + b \leftrightarrow c, \tag{13}$$

$$\mathcal{D}_{(c)}(\omega_b, \omega_c) + \mathcal{D}_{(d)}(\omega_b, \omega_c) = \frac{ie^3}{\omega_b\omega_c}\frac{1}{\beta}\int [dk] \sum_n \mathrm{Tr}[\hat{v}_{ab}\hat{\mathcal{G}}(i\omega_n + i\nu_c)\hat{v}_c\hat{\mathcal{G}}(i\omega_n)] + b \leftrightarrow c, \tag{14}$$

$$\mathcal{D}_{(e)}(\omega_b, \omega_c) + \mathcal{D}_{(f)}(\omega_b, \omega_c) = \frac{ie^3}{2\omega_b\omega_c}\frac{1}{\beta}\int [dk] \sum_n \mathrm{Tr}[\hat{v}_a\hat{\mathcal{G}}(i\omega_n + i\nu_b + i\nu_c)\hat{v}_{bc}\hat{\mathcal{G}}(i\omega_n)] + b \leftrightarrow c, \tag{15}$$

$$\mathcal{D}_{(g)}(\omega_b, \omega_c) + \mathcal{D}_{(h)}(\omega_b, \omega_c) = \frac{ie^3}{\omega_b\omega_c}\frac{1}{\beta}\int [dk] \sum_n \mathrm{Tr}[\hat{v}_a\hat{\mathcal{G}}(i\omega_n + i\nu_b + i\nu_c)\hat{v}_b\hat{\mathcal{G}}(i\omega_n + i\nu_c)\hat{v}_c\hat{\mathcal{G}}(i\omega_n)] + b \leftrightarrow c, \tag{16}$$

where ω_b and ω_c are frequencies of the driving electric field at b and c vertex, $\int [dk] \equiv \int d^n k/(2\pi)^n$ with n representing the dimension of the system,

$$\hat{\mathcal{G}}(i\omega_n) = \frac{1}{i\omega_n - \hat{\mathcal{H}}_0}. \tag{17}$$

is the Matsubara Green's function, ω_n and $\nu_{b,c}$ are the fermionic and bosonic Matsubara frequencies. Performing analytic continuation, we obtain the contributions in terms of the retarded and advanced Green's functions along the

real frequency axis

$$\mathcal{D}_{(a)}(\omega_b,\omega_c)+\mathcal{D}_{(b)}(\omega_b,\omega_c)=\frac{ie^3}{4\pi\omega_b\omega_c}\int[dk]\int_{-\infty}^{\infty}d\varepsilon f(\varepsilon)\mathrm{Tr}\big\{\hat{v}_{abc}[\hat{G}^R(\varepsilon)-\hat{G}^A(\varepsilon)]\big\}+b\leftrightarrow c, \tag{18}$$

$$\begin{aligned}\mathcal{D}_{(c)}(\omega_b,\omega_c)+\mathcal{D}_{(d)}(\omega_b,\omega_c)=\frac{ie^3}{2\pi\omega_b\omega_c}\int[dk]\int_{-\infty}^{\infty}d\varepsilon f(\varepsilon)\mathrm{Tr}\big\{&\hat{v}_{ab}\hat{G}^R(\varepsilon+\hbar\omega_c)\hat{v}_c[\hat{G}^R(\varepsilon)-\hat{G}^A(\varepsilon)]\\&+\hat{v}_{ab}[\hat{G}^R(\varepsilon)-\hat{G}^A(\varepsilon)]\hat{v}_c\hat{G}^A(\varepsilon-\hbar\omega_c)\big\}+b\leftrightarrow c,\end{aligned} \tag{19}$$

$$\begin{aligned}\mathcal{D}_{(e)}(\omega_b,\omega_c)+\mathcal{D}_{(f)}(\omega_b,\omega_c)=\frac{ie^3}{4\pi\omega_b\omega_c}\int[dk]\int_{-\infty}^{\infty}d\varepsilon f(\varepsilon)\mathrm{Tr}\big\{&\hat{v}_a\hat{G}^R(\varepsilon+\hbar\omega_b+\hbar\omega_c)\hat{v}_{bc}[\hat{G}^R(\varepsilon)-\hat{G}^A(\varepsilon)]\\&+\hat{v}_a[\hat{G}^R(\varepsilon)-\hat{G}^A(\varepsilon)]\hat{v}_{bc}\hat{G}^A(\varepsilon-\hbar\omega_b-\hbar\omega_c)\big\}+b\leftrightarrow c,\end{aligned} \tag{20}$$

$$\begin{aligned}\mathcal{D}_{(g)}(\omega_b,\omega_c)+\mathcal{D}_{(h)}(\omega_b,\omega_c)=\frac{ie^3}{2\pi\omega_b\omega_c}\int[dk]\int_{-\infty}^{\infty}d\varepsilon f(\varepsilon)\mathrm{Tr}\big\{&\hat{v}_a\hat{G}^R(\varepsilon+\hbar\omega_b+\hbar\omega_c)\hat{v}_b\hat{G}^R(\varepsilon+\hbar\omega_c)\hat{v}_c[\hat{G}^R(\varepsilon)-\hat{G}^A(\varepsilon)]\\&+\hat{v}_a\hat{G}^R(\varepsilon+\hbar\omega_b)\hat{v}_b[\hat{G}^R(\varepsilon)-\hat{G}^A(\varepsilon)]\hat{v}_c\hat{G}^A(\varepsilon-\hbar\omega_c)\\&+\hat{v}_a[\hat{G}^R(\varepsilon)-\hat{G}^A(\varepsilon)]\hat{v}_b\hat{G}^A(\varepsilon-\hbar\omega_b)\hat{v}_c\hat{G}^A(\varepsilon-\hbar\omega_b-\hbar\omega_c)\big\}+b\leftrightarrow c,\end{aligned} \tag{21}$$

where we have taken $i\nu_{b,c}\to\hbar\omega_{b,c}+i\delta$. And the quadratic conductivity can be obtained as a summation of all the diagrams

$$\Xi_{abc}(\omega_b,\omega_c)=\sum_{i=a}^{h}\mathcal{D}_{(i)}(\omega_b,\omega_c). \tag{22}$$

Noting the relation between the velocity operators [Supplementary Eq. (10)] and the identity

$$\begin{aligned}\partial_{\boldsymbol{k}}^a\mathrm{Tr}(\hat{\mathcal{O}})&=\partial_{\boldsymbol{k}}^a\sum_\eta\langle u_{\boldsymbol{k}}^\eta|\hat{\mathcal{O}}|u_{\boldsymbol{k}}^\eta\rangle\\&=\sum_\eta\Big[\langle u_{\boldsymbol{k}}^\eta|\partial_{\boldsymbol{k}}^a\hat{\mathcal{O}}|u_{\boldsymbol{k}}^\eta\rangle+\langle\partial_{\boldsymbol{k}}^a u_{\boldsymbol{k}}^\eta|\hat{\mathcal{O}}|u_{\boldsymbol{k}}^\eta\rangle+\langle u_{\boldsymbol{k}}^\eta|\hat{\mathcal{O}}|\partial_{\boldsymbol{k}}^a u_{\boldsymbol{k}}^\eta\rangle\Big]\\&=\mathrm{Tr}(\partial_{\boldsymbol{k}}^a\hat{\mathcal{O}})+\sum_{\eta\eta'}\Big[\langle\partial_{\boldsymbol{k}}^a u_{\boldsymbol{k}}^\eta|u_{\boldsymbol{k}}^{\eta'}\rangle\langle u_{\boldsymbol{k}}^{\eta'}|\hat{\mathcal{O}}|u_{\boldsymbol{k}}^\eta\rangle+\langle u_{\boldsymbol{k}}^\eta|\hat{\mathcal{O}}|u_{\boldsymbol{k}}^{\eta'}\rangle\langle u_{\boldsymbol{k}}^{\eta'}|\partial_{\boldsymbol{k}}^a u_{\boldsymbol{k}}^\eta\rangle\Big]\\&=\mathrm{Tr}(\partial_{\boldsymbol{k}}^a\hat{\mathcal{O}})-\sum_{\eta\eta'}\Big[\langle u_{\boldsymbol{k}}^{\eta'}|\hat{\mathcal{O}}|u_{\boldsymbol{k}}^\eta\rangle\langle u_{\boldsymbol{k}}^\eta|\partial_{\boldsymbol{k}}^a u_{\boldsymbol{k}}^{\eta'}\rangle-\langle u_{\boldsymbol{k}}^\eta|\hat{\mathcal{O}}|u_{\boldsymbol{k}}^{\eta'}\rangle\langle u_{\boldsymbol{k}}^{\eta'}|\partial_{\boldsymbol{k}}^a u_{\boldsymbol{k}}^\eta\rangle\Big]\\&=\mathrm{Tr}(\partial_{\boldsymbol{k}}^a\hat{\mathcal{O}}),\end{aligned} \tag{23}$$

we can apply the integration by parts to Supplementary Eq. (18) and (19) and obtain

$$\begin{aligned}\mathcal{D}_{(a)}(\omega_b,\omega_c)+\mathcal{D}_{(b)}(\omega_b,\omega_c)=\frac{ie^3}{4\pi\omega_b\omega_c}\int[dk]\int_{-\infty}^{\infty}d\varepsilon f(\varepsilon)\mathrm{Tr}\big\{&\hat{v}_a[2\hat{G}^R(\varepsilon)\hat{v}_b\hat{G}^R(\varepsilon)\hat{v}_c\hat{G}^R(\varepsilon)+\hat{G}^R(\varepsilon)\hat{v}_{bc}\hat{G}^R(\varepsilon)\\&-2\hat{G}^A(\varepsilon)\hat{v}_b\hat{G}^A(\varepsilon)\hat{v}_c\hat{G}^A(\varepsilon)-\hat{G}^A(\varepsilon)\hat{v}_{bc}\hat{G}^A(\varepsilon)]\big\}+b\leftrightarrow c,\end{aligned} \tag{24}$$

$$\begin{aligned}\mathcal{D}_{(c)}(\omega_b,\omega_c)+\mathcal{D}_{(d)}(\omega_b,\omega_c)=-\frac{ie^3}{2\pi\omega_b\omega_c}\int[dk]\int_{-\infty}^{\infty}d\varepsilon f(\varepsilon)\mathrm{Tr}\Big\{&\hat{v}_a\big\{\hat{G}^R(\varepsilon+\hbar\omega_c)\hat{v}_b\hat{G}^R(\varepsilon+\hbar\omega_c)\hat{v}_c[\hat{G}^R(\varepsilon)-\hat{G}^A(\varepsilon)]\\&+\hat{G}^R(\varepsilon+\hbar\omega_c)\hat{v}_{bc}[\hat{G}^R(\varepsilon)-\hat{G}^A(\varepsilon)]+\hat{G}^R(\varepsilon+\hbar\omega_b)\hat{v}_b[\hat{G}^R(\varepsilon)\hat{v}_c\hat{G}^R(\varepsilon)-\hat{G}^A(\varepsilon)\hat{v}_c\hat{G}^A(\varepsilon)]\\&+[\hat{G}^R(\varepsilon)\hat{v}_b\hat{G}^R(\varepsilon)-\hat{G}^A(\varepsilon)\hat{v}_b\hat{G}^A(\varepsilon)]\hat{v}_c\hat{G}^A(\varepsilon-\hbar\omega_c)+[\hat{G}^R(\varepsilon)-\hat{G}^A(\varepsilon)]\hat{v}_{bc}\hat{G}^A(\varepsilon-\hbar\omega_c)\\&+[\hat{G}^R(\varepsilon)-\hat{G}^A(\varepsilon)]\hat{v}_b\hat{G}^A(\varepsilon-\hbar\omega_b)\hat{v}_c\hat{G}^A(\varepsilon-\hbar\omega_b)\big\}\Big\}+b\leftrightarrow c,\end{aligned} \tag{25}$$

where we have performed several $b\leftrightarrow c$ for some terms and used the identity

$$\frac{1}{\hbar}\partial_{\boldsymbol{k}}^a\hat{G}^{R/A}(\varepsilon)=\hat{G}^{R/A}(\varepsilon)\hat{v}_b\hat{G}^{R/A}(\varepsilon). \tag{26}$$

Then, for all the contributions, we only have two types of terms, the first type is proportional to $\hat{v}_a\hat{v}_{bc}$ and the second type is proportional to $\hat{v}_a\hat{v}_b\hat{v}_c$. Thus, we can write that

$$\Xi_{abc}(\omega_b,\omega_c)=\mathcal{M}(\omega_b,\omega_c)+\mathcal{S}(\omega_b,\omega_c). \tag{27}$$

$\mathcal{M}(\omega_b,\omega_c)$ are the terms proportional to $\hat{v}_a\hat{v}_{bc}$, which can be obtained as

$$\begin{aligned}\mathcal{M}(\omega_b,\omega_c) =& \frac{ie^3}{4\pi\omega_b\omega_c}\int[dk]\int_{-\infty}^{\infty}d\varepsilon f(\varepsilon)\mathrm{Tr}\Big\{\hat{v}_a\big\{\hat{G}^R(\varepsilon)\hat{v}_{bc}\hat{G}^R(\varepsilon)-\hat{G}^A(\varepsilon)\hat{v}_{bc}\hat{G}^A(\varepsilon)\\ &-2\hat{G}^R(\varepsilon+\hbar\omega_c)\hat{v}_{bc}[\hat{G}^R(\varepsilon)-\hat{G}^A(\varepsilon)]-2[\hat{G}^R(\varepsilon)-\hat{G}^A(\varepsilon)]\hat{v}_{bc}\hat{G}^A(\varepsilon-\hbar\omega_c)\\ &+\hat{G}^R(\varepsilon+\hbar\omega_b+\hbar\omega_c)\hat{v}_{bc}[\hat{G}^R(\varepsilon)-\hat{G}^A(\varepsilon)]+[\hat{G}^R(\varepsilon)-\hat{G}^A(\varepsilon)]\hat{v}_{bc}\hat{G}^A(\varepsilon-\hbar\omega_b-\hbar\omega_c)\big\}\Big\}+b\leftrightarrow c\\ =& \mathcal{M}^{RR}(\omega_b,\omega_c)+\mathcal{M}^{AA}(\omega_b,\omega_c)+\mathcal{M}^{RA}(\omega_b,\omega_c). \end{aligned} \tag{28}$$

$\mathcal{M}^{RR}(\omega_b,\omega_c)$, $\mathcal{M}^{AA}(\omega_b,\omega_c)$, and $\mathcal{M}^{RA}(\omega_b,\omega_c)$ represent the terms that are proportional to $\hat{G}^R\hat{G}^R$, $\hat{G}^A\hat{G}^A$, and $\hat{G}^R\hat{G}^A$, respectively. Thus, we have

$$\begin{aligned}\mathcal{M}^{RR}(\omega_b,\omega_c) =& \frac{ie^3}{4\pi\omega_b\omega_c}\int[dk]\int_{-\infty}^{\infty}d\varepsilon f(\varepsilon)\mathrm{Tr}\Big\{\hat{v}_a\big[\hat{G}^R(\varepsilon)-\hat{G}^R(\varepsilon+\hbar\omega_b)-\hat{G}^R(\varepsilon+\hbar\omega_c)\\ &+\hat{G}^R(\varepsilon+\hbar\omega_b+\hbar\omega_c)\big]\hat{v}_{bc}\hat{G}^R(\varepsilon)\Big\}+b\leftrightarrow c, \end{aligned} \tag{29}$$

$$\begin{aligned}\mathcal{M}^{AA}(\omega_b,\omega_c) =& -\frac{ie^3}{4\pi\omega_b\omega_c}\int[dk]\int_{-\infty}^{\infty}d\varepsilon f(\varepsilon)\mathrm{Tr}\Big\{\hat{v}_a\hat{G}^A(\varepsilon)\hat{v}_{bc}\big[\hat{G}^A(\varepsilon)-\hat{G}^A(\varepsilon-\hbar\omega_b)-\hat{G}^A(\varepsilon-\hbar\omega_c)\\ &+\hat{G}^A(\varepsilon-\hbar\omega_b-\hbar\omega_c)\big]\Big\}+b\leftrightarrow c, \end{aligned} \tag{30}$$

$$\begin{aligned}\mathcal{M}^{RA}(\omega_b,\omega_c) =& \frac{ie^3}{4\pi\omega_b\omega_c}\int[dk]\int_{-\infty}^{\infty}d\varepsilon\mathrm{Tr}\Big\{\hat{v}_a\big\{[f(\varepsilon)-f(\varepsilon+\hbar\omega_b)]\hat{G}^R(\varepsilon+\hbar\omega_b)\\ &-[f(\varepsilon)-f(\varepsilon+\hbar\omega_b+\hbar\omega_c)]\hat{G}^R(\varepsilon+\hbar\omega_b+\hbar\omega_c)\\ &+[f(\varepsilon)-f(\varepsilon+\hbar\omega_c)]\hat{G}^R(\varepsilon+\hbar\omega_c)\big\}\hat{v}_{bc}\hat{G}^A(\varepsilon)\Big\}+b\leftrightarrow c \end{aligned} \tag{31}$$

$$\begin{aligned} =& \frac{ie^3}{4\pi\omega_b\omega_c}\int[dk]\int_{-\infty}^{\infty}d\varepsilon\mathrm{Tr}\Big\{\hat{v}_a\hat{G}^R(\varepsilon)\hat{v}_{bc}\big\{[f(\varepsilon-\hbar\omega_b)-f(\varepsilon)]\hat{G}^A(\varepsilon-\hbar\omega_b)\\ &-[f(\varepsilon-\hbar\omega_b-\hbar\omega_c)-f(\varepsilon)]\hat{G}^A(\varepsilon-\hbar\omega_b-\hbar\omega_c)\\ &+[f(\varepsilon-\hbar\omega_c)-f(\varepsilon)]\hat{G}^A(\varepsilon-\hbar\omega_c)\big\}\Big\}+b\leftrightarrow c. \end{aligned} \tag{32}$$

In the dc limit, we can expand the Green's functions as

$$\lim_{\omega\to 0}\hat{G}^{R/A}(\varepsilon+\hbar\omega)\simeq\hat{G}^{R/A}(\varepsilon)+\hbar\omega\frac{\partial\hat{G}^{R/A}(\varepsilon)}{\partial\varepsilon}+\frac{\hbar^2\omega^2}{2}\frac{\partial^2\hat{G}^{R/A}(\varepsilon)}{\partial\varepsilon^2}. \tag{33}$$

With this expansion, we can obtain that

$$\mathcal{M}^{RR}=\frac{ie^3\hbar^2}{4\pi}\int[dk]\int_{-\infty}^{\infty}d\varepsilon f(\varepsilon)\mathrm{Tr}\Big[\hat{v}_a\frac{\partial^2\hat{G}^R(\varepsilon)}{\partial\varepsilon^2}\hat{v}_{bc}\hat{G}^R(\varepsilon)\Big]+b\leftrightarrow c, \tag{34}$$

$$\mathcal{M}^{AA}=-\frac{ie^3\hbar^2}{4\pi}\int[dk]\int_{-\infty}^{\infty}d\varepsilon f(\varepsilon)\mathrm{Tr}\Big[\hat{v}_a\hat{G}^A(\varepsilon)\hat{v}_{bc}\frac{\partial^2\hat{G}^A(\varepsilon)}{\partial\varepsilon^2}\Big]+b\leftrightarrow c, \tag{35}$$

$$\mathcal{M}^{RA}=\frac{ie^3\hbar^2}{4\pi}\int[dk]\int_{-\infty}^{\infty}d\varepsilon\frac{\partial f(\varepsilon)}{\partial\varepsilon}\mathrm{Tr}\Big\{\hat{v}_a\big[\frac{\partial\hat{G}^R(\varepsilon)}{\partial\varepsilon}\hat{v}_{bc}\hat{G}^A(\varepsilon)-\hat{G}^R(\varepsilon)\hat{v}_{bc}\frac{\partial\hat{G}^A(\varepsilon)}{\partial\varepsilon}\big]\Big\}+b\leftrightarrow c, \tag{36}$$

and their combination reads

$$\begin{aligned}\mathcal{M}=& \frac{ie^3\hbar^2}{4\pi}\int[dk]\int_{-\infty}^{\infty}d\varepsilon f(\varepsilon)\mathrm{Tr}\Big[\hat{v}_a\frac{\partial^2\hat{G}^R(\varepsilon)}{\partial\varepsilon^2}\hat{v}_{bc}\hat{G}^R(\varepsilon)-\hat{v}_a\hat{G}^A(\varepsilon)\hat{v}_{bc}\frac{\partial^2\hat{G}^A(\varepsilon)}{\partial\varepsilon^2}\\ &+\hat{v}_a\frac{\partial\hat{G}^R(\varepsilon)}{\partial\varepsilon}\hat{v}_{bc}\hat{G}^A(\varepsilon)-\hat{v}_a\hat{G}^R(\varepsilon)\hat{v}_{bc}\frac{\partial\hat{G}^A(\varepsilon)}{\partial\varepsilon}\Big]+b\leftrightarrow c. \end{aligned} \tag{37}$$

$\mathcal{S}(\omega_b,\omega_c)$ are the terms proportional to $\hat{v}_a\hat{v}_b\hat{v}_c$, which can be obtained as

$$
\begin{aligned}
\mathcal{S}(\omega_b,\omega_c) = & \frac{ie^3}{2\pi\omega_b\omega_c}\int[dk]\int_{-\infty}^{\infty} d\varepsilon f(\varepsilon)\mathrm{Tr}\Big\{\hat{v}_a\{\hat{G}^R(\varepsilon)\hat{v}_b\hat{G}^R(\varepsilon)\hat{v}_c\hat{G}^R(\varepsilon)-\hat{G}^A(\varepsilon)\hat{v}_b\hat{G}^A(\varepsilon)\hat{v}_c\hat{G}^A(\varepsilon)\\
&-\hat{G}^R(\varepsilon+\hbar\omega_c)\hat{v}_b\hat{G}^R(\varepsilon+\hbar\omega_c)\hat{v}_c[\hat{G}^R(\varepsilon)-\hat{G}^A(\varepsilon)]-\hat{G}^R(\varepsilon+\hbar\omega_b)\hat{v}_b[\hat{G}^R(\varepsilon)\hat{v}_c\hat{G}^R(\varepsilon)-\hat{G}^A(\varepsilon)\hat{v}_c\hat{G}^A(\varepsilon)]\\
&-[\hat{G}^R(\varepsilon)\hat{v}_b\hat{G}^R(\varepsilon)-\hat{G}^A(\varepsilon)\hat{v}_b\hat{G}^A(\varepsilon)]\hat{v}_c\hat{G}^A(\varepsilon-\hbar\omega_c)-[\hat{G}^R(\varepsilon)-\hat{G}^A(\varepsilon)]\hat{v}_b\hat{G}^A(\varepsilon-\hbar\omega_b)\hat{v}_c\hat{G}^A(\varepsilon-\hbar\omega_b)\\
&+\hat{G}^R(\varepsilon+\hbar\omega_b+\hbar\omega_c)\hat{v}_b\hat{G}^R(\varepsilon+\hbar\omega_c)\hat{v}_c[\hat{G}^R(\varepsilon)-\hat{G}^A(\varepsilon)]+\hat{G}^R(\varepsilon+\hbar\omega_b)\hat{v}_b[\hat{G}^R(\varepsilon)-\hat{G}^A(\varepsilon)]\hat{v}_c\hat{G}^A(\varepsilon-\hbar\omega_c)\\
&+[\hat{G}^R(\varepsilon)-\hat{G}^A(\varepsilon)]\hat{v}_b\hat{G}^A(\varepsilon-\hbar\omega_b)\hat{v}_c\hat{G}^A(\varepsilon-\hbar\omega_b-\hbar\omega_c)\}\Big\}+b\leftrightarrow c\\
=&\ \mathcal{S}^{RRR}(\omega_b,\omega_c)+\mathcal{S}^{AAA}(\omega_b,\omega_c)+\mathcal{S}^{RRA}(\omega_b,\omega_c)+\mathcal{S}^{RAA}(\omega_b,\omega_c).
\end{aligned}
\tag{38}
$$

Thus, we have

$$
\begin{aligned}
\mathcal{S}^{RRR}(\omega_b,\omega_c) =& \frac{ie^3}{2\pi\omega_b\omega_c}\int[dk]\int_{-\infty}^{\infty} d\varepsilon f(\varepsilon)\mathrm{Tr}\Big\{\hat{v}_a\big[\hat{G}^R(\varepsilon)\hat{v}_b\hat{G}^R(\varepsilon)\hat{v}_c\hat{G}^R(\varepsilon)-\hat{G}^R(\varepsilon+\hbar\omega_c)\hat{v}_b\hat{G}^R(\varepsilon+\hbar\omega_c)\hat{v}_c\hat{G}^R(\varepsilon)\\
&-\hat{G}^R(\varepsilon+\hbar\omega_b)\hat{v}_b\hat{G}^R(\varepsilon)\hat{v}_c\hat{G}^R(\varepsilon)+\hat{G}^R(\varepsilon+\hbar\omega_b+\hbar\omega_c)\hat{v}_b\hat{G}^R(\varepsilon+\hbar\omega_c)\hat{v}_c\hat{G}^R(\varepsilon)\big]\Big\}+b\leftrightarrow c,
\end{aligned}
\tag{39}
$$

$$
\begin{aligned}
\mathcal{S}^{AAA}(\omega_b,\omega_c) =& -\frac{ie^3}{2\pi\omega_b\omega_c}\int[dk]\int_{-\infty}^{\infty} d\varepsilon f(\varepsilon)\mathrm{Tr}\Big\{\hat{v}_a\big[\hat{G}^A(\varepsilon)\hat{v}_b\hat{G}^A(\varepsilon)\hat{v}_c\hat{G}^A(\varepsilon)-\hat{G}^A(\varepsilon)\hat{v}_b\hat{G}^A(\varepsilon)\hat{v}_c\hat{G}^A(\varepsilon-\hbar\omega_c)\\
&-\hat{G}^A(\varepsilon)\hat{v}_b\hat{G}^A(\varepsilon-\hbar\omega_b)\hat{v}_c\hat{G}^A(\varepsilon-\hbar\omega_b)+\hat{G}^A(\varepsilon)\hat{v}_b\hat{G}^A(\varepsilon-\hbar\omega_b)\hat{v}_c\hat{G}^A(\varepsilon-\hbar\omega_b-\hbar\omega_c)\big]\Big\}+b\leftrightarrow c,
\end{aligned}
\tag{40}
$$

$$
\begin{aligned}
\mathcal{S}^{RRA}(\omega_b,\omega_c) =& \frac{ie^3}{2\pi\omega_b\omega_c}\int[dk]\int_{-\infty}^{\infty} d\varepsilon[f(\varepsilon)-f(\varepsilon+\hbar\omega_c)]\mathrm{Tr}\Big\{\hat{v}_a\big[\hat{G}^R(\varepsilon+\hbar\omega_c)\hat{v}_b\hat{G}^R(\varepsilon+\hbar\omega_c)\hat{v}_c\hat{G}^A(\varepsilon)\\
&-\hat{G}^R(\varepsilon+\hbar\omega_b+\hbar\omega_c)\hat{v}_b\hat{G}^R(\varepsilon+\hbar\omega_c)\hat{v}_c\hat{G}^A(\varepsilon)\big]\Big\}+b\leftrightarrow c,
\end{aligned}
\tag{41}
$$

$$
\begin{aligned}
\mathcal{S}^{RAA}(\omega_b,\omega_c) =& \frac{ie^3}{2\pi\omega_b\omega_c}\int[dk]\int_{-\infty}^{\infty} d\varepsilon[f(\varepsilon-\hbar\omega_b)-f(\varepsilon)]\mathrm{Tr}\Big\{\hat{v}_a\big[\hat{G}^R(\varepsilon)\hat{v}_b\hat{G}^A(\varepsilon-\hbar\omega_b)\hat{v}_c\hat{G}^A(\varepsilon-\hbar\omega_b)\\
&-\hat{G}^R(\varepsilon)\hat{v}_b\hat{G}^A(\varepsilon-\hbar\omega_b)\hat{v}_c\hat{G}^A(\varepsilon-\hbar\omega_b-\hbar\omega_c)\big]\Big\}+b\leftrightarrow c.
\end{aligned}
\tag{42}
$$

In the dc limit, the distribution function can also be expanded as

$$
\lim_{\omega\to 0} f(\varepsilon+\hbar\omega)\simeq f(\varepsilon)+\hbar\omega\frac{\partial f(\varepsilon)}{\partial\varepsilon}+\frac{\hbar^2\omega^2}{2}\frac{\partial^2 f(\varepsilon)}{\partial\varepsilon^2}.
\tag{43}
$$

With the expansions Supplementary Eq. (33) and (43), we can obtain that

$$
\mathcal{S}^{RRR} = \frac{ie^3\hbar^2}{2\pi}\int[dk]\int_{-\infty}^{\infty} d\varepsilon f(\varepsilon)\mathrm{Tr}\Big\{\hat{v}_a\frac{\partial}{\partial\varepsilon}\Big[\frac{\partial\hat{G}^R(\varepsilon)}{\partial\varepsilon}\hat{v}_b\hat{G}^R(\varepsilon)\Big]\hat{v}_c\hat{G}^R(\varepsilon)\Big\}+b\leftrightarrow c,
\tag{44}
$$

$$
\mathcal{S}^{AAA} = -\frac{ie^3\hbar^2}{2\pi}\int[dk]\int_{-\infty}^{\infty} d\varepsilon f(\varepsilon)\mathrm{Tr}\Big\{\hat{v}_a\hat{G}^A(\varepsilon)\hat{v}_b\frac{\partial}{\partial\varepsilon}\Big[\hat{G}^A(\varepsilon)\hat{v}_c\frac{\partial\hat{G}^A(\varepsilon)}{\partial\varepsilon}\Big]\Big\}+b\leftrightarrow c,
\tag{45}
$$

$$
\mathcal{S}^{RRA} = \frac{ie^3\hbar^2}{2\pi}\int[dk]\int_{-\infty}^{\infty} d\varepsilon\frac{\partial f(\varepsilon)}{\partial\varepsilon}\mathrm{Tr}\Big[\hat{v}_a\frac{\partial\hat{G}^R(\varepsilon)}{\partial\varepsilon}\hat{v}_b\hat{G}^R(\varepsilon)\hat{v}_c\hat{G}^A(\varepsilon)\Big]+b\leftrightarrow c,
\tag{46}
$$

$$
\mathcal{S}^{RAA} = -\frac{ie^3\hbar^2}{2\pi}\int[dk]\int_{-\infty}^{\infty} d\varepsilon\frac{\partial f(\varepsilon)}{\partial\varepsilon}\mathrm{Tr}\Big[\hat{v}_a\hat{G}^R(\varepsilon)\hat{v}_b\hat{G}^A(\varepsilon)\hat{v}_c\frac{\partial\hat{G}^A(\varepsilon)}{\partial\varepsilon}\Big]+b\leftrightarrow c,
\tag{47}
$$

and their combination reads

$$
\begin{aligned}
\mathcal{S} =& \frac{ie^3\hbar^2}{2\pi}\int[dk]\int_{-\infty}^{\infty} d\varepsilon\Big\{f(\varepsilon)\mathrm{Tr}\Big\{\hat{v}_a\frac{\partial}{\partial\varepsilon}\Big[\frac{\partial\hat{G}^R(\varepsilon)}{\partial\varepsilon}\hat{v}_b\hat{G}^R(\varepsilon)\Big]\hat{v}_c\hat{G}^R(\varepsilon)-\hat{v}_a\hat{G}^A(\varepsilon)\hat{v}_b\frac{\partial}{\partial\varepsilon}\Big[\hat{G}^A(\varepsilon)\hat{v}_c\frac{\partial\hat{G}^A(\varepsilon)}{\partial\varepsilon}\Big]\Big\}\\
&+\frac{\partial f(\varepsilon)}{\partial\varepsilon}\mathrm{Tr}\Big[\hat{v}_a\frac{\partial\hat{G}^R(\varepsilon)}{\partial\varepsilon}\hat{v}_b\hat{G}^R(\varepsilon)\hat{v}_c\hat{G}^A(\varepsilon)-\hat{v}_a\hat{G}^R(\varepsilon)\hat{v}_b\hat{G}^A(\varepsilon)\hat{v}_c\frac{\partial\hat{G}^A(\varepsilon)}{\partial\varepsilon}\Big]\Big\}+b\leftrightarrow c.
\end{aligned}
\tag{48}
$$

Thus, the total quadratic conductivity in the dc limit reads

$$\begin{aligned}\Xi_{abc} =& \frac{ie^3\hbar^2}{2\pi}\int[dk]\int_{-\infty}^{\infty}d\varepsilon\Bigg\{f(\varepsilon)\mathrm{Tr}\Big\{\frac{1}{2}\Big[\hat{v}_a\frac{\partial^2\hat{G}^R(\varepsilon)}{\partial\varepsilon^2}\hat{v}_{bc}\hat{G}^R(\varepsilon)-\hat{v}_a\hat{G}^A(\varepsilon)\hat{v}_{bc}\frac{\partial^2\hat{G}^A(\varepsilon)}{\partial\varepsilon^2}\Big]\\&+\hat{v}_a\frac{\partial}{\partial\varepsilon}\Big[\frac{\partial\hat{G}^R(\varepsilon)}{\partial\varepsilon}\hat{v}_b\hat{G}^R(\varepsilon)\Big]\hat{v}_c\hat{G}^R(\varepsilon)-\hat{v}_a\hat{G}^A(\varepsilon)\hat{v}_b\frac{\partial}{\partial\varepsilon}\Big[\hat{G}^A(\varepsilon)\hat{v}_c\frac{\partial\hat{G}^A(\varepsilon)}{\partial\varepsilon}\Big]\Big\}\\&+\frac{\partial f(\varepsilon)}{\partial\varepsilon}\mathrm{Tr}\Big\{\frac{1}{2}\Big[\hat{v}_a\frac{\partial\hat{G}^R(\varepsilon)}{\partial\varepsilon}\hat{v}_{bc}\hat{G}^A(\varepsilon)-\hat{v}_a\hat{G}^R(\varepsilon)\hat{v}_{bc}\frac{\partial\hat{G}^A(\varepsilon)}{\partial\varepsilon}\Big]\\&+\hat{v}_a\frac{\partial\hat{G}^R(\varepsilon)}{\partial\varepsilon}\hat{v}_b\hat{G}^R(\varepsilon)\hat{v}_c\hat{G}^A(\varepsilon)-\hat{v}_a\hat{G}^R(\varepsilon)\hat{v}_b\hat{G}^A(\varepsilon)\hat{v}_c\frac{\partial\hat{G}^A(\varepsilon)}{\partial\varepsilon}\Big\}\Bigg\}+b\leftrightarrow c\\=&\ \Xi^I_{abc}+\Xi^{II}_{abc}+\Xi^{III}_{abc},\end{aligned}\tag{49}$$

where

$$\Xi^I_{abc}=-\frac{e^3\hbar^2}{\pi}\int[dk]\int_{-\infty}^{\infty}d\varepsilon\frac{\partial f(\varepsilon)}{\partial\varepsilon}\mathrm{Im}\Big\{\mathrm{Tr}\Big[\hat{v}_a\frac{\partial\hat{G}^R(\varepsilon)}{\partial\varepsilon}\hat{v}_b\hat{G}^R(\varepsilon)\hat{v}_c\hat{G}^A(\varepsilon)\Big]\Big\}+b\leftrightarrow c,\tag{50}$$

$$\Xi^{II}_{abc}=-\frac{e^3\hbar^2}{2\pi}\int[dk]\int_{-\infty}^{\infty}d\varepsilon\frac{\partial f(\varepsilon)}{\partial\varepsilon}\mathrm{Im}\Big\{\mathrm{Tr}\Big[\hat{v}_a\frac{\partial\hat{G}^R(\varepsilon)}{\partial\varepsilon}\hat{v}_{bc}\hat{G}^A(\varepsilon)\Big]\Big\}+b\leftrightarrow c,\tag{51}$$

$$\begin{aligned}\Xi^{III}_{abc}=&-\frac{e^3\hbar^2}{2\pi}\int[dk]\int_{-\infty}^{\infty}d\varepsilon f(\varepsilon)\mathrm{Im}\Bigg\{\mathrm{Tr}\Big\{\hat{v}_a\frac{\partial^2\hat{G}^R(\varepsilon)}{\partial\varepsilon^2}\hat{v}_{bc}\hat{G}^R(\varepsilon)\\&+2\hat{v}_a\frac{\partial}{\partial\varepsilon}\Big[\frac{\partial\hat{G}^R(\varepsilon)}{\partial\varepsilon}\hat{v}_b\hat{G}^R(\varepsilon)\Big]\hat{v}_c\hat{G}^R(\varepsilon)\Big\}\Bigg\}+b\leftrightarrow c,\end{aligned}\tag{52}$$

and $\mathrm{Im}(\hat{\mathcal{O}})\equiv(\hat{\mathcal{O}}-\hat{\mathcal{O}}^\dagger)/2i$. Here, Ξ^I_{abc} and Ξ^{II}_{abc} represent the Fermi surface contributions, while Ξ^{III}_{abc} represent the Fermi sea contribution. In Ξ^{III}_{abc}, every term depends on the products of $\hat{G}^R$ only or $\hat{G}^A$ only. It can be shown that terms in Ξ^{III}_{abc} are smaller by a factor of $1/(\tau\varepsilon_F)\ll 1$ than terms in Ξ^I_{abc} and Ξ^{II}_{abc} in the weak disorder limit [8], we can hence neglect the Fermi sea contributions to the low-frequency transports.

C. Diagrammatic correspondence of the generic formula

The generic quadratic conductivity formula Supplementary Eqs. (50)-(52) result from the combination of all the diagrams shown in Supplementary Fig. 2, which do not have obvious diagrammatic correspondence in a standard sense. Thus, it is inconvenient to perform further diagrammatic calculations based on these formula, especially when one tries to calculate the individual intrinsic, side-jump or skew-scattering contribution in the eigenstate basis. Here we construct a diagrammatic correspondence of the generic formula Supplementary Eq. (50) and (51), which is useful for further diagrammatic calculations in the eigenstate basis.

According to Supplementary Eq. (50), Ξ^I_{abc} is proportional to the vertex functions products $\hat{v}_a\hat{v}_b\hat{v}_c$, which is mainly contributed from the triangular diagrams Supplementary Fig. 2 (g) and (h). Noting the Fermi surface contribution from these two diagrams are

$$\begin{aligned}\mathcal{S}_{g\text{-}h}(\omega_b,\omega_c)=&\frac{ie^3}{2\pi\omega_b\omega_c}\int[dk]\int_{-\infty}^{\infty}d\varepsilon\mathrm{Tr}\Big\{[f(\varepsilon+\hbar\omega_c)-f(\varepsilon)]\hat{v}_a\hat{G}^R(\varepsilon+\hbar\omega_b+\hbar\omega_c)\hat{v}_b\hat{G}^R(\varepsilon+\hbar\omega_c)\hat{v}_c\hat{G}^A(\varepsilon)\\&+[f(\varepsilon)-f(\varepsilon-\hbar\omega_b)]\hat{v}_a\hat{G}^R(\varepsilon)\hat{v}_b\hat{G}^A(\varepsilon-\hbar\omega_b)\hat{v}_c\hat{G}^A(\varepsilon-\hbar\omega_b-\hbar\omega_c)\Big\}+b\leftrightarrow c\\=&\frac{ie^3}{2\pi\omega_b\omega_c}\int[dk]\int_{-\infty}^{\infty}d\varepsilon\mathrm{Tr}\Big\{[f(\varepsilon+\hbar\omega_c)-f(\varepsilon)]\hat{v}_a\hat{G}^R(\varepsilon+\hbar\omega_b+\hbar\omega_c)\hat{v}_b\hat{G}^R(\varepsilon+\hbar\omega_c)\hat{v}_c\hat{G}^A(\varepsilon)\\&-[f(\varepsilon-\hbar\omega_c)-f(\varepsilon)]\hat{v}_a\hat{G}^R(\varepsilon)\hat{v}_c\hat{G}^A(\varepsilon-\hbar\omega_c)\hat{v}_b\hat{G}^A(\varepsilon-\hbar\omega_b-\hbar\omega_c)\Big\}+b\leftrightarrow c,\end{aligned}\tag{53}$$

where we have performed a $b\leftrightarrow c$ for the second term. Compared $\mathcal{S}_{g\text{-}h}(\omega_b,\omega_c)$ with $\mathcal{S}^{RRA}(\omega_b,\omega_c)$ and $\mathcal{S}^{RAA}(\omega_b,\omega_c)$, we can take $\omega_c\to 0$ and let $\omega_b=\omega$, then we have

$$\Xi^I_{abc}=\lim_{\omega\to 0}\frac{1}{\omega}[\tilde{\mathcal{D}}_{g\text{-}h}(\omega)-\tilde{\mathcal{D}}_{g\text{-}h}(0)]=\frac{\partial\tilde{\mathcal{D}}_{g\text{-}h}(\omega)}{\partial\omega}\Big|_{\omega=0},\tag{54}$$

where

$$\tilde{\mathcal{D}}_{g\text{-}h}(\omega) = \frac{ie^3\hbar}{2\pi}\int[dk]\int_{-\infty}^{\infty} d\varepsilon \frac{f(\varepsilon)}{\partial\varepsilon}\mathrm{Tr}\Big[\hat{v}_a\hat{G}^R(\varepsilon+\hbar\omega)\hat{v}_b\hat{G}^R(\varepsilon)\hat{v}_c\hat{G}^A(\varepsilon)$$
$$+\hat{v}_a\hat{G}^R(\varepsilon)\hat{v}_c\hat{G}^A(\varepsilon)\hat{v}_b\hat{G}^A(\varepsilon-\hbar\omega)\Big\} + b\leftrightarrow c$$
$$= \frac{ie^3\hbar}{2\pi}\int[dk]\int_{-\infty}^{\infty} d\varepsilon \frac{f(\varepsilon)}{\partial\varepsilon}\mathrm{Tr}\Big[\hat{v}_a\hat{G}^R(\varepsilon+\hbar\omega)\hat{v}_b\hat{G}^R(\varepsilon)\hat{v}_c\hat{G}^A(\varepsilon)$$
$$+\hat{v}_a\hat{G}^R(\varepsilon)\hat{v}_b\hat{G}^A(\varepsilon)\hat{v}_c\hat{G}^A(\varepsilon-\hbar\omega)\Big\} + b\leftrightarrow c, \tag{55}$$

and we again performed a $b \leftrightarrow c$ for the second term. As $\tilde{\mathcal{D}}_{g\text{-}h}(\omega)$ has a relatively simple and clear diagrammatic correspondence as shown in Supplementary Fig. 3 (a) and (b), the diagrammatic interpretation of Ξ^I_{abc} can be obtained accordingly.

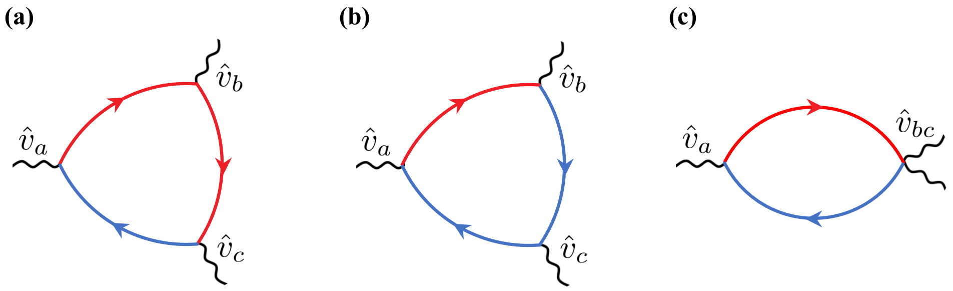

FIG. 3. Feynman diagrammatic interpretation of the quadratic conductivity Ξ^I_{abc} and Ξ^{II}_{abc}. The red and blue lines represent the retarded and advanced Green's functions, respectively.

According to Supplementary Eq. (51), Ξ^{II}_{abc} is proportional to the vertex functions products $\hat{v}_a\hat{v}_{bc}$, which mainly contributed from the two-photon diagrams Supplementary Fig. 2 (e) and (f). Thus, according to our previous analysis, we can also construct a diagrammatic interpretation of Ξ^{II}_{abc} as

$$\Xi^{II}_{abc} = \lim_{\omega\to 0}\frac{1}{\omega}[\tilde{\mathcal{D}}_{e\text{-}f}(\omega) - \tilde{\mathcal{D}}_{e\text{-}f}(0)] = \frac{\partial\tilde{\mathcal{D}}_{e\text{-}f}(\omega)}{\partial\omega}\Big|_{\omega=0}, \tag{56}$$

where

$$\tilde{\mathcal{D}}_{e\text{-}f}(\omega) = \frac{ie^3\hbar}{4\pi}\int[dk]\int_{-\infty}^{\infty} d\varepsilon \frac{f(\varepsilon)}{\partial\varepsilon}\mathrm{Tr}\Big[\hat{v}_a\hat{G}^R(\varepsilon+\hbar\omega)\hat{v}_{bc}\hat{G}^A(\varepsilon)$$
$$+\hat{v}_a\hat{G}^R(\varepsilon)\hat{v}_{bc}\hat{G}^A(\varepsilon-\hbar\omega)\Big] + b\leftrightarrow c, \tag{57}$$

and its diagrammatic correspondence is shown in Supplementary Fig. 3 (c).

D. Generic two-band model with disorder

A generic Hamiltonian for a noninteracting two-band system can be formally written as

$$\hat{\mathcal{H}}_g = h_{0\boldsymbol{k}} + h_{a\boldsymbol{k}}\sigma^a, \tag{58}$$

where $h_{0\boldsymbol{k}}$ and $h_{a\boldsymbol{k}}$ are analytical functions of $\boldsymbol{k}$ with $\{a,b,c\}\in\{x,y,z\}$. The dispersions of two energy bands are

$$\varepsilon^{\pm}_{\boldsymbol{k}} = h_{0\boldsymbol{k}} \pm h_{\boldsymbol{k}} \tag{59}$$

with

$$h_{\boldsymbol{k}} \equiv \sqrt{h^2_{x\boldsymbol{k}} + h^2_{y\boldsymbol{k}} + h^2_{z\boldsymbol{k}}}. \tag{60}$$

The eigen vectors of the upper and lower bands are $|\psi^{\pm}_{\boldsymbol{k}}\rangle=(e^{i\boldsymbol{k}\cdot\boldsymbol{r}}/\sqrt{\mathrm{Vol}})|u^{\pm}_{\boldsymbol{k}}\rangle$ with Vol denoting the general volume and

$$|u^{+}_{\boldsymbol{k}}\rangle=\begin{pmatrix}\cos\frac{\theta_{\boldsymbol{k}}}{2}\\ \sin\frac{\theta_{\boldsymbol{k}}}{2}e^{i\phi_{\boldsymbol{k}}}\end{pmatrix}=\frac{1}{N_{\boldsymbol{k}}}\begin{pmatrix}h_{z\boldsymbol{k}}+h_{\boldsymbol{k}}\\ h_{x\boldsymbol{k}}+ih_{y\boldsymbol{k}}\end{pmatrix},$$
$$|u^{-}_{\boldsymbol{k}}\rangle=\begin{pmatrix}\sin\frac{\theta_{\boldsymbol{k}}}{2}e^{-i\phi_{\boldsymbol{k}}}\\ -\cos\frac{\theta_{\boldsymbol{k}}}{2}\end{pmatrix}=\frac{1}{N_{\boldsymbol{k}}}\begin{pmatrix}h_{x\boldsymbol{k}}-ih_{y\boldsymbol{k}}\\ -h_{z\boldsymbol{k}}-h_{\boldsymbol{k}}\end{pmatrix},\tag{61}$$

with the normalization factor

$$N_{\boldsymbol{k}}\equiv\sqrt{2h_{\boldsymbol{k}}(h_{\boldsymbol{k}}+h_{z\boldsymbol{k}})}.\tag{62}$$

From the expressions of eigen vectors, we can obtain that

$$\hat{h}_{x\boldsymbol{k}}\equiv\frac{h_{x\boldsymbol{k}}}{h_{\boldsymbol{k}}}=\sin\theta_{\boldsymbol{k}}\cos\phi_{\boldsymbol{k}},\qquad \hat{h}_{y\boldsymbol{k}}\equiv\frac{h_{y\boldsymbol{k}}}{h_{\boldsymbol{k}}}=\sin\theta_{\boldsymbol{k}}\sin\phi_{\boldsymbol{k}},\qquad \hat{h}_{z\boldsymbol{k}}\equiv\frac{h_{z\boldsymbol{k}}}{h_{\boldsymbol{k}}}=\cos\theta_{\boldsymbol{k}},\tag{63}$$

which can be equivalently rewritten as

$$\sin\theta_{\boldsymbol{k}}=\frac{h_{\perp\boldsymbol{k}}}{h_{\boldsymbol{k}}},\qquad\cos\theta_{\boldsymbol{k}}=\frac{h_{z\boldsymbol{k}}}{h_{\boldsymbol{k}}},\qquad\sin\phi_{\boldsymbol{k}}=\frac{h_{y\boldsymbol{k}}}{h_{\perp\boldsymbol{k}}},\qquad\cos\phi_{\boldsymbol{k}}=\frac{h_{x\boldsymbol{k}}}{h_{\perp\boldsymbol{k}}},\qquad h_{\perp\boldsymbol{k}}\equiv\sqrt{h^2_{x\boldsymbol{k}}+h^2_{y\boldsymbol{k}}}.\tag{64}$$

To simplify the expressions of some intermediate results, it is more convenient to transform the eigen vector of the lower band into an equivalent form as

$$|u^{-}_{\boldsymbol{k}}\rangle=\begin{pmatrix}\sin\frac{\theta_{\boldsymbol{k}}}{2}e^{-i\phi_{\boldsymbol{k}}}\\ -\cos\frac{\theta_{\boldsymbol{k}}}{2}\end{pmatrix}\Rightarrow\begin{pmatrix}\sin\frac{\theta_{\boldsymbol{k}}}{2}\\ -\cos\frac{\theta_{\boldsymbol{k}}}{2}e^{i\phi_{\boldsymbol{k}}}\end{pmatrix}.\tag{65}$$

The velocity operators along the two directions can be formally written as

$$\hat{v}_a=\frac{1}{\hbar}\partial^a_{\boldsymbol{k}}\hat{\mathcal{H}}_0=\frac{1}{\hbar}(\partial^a_{\boldsymbol{k}}h_{0\boldsymbol{k}}+\partial^a_{\boldsymbol{k}}h_{b\boldsymbol{k}}\sigma^b),\tag{66}$$

where $\partial^a_{\boldsymbol{k}}\equiv\partial/\partial k_a$. The corresponding matrix elements in the eigen basis are

$$v^{++}_{a\boldsymbol{k}}\equiv\langle u^+_{\boldsymbol{k}}|\hat{v}_a|u^+_{\boldsymbol{k}}\rangle=\frac{1}{\hbar}\Big[\partial^a_{\boldsymbol{k}}h_{0\boldsymbol{k}}+\frac{1}{h_{\boldsymbol{k}}}\Big(h_{x\boldsymbol{k}}\partial^a_{\boldsymbol{k}}h_{x\boldsymbol{k}}+h_{y\boldsymbol{k}}\partial^a_{\boldsymbol{k}}h_{y\boldsymbol{k}}+h_{z\boldsymbol{k}}\partial^a_{\boldsymbol{k}}h_{z\boldsymbol{k}}\Big)\Big]=\frac{1}{\hbar}\partial^a_{\boldsymbol{k}}\varepsilon^+_{\boldsymbol{k}},\tag{67}$$
$$v^{--}_{a\boldsymbol{k}}\equiv\langle u^-_{\boldsymbol{k}}|\hat{v}_a|u^-_{\boldsymbol{k}}\rangle=\frac{1}{\hbar}\Big[\partial^a_{\boldsymbol{k}}h_{0\boldsymbol{k}}-\frac{1}{h_{\boldsymbol{k}}}\Big(h_{x\boldsymbol{k}}\partial^a_{\boldsymbol{k}}h_{x\boldsymbol{k}}+h_{y\boldsymbol{k}}\partial^a_{\boldsymbol{k}}h_{y\boldsymbol{k}}+h_{z\boldsymbol{k}}\partial^a_{\boldsymbol{k}}h_{z\boldsymbol{k}}\Big)\Big]=\frac{1}{\hbar}\partial^a_{\boldsymbol{k}}\varepsilon^-_{\boldsymbol{k}},\tag{68}$$
$$v^{+-}_{a\boldsymbol{k}}\equiv\langle u^+_{\boldsymbol{k}}|\hat{v}_a|u^-_{\boldsymbol{k}}\rangle=-\frac{1}{\hbar}\Big[\Big(\frac{h_{x\boldsymbol{k}}h_{z\boldsymbol{k}}}{h_{\perp\boldsymbol{k}}h_{\boldsymbol{k}}}+i\frac{h_{y\boldsymbol{k}}}{h_{\perp\boldsymbol{k}}}\Big)\partial^a_{\boldsymbol{k}}h_{x\boldsymbol{k}}+\Big(\frac{h_{y\boldsymbol{k}}h_{z\boldsymbol{k}}}{h_{\perp\boldsymbol{k}}h_{\boldsymbol{k}}}-i\frac{h_{x\boldsymbol{k}}}{h_{\perp\boldsymbol{k}}}\Big)\partial^a_{\boldsymbol{k}}h_{y\boldsymbol{k}}-\frac{h_{\perp\boldsymbol{k}}}{h_{\boldsymbol{k}}}\partial^a_{\boldsymbol{k}}h_{z\boldsymbol{k}}\Big],\tag{69}$$
$$v^{-+}_{a\boldsymbol{k}}\equiv\langle u^-_{\boldsymbol{k}}|\hat{v}_a|u^+_{\boldsymbol{k}}\rangle=-\frac{1}{\hbar}\Big[\Big(\frac{h_{x\boldsymbol{k}}h_{z\boldsymbol{k}}}{h_{\perp\boldsymbol{k}}h_{\boldsymbol{k}}}-i\frac{h_{y\boldsymbol{k}}}{h_{\perp\boldsymbol{k}}}\Big)\partial^a_{\boldsymbol{k}}h_{x\boldsymbol{k}}+\Big(\frac{h_{y\boldsymbol{k}}h_{z\boldsymbol{k}}}{h_{\perp\boldsymbol{k}}h_{\boldsymbol{k}}}+i\frac{h_{x\boldsymbol{k}}}{h_{\perp\boldsymbol{k}}}\Big)\partial^a_{\boldsymbol{k}}h_{y\boldsymbol{k}}-\frac{h_{\perp\boldsymbol{k}}}{h_{\boldsymbol{k}}}\partial^a_{\boldsymbol{k}}h_{z\boldsymbol{k}}\Big],\tag{70}$$

and the Berry curvature for each band can be written as

$$\Omega^{ab}_{\pm}=\varepsilon^{abc}\Omega^c_{\pm}=\mp\frac{\varepsilon^{efg}}{2h^3_{\boldsymbol{k}}}h_{e\boldsymbol{k}}\partial^a_{\boldsymbol{k}}h_{f\boldsymbol{k}}\partial^b_{\boldsymbol{k}}h_{g\boldsymbol{k}}.\tag{71}$$

For the disorder part, we consider randomly located δ-function scatters at $\boldsymbol{R}_i$

$$\hat{V}_{imp}(\boldsymbol{r})=\sum_i V_i\delta(\boldsymbol{r}-\boldsymbol{R}_i).\tag{72}$$

The disorder strength V_i satisfies $\langle V_i\rangle=0$, $\langle V_i^2\rangle=V_0^2$, and $\langle V_i^3\rangle=V_1^3$. $\langle...\rangle$ means ensemble averaging over disorder configurations. In the eigenstate (chiral) representation, the operator elements of the disorder potential can be written in a general form as

$$\begin{aligned}V^{\eta\eta'}_{\boldsymbol{k}\boldsymbol{k}'}&=V^0_{\boldsymbol{k},\boldsymbol{k}'}\frac{i^{(\eta+\eta')/2-1}}{4}\Big[\Big(e^{i\theta_{\boldsymbol{k}}/2}+\eta e^{-i\theta_{\boldsymbol{k}}/2}\Big)\Big(e^{i\theta_{\boldsymbol{k}'}/2}+\eta' e^{-i\theta_{\boldsymbol{k}'}/2}\Big)\\&\quad-\Big(e^{i\theta_{\boldsymbol{k}}/2}-\eta e^{-i\theta_{\boldsymbol{k}}/2}\Big)\Big(e^{i\theta_{\boldsymbol{k}'}/2}-\eta' e^{-i\theta_{\boldsymbol{k}'}/2}\Big)e^{i(\phi_{\boldsymbol{k}'}-\phi_{\boldsymbol{k}})}\Big]\\&=V^0_{\boldsymbol{k},\boldsymbol{k}'}\frac{i^{(\eta+\eta')/2-1}}{4}\Big\{\Big[e^{i(\theta_{\boldsymbol{k}}+\theta_{\boldsymbol{k}'})/2}+\eta\eta' e^{-i(\theta_{\boldsymbol{k}}+\theta_{\boldsymbol{k}'})/2}\Big]\Big[1-e^{i(\phi_{\boldsymbol{k}'}-\phi_{\boldsymbol{k}})}\Big]\\&\quad+\Big[\eta e^{-i(\theta_{\boldsymbol{k}}-\theta_{\boldsymbol{k}'})/2}+\eta' e^{i(\theta_{\boldsymbol{k}}-\theta_{\boldsymbol{k}'})/2}\Big]\Big[1+e^{i(\phi_{\boldsymbol{k}'}-\phi_{\boldsymbol{k}})}\Big]\Big\},\end{aligned}\tag{73}$$

where $V^{\eta\eta'}_{\boldsymbol{k}\boldsymbol{k}'}\equiv\langle u^\eta_{\boldsymbol{k}}|\hat{V}_{imp}|u^{\eta'}_{\boldsymbol{k}'}\rangle$ with $\eta,\eta'\in\pm$ and $V^0_{\boldsymbol{k},\boldsymbol{k}'}\equiv\sum_i V_i e^{i(\boldsymbol{k}'-\boldsymbol{k})\boldsymbol{R}_i}$.

Supplementary Note 3. DIAGRAMMATICS: II. TRIANGULAR DIAGRAMS

In this section, we turn to the calculation of the disorder averaged Fermi surface contribution Ξ^I_{abc} to the nonlinear Hall effect. To obtain the intrinsic, side-jump, and skew-scattering contributions within the diagrammatic approach, we adopt the eigenstate basis and consider the disorder effect in the the Born approximation. According to the analysis in Sec. Supplementary Note 2 C, the effective diagrammatic interpretation of the triangular diagrams are plotted in Supplementary Fig. 4, which effectively connects the original triangular diagrams with Matsubara Green's functions to the effective diagrams with retarded (advanced) Green's functions. Within the Born approximation, the effect of disorder on the retarded (advanced) Green's functions is well known as [8]

$$\tilde{G}^{R/A}_{\eta\boldsymbol{k}}(\varepsilon) = \frac{1}{\varepsilon - \varepsilon^\eta_{\boldsymbol{k}} \pm i\Gamma^\eta_{\boldsymbol{k}}}, \tag{74}$$

where $\Gamma^\eta_{\boldsymbol{k}} \equiv \hbar/(2\tau^\eta_{\boldsymbol{k}})$ with the elastic scattering time $\tau^\eta_{\boldsymbol{k}}$. For the vertex correction of a generic two band model, as the iteration in the relevant vertex correction involves only intraband elements and does not produce unusual effects [9], we can formally write that $v^{\eta\eta}_a \Rightarrow \tilde{v}^{\eta\eta}_a$ with $\eta \in \pm$, where the specific form of $\tilde{v}^{\eta\eta}_a$ is determined by the model details. There is another type of disorder correction that only exists in nonlinear response processes, which we refer to as the edge correction $\tilde{v}^{++}_b v^{++}_c \Rightarrow \mathcal{W}^{++}_{bc}$ (see Sec. Supplementary Note 8 A 3 for a concrete example).

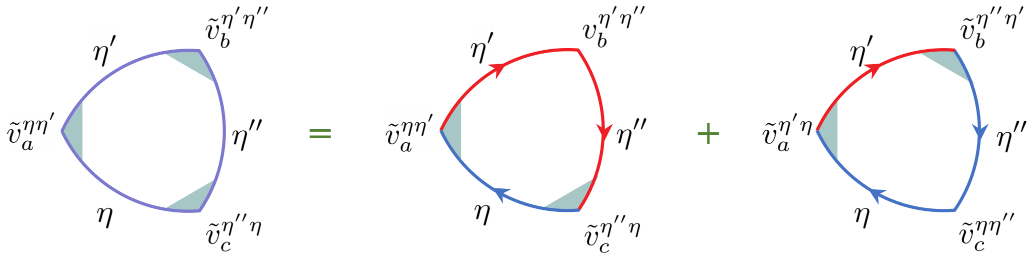

FIG. 4. Effective diagrammatic interpretation of the triangular diagram. The purple lines represent the Matsubara Green's function. The red and blue lines represent the retarded and advanced Green's functions, respectively. Within the effective diagrammatics, we only need to compute the clockwise diagrams, and anticlockwise diagrams are the $b \leftrightarrow c$ counterparts of the clockwise ones.

The disorder induced vertex/edge corrections can have ω-dependence. For example, the effect of disorder on the retarded-advanced vertex in the chiral basis is captured by the ladder diagrams. For band η, the vertex correction is given as

$$\begin{aligned}\tilde{v}^{\eta\eta}_{a\boldsymbol{k}}(\omega) &= v^{\eta\eta}_{a\boldsymbol{k}} + \int [dk'] \langle V^{\eta\eta}_{\boldsymbol{k}\boldsymbol{k}'} V^{\eta\eta}_{\boldsymbol{k}'\boldsymbol{k}} \rangle \tilde{G}^R_{\eta\boldsymbol{k}'}(\varepsilon + \hbar\omega) \tilde{v}^{\eta\eta}_{a\boldsymbol{k}'}(\omega) \tilde{G}^A_{\eta\boldsymbol{k}'}(\varepsilon) \\ &= v^{\eta\eta}_{a\boldsymbol{k}} + \frac{2\pi}{\hbar} \int [dk'] \frac{\tau^\eta_{\boldsymbol{k}'}}{1 - i\omega\tau^\eta_{\boldsymbol{k}'}} \langle V^{\eta\eta}_{\boldsymbol{k}\boldsymbol{k}'} V^{\eta\eta}_{\boldsymbol{k}'\boldsymbol{k}} \rangle \tilde{v}^{\eta\eta}_{a\boldsymbol{k}'}(\omega) \delta(\varepsilon - \varepsilon^\eta_{\boldsymbol{k}'}),\end{aligned} \tag{75}$$

where we have assumed $\hbar\omega \ll \varepsilon$ and used the identity Supplementary Eq. (220). Noting that the bare velocity $v^{\eta\eta}_{a\boldsymbol{k}}$ and the disorder potential $V^{\eta\eta}_{\boldsymbol{k}\boldsymbol{k}'}$ are both ω-independent, thus the only ω-dependence of the corrected velocity $\tilde{v}^{\eta\eta}_{a\boldsymbol{k}}(\omega)$ comes from the factor $\tau^\eta_{\boldsymbol{k}'}/(1 - i\omega\tau^\eta_{\boldsymbol{k}'})$. To gain some further insight, we multiply $\delta(\varepsilon - \varepsilon^\eta_{\boldsymbol{k}})$ on both sides of the above equation, and obtain that

$$\begin{aligned}\tilde{v}^{\eta\eta}_{a\boldsymbol{k}}(\omega) &= v^{\eta\eta}_{a\boldsymbol{k}} + \frac{2\pi}{\hbar} \int [dk'] \frac{\tau^\eta_{\boldsymbol{k}'}}{1 - i\omega\tau^\eta_{\boldsymbol{k}'}} \langle V^{\eta\eta}_{\boldsymbol{k}\boldsymbol{k}'} V^{\eta\eta}_{\boldsymbol{k}'\boldsymbol{k}} \rangle \tilde{v}^{\eta\eta}_{a\boldsymbol{k}'}(\omega) \delta(\varepsilon^\eta_{\boldsymbol{k}} - \varepsilon^\eta_{\boldsymbol{k}'}) \\ &= v^{\eta\eta}_{a\boldsymbol{k}} + \frac{A_{\boldsymbol{k}}}{1 - i\omega\tau B_{\boldsymbol{k}}} \tilde{v}^{\eta\eta}_{a\boldsymbol{k}}(\omega),\end{aligned} \tag{76}$$

where we have defined $\tau \equiv 1/n_i V_0^2$ and used the fact $\tau^\eta_{\boldsymbol{k}} \sim \tau$ and $\langle V^{\eta\eta}_{\boldsymbol{k}\boldsymbol{k}'} V^{\eta\eta}_{\boldsymbol{k}'\boldsymbol{k}} \rangle \sim 1/\tau$. Here A_k and B_k are some general real functions of order $(n_i V_0^2)^0$. Then, we can obtain a formal expression of the corrected vertex as

$$\tilde{v}^{\eta\eta}_{a\boldsymbol{k}}(\omega) = \frac{1 - i\omega\tau B_{\boldsymbol{k}}}{1 - A_{\boldsymbol{k}} - i\omega\tau B_{\boldsymbol{k}}} v^{\eta\eta}_{a\boldsymbol{k}}, \tag{77}$$

from which the ω-dependence of the corrected velocity can be seen. In the $\omega\tau \ll 1$ limit, the corrected velocity is nearly ω-independent. Under the small ω expansion we have

$$\tilde{v}^{\eta\eta}_{a\boldsymbol{k}}(\omega) \sim \tilde{v}^{\eta\eta}_{a\boldsymbol{k}} + i\omega\tau\delta\tilde{v}^{\eta\eta}_{a\boldsymbol{k}}, \tag{78}$$

where $\tilde{v}_{a\boldsymbol{k}}^{\eta\eta} = v_{a\boldsymbol{k}}^{\eta\eta}/(1 - A_{\boldsymbol{k}})$ is the disorder modified velocity in the dc limit, and $\delta\tilde{v}_{a\boldsymbol{k}}^{\eta\eta} = A_{\boldsymbol{k}}B_{\boldsymbol{k}}/(1 - A_{\boldsymbol{k}})$ is the leading ω-dependent modification to the velocity. The ω-dependent vertex/edge corrections are similar with the expansion of the factor $1/(1 - i\omega\tau)$ in diagrammatic calculations. One can check that these types of contribution vanishes for the triangular diagrams, thus we can neglect the ω-dependent terms of the vertex/edge corrections. However, these contributions can not be neglected in the two-photon process. We will discuss it in Sec. Supplementary Note 4.

A. Intrinsic contribution

Different from the anomalous Hall effect [10], the intrinsic contribution to the nonlinear Hall effect is disorder-dependent [1, 4]. Here the term "intrinsic" is still used to emphasize its geometric origin and the fact that no special scattering events (such as side-jump and skew-scattering) are involved. The Feynman diagrams of the intrinsic contribution to the nonlinear Hall effect are summarised in Supplementary Fig. 5. By denoting the diagrams in

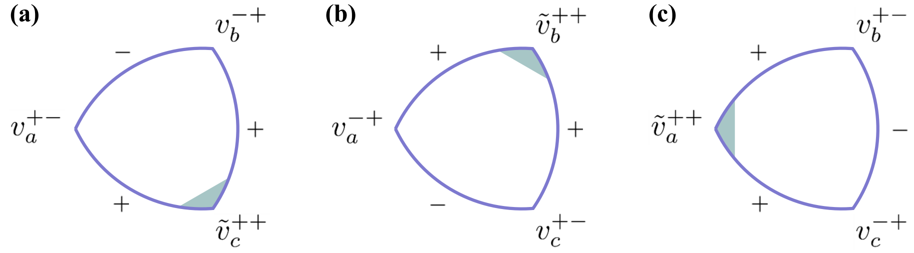

FIG. 5. Feynman diagrams of the intrinsic contribution to the nonlinear Hall effect.

Supplementary Fig. 5 (a)-(c) as $\tilde{\mathcal{D}}_{(a)}^{in}(\omega)$-$\tilde{\mathcal{D}}_{(c)}^{in}(\omega)$, we have

$$\begin{aligned}
\tilde{\mathcal{D}}_{(a)}^{in}(\omega) &= \frac{ie^3\hbar}{2\pi}\int[dk]\int_{-\infty}^{\infty} d\varepsilon f'(\varepsilon) v_{a\boldsymbol{k}}^{+-} v_{b\boldsymbol{k}}^{-+}\Big[\tilde{v}_{c\boldsymbol{k}}^{++}\tilde{G}_{-\boldsymbol{k}}^{R}(\varepsilon_1^+)\tilde{G}_{+\boldsymbol{k}}^{R}(\varepsilon)\tilde{G}_{+\boldsymbol{k}}^{A}(\varepsilon) \\
&\quad + v_{c\boldsymbol{k}}^{++}\tilde{G}_{-\boldsymbol{k}}^{R}(\varepsilon)\tilde{G}_{+\boldsymbol{k}}^{A}(\varepsilon)\tilde{G}_{+\boldsymbol{k}}^{A}(\varepsilon_1^-)\Big] + b \leftrightarrow c \\
&\simeq -e^3\int[dk]\int_{-\infty}^{\infty} d\varepsilon \tau_{\boldsymbol{k}}^{+} f'(\varepsilon_{\boldsymbol{k}}^{+})\frac{I_{ab}^{in}(\boldsymbol{k})\tilde{v}_{c\boldsymbol{k}}^{++}}{2h_{\boldsymbol{k}} + \hbar\omega} + b \leftrightarrow c, && (79) \\
\tilde{\mathcal{D}}_{(b)}^{in}(\omega) &= \frac{ie^3\hbar}{2\pi}\int[dk]\int_{-\infty}^{\infty} d\varepsilon f'(\varepsilon) v_{a\boldsymbol{k}}^{-+} v_{c\boldsymbol{k}}^{+-}\Big[v_{b\boldsymbol{k}}^{++}\tilde{G}_{+\boldsymbol{k}}^{R}(\varepsilon_1^+)\tilde{G}_{+\boldsymbol{k}}^{R}(\varepsilon)\tilde{G}_{-\boldsymbol{k}}^{A}(\varepsilon) \\
&\quad + \tilde{v}_{b\boldsymbol{k}}^{++}\tilde{G}_{+\boldsymbol{k}}^{R}(\varepsilon)\tilde{G}_{+\boldsymbol{k}}^{A}(\varepsilon)\tilde{G}_{-\boldsymbol{k}}^{A}(\varepsilon_1^-)\Big] + b \leftrightarrow c \\
&\simeq e^3\int[dk]\int_{-\infty}^{\infty} d\varepsilon \tau_{\boldsymbol{k}}^{+} f'(\varepsilon_{\boldsymbol{k}}^{+})\frac{I_{ac}^{in}(\boldsymbol{k})\tilde{v}_{b\boldsymbol{k}}^{++}}{2h_{\boldsymbol{k}} - \hbar\omega} + b \leftrightarrow c, && (80) \\
\tilde{\mathcal{D}}_{(c)}^{in}(\omega) &= \frac{ie^3\hbar}{2\pi}\int[dk]\int_{-\infty}^{\infty} d\varepsilon f'(\varepsilon) v_{b\boldsymbol{k}}^{+-} v_{c\boldsymbol{k}}^{-+}\Big[\tilde{v}_{a\boldsymbol{k}}^{++}\tilde{G}_{+\boldsymbol{k}}^{R}(\varepsilon_1^+)\tilde{G}_{-\boldsymbol{k}}^{R}(\varepsilon)\tilde{G}_{+\boldsymbol{k}}^{A}(\varepsilon) \\
&\quad + \tilde{v}_{a\boldsymbol{k}}^{++}\tilde{G}_{+\boldsymbol{k}}^{R}(\varepsilon)\tilde{G}_{-\boldsymbol{k}}^{A}(\varepsilon)\tilde{G}_{+\boldsymbol{k}}^{A}(\varepsilon_1^-)\Big] + b \leftrightarrow c \\
&\simeq -e^3\int[dk]\int_{-\infty}^{\infty} d\varepsilon \frac{\tau_{\boldsymbol{k}}^{+}}{1 - i\omega\tau_{\boldsymbol{k}}^{+}} f'(\varepsilon_{\boldsymbol{k}}^{+})\frac{\tilde{v}_{a\boldsymbol{k}}^{++} I_{bc}^{in}(\boldsymbol{k})}{h_{\boldsymbol{k}}} + b \leftrightarrow c \simeq 0, && (81)
\end{aligned}$$

where $\varepsilon_1^{\pm} \equiv \varepsilon \pm \hbar\omega$ and we only keep the leading order terms $[\sim (n_i V_0^2)^{-1}]$ that contribute to the nonlinear Hall response in time-reversal symmetric systems. And by $\simeq 0$ it means that the diagram has no leading order contribution.

Thus, the corresponding intrinsic contribution is

$$\Xi_{abc}^{I\text{-}in} = \sum_{i=a}^{c} \frac{\partial \tilde{\mathcal{D}}_{(i)}^{in}(\omega)}{\partial \omega}\Big|_{\omega=0}$$
$$= \frac{e^3\hbar}{2}\int [dk]\tau_{\boldsymbol{k}}^{+} f'(\varepsilon_{\boldsymbol{k}}^{+})\frac{I_{ab}^{in}(\boldsymbol{k})}{h_{\boldsymbol{k}}^2}\tilde{v}_{c\boldsymbol{k}}^{++} + b \leftrightarrow c$$
$$= -\frac{e^3}{\hbar}\int [dk]\tau_{\boldsymbol{k}}^{+} f'(\varepsilon_{\boldsymbol{k}}^{+})\varepsilon^{abd}\Omega_{d\boldsymbol{k}}^{+}\tilde{v}_{c\boldsymbol{k}}^{++} + b \leftrightarrow c, \tag{82}$$

And the intrinsic contribution to χ_{abc}^{I} in the dc limit is

$$\chi_{abc}^{I\text{-}in} = -\frac{e^3}{4\hbar}\int [dk]\tau_{\boldsymbol{k}}^{+} f'(\varepsilon_{\boldsymbol{k}}^{+})\varepsilon^{abd}\Omega_{d\boldsymbol{k}}^{+}\tilde{v}_{c\boldsymbol{k}}^{++} + b \leftrightarrow c. \tag{83}$$

B. Side-jump contribution

According to previous works on the anomalous [9, 11] and nonlinear Hall effects [4], the side-jump diagrams can be classified into two categories. The first one is due to the accumulation of the coordinate shifts after many scattering, thus corresponding to the diagrams with off-diagonal velocity elements in the output vertex (a), here we denote them as the anomalous velocity induced side-jump diagram with superscript $\mathcal{D}^{sj,1}$. The second one is due to the anomalous correction to the distribution function, thus corresponding to the diagrams with diagonal output vertex, that is, all of the off-diagonal velocity elements appear in the input vertex (b or c), here we denote them as the anomalous distribution induced side-jump diagram with superscript $\mathcal{D}^{sj,2}$.

1. Anomalous velocity induced side-jump contribution

The anomalous velocity induced side-jump diagrams are characterized with off-diagonal velocity on the output vertex, and are summarised in Supplementary Fig. 6. By denoting the diagrams in Supplementary Fig. 6 (a)-(f) as

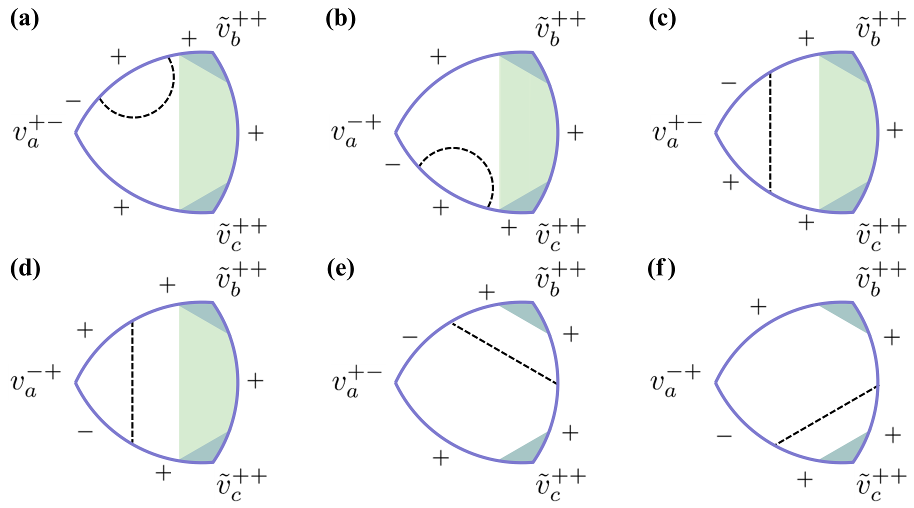

FIG. 6. Feynman diagrams of the anomalous velocity induced side-jump contribution to the nonlinear Hall effect.

$\tilde{\mathcal{D}}^{sj,1}_{(a)}(\omega)$-$\tilde{\mathcal{D}}^{sj,1}_{(f)}(\omega)$, we have

$$\begin{aligned}\tilde{\mathcal{D}}^{sj,1}_{(a)}(\omega) &= \frac{ie^3\hbar}{2\pi}\int[dk]\int[dk']\int_{-\infty}^{\infty}d\varepsilon f'(\varepsilon)v^{+-}_{a\boldsymbol{k}}\langle V^{-+}_{\boldsymbol{k}\boldsymbol{k}'}V^{++}_{\boldsymbol{k}'\boldsymbol{k}}\rangle\Big[\mathcal{W}^{++}_{cb\boldsymbol{k}}\tilde{G}^R_{-\boldsymbol{k}}(\varepsilon^+_1)\tilde{G}^R_{+\boldsymbol{k}'}(\varepsilon^+_1)\tilde{G}^R_{+\boldsymbol{k}}(\varepsilon^+_1)\tilde{G}^R_{+\boldsymbol{k}}(\varepsilon)\tilde{G}^A_{+\boldsymbol{k}}(\varepsilon)\\
&\quad+\mathcal{W}^{++}_{bc\boldsymbol{k}}\tilde{G}^R_{-\boldsymbol{k}}(\varepsilon)\tilde{G}^R_{+\boldsymbol{k}'}(\varepsilon)\tilde{G}^R_{+\boldsymbol{k}}(\varepsilon)\tilde{G}^A_{+\boldsymbol{k}}(\varepsilon)\tilde{G}^A_{+\boldsymbol{k}}(\varepsilon^-_1)\Big]+b\leftrightarrow c\\
&\simeq \frac{\pi e^3}{\hbar}\int[dk]\int[dk']\frac{(\tau^+_{\boldsymbol{k}})^2}{1-i\omega\tau^+_{\boldsymbol{k}}}f'(\varepsilon^+_{\boldsymbol{k}})I^{sj}_a(\boldsymbol{k},\boldsymbol{k}')\Big[\frac{\mathcal{W}^{++}_{cb\boldsymbol{k}}}{2h_{\boldsymbol{k}}+\hbar\omega}\delta(\varepsilon^+_{\boldsymbol{k}}-\varepsilon^+_{\boldsymbol{k}'}+\hbar\omega)\\
&\quad-\frac{\mathcal{W}^{++}_{bc\boldsymbol{k}}}{2h_{\boldsymbol{k}}}\delta(\varepsilon^+_{\boldsymbol{k}}-\varepsilon^+_{\boldsymbol{k}'})\Big]+b\leftrightarrow c,\end{aligned}\tag{84}$$

$$\begin{aligned}\tilde{\mathcal{D}}^{sj,1}_{(b)}(\omega) &= \frac{ie^3\hbar}{2\pi}\int[dk]\int[dk']\int_{-\infty}^{\infty}d\varepsilon f'(\varepsilon)v^{-+}_{a\boldsymbol{k}}\langle V^{++}_{\boldsymbol{k}\boldsymbol{k}'}V^{+-}_{\boldsymbol{k}'\boldsymbol{k}}\rangle\Big[\mathcal{W}^{++}_{cb\boldsymbol{k}}\tilde{G}^R_{+\boldsymbol{k}}(\varepsilon^+_1)\tilde{G}^R_{+\boldsymbol{k}}(\varepsilon)\tilde{G}^A_{+\boldsymbol{k}}(\varepsilon)\tilde{G}^A_{+\boldsymbol{k}'}(\varepsilon)\tilde{G}^A_{-\boldsymbol{k}}(\varepsilon)\\
&\quad+\mathcal{W}^{++}_{bc\boldsymbol{k}}\tilde{G}^R_{+\boldsymbol{k}}(\varepsilon)\tilde{G}^A_{+\boldsymbol{k}}(\varepsilon)\tilde{G}^A_{+\boldsymbol{k}}(\varepsilon^-_1)\tilde{G}^A_{+\boldsymbol{k}'}(\varepsilon^-_1)\tilde{G}^A_{-\boldsymbol{k}}(\varepsilon^-_1)\Big]+b\leftrightarrow c\\
&\simeq \frac{\pi e^3}{\hbar}\int[dk]\int[dk']\frac{(\tau^+_{\boldsymbol{k}})^2}{1-i\omega\tau^+_{\boldsymbol{k}}}f'(\varepsilon^+_{\boldsymbol{k}})I^{sj}_a(\boldsymbol{k},\boldsymbol{k}')\Big[\frac{\mathcal{W}^{++}_{cb\boldsymbol{k}}}{2h_{\boldsymbol{k}}}\delta(\varepsilon^+_{\boldsymbol{k}}-\varepsilon^+_{\boldsymbol{k}'})\\
&\quad-\frac{\mathcal{W}^{++}_{bc\boldsymbol{k}}}{2h_{\boldsymbol{k}}-\hbar\omega}\delta(\varepsilon^+_{\boldsymbol{k}}-\varepsilon^+_{\boldsymbol{k}'}-\hbar\omega)\Big]+b\leftrightarrow c,\end{aligned}\tag{85}$$

$$\begin{aligned}\tilde{\mathcal{D}}^{sj,1}_{(c)}(\omega) &= \frac{ie^3\hbar}{2\pi}\int[dk]\int[dk']\int_{-\infty}^{\infty}d\varepsilon f'(\varepsilon)v^{+-}_{a\boldsymbol{k}'}\langle V^{-+}_{\boldsymbol{k}'\boldsymbol{k}}V^{++}_{\boldsymbol{k}\boldsymbol{k}'}\rangle\Big[\mathcal{W}^{++}_{cb\boldsymbol{k}}\tilde{G}^R_{-\boldsymbol{k}'}(\varepsilon^+_1)\tilde{G}^R_{+\boldsymbol{k}}(\varepsilon^+_1)\tilde{G}^R_{+\boldsymbol{k}}(\varepsilon)\tilde{G}^A_{+\boldsymbol{k}}(\varepsilon)\tilde{G}^A_{+\boldsymbol{k}'}(\varepsilon)\\
&\quad+\mathcal{W}^{++}_{bc\boldsymbol{k}}\tilde{G}^R_{-\boldsymbol{k}'}(\varepsilon)\tilde{G}^R_{+\boldsymbol{k}}(\varepsilon)\tilde{G}^A_{+\boldsymbol{k}}(\varepsilon)\tilde{G}^A_{+\boldsymbol{k}}(\varepsilon^-_1)\tilde{G}^A_{+\boldsymbol{k}'}(\varepsilon^-_1)\Big]+b\leftrightarrow c\\
&\simeq -\frac{\pi e^3}{\hbar}\int[dk]\int[dk']\frac{(\tau^+_{\boldsymbol{k}})^2}{1-i\omega\tau^+_{\boldsymbol{k}}}f'(\varepsilon^+_{\boldsymbol{k}})I^{sj}_a(\boldsymbol{k}',\boldsymbol{k})\Big[\frac{\mathcal{W}^{++}_{cb\boldsymbol{k}}}{2h_{\boldsymbol{k}'}+\hbar\omega}\delta(\varepsilon^+_{\boldsymbol{k}}-\varepsilon^+_{\boldsymbol{k}'})\\
&\quad-\frac{\mathcal{W}^{++}_{bc\boldsymbol{k}}}{\varepsilon^+_{\boldsymbol{k}}-\varepsilon^-_{\boldsymbol{k}'}}\delta(\varepsilon^+_{\boldsymbol{k}}-\varepsilon^+_{\boldsymbol{k}'}-\hbar\omega)\Big]+b\leftrightarrow c,\end{aligned}\tag{86}$$

$$\begin{aligned}\tilde{\mathcal{D}}^{sj,1}_{(d)}(\omega) &= \frac{ie^3\hbar}{2\pi}\int[dk]\int[dk']\int_{-\infty}^{\infty}d\varepsilon f'(\varepsilon)v^{-+}_{a\boldsymbol{k}'}\langle V^{++}_{\boldsymbol{k}'\boldsymbol{k}}V^{+-}_{\boldsymbol{k}\boldsymbol{k}'}\rangle\Big[\mathcal{W}^{++}_{cb\boldsymbol{k}}\tilde{G}^R_{+\boldsymbol{k}'}(\varepsilon^+_1)\tilde{G}^R_{+\boldsymbol{k}}(\varepsilon^+_1)\tilde{G}^R_{+\boldsymbol{k}}(\varepsilon)\tilde{G}^A_{+\boldsymbol{k}}(\varepsilon)\tilde{G}^A_{-\boldsymbol{k}'}(\varepsilon)\\
&\quad+\mathcal{W}^{++}_{bc\boldsymbol{k}}\tilde{G}^R_{+\boldsymbol{k}'}(\varepsilon)\tilde{G}^R_{+\boldsymbol{k}}(\varepsilon)\tilde{G}^A_{+\boldsymbol{k}}(\varepsilon)\tilde{G}^A_{+\boldsymbol{k}}(\varepsilon^-_1)\tilde{G}^A_{-\boldsymbol{k}'}(\varepsilon^-_1)\Big]+b\leftrightarrow c\\
&\simeq -\frac{\pi e^3}{\hbar}\int[dk]\int[dk']\frac{(\tau^+_{\boldsymbol{k}})^2}{1-i\omega\tau^+_{\boldsymbol{k}}}f'(\varepsilon^+_{\boldsymbol{k}})I^{sj}_a(\boldsymbol{k}',\boldsymbol{k})\Big[\frac{\mathcal{W}^{++}_{cb\boldsymbol{k}}}{\varepsilon^+_{\boldsymbol{k}}-\varepsilon^-_{\boldsymbol{k}'}}\delta(\varepsilon^+_{\boldsymbol{k}}-\varepsilon^+_{\boldsymbol{k}'}+\hbar\omega)\\
&\quad-\frac{\mathcal{W}^{++}_{bc\boldsymbol{k}}}{2h_{\boldsymbol{k}'}-\hbar\omega}\delta(\varepsilon^+_{\boldsymbol{k}}-\varepsilon^+_{\boldsymbol{k}'})\Big]+b\leftrightarrow c,\end{aligned}\tag{87}$$

$$\begin{aligned}\tilde{\mathcal{D}}^{sj,1}_{(e)}(\omega) &= \frac{ie^3\hbar}{2\pi}\int[dk]\int[dk']\int_{-\infty}^{\infty}d\varepsilon f'(\varepsilon)v^{+-}_{a\boldsymbol{k}}\langle V^{-+}_{\boldsymbol{k}\boldsymbol{k}'}V^{++}_{\boldsymbol{k}'\boldsymbol{k}}\rangle\Big[v^{++}_{b\boldsymbol{k}'}\tilde{v}^{++}_{c\boldsymbol{k}}\tilde{G}^R_{-\boldsymbol{k}}(\varepsilon^+_1)\tilde{G}^R_{+\boldsymbol{k}'}(\varepsilon^+_1)\tilde{G}^R_{+\boldsymbol{k}'}(\varepsilon)\tilde{G}^R_{+\boldsymbol{k}}(\varepsilon)\tilde{G}^A_{+\boldsymbol{k}}(\varepsilon)\\
&\quad+\tilde{v}^{++}_{b\boldsymbol{k}'}v^{++}_{c\boldsymbol{k}}\tilde{G}^R_{-\boldsymbol{k}}(\varepsilon)\tilde{G}^R_{+\boldsymbol{k}'}(\varepsilon)\tilde{G}^A_{+\boldsymbol{k}'}(\varepsilon)\tilde{G}^A_{+\boldsymbol{k}}(\varepsilon)\tilde{G}^A_{+\boldsymbol{k}}(\varepsilon^-_1)\Big]+b\leftrightarrow c\\
&\simeq \frac{\pi e^3}{\hbar}\int[dk]\int[dk']\tau^+_{\boldsymbol{k}}\tau^+_{\boldsymbol{k}'}f'(\varepsilon^+_{\boldsymbol{k}})I^{sj}_a(\boldsymbol{k},\boldsymbol{k}')\Big[\frac{v^{++}_{b\boldsymbol{k}'}\tilde{v}^{++}_{c\boldsymbol{k}}}{(1-i\omega\tau^+_{\boldsymbol{k}'})(2h_{\boldsymbol{k}}+\hbar\omega)}\\
&\quad+\frac{\tilde{v}^{++}_{b\boldsymbol{k}'}v^{++}_{c\boldsymbol{k}}}{(1-i\omega\tau^+_{\boldsymbol{k}})2h_{\boldsymbol{k}}}\Big]\delta(\varepsilon^+_{\boldsymbol{k}}-\varepsilon^+_{\boldsymbol{k}'})+b\leftrightarrow c,\end{aligned}\tag{88}$$

$$\tilde{\mathcal{D}}^{sj,1}_{(f)}(\omega) = \frac{ie^3\hbar}{2\pi}\int[dk]\int[dk']\int_{-\infty}^{\infty} d\varepsilon f'(\varepsilon) v^{-+}_{a\boldsymbol{k}}\langle V^{++}_{\boldsymbol{k}\boldsymbol{k}'}V^{+-}_{\boldsymbol{k}'\boldsymbol{k}}\rangle\Big[v^{++}_{b\boldsymbol{k}}\tilde{v}^{++}_{c\boldsymbol{k}'}\tilde{G}^R_{+\boldsymbol{k}}(\varepsilon_1^+)\tilde{G}^R_{+\boldsymbol{k}}(\varepsilon)\tilde{G}^R_{+\boldsymbol{k}'}(\varepsilon)\tilde{G}^A_{+\boldsymbol{k}'}(\varepsilon)\tilde{G}^A_{-\boldsymbol{k}}(\varepsilon)$$
$$+\tilde{v}^{++}_{b\boldsymbol{k}}v^{++}_{c\boldsymbol{k}'}\tilde{G}^R_{+\boldsymbol{k}}(\varepsilon)\tilde{G}^A_{+\boldsymbol{k}}(\varepsilon)\tilde{G}^A_{+\boldsymbol{k}'}(\varepsilon)\tilde{G}^A_{+\boldsymbol{k}'}(\varepsilon_1^-)\tilde{G}^A_{-\boldsymbol{k}}(\varepsilon_1^-)\Big] + b\leftrightarrow c$$
$$\simeq -\frac{\pi e^3}{\hbar}\int[dk]\int[dk']\tau^+_{\boldsymbol{k}}\tau^+_{\boldsymbol{k}'}f'(\varepsilon^+_{\boldsymbol{k}})I^{sj}_a(\boldsymbol{k},\boldsymbol{k}')\Big[\frac{v^{++}_{b\boldsymbol{k}}\tilde{v}^{++}_{c\boldsymbol{k}'}}{(1-i\omega\tau^+_{\boldsymbol{k}})2h_{\boldsymbol{k}}}$$
$$+\frac{\tilde{v}^{++}_{b\boldsymbol{k}}v^{++}_{c\boldsymbol{k}'}}{(1-i\omega\tau^+_{\boldsymbol{k}'})(2h_{\boldsymbol{k}}-\hbar\omega)}\Big]\delta(\varepsilon^+_{\boldsymbol{k}}-\varepsilon^+_{\boldsymbol{k}'}) + b\leftrightarrow c, \tag{89}$$

where $\mathcal{W}^{++}_{bc\boldsymbol{k}}$ are the edge correction functions discussed in Sec. Supplementary Note 8 A 3. By combining all of the relevant terms, the anomalous velocity induced side-jump contribution can be obtained as

$$\Xi^{I\text{-}sj,1}_{abc} = \sum_{i=a}^{f}\frac{\partial\tilde{\mathcal{D}}^{sj,1}_{(i)}(\omega)}{\partial\omega}\Big|_{\omega=0}$$
$$= e^3\int[dk]\int[dk']\tau^+_{\boldsymbol{k}}f'(\varepsilon^+_{\boldsymbol{k}})\Big\{\tau^+_{\boldsymbol{k}}\mathcal{W}^{++}_{bc\boldsymbol{k}}\Big[V^{sj,0}_{a\boldsymbol{k}\boldsymbol{k}'}\delta'(\varepsilon^+_{\boldsymbol{k}}-\varepsilon^+_{\boldsymbol{k}'}) - V^{sj,1}_{a\boldsymbol{k}\boldsymbol{k}'}\delta(\varepsilon^+_{\boldsymbol{k}}-\varepsilon^+_{\boldsymbol{k}'})\Big]$$
$$-\frac{1}{2}\tau^+_{\boldsymbol{k}'}\tilde{v}^{++}_{b\boldsymbol{k}}v^{++}_{c\boldsymbol{k}'}(V^{sj,1}_{a\boldsymbol{k}\boldsymbol{k}'}+V^{sj,2}_{a\boldsymbol{k}\boldsymbol{k}'})\delta(\varepsilon^+_{\boldsymbol{k}}-\varepsilon^+_{\boldsymbol{k}'})\Big\} + b\leftrightarrow c, \tag{90}$$

And the anomalous velocity induced side-jump contribution to χ^I_{abc} in the dc limit is

$$\chi^{I\text{-}sj,1}_{abc} = \frac{e^3}{4}\int[dk]\int[dk']\tau^+_{\boldsymbol{k}}f'(\varepsilon^+_{\boldsymbol{k}})\Big\{\tau^+_{\boldsymbol{k}}\mathcal{W}^{++}_{bc\boldsymbol{k}}\Big[V^{sj,0}_{a\boldsymbol{k}\boldsymbol{k}'}\delta'(\varepsilon^+_{\boldsymbol{k}}-\varepsilon^+_{\boldsymbol{k}'}) - V^{sj,1}_{a\boldsymbol{k}\boldsymbol{k}'}\delta(\varepsilon^+_{\boldsymbol{k}}-\varepsilon^+_{\boldsymbol{k}'})\Big]$$
$$-\frac{1}{2}\tau^+_{\boldsymbol{k}'}\tilde{v}^{++}_{b\boldsymbol{k}}v^{++}_{c\boldsymbol{k}'}(V^{sj,1}_{a\boldsymbol{k}\boldsymbol{k}'}+V^{sj,2}_{a\boldsymbol{k}\boldsymbol{k}'})\delta(\varepsilon^+_{\boldsymbol{k}}-\varepsilon^+_{\boldsymbol{k}'})\Big\} + b\leftrightarrow c. \tag{91}$$

2. *Anomalous distribution induced side-jump contribution*

The anomalous distribution induced side-jump diagrams are summarised in Supplementary Fig. 7. By denoting the diagrams in Supplementary Fig. 7 (a)-(l) as $\tilde{\mathcal{D}}^{sj,2}_{(a)}(\omega)$-$\tilde{\mathcal{D}}^{sj,2}_{(l)}(\omega)$, we have

$$\mathcal{D}^{sj,2}_{(a)}(\omega) = \frac{ie^3\hbar}{2\pi}\int[dk]\int[dk']\int_{-\infty}^{\infty} d\varepsilon f'(\varepsilon) v^{+-}_{b\boldsymbol{k}}\langle V^{-+}_{\boldsymbol{k}\boldsymbol{k}'}V^{++}_{\boldsymbol{k}'\boldsymbol{k}}\rangle\Big[\tilde{v}^{++}_{a\boldsymbol{k}}\tilde{v}^{++}_{c\boldsymbol{k}}\tilde{G}^R_{+\boldsymbol{k}}(\varepsilon_1^+)\tilde{G}^R_{-\boldsymbol{k}}(\varepsilon)\tilde{G}^R_{+\boldsymbol{k}'}(\varepsilon)\tilde{G}^R_{+\boldsymbol{k}}(\varepsilon)\tilde{G}^A_{+\boldsymbol{k}}(\varepsilon)$$
$$+\mathcal{W}^{++}_{ac\boldsymbol{k}}\tilde{G}^R_{+\boldsymbol{k}}(\varepsilon)\tilde{G}^A_{-\boldsymbol{k}}(\varepsilon)\tilde{G}^A_{+\boldsymbol{k}'}(\varepsilon)\tilde{G}^A_{+\boldsymbol{k}}(\varepsilon)\tilde{G}^A_{+\boldsymbol{k}}(\varepsilon_1^-)\Big] + b\leftrightarrow c$$
$$\simeq \frac{\pi e^3}{\hbar}\int[dk]\int[dk']\frac{(\tau^+_{\boldsymbol{k}})^2}{1-i\omega\tau^+_{\boldsymbol{k}}}f'(\varepsilon^+_{\boldsymbol{k}})I^{sj}_b(\boldsymbol{k},\boldsymbol{k}')\Big[\frac{\tilde{v}^{++}_{a\boldsymbol{k}}\tilde{v}^{++}_{c\boldsymbol{k}}}{2h_{\boldsymbol{k}}}+\frac{\mathcal{W}^{++}_{ac\boldsymbol{k}}}{2h_{\boldsymbol{k}}}\Big]\delta(\varepsilon^+_{\boldsymbol{k}}-\varepsilon^+_{\boldsymbol{k}'}) + b\leftrightarrow c, \tag{92}$$

$$\mathcal{D}^{sj,2}_{(b)}(\omega) = \frac{ie^3\hbar}{2\pi}\int[dk]\int[dk']\int_{-\infty}^{\infty} d\varepsilon f'(\varepsilon) v^{-+}_{b\boldsymbol{k}}\langle V^{++}_{\boldsymbol{k}\boldsymbol{k}'}V^{+-}_{\boldsymbol{k}'\boldsymbol{k}}\rangle\Big[\tilde{v}^{++}_{a\boldsymbol{k}}\tilde{v}^{++}_{c\boldsymbol{k}}\tilde{G}^R_{+\boldsymbol{k}}(\varepsilon_1^+)\tilde{G}^R_{+\boldsymbol{k}'}(\varepsilon_1^+)\tilde{G}^R_{-\boldsymbol{k}}(\varepsilon_1^+)\tilde{G}^R_{+\boldsymbol{k}}(\varepsilon)\tilde{G}^A_{+\boldsymbol{k}}(\varepsilon)$$
$$+\mathcal{W}^{++}_{ac\boldsymbol{k}}\tilde{G}^R_{+\boldsymbol{k}}(\varepsilon)\tilde{G}^R_{+\boldsymbol{k}'}(\varepsilon)\tilde{G}^R_{-\boldsymbol{k}}(\varepsilon)\tilde{G}^A_{+\boldsymbol{k}}(\varepsilon)\tilde{G}^A_{+\boldsymbol{k}}(\varepsilon_1^-)\Big] + b\leftrightarrow c$$
$$\simeq -\frac{\pi e^3}{\hbar}\int[dk]\int[dk']\frac{(\tau^+_{\boldsymbol{k}})^2}{1-i\omega\tau^+_{\boldsymbol{k}}}f'(\varepsilon^+_{\boldsymbol{k}})I^{sj}_b(\boldsymbol{k},\boldsymbol{k}')\Big[\frac{\tilde{v}^{++}_{a\boldsymbol{k}}\tilde{v}^{++}_{c\boldsymbol{k}}}{2h_{\boldsymbol{k}}+\hbar\omega}\delta(\varepsilon^+_{\boldsymbol{k}}-\varepsilon^+_{\boldsymbol{k}'}+\hbar\omega)$$
$$-\frac{\mathcal{W}^{++}_{ac\boldsymbol{k}}}{2h_{\boldsymbol{k}}}\delta(\varepsilon^+_{\boldsymbol{k}}-\varepsilon^+_{\boldsymbol{k}'})\Big] + b\leftrightarrow c, \tag{93}$$

$$\mathcal{D}^{sj,2}_{(c)}(\omega) = \frac{ie^3\hbar}{2\pi}\int[dk]\int[dk']\int_{-\infty}^{\infty} d\varepsilon f'(\varepsilon) v^{+-}_{b\boldsymbol{k}'}\langle V^{++}_{\boldsymbol{k}\boldsymbol{k}'}V^{-+}_{\boldsymbol{k}'\boldsymbol{k}}\rangle\Big[\tilde{v}^{++}_{a\boldsymbol{k}}\tilde{v}^{++}_{c\boldsymbol{k}}\tilde{G}^R_{+\boldsymbol{k}}(\varepsilon_1^+)\tilde{G}^R_{+\boldsymbol{k}'}(\varepsilon_1^+)\tilde{G}^R_{-\boldsymbol{k}'}(\varepsilon)\tilde{G}^R_{+\boldsymbol{k}}(\varepsilon)\tilde{G}^A_{+\boldsymbol{k}}(\varepsilon)$$
$$+\mathcal{W}^{++}_{ac\boldsymbol{k}}\tilde{G}^R_{+\boldsymbol{k}}(\varepsilon)\tilde{G}^R_{+\boldsymbol{k}'}(\varepsilon)\tilde{G}^A_{-\boldsymbol{k}'}(\varepsilon)\tilde{G}^A_{+\boldsymbol{k}}(\varepsilon)\tilde{G}^A_{+\boldsymbol{k}}(\varepsilon_1^-)\Big] + b\leftrightarrow c$$
$$\simeq \frac{\pi e^3}{\hbar}\int[dk]\int[dk']\frac{(\tau^+_{\boldsymbol{k}})^2}{1-i\omega\tau^+_{\boldsymbol{k}}}f'(\varepsilon^+_{\boldsymbol{k}})I^{sj}_b(\boldsymbol{k}',\boldsymbol{k})\Big[\frac{\tilde{v}^{++}_{a\boldsymbol{k}}\tilde{v}^{++}_{c\boldsymbol{k}}}{\varepsilon^+_{\boldsymbol{k}}-\varepsilon^-_{\boldsymbol{k}'}}\delta(\varepsilon^+_{\boldsymbol{k}}-\varepsilon^+_{\boldsymbol{k}'}+\hbar\omega)$$
$$-\frac{\mathcal{W}^{++}_{ac\boldsymbol{k}}}{2h_{\boldsymbol{k}'}}\delta(\varepsilon^+_{\boldsymbol{k}}-\varepsilon^+_{\boldsymbol{k}'})\Big] + b\leftrightarrow c, \tag{94}$$

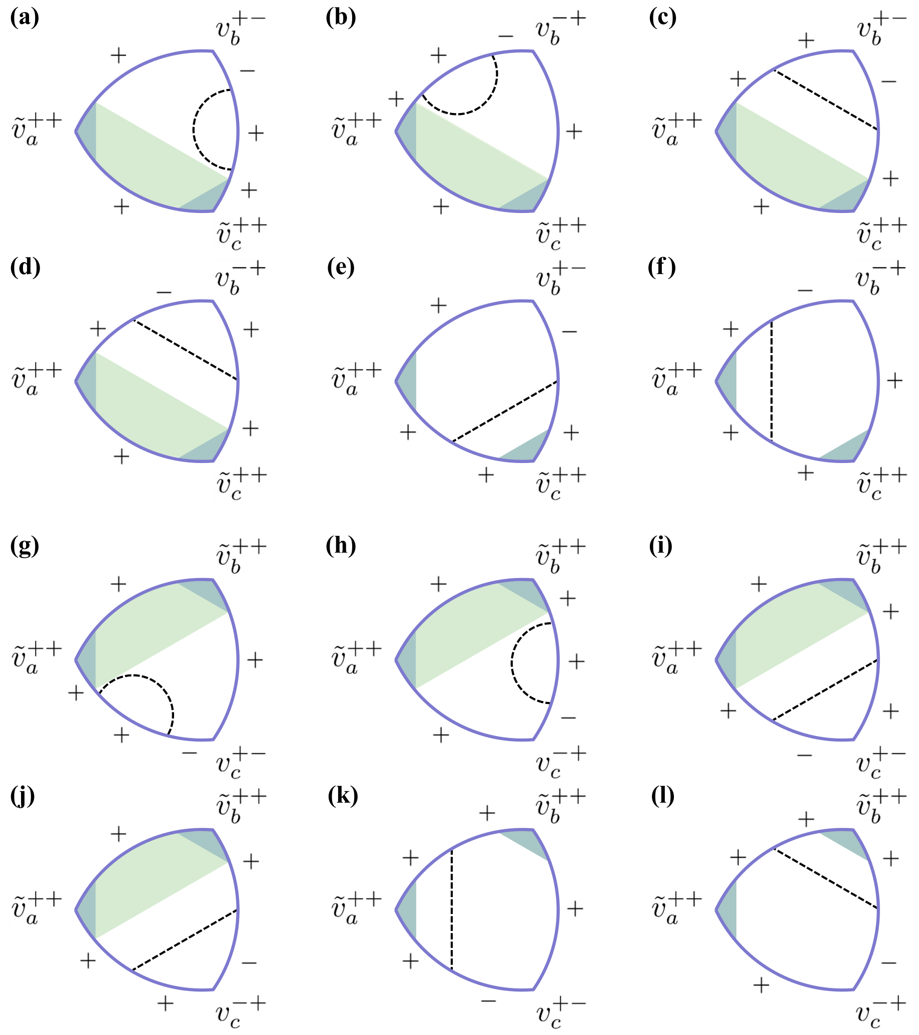

FIG. 7. Feynman diagrams of the anomalous distribution induced side-jump contribution to the nonlinear Hall effect.

$$
\begin{aligned}
\mathcal{D}^{sj,2}_{(d)}(\omega) = & \frac{ie^3\hbar}{2\pi}\int[dk]\int[dk']\int_{-\infty}^{\infty} d\varepsilon f'(\varepsilon) v^{-+}_{b\boldsymbol{k}'}\langle V^{+-}_{\boldsymbol{k}\boldsymbol{k}'}V^{++}_{\boldsymbol{k}'\boldsymbol{k}}\rangle\Big[\tilde{v}^{++}_{a\boldsymbol{k}}\tilde{v}^{++}_{c\boldsymbol{k}}\tilde{G}^R_{+\boldsymbol{k}}(\varepsilon^+_1)\tilde{G}^R_{-\boldsymbol{k}'}(\varepsilon^+_1)\tilde{G}^R_{+\boldsymbol{k}'}(\varepsilon)\tilde{G}^R_{+\boldsymbol{k}}(\varepsilon)\tilde{G}^A_{+\boldsymbol{k}}(\varepsilon) \\
& +\mathcal{W}^{++}_{ac\boldsymbol{k}}\tilde{G}^R_{+\boldsymbol{k}}(\varepsilon)\tilde{G}^R_{-\boldsymbol{k}'}(\varepsilon)\tilde{G}^A_{+\boldsymbol{k}'}(\varepsilon)\tilde{G}^A_{+\boldsymbol{k}}(\varepsilon)\tilde{G}^A_{+\boldsymbol{k}}(\varepsilon^-_1)\Big] + b\leftrightarrow c \\
\simeq & -\frac{\pi e^3}{\hbar}\int[dk]\int[dk']\frac{(\tau^+_{\boldsymbol{k}})^2}{1-i\omega\tau^+_{\boldsymbol{k}}}f'(\varepsilon^+_{\boldsymbol{k}})I^{sj}_b(\boldsymbol{k}',\boldsymbol{k})\Big[\frac{\tilde{v}^{++}_{a\boldsymbol{k}}\tilde{v}^{++}_{c\boldsymbol{k}}}{2h_{\boldsymbol{k}'}+\hbar\omega}+\frac{\mathcal{W}^{++}_{ac\boldsymbol{k}}}{2h_{\boldsymbol{k}'}}\Big]\delta(\varepsilon^+_{\boldsymbol{k}}-\varepsilon^+_{\boldsymbol{k}'}) + b\leftrightarrow c,
\end{aligned} \tag{95}
$$

$$
\begin{aligned}
\mathcal{D}^{sj,2}_{(e)}(\omega) = & \frac{ie^3\hbar}{2\pi}\int[dk]\int[dk']\int_{-\infty}^{\infty} d\varepsilon f'(\varepsilon)\tilde{v}^{++}_{a\boldsymbol{k}} v^{+-}_{b\boldsymbol{k}}\langle V^{-+}_{\boldsymbol{k}\boldsymbol{k}'}V^{++}_{\boldsymbol{k}'\boldsymbol{k}}\rangle\Big[\tilde{v}^{++}_{c\boldsymbol{k}'}\tilde{G}^R_{+\boldsymbol{k}}(\varepsilon^+_1)\tilde{G}^R_{-\boldsymbol{k}}(\varepsilon)\tilde{G}^R_{+\boldsymbol{k}'}(\varepsilon)\tilde{G}^A_{+\boldsymbol{k}'}(\varepsilon)\tilde{G}^A_{+\boldsymbol{k}}(\varepsilon) \\
& +v^{++}_{c\boldsymbol{k}'}\tilde{G}^R_{+\boldsymbol{k}}(\varepsilon)\tilde{G}^A_{-\boldsymbol{k}}(\varepsilon)\tilde{G}^A_{+\boldsymbol{k}'}(\varepsilon)\tilde{G}^A_{+\boldsymbol{k}'}(\varepsilon^-_1)\tilde{G}^A_{+\boldsymbol{k}}(\varepsilon^-_1)\Big] + b\leftrightarrow c \\
\simeq & -\frac{2\pi e^3}{\hbar}\int[dk]\int[dk']\frac{\tau^+_{\boldsymbol{k}}\tau^+_{\boldsymbol{k}'}}{1-i\omega\tau^+_{\boldsymbol{k}}}f'(\varepsilon^+_{\boldsymbol{k}})\tilde{v}^{++}_{a\boldsymbol{k}}I^{sj}_b(\boldsymbol{k},\boldsymbol{k}')\Big[\frac{\tilde{v}^{++}_{c\boldsymbol{k}'}}{2h_{\boldsymbol{k}}} \\
& -\frac{v^{++}_{c\boldsymbol{k}'}}{(1-i\omega\tau^+_{\boldsymbol{k}'})4h_{\boldsymbol{k}}}\Big]\delta(\varepsilon^+_{\boldsymbol{k}}-\varepsilon^+_{\boldsymbol{k}'}) + b\leftrightarrow c,
\end{aligned} \tag{96}
$$

$$\mathcal{D}^{sj,2}_{(f)}(\omega)=\frac{ie^3\hbar}{2\pi}\int[dk]\int[dk']\int_{-\infty}^{\infty}d\varepsilon f'(\varepsilon)\tilde{v}^{++}_{a\boldsymbol{k}'}v^{-+}_{b\boldsymbol{k}}\langle V^{+-}_{\boldsymbol{k}'\boldsymbol{k}}V^{++}_{\boldsymbol{k}\boldsymbol{k}'}\rangle\Big[\tilde{v}^{++}_{c\boldsymbol{k}}\tilde{G}^R_{+\boldsymbol{k}'}(\varepsilon_1^+)\tilde{G}^R_{-\boldsymbol{k}}(\varepsilon_1^+)\tilde{G}^R_{+\boldsymbol{k}}(\varepsilon)\tilde{G}^A_{+\boldsymbol{k}}(\varepsilon)\tilde{G}^A_{+\boldsymbol{k}'}(\varepsilon)$$
$$+v^{++}_{c\boldsymbol{k}}\tilde{G}^R_{+\boldsymbol{k}'}(\varepsilon)\tilde{G}^R_{-\boldsymbol{k}}(\varepsilon)\tilde{G}^A_{+\boldsymbol{k}}(\varepsilon)\tilde{G}^A_{+\boldsymbol{k}}(\varepsilon_1^-)\tilde{G}^A_{+\boldsymbol{k}'}(\varepsilon_1^-)\Big]+b\leftrightarrow c$$
$$\simeq\frac{2\pi e^3}{\hbar}\int[dk]\int[dk']\frac{\tau^+_{\boldsymbol{k}}\tau^+_{\boldsymbol{k}'}}{1-i\omega\tau^+_{\boldsymbol{k}'}}f'(\varepsilon^+_{\boldsymbol{k}})\tilde{v}^{++}_{a\boldsymbol{k}'}I^{sj}_b(\boldsymbol{k},\boldsymbol{k}')\Big[\frac{\tilde{v}^{++}_{c\boldsymbol{k}}}{2h_{\boldsymbol{k}}+\hbar\omega}$$
$$-\frac{v^{++}_{c\boldsymbol{k}}}{(1-i\omega\tau^+_{\boldsymbol{k}})4h_{\boldsymbol{k}}}\Big]\delta(\varepsilon^+_{\boldsymbol{k}}-\varepsilon^+_{\boldsymbol{k}'})+b\leftrightarrow c,\tag{97}$$

$$\mathcal{D}^{sj,2}_{(g)}(\omega)=\frac{ie^3\hbar}{2\pi}\int[dk]\int[dk']\int_{-\infty}^{\infty}d\varepsilon f'(\varepsilon)v^{+-}_{c\boldsymbol{k}}\langle V^{-+}_{\boldsymbol{k}\boldsymbol{k}'}V^{++}_{\boldsymbol{k}'\boldsymbol{k}}\rangle\Big[\mathcal{W}^{++}_{ab\boldsymbol{k}}\tilde{G}^R_{+\boldsymbol{k}}(\varepsilon_1^+)\tilde{G}^R_{+\boldsymbol{k}}(\varepsilon)\tilde{G}^A_{-\boldsymbol{k}}(\varepsilon)\tilde{G}^A_{+\boldsymbol{k}'}(\varepsilon)\tilde{G}^A_{+\boldsymbol{k}}(\varepsilon)$$
$$+\tilde{v}^{++}_{a\boldsymbol{k}}\tilde{v}^{++}_{b\boldsymbol{k}}\tilde{G}^R_{+\boldsymbol{k}}(\varepsilon)\tilde{G}^A_{+\boldsymbol{k}}(\varepsilon)\tilde{G}^A_{-\boldsymbol{k}}(\varepsilon_1^-)\tilde{G}^A_{+\boldsymbol{k}'}(\varepsilon_1^-)\tilde{G}^A_{+\boldsymbol{k}}(\varepsilon_1^-)\Big]+b\leftrightarrow c$$
$$\simeq-\frac{\pi e^3}{\hbar}\int[dk]\int[dk']\frac{(\tau^+_{\boldsymbol{k}})^2}{1-i\omega\tau^+_{\boldsymbol{k}}}f'(\varepsilon^+_{\boldsymbol{k}})I^{sj}_c(\boldsymbol{k},\boldsymbol{k}')\Big[\frac{\mathcal{W}^{++}_{ab\boldsymbol{k}}}{2h_{\boldsymbol{k}}}\delta(\varepsilon^+_{\boldsymbol{k}}-\varepsilon^+_{\boldsymbol{k}'})$$
$$-\frac{\tilde{v}^{++}_{a\boldsymbol{k}}\tilde{v}^{++}_{b\boldsymbol{k}}}{2h_{\boldsymbol{k}}-\hbar\omega}\delta(\varepsilon^+_{\boldsymbol{k}}-\varepsilon^+_{\boldsymbol{k}'}-\hbar\omega)\Big]+b\leftrightarrow c,\tag{98}$$

$$\mathcal{D}^{sj,2}_{(h)}(\omega)=\frac{ie^3\hbar}{2\pi}\int[dk]\int[dk']\int_{-\infty}^{\infty}d\varepsilon f'(\varepsilon)v^{-+}_{c\boldsymbol{k}}\langle V^{++}_{\boldsymbol{k}\boldsymbol{k}'}V^{+-}_{\boldsymbol{k}'\boldsymbol{k}}\rangle\Big[\mathcal{W}^{++}_{ab\boldsymbol{k}}\tilde{G}^R_{+\boldsymbol{k}}(\varepsilon_1^+)\tilde{G}^R_{+\boldsymbol{k}}(\varepsilon)\tilde{G}^R_{+\boldsymbol{k}'}(\varepsilon)\tilde{G}^R_{-\boldsymbol{k}}(\varepsilon)\tilde{G}^A_{+\boldsymbol{k}}(\varepsilon)$$
$$+\tilde{v}^{++}_{a\boldsymbol{k}}\tilde{v}^{++}_{b\boldsymbol{k}}\tilde{G}^R_{+\boldsymbol{k}}(\varepsilon)\tilde{G}^A_{+\boldsymbol{k}}(\varepsilon)\tilde{G}^A_{+\boldsymbol{k}'}(\varepsilon)\tilde{G}^A_{-\boldsymbol{k}}(\varepsilon)\tilde{G}^A_{+\boldsymbol{k}}(\varepsilon_1^-)\Big]+b\leftrightarrow c$$
$$\simeq-\frac{\pi e^3}{\hbar}\int[dk]\int[dk']\frac{(\tau^+_{\boldsymbol{k}})^2}{1-i\omega\tau^+_{\boldsymbol{k}}}f'(\varepsilon^+_{\boldsymbol{k}})I^{sj}_c(\boldsymbol{k},\boldsymbol{k}')\Big[\frac{\mathcal{W}^{++}_{ab\boldsymbol{k}}}{2h_{\boldsymbol{k}}}+\frac{\tilde{v}^{++}_{a\boldsymbol{k}}\tilde{v}^{++}_{b\boldsymbol{k}}}{2h_{\boldsymbol{k}}}\Big]\delta(\varepsilon^+_{\boldsymbol{k}}-\varepsilon^+_{\boldsymbol{k}'})+b\leftrightarrow c,\tag{99}$$

$$\mathcal{D}^{sj,2}_{(i)}(\omega)=\frac{ie^3\hbar}{2\pi}\int[dk]\int[dk']\int_{-\infty}^{\infty}d\varepsilon f'(\varepsilon)v^{+-}_{c\boldsymbol{k}'}\langle V^{++}_{\boldsymbol{k}\boldsymbol{k}'}V^{-+}_{\boldsymbol{k}'\boldsymbol{k}}\rangle\Big[\mathcal{W}^{++}_{ab\boldsymbol{k}}\tilde{G}^R_{+\boldsymbol{k}}(\varepsilon_1^+)\tilde{G}^R_{+\boldsymbol{k}}(\varepsilon)\tilde{G}^R_{+\boldsymbol{k}'}(\varepsilon)\tilde{G}^A_{-\boldsymbol{k}'}(\varepsilon)\tilde{G}^A_{+\boldsymbol{k}}(\varepsilon)$$
$$+\tilde{v}^{++}_{a\boldsymbol{k}}\tilde{v}^{++}_{b\boldsymbol{k}}\tilde{G}^R_{+\boldsymbol{k}}(\varepsilon)\tilde{G}^A_{+\boldsymbol{k}}(\varepsilon)\tilde{G}^A_{+\boldsymbol{k}'}(\varepsilon)\tilde{G}^A_{-\boldsymbol{k}'}(\varepsilon_1^-)\tilde{G}^A_{+\boldsymbol{k}}(\varepsilon_1^-)\Big]+b\leftrightarrow c$$
$$\simeq\frac{\pi e^3}{\hbar}\int[dk]\int[dk']\frac{(\tau^+_{\boldsymbol{k}})^2}{1-i\omega\tau^+_{\boldsymbol{k}}}f'(\varepsilon^+_{\boldsymbol{k}})I^{sj}_c(\boldsymbol{k}',\boldsymbol{k})\Big[\frac{\mathcal{W}^{++}_{ab\boldsymbol{k}}}{2h_{\boldsymbol{k}'}}+\frac{\tilde{v}^{++}_{a\boldsymbol{k}}\tilde{v}^{++}_{b\boldsymbol{k}}}{2h_{\boldsymbol{k}'}-\hbar\omega}\Big]\delta(\varepsilon^+_{\boldsymbol{k}}-\varepsilon^+_{\boldsymbol{k}'})+b\leftrightarrow c,\tag{100}$$

$$\mathcal{D}^{sj,2}_{(j)}(\omega)=\frac{ie^3\hbar}{2\pi}\int[dk]\int[dk']\int_{-\infty}^{\infty}d\varepsilon f'(\varepsilon)v^{-+}_{c\boldsymbol{k}'}\langle V^{+-}_{\boldsymbol{k}\boldsymbol{k}'}V^{++}_{\boldsymbol{k}'\boldsymbol{k}}\rangle\Big[\mathcal{W}^{++}_{ab\boldsymbol{k}}\tilde{G}^R_{+\boldsymbol{k}}(\varepsilon_1^+)\tilde{G}^R_{+\boldsymbol{k}}(\varepsilon)\tilde{G}^R_{-\boldsymbol{k}'}(\varepsilon)\tilde{G}^A_{+\boldsymbol{k}'}(\varepsilon)\tilde{G}^A_{+\boldsymbol{k}}(\varepsilon)$$
$$+\tilde{v}^{++}_{a\boldsymbol{k}}\tilde{v}^{++}_{b\boldsymbol{k}}\tilde{G}^R_{+\boldsymbol{k}}(\varepsilon)\tilde{G}^A_{+\boldsymbol{k}}(\varepsilon)\tilde{G}^A_{-\boldsymbol{k}'}(\varepsilon)\tilde{G}^A_{+\boldsymbol{k}'}(\varepsilon_1^-)\tilde{G}^A_{+\boldsymbol{k}}(\varepsilon_1^-)\Big]+b\leftrightarrow c$$
$$\simeq\frac{\pi e^3}{\hbar}\int[dk]\int[dk']\frac{(\tau^+_{\boldsymbol{k}})^2}{1-i\omega\tau^+_{\boldsymbol{k}}}f'(\varepsilon^+_{\boldsymbol{k}})I^{sj}_c(\boldsymbol{k}',\boldsymbol{k})\Big[\frac{\mathcal{W}^{++}_{ab\boldsymbol{k}}}{2h_{\boldsymbol{k}'}}\delta(\varepsilon^+_{\boldsymbol{k}}-\varepsilon^+_{\boldsymbol{k}'})$$
$$-\frac{\tilde{v}^{++}_{a\boldsymbol{k}}\tilde{v}^{++}_{b\boldsymbol{k}}}{\varepsilon^+_{\boldsymbol{k}}-\varepsilon^-_{\boldsymbol{k}'}}\delta(\varepsilon^+_{\boldsymbol{k}}-\varepsilon^+_{\boldsymbol{k}'}-\hbar\omega)\Big]+b\leftrightarrow c,\tag{101}$$

$$\mathcal{D}^{sj,2}_{(k)}(\omega)=\frac{ie^3\hbar}{2\pi}\int[dk]\int[dk']\int_{-\infty}^{\infty}d\varepsilon f'(\varepsilon)\tilde{v}^{++}_{a\boldsymbol{k}'}v^{+-}_{c\boldsymbol{k}}\langle V^{++}_{\boldsymbol{k}'\boldsymbol{k}}V^{-+}_{\boldsymbol{k}\boldsymbol{k}'}\rangle\Big[v^{++}_{b\boldsymbol{k}}\tilde{G}^R_{+\boldsymbol{k}'}(\varepsilon_1^+)\tilde{G}^R_{+\boldsymbol{k}}(\varepsilon_1^+)\tilde{G}^R_{+\boldsymbol{k}}(\varepsilon)\tilde{G}^A_{-\boldsymbol{k}}(\varepsilon)\tilde{G}^A_{+\boldsymbol{k}'}(\varepsilon)$$
$$+\tilde{v}^{++}_{b\boldsymbol{k}}\tilde{G}^R_{+\boldsymbol{k}'}(\varepsilon)\tilde{G}^R_{+\boldsymbol{k}}(\varepsilon)\tilde{G}^A_{+\boldsymbol{k}}(\varepsilon)\tilde{G}^A_{-\boldsymbol{k}}(\varepsilon_1^-)\tilde{G}^A_{+\boldsymbol{k}'}(\varepsilon_1^-)\Big]+b\leftrightarrow c$$
$$\simeq\frac{2\pi e^3}{\hbar}\int[dk]\int[dk']\frac{\tau^+_{\boldsymbol{k}}\tau^+_{\boldsymbol{k}'}f'(\varepsilon^+_{\boldsymbol{k}})}{1-i\omega\tau^+_{\boldsymbol{k}'}}\tilde{v}^{++}_{a\boldsymbol{k}'}I^{sj}_c(\boldsymbol{k},\boldsymbol{k}')\Big[\frac{v^{++}_{b\boldsymbol{k}}}{(1-i\omega\tau^+_{\boldsymbol{k}})4h_{\boldsymbol{k}}}$$
$$-\frac{\tilde{v}^{++}_{b\boldsymbol{k}}}{2h_{\boldsymbol{k}}-\hbar\omega}\Big]\delta(\varepsilon^+_{\boldsymbol{k}}-\varepsilon^+_{\boldsymbol{k}'})+b\leftrightarrow c,\tag{102}$$

$$\mathcal{D}^{sj,2}_{(l)}(\omega)=\frac{ie^3\hbar}{2\pi}\int[dk]\int[dk']\int_{-\infty}^{\infty}d\varepsilon f'(\varepsilon)\tilde{v}^{++}_{a\boldsymbol{k}}v^{-+}_{c\boldsymbol{k}}\langle V^{++}_{\boldsymbol{k}\boldsymbol{k}'}V^{+-}_{\boldsymbol{k}'\boldsymbol{k}}\rangle\Big[v^{++}_{b\boldsymbol{k}'}\tilde{G}^R_{+\boldsymbol{k}}(\varepsilon_1^+)\tilde{G}^R_{+\boldsymbol{k}'}(\varepsilon_1^+)\tilde{G}^R_{+\boldsymbol{k}'}(\varepsilon)\tilde{G}^R_{-\boldsymbol{k}}(\varepsilon)\tilde{G}^A_{+\boldsymbol{k}}(\varepsilon)$$
$$+\tilde{v}^{++}_{b\boldsymbol{k}'}\tilde{G}^R_{+\boldsymbol{k}}(\varepsilon)\tilde{G}^R_{+\boldsymbol{k}'}(\varepsilon)\tilde{G}^A_{+\boldsymbol{k}'}(\varepsilon)\tilde{G}^A_{-\boldsymbol{k}}(\varepsilon)\tilde{G}^A_{+\boldsymbol{k}}(\varepsilon_1^-)\Big]+b\leftrightarrow c$$
$$\simeq-\frac{2\pi e^3}{\hbar}\int[dk]\int[dk']\frac{\tau^+_{\boldsymbol{k}}\tau^+_{\boldsymbol{k}'}f'(\varepsilon^+_{\boldsymbol{k}})}{1-i\omega\tau^+_{\boldsymbol{k}}}\tilde{v}^{++}_{a\boldsymbol{k}}I^{sj}_c(\boldsymbol{k},\boldsymbol{k}')\Big[\frac{v^{++}_{b\boldsymbol{k}'}}{(1-i\omega\tau^+_{\boldsymbol{k}'})4h_{\boldsymbol{k}}}-\frac{\tilde{v}^{++}_{b\boldsymbol{k}'}}{2h_{\boldsymbol{k}}}\Big]\delta(\varepsilon^+_{\boldsymbol{k}}-\varepsilon^+_{\boldsymbol{k}'})+b\leftrightarrow c. \tag{103}$$

Combining all the relevant contributions in the above results, the anomalous distribution induced side-jump contribution can be found as

$$\Xi^{I\text{-}sj,2}_{abc}=\sum_{i=a}^{l}\frac{\partial\tilde{\mathcal{D}}^{sj,2}_{(i)}(\omega)}{\partial\omega}\Big|_{\omega=0}$$
$$=-e^3\int[dk]\int[dk']\tau^+_{\boldsymbol{k}}f'(\varepsilon^+_{\boldsymbol{k}})\Big\{\tau^+_{\boldsymbol{k}}\tilde{v}^{++}_{a\boldsymbol{k}}\tilde{v}^{++}_{b\boldsymbol{k}}\Big[V^{sj,0}_{c\boldsymbol{k}\boldsymbol{k}'}\delta'(\varepsilon^+_{\boldsymbol{k}}-\varepsilon^+_{\boldsymbol{k}'})-V^{sj,2}_{c\boldsymbol{k}\boldsymbol{k}'}\delta(\varepsilon^+_{\boldsymbol{k}}-\varepsilon^+_{\boldsymbol{k}'})\Big]$$
$$+\tau^+_{\boldsymbol{k}'}\tilde{v}^{++}_{a\boldsymbol{k}'}\tilde{v}^{++}_{b\boldsymbol{k}}(V^{sj,1}_{c\boldsymbol{k}\boldsymbol{k}'}+V^{sj,2}_{c\boldsymbol{k}\boldsymbol{k}'})\delta(\varepsilon^+_{\boldsymbol{k}}-\varepsilon^+_{\boldsymbol{k}'})\Big\}+b\leftrightarrow c, \tag{104}$$

And the anomalous distribution induced side-jump contribution to χ^I_{abc} in the dc limit is

$$\chi^{I\text{-}sj,2}_{abc}=-\frac{e^3}{4}\int[dk]\int[dk']\tau^+_{\boldsymbol{k}}f'(\varepsilon^+_{\boldsymbol{k}})\Big\{\tau^+_{\boldsymbol{k}}\tilde{v}^{++}_{a\boldsymbol{k}}\tilde{v}^{++}_{b\boldsymbol{k}}\Big[V^{sj,0}_{c\boldsymbol{k}\boldsymbol{k}'}\delta'(\varepsilon^+_{\boldsymbol{k}}-\varepsilon^+_{\boldsymbol{k}'})-V^{sj,2}_{c\boldsymbol{k}\boldsymbol{k}'}\delta(\varepsilon^+_{\boldsymbol{k}}-\varepsilon^+_{\boldsymbol{k}'})\Big]$$
$$+\tau^+_{\boldsymbol{k}'}\tilde{v}^{++}_{a\boldsymbol{k}'}\tilde{v}^{++}_{b\boldsymbol{k}}(V^{sj,1}_{c\boldsymbol{k}\boldsymbol{k}'}+V^{sj,2}_{c\boldsymbol{k}\boldsymbol{k}'})\delta(\varepsilon^+_{\boldsymbol{k}}-\varepsilon^+_{\boldsymbol{k}'})\Big\}+b\leftrightarrow c. \tag{105}$$

C. Skew-scattering contribution

According to previous works on the anomalous [9, 11] and nonlinear Hall effects [4], the skew-scattering diagrams can also be classified into two categories. The first one is contributed from the antisymmetric parts of the Gaussian disorder distribution, we denote them as the intrinsic skew-scattering diagrams with superscript $\mathcal{D}^{sk,1}$. The second one is contributed from the antisymmetric parts of the non-Gaussian disorder distribution, we denote them as the extrinsic skew-scattering diagrams with superscript $\mathcal{D}^{sk,2}$.

1. *Intrinsic skew-scattering contribution*

The intrinsic skew-scattering diagrams are summarised in Supplementary Fig. 8. By denoting the diagrams in Supplementary Fig. 8 (a)-(u) as $\tilde{\mathcal{D}}^{sk,1}_{(a)}(\omega)$-$\tilde{\mathcal{D}}^{sk,1}_{(u)}(\omega)$, we have

$$\mathcal{D}^{sk,1}_{(a)}(\omega)=\frac{ie^3\hbar}{2\pi}\int[dk]\int[dk']\int[dk'']\int_{-\infty}^{\infty}d\varepsilon f'(\varepsilon)\tilde{v}^{++}_{a\boldsymbol{k}}\langle V^{+-}_{\boldsymbol{k}\boldsymbol{k}'}V^{++}_{\boldsymbol{k}'\boldsymbol{k}}\rangle\langle V^{-+}_{\boldsymbol{k}'\boldsymbol{k}''}V^{++}_{\boldsymbol{k}''\boldsymbol{k}'}\rangle$$
$$\times\Big[\mathcal{W}^{++}_{cb\boldsymbol{k}'}\tilde{G}^R_{+\boldsymbol{k}}(\varepsilon_1^+)\tilde{G}^R_{-\boldsymbol{k}'}(\varepsilon_1^+)\tilde{G}^R_{+\boldsymbol{k}''}(\varepsilon_1^+)\tilde{G}^R_{+\boldsymbol{k}'}(\varepsilon_1^+)\tilde{G}^R_{+\boldsymbol{k}'}(\varepsilon)\tilde{G}^A_{+\boldsymbol{k}'}(\varepsilon)\tilde{G}^A_{+\boldsymbol{k}}(\varepsilon)$$
$$+\mathcal{W}^{++}_{bc\boldsymbol{k}'}\tilde{G}^R_{+\boldsymbol{k}}(\varepsilon)\tilde{G}^R_{-\boldsymbol{k}'}(\varepsilon)\tilde{G}^R_{+\boldsymbol{k}''}(\varepsilon)\tilde{G}^R_{+\boldsymbol{k}'}(\varepsilon)\tilde{G}^A_{+\boldsymbol{k}'}(\varepsilon)\tilde{G}^A_{+\boldsymbol{k}'}(\varepsilon_1^-)\tilde{G}^A_{+\boldsymbol{k}}(\varepsilon_1^-)\Big]+b\leftrightarrow c$$
$$\simeq\frac{2\pi^2e^3}{\hbar^2}\int[dk]\int[dk']\int[dk'']\frac{\tau^+_{\boldsymbol{k}}(\tau^+_{\boldsymbol{k}'})^2f'(\varepsilon^+_{\boldsymbol{k}})}{(1-i\omega\tau^+_{\boldsymbol{k}})(1-i\omega\tau^+_{\boldsymbol{k}'})}I^{sk,1}(\boldsymbol{k}',\boldsymbol{k}'',\boldsymbol{k})\tilde{v}^{++}_{a\boldsymbol{k}}$$
$$\times\Big[\frac{\mathcal{W}^{++}_{cb\boldsymbol{k}'}}{2h_{\boldsymbol{k}'}+\hbar\omega}\delta(\varepsilon^+_{\boldsymbol{k}}-\varepsilon^+_{\boldsymbol{k}''}+\hbar\omega)-\frac{\mathcal{W}^{++}_{bc\boldsymbol{k}'}}{2h_{\boldsymbol{k}'}}\delta(\varepsilon^+_{\boldsymbol{k}}-\varepsilon^+_{\boldsymbol{k}''})\Big]\delta(\varepsilon^+_{\boldsymbol{k}}-\varepsilon^+_{\boldsymbol{k}'})+b\leftrightarrow c, \tag{106}$$

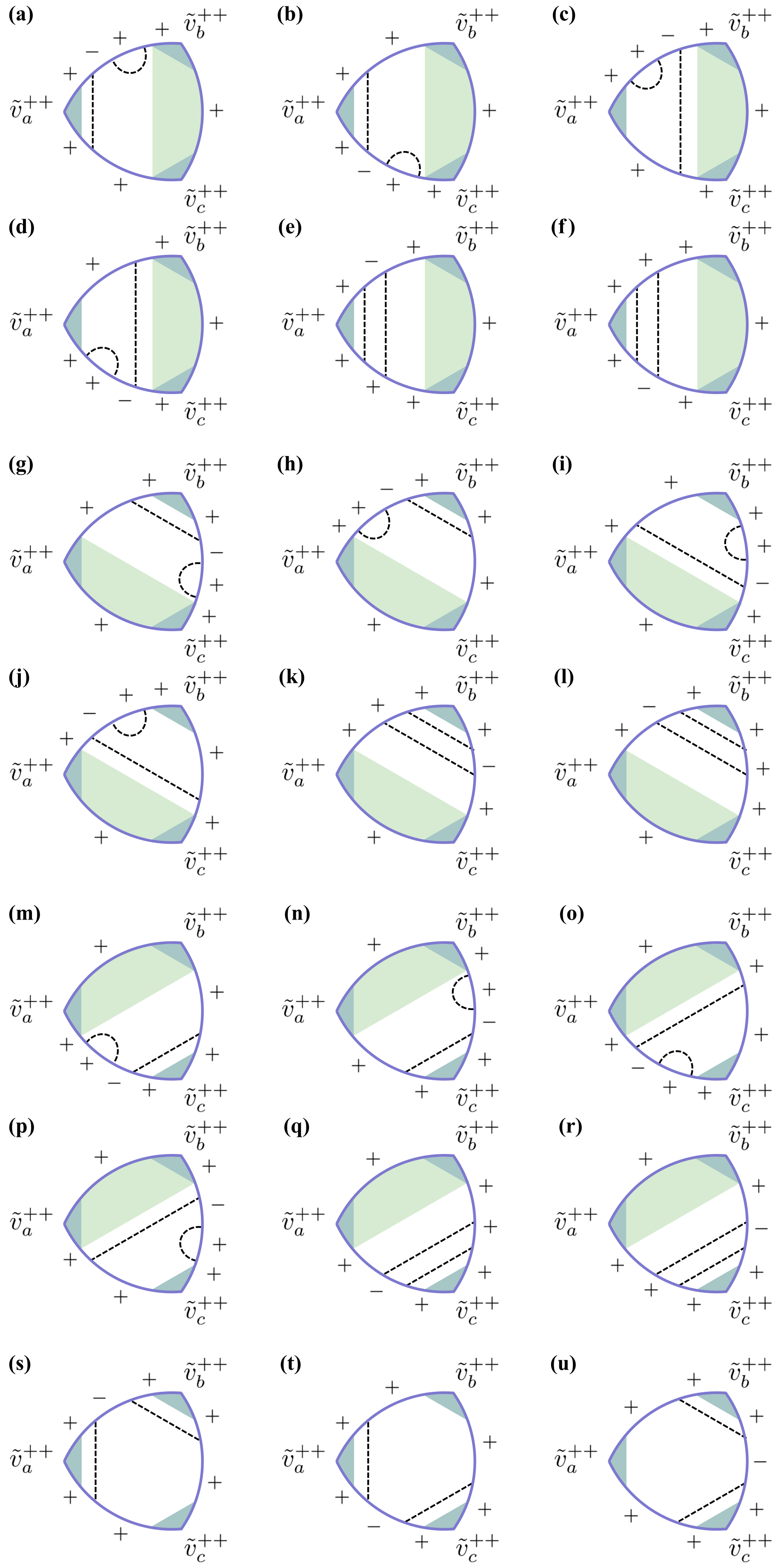

FIG. 8. Feynman diagrams of the intrinsic skew-scattering contribution to the nonlinear Hall effect.

$$\mathcal{D}^{sk,1}_{(b)}(\omega) = \frac{ie^3\hbar}{2\pi}\int[dk]\int[dk']\int[dk'']\int_{-\infty}^{\infty} d\varepsilon f'(\varepsilon)\tilde{v}^{++}_{a\boldsymbol{k}}\langle V^{++}_{\boldsymbol{k}\boldsymbol{k}'}V^{-+}_{\boldsymbol{k}'\boldsymbol{k}}\rangle\langle V^{++}_{\boldsymbol{k}'\boldsymbol{k}''}V^{+-}_{\boldsymbol{k}''\boldsymbol{k}'}\rangle$$
$$\times\Big[\mathcal{W}^{++}_{cb\boldsymbol{k}'}\tilde{G}^R_{+\boldsymbol{k}}(\varepsilon_1^+)\tilde{G}^R_{+\boldsymbol{k}'}(\varepsilon_1^+)\tilde{G}^R_{+\boldsymbol{k}'}(\varepsilon)\tilde{G}^A_{+\boldsymbol{k}'}(\varepsilon)\tilde{G}^A_{+\boldsymbol{k}''}(\varepsilon)\tilde{G}^A_{-\boldsymbol{k}'}(\varepsilon)\tilde{G}^A_{+\boldsymbol{k}}(\varepsilon)$$
$$+\mathcal{W}^{++}_{bc\boldsymbol{k}'}\tilde{G}^R_{+\boldsymbol{k}}(\varepsilon)\tilde{G}^R_{+\boldsymbol{k}'}(\varepsilon)\tilde{G}^A_{+\boldsymbol{k}'}(\varepsilon)\tilde{G}^A_{+\boldsymbol{k}'}(\varepsilon_1^-)\tilde{G}^A_{+\boldsymbol{k}''}(\varepsilon_1^-)\tilde{G}^A_{-\boldsymbol{k}'}(\varepsilon_1^-)\tilde{G}^A_{+\boldsymbol{k}}(\varepsilon_1^-)\Big] + b\leftrightarrow c$$
$$\simeq \frac{2\pi^2e^3}{\hbar^2}\int[dk]\int[dk']\int[dk'']\frac{\tau^+_{\boldsymbol{k}}(\tau^+_{\boldsymbol{k}'})^2 f'(\varepsilon^+_{\boldsymbol{k}})}{(1-i\omega\tau^+_{\boldsymbol{k}})(1-i\omega\tau^+_{\boldsymbol{k}'})} I^{sk,1}(\boldsymbol{k}',\boldsymbol{k}'',\boldsymbol{k})\tilde{v}^{++}_{a\boldsymbol{k}}$$
$$\times\Big[\frac{\mathcal{W}^{++}_{cb\boldsymbol{k}'}}{2h_{\boldsymbol{k}'}}\delta(\varepsilon^+_{\boldsymbol{k}}-\varepsilon^+_{\boldsymbol{k}''}) - \frac{\mathcal{W}^{++}_{bc\boldsymbol{k}'}}{2h_{\boldsymbol{k}'}-\hbar\omega}\delta(\varepsilon^+_{\boldsymbol{k}}-\varepsilon^+_{\boldsymbol{k}''}-\hbar\omega)\Big]\delta(\varepsilon^+_{\boldsymbol{k}}-\varepsilon^+_{\boldsymbol{k}'}) + b\leftrightarrow c, \tag{107}$$

$$\mathcal{D}^{sk,1}_{(c)}(\omega) = \frac{ie^3\hbar}{2\pi}\int[dk]\int[dk']\int[dk'']\int_{-\infty}^{\infty} d\varepsilon f'(\varepsilon)\tilde{v}^{++}_{a\boldsymbol{k}}\langle V^{++}_{\boldsymbol{k}\boldsymbol{k}''}V^{+-}_{\boldsymbol{k}''\boldsymbol{k}}\rangle\langle V^{-+}_{\boldsymbol{k}\boldsymbol{k}'}V^{++}_{\boldsymbol{k}'\boldsymbol{k}}\rangle$$
$$\times\Big[\mathcal{W}^{++}_{cb\boldsymbol{k}'}\tilde{G}^R_{+\boldsymbol{k}}(\varepsilon_1^+)\tilde{G}^R_{+\boldsymbol{k}''}(\varepsilon_1^+)\tilde{G}^R_{-\boldsymbol{k}}(\varepsilon_1^+)\tilde{G}^R_{+\boldsymbol{k}'}(\varepsilon_1^+)\tilde{G}^R_{+\boldsymbol{k}'}(\varepsilon)\tilde{G}^A_{+\boldsymbol{k}'}(\varepsilon)\tilde{G}^A_{+\boldsymbol{k}}(\varepsilon)$$
$$+\mathcal{W}^{++}_{bc\boldsymbol{k}'}\tilde{G}^R_{+\boldsymbol{k}}(\varepsilon)\tilde{G}^R_{+\boldsymbol{k}''}(\varepsilon)\tilde{G}^R_{-\boldsymbol{k}}(\varepsilon)\tilde{G}^R_{+\boldsymbol{k}'}(\varepsilon)\tilde{G}^A_{+\boldsymbol{k}'}(\varepsilon)\tilde{G}^A_{+\boldsymbol{k}'}(\varepsilon_1^-)\tilde{G}^A_{+\boldsymbol{k}}(\varepsilon_1^-)\Big] + b\leftrightarrow c$$
$$\simeq \frac{2\pi^2e^3}{\hbar^2}\int[dk]\int[dk']\int[dk'']\frac{\tau^+_{\boldsymbol{k}}(\tau^+_{\boldsymbol{k}'})^2 f'(\varepsilon^+_{\boldsymbol{k}})}{(1-i\omega\tau^+_{\boldsymbol{k}})(1-i\omega\tau^+_{\boldsymbol{k}'})} I^{sk,1}(\boldsymbol{k},\boldsymbol{k}',\boldsymbol{k}'')\tilde{v}^{++}_{a\boldsymbol{k}}$$
$$\times\Big[\frac{\mathcal{W}^{++}_{cb\boldsymbol{k}'}}{2h_{\boldsymbol{k}}+\hbar\omega}\delta(\varepsilon^+_{\boldsymbol{k}}-\varepsilon^+_{\boldsymbol{k}''}+\hbar\omega) - \frac{\mathcal{W}^{++}_{bc\boldsymbol{k}'}}{2h_{\boldsymbol{k}}}\delta(\varepsilon^+_{\boldsymbol{k}}-\varepsilon^+_{\boldsymbol{k}''})\Big]\delta(\varepsilon^+_{\boldsymbol{k}}-\varepsilon^+_{\boldsymbol{k}'}) + b\leftrightarrow c, \tag{108}$$

$$\mathcal{D}^{sk,1}_{(d)}(\omega) = \frac{ie^3\hbar}{2\pi}\int[dk]\int[dk']\int[dk'']\int_{-\infty}^{\infty} d\varepsilon f'(\varepsilon)\tilde{v}^{++}_{a\boldsymbol{k}}\langle V^{++}_{\boldsymbol{k}\boldsymbol{k}'}V^{+-}_{\boldsymbol{k}'\boldsymbol{k}}\rangle\langle V^{-+}_{\boldsymbol{k}\boldsymbol{k}''}V^{++}_{\boldsymbol{k}''\boldsymbol{k}}\rangle$$
$$\times\Big[\mathcal{W}^{++}_{cb\boldsymbol{k}'}\tilde{G}^R_{+\boldsymbol{k}}(\varepsilon_1^+)\tilde{G}^R_{+\boldsymbol{k}'}(\varepsilon_1^+)\tilde{G}^R_{+\boldsymbol{k}'}(\varepsilon)\tilde{G}^A_{+\boldsymbol{k}'}(\varepsilon)\tilde{G}^A_{-\boldsymbol{k}}(\varepsilon)\tilde{G}^A_{+\boldsymbol{k}''}(\varepsilon)\tilde{G}^A_{+\boldsymbol{k}}(\varepsilon)$$
$$+\mathcal{W}^{++}_{bc\boldsymbol{k}'}\tilde{G}^R_{+\boldsymbol{k}}(\varepsilon)\tilde{G}^R_{+\boldsymbol{k}'}(\varepsilon)\tilde{G}^A_{+\boldsymbol{k}'}(\varepsilon)\tilde{G}^A_{+\boldsymbol{k}'}(\varepsilon_1^-)\tilde{G}^A_{-\boldsymbol{k}}(\varepsilon_1^-)\tilde{G}^A_{+\boldsymbol{k}''}(\varepsilon_1^-)\tilde{G}^A_{+\boldsymbol{k}}(\varepsilon_1^-)\Big] + b\leftrightarrow c$$
$$\simeq \frac{2\pi^2e^3}{\hbar^2}\int[dk]\int[dk']\int[dk'']\frac{\tau^+_{\boldsymbol{k}}(\tau^+_{\boldsymbol{k}'})^2 f'(\varepsilon^+_{\boldsymbol{k}})}{(1-i\omega\tau^+_{\boldsymbol{k}})(1-i\omega\tau^+_{\boldsymbol{k}'})} I^{sk,1}(\boldsymbol{k},\boldsymbol{k}',\boldsymbol{k}'')\tilde{v}^{++}_{a\boldsymbol{k}}$$
$$\times\Big[\frac{\mathcal{W}^{++}_{cb\boldsymbol{k}'}}{2h_{\boldsymbol{k}}}\delta(\varepsilon^+_{\boldsymbol{k}}-\varepsilon^+_{\boldsymbol{k}''}) - \frac{\mathcal{W}^{++}_{bc\boldsymbol{k}'}}{2h_{\boldsymbol{k}}-\hbar\omega}\delta(\varepsilon^+_{\boldsymbol{k}}-\varepsilon^+_{\boldsymbol{k}''}-\hbar\omega)\Big]\delta(\varepsilon^+_{\boldsymbol{k}}-\varepsilon^+_{\boldsymbol{k}'}) + b\leftrightarrow c, \tag{109}$$

$$\mathcal{D}^{sk,1}_{(e)}(\omega) = \frac{ie^3\hbar}{2\pi}\int[dk]\int[dk']\int[dk'']\int_{-\infty}^{\infty} d\varepsilon f'(\varepsilon)\tilde{v}^{++}_{a\boldsymbol{k}}\langle V^{+-}_{\boldsymbol{k}\boldsymbol{k}''}V^{++}_{\boldsymbol{k}''\boldsymbol{k}}\rangle\langle V^{-+}_{\boldsymbol{k}''\boldsymbol{k}'}V^{++}_{\boldsymbol{k}'\boldsymbol{k}''}\rangle$$
$$\times\Big[\mathcal{W}^{++}_{cb\boldsymbol{k}'}\tilde{G}^R_{+\boldsymbol{k}}(\varepsilon_1^+)\tilde{G}^R_{-\boldsymbol{k}''}(\varepsilon_1^+)\tilde{G}^R_{+\boldsymbol{k}'}(\varepsilon_1^+)\tilde{G}^R_{+\boldsymbol{k}'}(\varepsilon)\tilde{G}^A_{+\boldsymbol{k}'}(\varepsilon)\tilde{G}^A_{+\boldsymbol{k}''}(\varepsilon)\tilde{G}^A_{+\boldsymbol{k}}(\varepsilon)$$
$$+\mathcal{W}^{++}_{bc\boldsymbol{k}'}\tilde{G}^R_{+\boldsymbol{k}}(\varepsilon)\tilde{G}^R_{-\boldsymbol{k}''}(\varepsilon)\tilde{G}^R_{+\boldsymbol{k}'}(\varepsilon)\tilde{G}^A_{+\boldsymbol{k}'}(\varepsilon)\tilde{G}^A_{+\boldsymbol{k}'}(\varepsilon_1^-)\tilde{G}^A_{+\boldsymbol{k}''}(\varepsilon_1^-)\tilde{G}^A_{+\boldsymbol{k}}(\varepsilon_1^-)\Big] + b\leftrightarrow c$$
$$\simeq \frac{2\pi^2e^3}{\hbar^2}\int[dk]\int[dk']\int[dk'']\frac{\tau^+_{\boldsymbol{k}}(\tau^+_{\boldsymbol{k}'})^2 f'(\varepsilon^+_{\boldsymbol{k}})}{(1-i\omega\tau^+_{\boldsymbol{k}})(1-i\omega\tau^+_{\boldsymbol{k}'})} I^{sk,1}(\boldsymbol{k}'',\boldsymbol{k},\boldsymbol{k}')\tilde{v}^{++}_{a\boldsymbol{k}}$$
$$\times\Big[\frac{\mathcal{W}^{++}_{cb\boldsymbol{k}'}}{2h_{\boldsymbol{k}''}+\hbar\omega}\delta(\varepsilon^+_{\boldsymbol{k}}-\varepsilon^+_{\boldsymbol{k}''}) - \frac{\mathcal{W}^{++}_{bc\boldsymbol{k}'}}{\varepsilon^+_{\boldsymbol{k}}-\varepsilon^-_{\boldsymbol{k}''}}\delta(\varepsilon^+_{\boldsymbol{k}}-\varepsilon^+_{\boldsymbol{k}''}-\hbar\omega)\Big]\delta(\varepsilon^+_{\boldsymbol{k}}-\varepsilon^+_{\boldsymbol{k}'}) + b\leftrightarrow c, \tag{110}$$

$$\mathcal{D}^{sk,1}_{(f)}(\omega) = \frac{ie^3\hbar}{2\pi}\int[dk]\int[dk']\int[dk'']\int_{-\infty}^{\infty}d\varepsilon f'(\varepsilon)\tilde{v}^{++}_{a\boldsymbol{k}}\langle V^{++}_{\boldsymbol{k}\boldsymbol{k}''}V^{-+}_{\boldsymbol{k}''\boldsymbol{k}}\rangle\langle V^{++}_{\boldsymbol{k}''\boldsymbol{k}'}V^{+-}_{\boldsymbol{k}'\boldsymbol{k}''}\rangle$$
$$\times\Big[\mathcal{W}^{++}_{cb\boldsymbol{k}'}\tilde{G}^R_{+\boldsymbol{k}}(\varepsilon_1^+)\tilde{G}^R_{+\boldsymbol{k}''}(\varepsilon_1^+)\tilde{G}^R_{+\boldsymbol{k}'}(\varepsilon_1^+)\tilde{G}^R_{+\boldsymbol{k}'}(\varepsilon)\tilde{G}^A_{+\boldsymbol{k}'}(\varepsilon)\tilde{G}^A_{-\boldsymbol{k}''}(\varepsilon)\tilde{G}^A_{+\boldsymbol{k}}(\varepsilon)$$
$$+\mathcal{W}^{++}_{bc\boldsymbol{k}'}\tilde{G}^R_{+\boldsymbol{k}}(\varepsilon)\tilde{G}^R_{+\boldsymbol{k}''}(\varepsilon)\tilde{G}^R_{+\boldsymbol{k}'}(\varepsilon)\tilde{G}^A_{+\boldsymbol{k}'}(\varepsilon)\tilde{G}^A_{+\boldsymbol{k}'}(\varepsilon_1^-)\tilde{G}^A_{-\boldsymbol{k}''}(\varepsilon_1^-)\tilde{G}^A_{+\boldsymbol{k}}(\varepsilon_1^-)\Big] + b\leftrightarrow c$$
$$\simeq \frac{2\pi^2e^3}{\hbar^2}\int[dk]\int[dk']\int[dk'']\frac{\tau^+_{\boldsymbol{k}}(\tau^+_{\boldsymbol{k}'})^2f'(\varepsilon^+_{\boldsymbol{k}})}{(1-i\omega\tau^+_{\boldsymbol{k}})(1-i\omega\tau^+_{\boldsymbol{k}'})}I^{sk,1}(\boldsymbol{k}'',\boldsymbol{k},\boldsymbol{k}')\tilde{v}^{++}_{a\boldsymbol{k}}$$
$$\times\Big[\frac{\mathcal{W}^{++}_{cb\boldsymbol{k}'}}{\varepsilon^+_{\boldsymbol{k}}-\varepsilon^-_{\boldsymbol{k}''}}\delta(\varepsilon^+_{\boldsymbol{k}}-\varepsilon^+_{\boldsymbol{k}''}+\hbar\omega)-\frac{\mathcal{W}^{++}_{bc\boldsymbol{k}'}}{2h_{\boldsymbol{k}''}-\hbar\omega}\delta(\varepsilon^+_{\boldsymbol{k}}-\varepsilon^+_{\boldsymbol{k}''})\Big]\delta(\varepsilon^+_{\boldsymbol{k}}-\varepsilon^+_{\boldsymbol{k}'})+b\leftrightarrow c, \tag{111}$$

$$\mathcal{D}^{sk,1}_{(g)}(\omega) = \frac{ie^3\hbar}{2\pi}\int[dk]\int[dk']\int_{-\infty}^{\infty}d\varepsilon f'(\varepsilon)\langle V^{++}_{\boldsymbol{k}\boldsymbol{k}'}V^{+-}_{\boldsymbol{k}'\boldsymbol{k}}\rangle\langle V^{-+}_{\boldsymbol{k}\boldsymbol{k}''}V^{++}_{\boldsymbol{k}''\boldsymbol{k}}\rangle$$
$$\times\Big[\tilde{v}^{++}_{a\boldsymbol{k}}v^{++}_{b\boldsymbol{k}'}\tilde{v}^{++}_{c\boldsymbol{k}}\tilde{G}^R_{+\boldsymbol{k}}(\varepsilon_1^+)\tilde{G}^R_{+\boldsymbol{k}'}(\varepsilon_1^+)\tilde{G}^R_{+\boldsymbol{k}'}(\varepsilon)\tilde{G}^R_{-\boldsymbol{k}}(\varepsilon)\tilde{G}^R_{+\boldsymbol{k}''}(\varepsilon)\tilde{G}^R_{+\boldsymbol{k}}(\varepsilon)\tilde{G}^A_{+\boldsymbol{k}}(\varepsilon)$$
$$+\tilde{v}^{++}_{b\boldsymbol{k}'}\mathcal{W}_{ac\boldsymbol{k}}\tilde{G}^R_{+\boldsymbol{k}}(\varepsilon)\tilde{G}^R_{+\boldsymbol{k}'}(\varepsilon)\tilde{G}^A_{+\boldsymbol{k}'}(\varepsilon)\tilde{G}^A_{-\boldsymbol{k}}(\varepsilon)\tilde{G}^A_{+\boldsymbol{k}''}(\varepsilon)\tilde{G}^A_{+\boldsymbol{k}}(\varepsilon)\tilde{G}^A_{+\boldsymbol{k}}(\varepsilon_1^-)\Big] + b\leftrightarrow c$$
$$\simeq \frac{\pi^2e^3}{\hbar^2}\int[dk]\int[dk']\int[dk'']\frac{\tau^+_{\boldsymbol{k}'}(\tau^+_{\boldsymbol{k}})^2}{1-i\omega\tau^+_{\boldsymbol{k}}}f'(\varepsilon^+_{\boldsymbol{k}})I^{sk,1}(\boldsymbol{k},\boldsymbol{k}',\boldsymbol{k}'')$$
$$\times\Big[\frac{\tilde{v}^{++}_{a\boldsymbol{k}}v^{++}_{b\boldsymbol{k}'}\tilde{v}^{++}_{c\boldsymbol{k}}}{(1-i\omega\tau^+_{\boldsymbol{k}'})2h_{\boldsymbol{k}}}-\frac{\tilde{v}^{++}_{b\boldsymbol{k}'}\mathcal{W}_{ac\boldsymbol{k}}}{h_{\boldsymbol{k}}}\Big]\delta(\varepsilon^+_{\boldsymbol{k}}-\varepsilon^+_{\boldsymbol{k}'})\delta(\varepsilon^+_{\boldsymbol{k}}-\varepsilon^+_{\boldsymbol{k}''})+b\leftrightarrow c, \tag{112}$$

$$\mathcal{D}^{sk,1}_{(h)}(\omega) = \frac{ie^3\hbar}{2\pi}\int[dk]\int[dk']\int[dk'']\int_{-\infty}^{\infty}d\varepsilon f'(\varepsilon)\langle V^{++}_{\boldsymbol{k}\boldsymbol{k}''}V^{+-}_{\boldsymbol{k}''\boldsymbol{k}}\rangle\langle V^{-+}_{\boldsymbol{k}\boldsymbol{k}'}V^{++}_{\boldsymbol{k}'\boldsymbol{k}}\rangle$$
$$\times\Big[\tilde{v}^{++}_{a\boldsymbol{k}}v^{++}_{b\boldsymbol{k}'}\tilde{v}^{++}_{c\boldsymbol{k}}\tilde{G}^R_{+\boldsymbol{k}}(\varepsilon_1^+)\tilde{G}^R_{+\boldsymbol{k}''}(\varepsilon_1^+)\tilde{G}^R_{-\boldsymbol{k}}(\varepsilon_1^+)\tilde{G}^R_{+\boldsymbol{k}'}(\varepsilon_1^+)\tilde{G}^R_{+\boldsymbol{k}'}(\varepsilon)\tilde{G}^R_{+\boldsymbol{k}}(\varepsilon)\tilde{G}^A_{+\boldsymbol{k}}(\varepsilon)$$
$$+\tilde{v}^{++}_{b\boldsymbol{k}'}\mathcal{W}_{ac\boldsymbol{k}}\tilde{G}^R_{+\boldsymbol{k}}(\varepsilon)\tilde{G}^R_{+\boldsymbol{k}''}(\varepsilon)\tilde{G}^R_{-\boldsymbol{k}}(\varepsilon)\tilde{G}^R_{+\boldsymbol{k}'}(\varepsilon)\tilde{G}^A_{+\boldsymbol{k}'}(\varepsilon)\tilde{G}^A_{+\boldsymbol{k}}(\varepsilon)\tilde{G}^A_{+\boldsymbol{k}}(\varepsilon_1^-)\Big] + b\leftrightarrow c$$
$$\simeq -\frac{\pi^2e^3}{\hbar^2}\int[dk]\int[dk']\int[dk'']\frac{\tau^+_{\boldsymbol{k}'}(\tau^+_{\boldsymbol{k}})^2}{1-i\omega\tau^+_{\boldsymbol{k}}}f'(\varepsilon^+_{\boldsymbol{k}})I^{sk,1}(\boldsymbol{k},\boldsymbol{k}',\boldsymbol{k}'')$$
$$\times\Big[\frac{\tilde{v}^{++}_{a\boldsymbol{k}}v^{++}_{b\boldsymbol{k}'}\tilde{v}^{++}_{c\boldsymbol{k}}}{(1-i\omega\tau^+_{\boldsymbol{k}'})(2h_{\boldsymbol{k}}+\hbar\omega)}\delta(\varepsilon^+_{\boldsymbol{k}}-\varepsilon^+_{\boldsymbol{k}''}+\hbar\omega)+\frac{\tilde{v}^{++}_{b\boldsymbol{k}'}\mathcal{W}_{ac\boldsymbol{k}}}{h_{\boldsymbol{k}}}\delta(\varepsilon^+_{\boldsymbol{k}}-\varepsilon^+_{\boldsymbol{k}''})\Big]\delta(\varepsilon^+_{\boldsymbol{k}}-\varepsilon^+_{\boldsymbol{k}'})+b\leftrightarrow c, \tag{113}$$

$$\mathcal{D}^{sk,1}_{(i)}(\omega) = \frac{ie^3\hbar}{2\pi}\int[dk]\int[dk']\int_{-\infty}^{\infty}d\varepsilon f'(\varepsilon)\langle V^{++}_{\boldsymbol{k}\boldsymbol{k}'}V^{-+}_{\boldsymbol{k}'\boldsymbol{k}}\rangle\langle V^{++}_{\boldsymbol{k}'\boldsymbol{k}''}V^{+-}_{\boldsymbol{k}''\boldsymbol{k}'}\rangle$$
$$\times\Big[\tilde{v}^{++}_{a\boldsymbol{k}}v^{++}_{b\boldsymbol{k}'}\tilde{v}^{++}_{c\boldsymbol{k}}\tilde{G}^R_{+\boldsymbol{k}}(\varepsilon_1^+)\tilde{G}^R_{+\boldsymbol{k}'}(\varepsilon_1^+)\tilde{G}^R_{+\boldsymbol{k}'}(\varepsilon)\tilde{G}^R_{+\boldsymbol{k}''}(\varepsilon)\tilde{G}^R_{-\boldsymbol{k}'}(\varepsilon)\tilde{G}^R_{+\boldsymbol{k}}(\varepsilon)\tilde{G}^A_{+\boldsymbol{k}}(\varepsilon)$$
$$+\tilde{v}^{++}_{b\boldsymbol{k}'}\mathcal{W}_{ac\boldsymbol{k}}\tilde{G}^R_{+\boldsymbol{k}}(\varepsilon)\tilde{G}^R_{+\boldsymbol{k}'}(\varepsilon)\tilde{G}^A_{+\boldsymbol{k}'}(\varepsilon)\tilde{G}^A_{+\boldsymbol{k}''}(\varepsilon)\tilde{G}^A_{-\boldsymbol{k}'}(\varepsilon)\tilde{G}^A_{+\boldsymbol{k}}(\varepsilon)\tilde{G}^A_{+\boldsymbol{k}}(\varepsilon_1^-)\Big] + b\leftrightarrow c$$
$$\simeq \frac{\pi^2e^3}{\hbar^2}\int[dk]\int[dk']\int[dk'']\frac{\tau^+_{\boldsymbol{k}'}(\tau^+_{\boldsymbol{k}})^2}{1-i\omega\tau^+_{\boldsymbol{k}}}f'(\varepsilon^+_{\boldsymbol{k}})I^{sk,1}(\boldsymbol{k}',\boldsymbol{k}'',\boldsymbol{k})$$
$$\times\Big[\frac{\tilde{v}^{++}_{a\boldsymbol{k}}v^{++}_{b\boldsymbol{k}'}\tilde{v}^{++}_{c\boldsymbol{k}}}{(1-i\omega\tau^+_{\boldsymbol{k}'})2h_{\boldsymbol{k}'}}-\frac{\tilde{v}^{++}_{b\boldsymbol{k}'}\mathcal{W}_{ac\boldsymbol{k}}}{h_{\boldsymbol{k}'}}\Big]\delta(\varepsilon^+_{\boldsymbol{k}}-\varepsilon^+_{\boldsymbol{k}'})\delta(\varepsilon^+_{\boldsymbol{k}}-\varepsilon^+_{\boldsymbol{k}''})+b\leftrightarrow c, \tag{114}$$

$$\mathcal{D}^{sk,1}_{(j)}(\omega) = \frac{ie^3\hbar}{2\pi}\int[dk]\int[dk']\int[dk'']\int_{-\infty}^{\infty} d\varepsilon f'_{-}(\varepsilon)\langle V^{+-}_{\boldsymbol{k}\boldsymbol{k}'}V^{++}_{\boldsymbol{k}'\boldsymbol{k}}\rangle\langle V^{-+}_{\boldsymbol{k}'\boldsymbol{k}''}V^{++}_{\boldsymbol{k}''\boldsymbol{k}'}\rangle$$
$$\times\Big[\tilde{v}^{++}_{a\boldsymbol{k}}v^{++}_{b\boldsymbol{k}'}\tilde{v}^{++}_{c\boldsymbol{k}}\tilde{G}^R_{+\boldsymbol{k}}(\varepsilon_1^+)\tilde{G}^R_{-\boldsymbol{k}'}(\varepsilon_1^+)\tilde{G}^R_{+\boldsymbol{k}''}(\varepsilon_1^+)\tilde{G}^R_{+\boldsymbol{k}'}(\varepsilon_1^+)\tilde{G}^R_{+\boldsymbol{k}'}(\varepsilon)\tilde{G}^R_{+\boldsymbol{k}}(\varepsilon)\tilde{G}^A_{+\boldsymbol{k}}(\varepsilon)$$
$$+\tilde{v}^{++}_{b\boldsymbol{k}'}\mathcal{W}_{ac\boldsymbol{k}}\tilde{G}^R_{+\boldsymbol{k}}(\varepsilon)\tilde{G}^R_{-\boldsymbol{k}'}(\varepsilon)\tilde{G}^R_{+\boldsymbol{k}''}(\varepsilon)\tilde{G}^R_{+\boldsymbol{k}'}(\varepsilon)\tilde{G}^A_{+\boldsymbol{k}'}(\varepsilon)\tilde{G}^A_{+\boldsymbol{k}}(\varepsilon)\tilde{G}^A_{+\boldsymbol{k}}(\varepsilon_1^-)\Big] + b\leftrightarrow c$$
$$\simeq -\frac{\pi^2e^3}{\hbar^2}\int[dk]\int[dk']\int[dk'']\frac{\tau^+_{\boldsymbol{k}'}(\tau^+_{\boldsymbol{k}})^2}{1-i\omega\tau^+_{\boldsymbol{k}}}f'(\varepsilon^+_{\boldsymbol{k}})I^{sk,1}(\boldsymbol{k}',\boldsymbol{k}'',\boldsymbol{k})$$
$$\times\Big[\frac{\tilde{v}^{++}_{a\boldsymbol{k}}v^{++}_{b\boldsymbol{k}'}\tilde{v}^{++}_{c\boldsymbol{k}}}{(1-i\omega\tau^+_{\boldsymbol{k}'})(2h_{\boldsymbol{k}'}+\hbar\omega)}\delta(\varepsilon^+_{\boldsymbol{k}}-\varepsilon^+_{\boldsymbol{k}''}+\hbar\omega)+\frac{\tilde{v}^{++}_{b\boldsymbol{k}'}\mathcal{W}_{ac\boldsymbol{k}}}{h_{\boldsymbol{k}'}}\delta(\varepsilon^+_{\boldsymbol{k}}-\varepsilon^+_{\boldsymbol{k}''})\Big]\delta(\varepsilon^+_{\boldsymbol{k}}-\varepsilon^+_{\boldsymbol{k}'})+b\leftrightarrow c, \quad (115)$$

$$\mathcal{D}^{sk,1}_{(k)}(\omega) = \frac{ie^3\hbar}{2\pi}\int[dk]\int[dk']\int[dk'']\int_{-\infty}^{\infty} d\varepsilon f'(\varepsilon)\langle V^{++}_{\boldsymbol{k}\boldsymbol{k}''}V^{-+}_{\boldsymbol{k}''\boldsymbol{k}}\rangle\langle V^{++}_{\boldsymbol{k}''\boldsymbol{k}'}V^{+-}_{\boldsymbol{k}'\boldsymbol{k}''}\rangle$$
$$\times\Big[\tilde{v}^{++}_{a\boldsymbol{k}}v^{++}_{b\boldsymbol{k}'}\tilde{v}^{++}_{c\boldsymbol{k}}\tilde{G}^R_{+\boldsymbol{k}}(\varepsilon_1^+)\tilde{G}^R_{+\boldsymbol{k}''}(\varepsilon_1^+)\tilde{G}^R_{+\boldsymbol{k}'}(\varepsilon_1^+)\tilde{G}^R_{+\boldsymbol{k}'}(\varepsilon)\tilde{G}^R_{-\boldsymbol{k}''}(\varepsilon)\tilde{G}^R_{+\boldsymbol{k}}(\varepsilon)\tilde{G}^A_{+\boldsymbol{k}}(\varepsilon)$$
$$+\tilde{v}^{++}_{b\boldsymbol{k}'}\mathcal{W}_{ac\boldsymbol{k}}\tilde{G}^R_{+\boldsymbol{k}}(\varepsilon)\tilde{G}^R_{+\boldsymbol{k}''}(\varepsilon)\tilde{G}^R_{+\boldsymbol{k}'}(\varepsilon)\tilde{G}^A_{+\boldsymbol{k}'}(\varepsilon)\tilde{G}^A_{-\boldsymbol{k}''}(\varepsilon)\tilde{G}^A_{+\boldsymbol{k}}(\varepsilon)\tilde{G}^A_{+\boldsymbol{k}}(\varepsilon_1^-)\Big] + b\leftrightarrow c$$
$$\simeq -\frac{\pi^2e^3}{\hbar^2}\int[dk]\int[dk']\int[dk'']\frac{\tau^+_{\boldsymbol{k}'}(\tau^+_{\boldsymbol{k}})^2}{1-i\omega\tau^+_{\boldsymbol{k}}}f'(\varepsilon^+_{\boldsymbol{k}})I^{sk,1}(\boldsymbol{k}'',\boldsymbol{k},\boldsymbol{k}')$$
$$\times\Big[\frac{\tilde{v}^{++}_{a\boldsymbol{k}}v^{++}_{b\boldsymbol{k}'}\tilde{v}^{++}_{c\boldsymbol{k}}}{(1-i\omega\tau^+_{\boldsymbol{k}'})(\varepsilon^+_{\boldsymbol{k}}-\varepsilon^-_{\boldsymbol{k}''})}\delta(\varepsilon^+_{\boldsymbol{k}}-\varepsilon^+_{\boldsymbol{k}''}+\hbar\omega)+\frac{\tilde{v}^{++}_{b\boldsymbol{k}'}\mathcal{W}_{ac\boldsymbol{k}}}{h_{\boldsymbol{k}''}}\delta(\varepsilon^+_{\boldsymbol{k}}-\varepsilon^+_{\boldsymbol{k}''})\Big]\delta(\varepsilon^+_{\boldsymbol{k}}-\varepsilon^+_{\boldsymbol{k}'})+b\leftrightarrow c, \quad (116)$$

$$\mathcal{D}^{sk,1}_{(l)}(\omega) = \frac{ie^3\hbar}{2\pi}\int[dk]\int[dk']\int[dk'']\int_{-\infty}^{\infty} d\varepsilon f'(\varepsilon)\langle V^{+-}_{\boldsymbol{k}\boldsymbol{k}''}V^{++}_{\boldsymbol{k}''\boldsymbol{k}}\rangle\langle V^{-+}_{\boldsymbol{k}''\boldsymbol{k}'}V^{++}_{\boldsymbol{k}'\boldsymbol{k}''}\rangle$$
$$\times\Big[\tilde{v}^{++}_{a\boldsymbol{k}}v^{++}_{b\boldsymbol{k}'}\tilde{v}^{++}_{c\boldsymbol{k}}\tilde{G}^R_{+\boldsymbol{k}}(\varepsilon_1^+)\tilde{G}^R_{-\boldsymbol{k}''}(\varepsilon_1^+)\tilde{G}^R_{+\boldsymbol{k}'}(\varepsilon_1^+)\tilde{G}^R_{+\boldsymbol{k}'}(\varepsilon)\tilde{G}^R_{+\boldsymbol{k}''}(\varepsilon)\tilde{G}^R_{+\boldsymbol{k}}(\varepsilon)\tilde{G}^A_{+\boldsymbol{k}}(\varepsilon)$$
$$+\tilde{v}^{++}_{b\boldsymbol{k}'}\mathcal{W}_{ac\boldsymbol{k}}\tilde{G}^R_{+\boldsymbol{k}}(\varepsilon)\tilde{G}^R_{-\boldsymbol{k}''}(\varepsilon)\tilde{G}^R_{+\boldsymbol{k}'}(\varepsilon)\tilde{G}^A_{+\boldsymbol{k}'}(\varepsilon)\tilde{G}^A_{+\boldsymbol{k}''}(\varepsilon)\tilde{G}^A_{+\boldsymbol{k}}(\varepsilon)\tilde{G}^A_{+\boldsymbol{k}}(\varepsilon_1^-)\Big] + b\leftrightarrow c$$
$$\simeq \frac{\pi^2e^3}{\hbar^2}\int[dk]\int[dk']\int[dk'']\frac{\tau^+_{\boldsymbol{k}'}(\tau^+_{\boldsymbol{k}})^2}{1-i\omega\tau^+_{\boldsymbol{k}}}f'(\varepsilon^+_{\boldsymbol{k}})I^{sk,1}(\boldsymbol{k}'',\boldsymbol{k},\boldsymbol{k}')$$
$$\times\Big[\frac{\tilde{v}^{++}_{a\boldsymbol{k}}v^{++}_{b\boldsymbol{k}'}\tilde{v}^{++}_{c\boldsymbol{k}}}{(1-i\omega\tau^+_{\boldsymbol{k}'})(2h_{\boldsymbol{k}''}+\hbar\omega)}-\frac{\tilde{v}^{++}_{b\boldsymbol{k}'}\mathcal{W}_{ac\boldsymbol{k}}}{h_{\boldsymbol{k}''}}\Big]\delta(\varepsilon^+_{\boldsymbol{k}}-\varepsilon^+_{\boldsymbol{k}'})\delta(\varepsilon^+_{\boldsymbol{k}}-\varepsilon^+_{\boldsymbol{k}''})+b\leftrightarrow c, \quad (117)$$

$$\mathcal{D}^{sk,1}_{(m)}(\omega) = \frac{ie^3\hbar}{2\pi}\int[dk]\int[dk']\int[dk'']\int_{-\infty}^{\infty} d\varepsilon f'(\varepsilon)\langle V^{++}_{\boldsymbol{k}\boldsymbol{k}'}V^{+-}_{\boldsymbol{k}'\boldsymbol{k}}\rangle\langle V^{-+}_{\boldsymbol{k}\boldsymbol{k}''}V^{++}_{\boldsymbol{k}''\boldsymbol{k}}\rangle$$
$$\times\Big[\mathcal{W}^{++}_{ab\boldsymbol{k}}\tilde{v}^{++}_{c\boldsymbol{k}'}\tilde{G}^R_{+\boldsymbol{k}}(\varepsilon_1^+)\tilde{G}^R_{+\boldsymbol{k}}(\varepsilon)\tilde{G}^R_{+\boldsymbol{k}'}(\varepsilon)\tilde{G}^A_{+\boldsymbol{k}'}(\varepsilon)\tilde{G}^A_{-\boldsymbol{k}}(\varepsilon)\tilde{G}^A_{+\boldsymbol{k}''}(\varepsilon)\tilde{G}^A_{+\boldsymbol{k}}(\varepsilon)$$
$$+\tilde{v}^{++}_{a\boldsymbol{k}}\tilde{v}^{++}_{b\boldsymbol{k}}v^{++}_{c\boldsymbol{k}'}\tilde{G}^R_{+\boldsymbol{k}}(\varepsilon)\tilde{G}^A_{+\boldsymbol{k}}(\varepsilon)\tilde{G}^A_{+\boldsymbol{k}'}(\varepsilon)\tilde{G}^A_{+\boldsymbol{k}'}(\varepsilon_1^-)\tilde{G}^A_{-\boldsymbol{k}}(\varepsilon_1^-)\tilde{G}^A_{+\boldsymbol{k}''}(\varepsilon_1^-)\tilde{G}^A_{+\boldsymbol{k}}(\varepsilon_1^-)\Big] + b\leftrightarrow c$$
$$\simeq \frac{\pi^2e^3}{\hbar^2}\int[dk]\int[dk']\int[dk'']\frac{\tau^+_{\boldsymbol{k}'}(\tau^+_{\boldsymbol{k}})^2}{1-i\omega\tau^+_{\boldsymbol{k}}}f'(\varepsilon^+_{\boldsymbol{k}})I^{sk,1}(\boldsymbol{k},\boldsymbol{k}',\boldsymbol{k}'')$$
$$\times\Big[\frac{\mathcal{W}^{++}_{ab\boldsymbol{k}}\tilde{v}^{++}_{c\boldsymbol{k}'}}{h_{\boldsymbol{k}}}\delta(\varepsilon^+_{\boldsymbol{k}}-\varepsilon^+_{\boldsymbol{k}''})+\frac{\tilde{v}^{++}_{a\boldsymbol{k}}\tilde{v}^{++}_{b\boldsymbol{k}}v^{++}_{c\boldsymbol{k}'}}{(1-i\omega\tau^+_{\boldsymbol{k}'})(2h_{\boldsymbol{k}}-\hbar\omega)}\delta(\varepsilon^+_{\boldsymbol{k}}-\varepsilon^+_{\boldsymbol{k}''}-\hbar\omega)\Big]\delta(\varepsilon^+_{\boldsymbol{k}}-\varepsilon^+_{\boldsymbol{k}'})+b\leftrightarrow c, \quad (118)$$

$$\begin{aligned}\mathcal{D}^{sk,1}_{(n)}(\omega) &= \frac{ie^3\hbar}{2\pi}\int[dk]\int[dk']\int[dk'']\int_{-\infty}^{\infty} d\varepsilon f'(\varepsilon)\langle V^{++}_{\boldsymbol{k}\boldsymbol{k}''}V^{+-}_{\boldsymbol{k}''\boldsymbol{k}}\rangle\langle V^{-+}_{\boldsymbol{k}\boldsymbol{k}'}V^{++}_{\boldsymbol{k}'\boldsymbol{k}}\rangle\\
&\times\Big[\mathcal{W}^{++}_{ab\boldsymbol{k}}\tilde{v}^{++}_{c\boldsymbol{k}'}\tilde{G}^R_{+\boldsymbol{k}}(\varepsilon_1^+)\tilde{G}^R_{+\boldsymbol{k}}(\varepsilon)\tilde{G}^R_{+\boldsymbol{k}''}(\varepsilon)\tilde{G}^R_{-\boldsymbol{k}}(\varepsilon)\tilde{G}^R_{+\boldsymbol{k}'}(\varepsilon)\tilde{G}^A_{+\boldsymbol{k}'}(\varepsilon)\tilde{G}^A_{+\boldsymbol{k}}(\varepsilon)\\
&+\tilde{v}^{++}_{a\boldsymbol{k}}\tilde{v}^{++}_{b\boldsymbol{k}}v^{++}_{c\boldsymbol{k}'}\tilde{G}^R_{+\boldsymbol{k}}(\varepsilon)\tilde{G}^A_{+\boldsymbol{k}}(\varepsilon)\tilde{G}^A_{+\boldsymbol{k}''}(\varepsilon)\tilde{G}^A_{-\boldsymbol{k}}(\varepsilon)\tilde{G}^A_{+\boldsymbol{k}'}(\varepsilon)\tilde{G}^A_{+\boldsymbol{k}'}(\varepsilon_1^-)\tilde{G}^A_{+\boldsymbol{k}}(\varepsilon_1^-)\Big] + b\leftrightarrow c\\
&\simeq \frac{\pi^2e^3}{\hbar^2}\int[dk]\int[dk']\int[dk'']\frac{\tau^+_{\boldsymbol{k}'}(\tau^+_{\boldsymbol{k}})^2}{1-i\omega\tau^+_{\boldsymbol{k}}}f'(\varepsilon^+_{\boldsymbol{k}})I^{sk,1}(\boldsymbol{k},\boldsymbol{k}',\boldsymbol{k}'')\\
&\times\Big[\frac{\mathcal{W}^{++}_{ab\boldsymbol{k}}\tilde{v}^{++}_{c\boldsymbol{k}'}}{h_{\boldsymbol{k}}} - \frac{\tilde{v}^{++}_{a\boldsymbol{k}}\tilde{v}^{++}_{b\boldsymbol{k}}v^{++}_{c\boldsymbol{k}'}}{(1-i\omega\tau^+_{\boldsymbol{k}'})2h_{\boldsymbol{k}}}\Big]\delta(\varepsilon^+_{\boldsymbol{k}}-\varepsilon^+_{\boldsymbol{k}'})\delta(\varepsilon^+_{\boldsymbol{k}}-\varepsilon^+_{\boldsymbol{k}''}) + b\leftrightarrow c,\end{aligned}\tag{119}$$

$$\begin{aligned}\mathcal{D}^{sk,1}_{(o)}(\omega) &= \frac{ie^3\hbar}{2\pi}\int[dk]\int[dk']\int[dk'']\int_{-\infty}^{\infty} d\varepsilon f'(\varepsilon)\langle V^{++}_{\boldsymbol{k}\boldsymbol{k}'}V^{-+}_{\boldsymbol{k}'\boldsymbol{k}}\rangle\langle V^{++}_{\boldsymbol{k}'\boldsymbol{k}''}V^{+-}_{\boldsymbol{k}''\boldsymbol{k}'}\rangle\\
&\times\Big[\mathcal{W}^{++}_{ab\boldsymbol{k}}\tilde{v}^{++}_{c\boldsymbol{k}'}\tilde{G}^R_{+\boldsymbol{k}}(\varepsilon_1^+)\tilde{G}^R_{+\boldsymbol{k}}(\varepsilon)\tilde{G}^R_{+\boldsymbol{k}'}(\varepsilon)\tilde{G}^A_{+\boldsymbol{k}'}(\varepsilon)\tilde{G}^A_{+\boldsymbol{k}''}(\varepsilon)\tilde{G}^A_{-\boldsymbol{k}'}(\varepsilon)\tilde{G}^A_{+\boldsymbol{k}}(\varepsilon)\\
&+\tilde{v}^{++}_{a\boldsymbol{k}}\tilde{v}^{++}_{b\boldsymbol{k}}v^{++}_{c\boldsymbol{k}'}\tilde{G}^R_{+\boldsymbol{k}}(\varepsilon)\tilde{G}^A_{+\boldsymbol{k}}(\varepsilon)\tilde{G}^A_{+\boldsymbol{k}'}(\varepsilon)\tilde{G}^A_{+\boldsymbol{k}'}(\varepsilon_1^-)\tilde{G}^A_{+\boldsymbol{k}''}(\varepsilon_1^-)\tilde{G}^A_{-\boldsymbol{k}'}(\varepsilon_1^-)\tilde{G}^A_{+\boldsymbol{k}}(\varepsilon_1^-)\Big] + b\leftrightarrow c\\
&\simeq \frac{\pi^2e^3}{\hbar^2}\int[dk]\int[dk']\int[dk'']\frac{\tau^+_{\boldsymbol{k}'}(\tau^+_{\boldsymbol{k}})^2}{1-i\omega\tau^+_{\boldsymbol{k}}}f'(\varepsilon^+_{\boldsymbol{k}})I^{sk,1}(\boldsymbol{k}',\boldsymbol{k}'',\boldsymbol{k})\\
&\times\Big[\frac{\mathcal{W}^{++}_{ab\boldsymbol{k}}\tilde{v}^{++}_{c\boldsymbol{k}'}}{h_{\boldsymbol{k}'}}\delta(\varepsilon^+_{\boldsymbol{k}}-\varepsilon^+_{\boldsymbol{k}''}) + \frac{\tilde{v}^{++}_{a\boldsymbol{k}}\tilde{v}^{++}_{b\boldsymbol{k}}v^{++}_{c\boldsymbol{k}'}}{(1-i\omega\tau^+_{\boldsymbol{k}'})(2h_{\boldsymbol{k}'}-\hbar\omega)}\delta(\varepsilon^+_{\boldsymbol{k}}-\varepsilon^+_{\boldsymbol{k}''}-\hbar\omega)\Big]\delta(\varepsilon^+_{\boldsymbol{k}}-\varepsilon^+_{\boldsymbol{k}'}) + b\leftrightarrow c,\end{aligned}\tag{120}$$

$$\begin{aligned}\mathcal{D}^{sk,1}_{(p)}(\omega) &= \frac{ie^3\hbar}{2\pi}\int[dk]\int[dk']\int[dk'']\int_{-\infty}^{\infty} d\varepsilon f'(\varepsilon)\langle V^{+-}_{\boldsymbol{k}\boldsymbol{k}'}V^{++}_{\boldsymbol{k}'\boldsymbol{k}}\rangle\langle V^{-+}_{\boldsymbol{k}'\boldsymbol{k}''}V^{++}_{\boldsymbol{k}''\boldsymbol{k}'}\rangle\\
&\times\Big[\mathcal{W}^{++}_{ab\boldsymbol{k}}\tilde{v}^{++}_{c\boldsymbol{k}'}\tilde{G}^R_{+\boldsymbol{k}}(\varepsilon_1^+)\tilde{G}^R_{+\boldsymbol{k}}(\varepsilon)\tilde{G}^R_{-\boldsymbol{k}'}(\varepsilon)\tilde{G}^R_{+\boldsymbol{k}''}(\varepsilon)\tilde{G}^R_{+\boldsymbol{k}'}(\varepsilon)\tilde{G}^A_{+\boldsymbol{k}'}(\varepsilon)\tilde{G}^A_{+\boldsymbol{k}}(\varepsilon)\\
&+\tilde{v}^{++}_{a\boldsymbol{k}}\tilde{v}^{++}_{b\boldsymbol{k}}v^{++}_{c\boldsymbol{k}'}\tilde{G}^R_{+\boldsymbol{k}}(\varepsilon)\tilde{G}^A_{+\boldsymbol{k}}(\varepsilon)\tilde{G}^A_{-\boldsymbol{k}'}(\varepsilon)\tilde{G}^A_{+\boldsymbol{k}''}(\varepsilon)\tilde{G}^A_{+\boldsymbol{k}'}(\varepsilon)\tilde{G}^A_{+\boldsymbol{k}'}(\varepsilon_1^-)\tilde{G}^A_{+\boldsymbol{k}}(\varepsilon_1^-)\Big] + b\leftrightarrow c\\
&\simeq \frac{\pi^2e^3}{\hbar^2}\int[dk]\int[dk']\int[dk'']\frac{\tau^+_{\boldsymbol{k}'}(\tau^+_{\boldsymbol{k}})^2}{1-i\omega\tau^+_{\boldsymbol{k}}}f'(\varepsilon^+_{\boldsymbol{k}})I^{sk,1}(\boldsymbol{k}',\boldsymbol{k}'',\boldsymbol{k})\\
&\times\Big[\frac{\mathcal{W}^{++}_{ab\boldsymbol{k}}\tilde{v}^{++}_{c\boldsymbol{k}'}}{h_{\boldsymbol{k}'}} - \frac{\tilde{v}^{++}_{a\boldsymbol{k}}\tilde{v}^{++}_{b\boldsymbol{k}}v^{++}_{c\boldsymbol{k}'}}{(1-i\omega\tau^+_{\boldsymbol{k}'})2h_{\boldsymbol{k}'}}\Big]\delta(\varepsilon^+_{\boldsymbol{k}}-\varepsilon^+_{\boldsymbol{k}'})\delta(\varepsilon^+_{\boldsymbol{k}}-\varepsilon^+_{\boldsymbol{k}''}) + b\leftrightarrow c,\end{aligned}\tag{121}$$

$$\begin{aligned}\mathcal{D}^{sk,1}_{(q)}(\omega) &= \frac{ie^3\hbar}{2\pi}\int[dk]\int[dk']\int[dk'']\int_{-\infty}^{\infty} d\varepsilon f'(\varepsilon)\langle V^{++}_{\boldsymbol{k}\boldsymbol{k}''}V^{-+}_{\boldsymbol{k}''\boldsymbol{k}}\rangle\langle V^{++}_{\boldsymbol{k}''\boldsymbol{k}'}V^{+-}_{\boldsymbol{k}'\boldsymbol{k}''}\rangle\\
&\times\Big[\mathcal{W}^{++}_{ab\boldsymbol{k}}\tilde{v}^{++}_{c\boldsymbol{k}'}\tilde{G}^R_{+\boldsymbol{k}}(\varepsilon_1^+)\tilde{G}^R_{+\boldsymbol{k}}(\varepsilon)\tilde{G}^R_{+\boldsymbol{k}''}(\varepsilon)\tilde{G}^R_{+\boldsymbol{k}'}(\varepsilon)\tilde{G}^A_{+\boldsymbol{k}'}(\varepsilon)\tilde{G}^A_{-\boldsymbol{k}''}(\varepsilon)\tilde{G}^A_{+\boldsymbol{k}}(\varepsilon)\\
&+\tilde{v}^{++}_{a\boldsymbol{k}}\tilde{v}^{++}_{b\boldsymbol{k}}v^{++}_{c\boldsymbol{k}'}\tilde{G}^R_{+\boldsymbol{k}}(\varepsilon)\tilde{G}^A_{+\boldsymbol{k}}(\varepsilon)\tilde{G}^A_{+\boldsymbol{k}''}(\varepsilon)\tilde{G}^A_{+\boldsymbol{k}'}(\varepsilon)\tilde{G}^A_{+\boldsymbol{k}'}(\varepsilon_1^-)\tilde{G}^A_{-\boldsymbol{k}''}(\varepsilon_1^-)\tilde{G}^A_{+\boldsymbol{k}}(\varepsilon_1^-)\Big] + b\leftrightarrow c\\
&\simeq \frac{\pi^2e^3}{\hbar^2}\int[dk]\int[dk']\int[dk'']\frac{\tau^+_{\boldsymbol{k}'}(\tau^+_{\boldsymbol{k}})^2}{1-i\omega\tau^+_{\boldsymbol{k}}}f'(\varepsilon^+_{\boldsymbol{k}})I^{sk,1}(\boldsymbol{k}'',\boldsymbol{k},\boldsymbol{k}')\\
&\times\Big[\frac{\mathcal{W}^{++}_{ab\boldsymbol{k}}\tilde{v}^{++}_{c\boldsymbol{k}'}}{h_{\boldsymbol{k}''}} - \frac{\tilde{v}^{++}_{a\boldsymbol{k}}\tilde{v}^{++}_{b\boldsymbol{k}}v^{++}_{c\boldsymbol{k}'}}{(1-i\omega\tau^+_{\boldsymbol{k}'})(2h_{\boldsymbol{k}''}-\hbar\omega)}\Big]\delta(\varepsilon^+_{\boldsymbol{k}}-\varepsilon^+_{\boldsymbol{k}'})\delta(\varepsilon^+_{\boldsymbol{k}}-\varepsilon^+_{\boldsymbol{k}''}) + b\leftrightarrow c,\end{aligned}\tag{122}$$

$$\mathcal{D}^{sk,1}_{(r)}(\omega) = \frac{ie^3\hbar}{2\pi}\int[dk]\int[dk']\int[dk'']\int_{-\infty}^{\infty} d\varepsilon f'(\varepsilon)\langle V^{+-}_{\boldsymbol{k}\boldsymbol{k}''}V^{++}_{\boldsymbol{k}''\boldsymbol{k}}\rangle\langle V^{-+}_{\boldsymbol{k}''\boldsymbol{k}'}V^{++}_{\boldsymbol{k}'\boldsymbol{k}''}\rangle$$
$$\times\Big[\mathcal{W}^{++}_{ab\boldsymbol{k}}\tilde{v}^{++}_{c\boldsymbol{k}'}\tilde{G}^R_{+\boldsymbol{k}}(\varepsilon_1^+)\tilde{G}^R_{+\boldsymbol{k}}(\varepsilon)\tilde{G}^R_{-\boldsymbol{k}''}(\varepsilon)\tilde{G}^R_{+\boldsymbol{k}'}(\varepsilon)\tilde{G}^A_{+\boldsymbol{k}'}(\varepsilon)\tilde{G}^A_{+\boldsymbol{k}''}(\varepsilon)\tilde{G}^A_{+\boldsymbol{k}}(\varepsilon)$$
$$+\tilde{v}^{++}_{a\boldsymbol{k}}\tilde{v}^{++}_{b\boldsymbol{k}}v^{++}_{c\boldsymbol{k}'}\tilde{G}^R_{+\boldsymbol{k}}(\varepsilon)\tilde{G}^A_{+\boldsymbol{k}}(\varepsilon)\tilde{G}^A_{-\boldsymbol{k}''}(\varepsilon)\tilde{G}^A_{+\boldsymbol{k}'}(\varepsilon)\tilde{G}^A_{+\boldsymbol{k}'}(\varepsilon_1^-)\tilde{G}^A_{+\boldsymbol{k}''}(\varepsilon_1^-)\tilde{G}^A_{+\boldsymbol{k}}(\varepsilon_1^-)\Big] + b\leftrightarrow c$$
$$\simeq \frac{\pi^2e^3}{\hbar^2}\int[dk]\int[dk']\int[dk'']\frac{\tau^+_{\boldsymbol{k}'}(\tau^+_{\boldsymbol{k}})^2}{1-i\omega\tau^+_{\boldsymbol{k}}}f'(\varepsilon^+_{\boldsymbol{k}})I^{sk,1}(\boldsymbol{k}'',\boldsymbol{k},\boldsymbol{k}')$$
$$\Big[\frac{\mathcal{W}^{++}_{ab\boldsymbol{k}}\tilde{v}^{++}_{c\boldsymbol{k}'}}{h_{\boldsymbol{k}''}}\delta(\varepsilon^+_{\boldsymbol{k}}-\varepsilon^+_{\boldsymbol{k}''}) + \frac{\tilde{v}^{++}_{a\boldsymbol{k}}\tilde{v}^{++}_{b\boldsymbol{k}}v^{++}_{c\boldsymbol{k}'}}{(1-i\omega\tau^+_{\boldsymbol{k}'})(\varepsilon^+_{\boldsymbol{k}}-\varepsilon^-_{\boldsymbol{k}''})}\delta(\varepsilon^+_{\boldsymbol{k}}-\varepsilon^+_{\boldsymbol{k}''}-\hbar\omega)\Big]\delta(\varepsilon^+_{\boldsymbol{k}}-\varepsilon^+_{\boldsymbol{k}'}) + b\leftrightarrow c, \tag{123}$$

$$\mathcal{D}^{sk,1}_{(s)}(\omega) = \frac{ie^3\hbar}{2\pi}\int[dk]\int[dk']\int[dk'']\int_{-\infty}^{\infty} d\varepsilon f'(\varepsilon)\tilde{v}^{++}_{a\boldsymbol{k}}\langle V^{+-}_{\boldsymbol{k}\boldsymbol{k}''}V^{++}_{\boldsymbol{k}''\boldsymbol{k}}\rangle\langle V^{-+}_{\boldsymbol{k}''\boldsymbol{k}'}V^{++}_{\boldsymbol{k}'\boldsymbol{k}''}\rangle$$
$$\times\Big[v^{++}_{b\boldsymbol{k}'}\tilde{v}^{++}_{c\boldsymbol{k}''}\tilde{G}^R_{+\boldsymbol{k}}(\varepsilon_1^+)\tilde{G}^R_{-\boldsymbol{k}''}(\varepsilon_1^+)\tilde{G}^R_{+\boldsymbol{k}'}(\varepsilon_1^+)\tilde{G}^R_{+\boldsymbol{k}'}(\varepsilon)\tilde{G}^R_{+\boldsymbol{k}''}(\varepsilon)\tilde{G}^A_{+\boldsymbol{k}''}(\varepsilon)\tilde{G}^A_{+\boldsymbol{k}}(\varepsilon)$$
$$+\tilde{v}^{++}_{b\boldsymbol{k}'}v^{++}_{c\boldsymbol{k}''}\tilde{G}^R_{+\boldsymbol{k}}(\varepsilon)\tilde{G}^R_{-\boldsymbol{k}''}(\varepsilon)\tilde{G}^R_{+\boldsymbol{k}'}(\varepsilon)\tilde{G}^A_{+\boldsymbol{k}'}(\varepsilon)\tilde{G}^A_{+\boldsymbol{k}''}(\varepsilon)\tilde{G}^A_{+\boldsymbol{k}''}(\varepsilon_1^-)\tilde{G}^A_{+\boldsymbol{k}}(\varepsilon_1^-)\Big] + b\leftrightarrow c$$
$$\simeq -\frac{2\pi^2e^3}{\hbar^2}\int[dk]\int[dk']\int[dk'']\frac{\tau^+_{\boldsymbol{k}}\tau^+_{\boldsymbol{k}'}\tau^+_{\boldsymbol{k}''}}{1-i\omega\tau^+_{\boldsymbol{k}}}f'(\varepsilon^+_{\boldsymbol{k}})I^{sk,1}(\boldsymbol{k}'',\boldsymbol{k},\boldsymbol{k}')\tilde{v}^{++}_{a\boldsymbol{k}}$$
$$\times\Big[\frac{v^{++}_{b\boldsymbol{k}'}\tilde{v}^{++}_{c\boldsymbol{k}''}}{(1-i\omega\tau^+_{\boldsymbol{k}'})(2h_{\boldsymbol{k}''}+\hbar\omega)} + \frac{\tilde{v}^{++}_{b\boldsymbol{k}'}v^{++}_{c\boldsymbol{k}''}}{(1-i\omega\tau^+_{\boldsymbol{k}''})2h_{\boldsymbol{k}''}}\Big]\delta(\varepsilon^+_{\boldsymbol{k}}-\varepsilon^+_{\boldsymbol{k}'})\delta(\varepsilon^+_{\boldsymbol{k}}-\varepsilon^+_{\boldsymbol{k}''}) + b\leftrightarrow c, \tag{124}$$

$$\mathcal{D}^{sk,1}_{(t)}(\omega) = \frac{ie^3\hbar}{2\pi}\int[dk]\int[dk']\int[dk'']\int_{-\infty}^{\infty} d\varepsilon f'(\varepsilon)\tilde{v}^{++}_{a\boldsymbol{k}}\langle V^{++}_{\boldsymbol{k}\boldsymbol{k}'}V^{-+}_{\boldsymbol{k}'\boldsymbol{k}}\rangle\langle V^{++}_{\boldsymbol{k}'\boldsymbol{k}''}V^{+-}_{\boldsymbol{k}''\boldsymbol{k}'}\rangle$$
$$\times\Big[v^{++}_{b\boldsymbol{k}'}\tilde{v}^{++}_{c\boldsymbol{k}''}\tilde{G}^R_{+\boldsymbol{k}}(\varepsilon_1^+)\tilde{G}^R_{+\boldsymbol{k}'}(\varepsilon_1^+)\tilde{G}^R_{+\boldsymbol{k}'}(\varepsilon)\tilde{G}^R_{+\boldsymbol{k}''}(\varepsilon)\tilde{G}^A_{+\boldsymbol{k}''}(\varepsilon)\tilde{G}^A_{-\boldsymbol{k}'}(\varepsilon)\tilde{G}^A_{+\boldsymbol{k}}(\varepsilon)$$
$$+\tilde{v}^{++}_{b\boldsymbol{k}'}v^{++}_{c\boldsymbol{k}''}\tilde{G}^R_{+\boldsymbol{k}}(\varepsilon)\tilde{G}^R_{+\boldsymbol{k}'}(\varepsilon)\tilde{G}^A_{+\boldsymbol{k}'}(\varepsilon)\tilde{G}^A_{+\boldsymbol{k}''}(\varepsilon)\tilde{G}^A_{+\boldsymbol{k}''}(\varepsilon_1^-)\tilde{G}^A_{-\boldsymbol{k}'}(\varepsilon_1^-)\tilde{G}^A_{+\boldsymbol{k}}(\varepsilon_1^-)\Big] + b\leftrightarrow c$$
$$\simeq -\frac{2\pi^2e^3}{\hbar^2}\int[dk]\int[dk']\int[dk'']\frac{\tau^+_{\boldsymbol{k}}\tau^+_{\boldsymbol{k}'}\tau^+_{\boldsymbol{k}''}}{1-i\omega\tau^+_{\boldsymbol{k}}}f'(\varepsilon^+_{\boldsymbol{k}})I^{sk,1}(\boldsymbol{k}',\boldsymbol{k}'',\boldsymbol{k})\tilde{v}^{++}_{a\boldsymbol{k}}$$
$$\times\Big[\frac{v^{++}_{b\boldsymbol{k}'}\tilde{v}^{++}_{c\boldsymbol{k}''}}{(1-i\omega\tau^+_{\boldsymbol{k}'})2h_{\boldsymbol{k}'}} + \frac{\tilde{v}^{++}_{b\boldsymbol{k}'}v^{++}_{c\boldsymbol{k}''}}{(1-i\omega\tau^+_{\boldsymbol{k}''})(2h_{\boldsymbol{k}'}-\hbar\omega)}\Big]\delta(\varepsilon^+_{\boldsymbol{k}}-\varepsilon^+_{\boldsymbol{k}'})\delta(\varepsilon^+_{\boldsymbol{k}}-\varepsilon^+_{\boldsymbol{k}''}) + b\leftrightarrow c, \tag{125}$$

$$\mathcal{D}^{sk,1}_{(u)}(\omega) = \frac{ie^3\hbar}{2\pi}\int[dk]\int[dk']\int[dk'']\int_{-\infty}^{\infty} d\varepsilon f'(\varepsilon)\tilde{v}^{++}_{a\boldsymbol{k}}\langle V^{++}_{\boldsymbol{k}\boldsymbol{k}'}V^{+-}_{\boldsymbol{k}'\boldsymbol{k}}\rangle\langle V^{-+}_{\boldsymbol{k}\boldsymbol{k}''}V^{++}_{\boldsymbol{k}''\boldsymbol{k}}\rangle$$
$$\times\Big[v^{++}_{b\boldsymbol{k}'}\tilde{v}^{++}_{c\boldsymbol{k}''}\tilde{G}^R_{+\boldsymbol{k}}(\varepsilon_1^+)\tilde{G}^R_{+\boldsymbol{k}'}(\varepsilon_1^+)\tilde{G}^R_{+\boldsymbol{k}'}(\varepsilon)\tilde{G}^R_{-\boldsymbol{k}}(\varepsilon)\tilde{G}^R_{+\boldsymbol{k}''}(\varepsilon)\tilde{G}^A_{+\boldsymbol{k}''}(\varepsilon)\tilde{G}^A_{+\boldsymbol{k}}(\varepsilon)$$
$$+\tilde{v}^{++}_{b\boldsymbol{k}'}v^{++}_{c\boldsymbol{k}''}\tilde{G}^R_{+\boldsymbol{k}}(\varepsilon)\tilde{G}^R_{+\boldsymbol{k}'}(\varepsilon)\tilde{G}^A_{+\boldsymbol{k}'}(\varepsilon)\tilde{G}^A_{-\boldsymbol{k}}(\varepsilon)\tilde{G}^A_{+\boldsymbol{k}''}(\varepsilon)\tilde{G}^A_{+\boldsymbol{k}''}(\varepsilon_1^-)\tilde{G}^A_{+\boldsymbol{k}}(\varepsilon_1^-)\Big] + b\leftrightarrow c$$
$$\simeq -\frac{2\pi^2e^3}{\hbar^2}\int[dk]\int[dk']\int[dk'']\frac{\tau^+_{\boldsymbol{k}}\tau^+_{\boldsymbol{k}'}\tau^+_{\boldsymbol{k}''}}{1-i\omega\tau^+_{\boldsymbol{k}}}f'(\varepsilon^+_{\boldsymbol{k}})I^{sk,1}(\boldsymbol{k},\boldsymbol{k}',\boldsymbol{k}'')\tilde{v}^{++}_{a\boldsymbol{k}}$$
$$\Big[\frac{v^{++}_{b\boldsymbol{k}'}\tilde{v}^{++}_{c\boldsymbol{k}''}}{(1-i\omega\tau^+_{\boldsymbol{k}'})2h_{\boldsymbol{k}}} + \frac{\tilde{v}^{++}_{b\boldsymbol{k}'}v^{++}_{c\boldsymbol{k}''}}{(1-i\omega\tau^+_{\boldsymbol{k}''})2h_{\boldsymbol{k}}}\Big]\delta(\varepsilon^+_{\boldsymbol{k}}-\varepsilon^+_{\boldsymbol{k}'})\delta(\varepsilon^+_{\boldsymbol{k}}-\varepsilon^+_{\boldsymbol{k}''}) + b\leftrightarrow c, \tag{126}$$

By combining all the relevant contributions in the above results, the intrinsic skew-scattering contribution can be found as

$$\Xi^{I\text{-}sk,1}_{abc} = \sum_{i=a}^{u}\frac{\partial\tilde{\mathcal{D}}^{sk,1}_{(i)}(\omega)}{\partial\omega}\Big|_{\omega=0}$$
$$= e^3\int[dk]\int[dk']\int[dk'']\tau^+_{\boldsymbol{k}}\tau^+_{\boldsymbol{k}'}f'(\varepsilon^+_{\boldsymbol{k}})\Big[\Big(\tau^+_{\boldsymbol{k}'}\tilde{v}^{++}_{a\boldsymbol{k}}\mathcal{W}^{++}_{bc\boldsymbol{k}'} - \frac{\tau^+_{\boldsymbol{k}}}{2}\tilde{v}^{++}_{a\boldsymbol{k}}\tilde{v}^{++}_{b\boldsymbol{k}}v^{++}_{c\boldsymbol{k}'}\Big)W^{sk,1\alpha}_{\boldsymbol{k}\boldsymbol{k}'\boldsymbol{k}''}\delta'(\varepsilon^+_{\boldsymbol{k}}-\varepsilon^+_{\boldsymbol{k}''})$$
$$-\Big(\tau^+_{\boldsymbol{k}'}\tilde{v}^{++}_{a\boldsymbol{k}}\mathcal{W}^{++}_{bc\boldsymbol{k}'}W^{sk,1\beta}_{\boldsymbol{k}\boldsymbol{k}'\boldsymbol{k}''} - \frac{\tau^+_{\boldsymbol{k}}}{2}\tilde{v}^{++}_{a\boldsymbol{k}}\tilde{v}^{++}_{b\boldsymbol{k}}v^{++}_{c\boldsymbol{k}'}W^{sk,1\gamma}_{\boldsymbol{k}\boldsymbol{k}'\boldsymbol{k}''}\Big)\delta(\varepsilon^+_{\boldsymbol{k}}-\varepsilon^+_{\boldsymbol{k}''})\Big]\delta(\varepsilon^+_{\boldsymbol{k}}-\varepsilon^+_{\boldsymbol{k}'}) + b\leftrightarrow c, \tag{127}$$

And the intrinsic skew-scattering contribution to χ^{I}_{abc} in the dc limit is

$$\chi^{I\text{-}sk,1}_{abc} = \frac{e^3}{4}\int[dk]\int[dk']\int[dk'']\tau^+_{\boldsymbol{k}}\tau^+_{\boldsymbol{k}'}f'(\varepsilon^+_{\boldsymbol{k}})\Big[\Big(\tau^+_{\boldsymbol{k}'}\tilde{v}^{++}_{a\boldsymbol{k}}\mathcal{W}^{++}_{bc\boldsymbol{k}'} - \frac{\tau^+_{\boldsymbol{k}}}{2}\tilde{v}^{++}_{a\boldsymbol{k}}\tilde{v}^{++}_{b\boldsymbol{k}}v^{++}_{c\boldsymbol{k}'}\Big)W^{sk,1\alpha}_{\boldsymbol{k}\boldsymbol{k}'\boldsymbol{k}''}\delta'(\varepsilon^+_{\boldsymbol{k}} - \varepsilon^+_{\boldsymbol{k}''})$$
$$-\Big(\tau^+_{\boldsymbol{k}'}\tilde{v}^{++}_{a\boldsymbol{k}}\mathcal{W}^{++}_{bc\boldsymbol{k}'}W^{sk,1\beta}_{\boldsymbol{k}\boldsymbol{k}'\boldsymbol{k}''} - \frac{\tau^+_{\boldsymbol{k}}}{2}\tilde{v}^{++}_{a\boldsymbol{k}}\tilde{v}^{++}_{b\boldsymbol{k}}v^{++}_{c\boldsymbol{k}'}W^{sk,1\gamma}_{\boldsymbol{k}\boldsymbol{k}'\boldsymbol{k}''}\Big)\delta(\varepsilon^+_{\boldsymbol{k}} - \varepsilon^+_{\boldsymbol{k}''})\Big]\delta(\varepsilon^+_{\boldsymbol{k}} - \varepsilon^+_{\boldsymbol{k}'}) + b \leftrightarrow c. \tag{128}$$

2. *Extrinsic skew-scattering contribution*

The extrinsic skew-scattering diagrams are summarised in Supplementary Fig. 9. By denoting the diagrams in

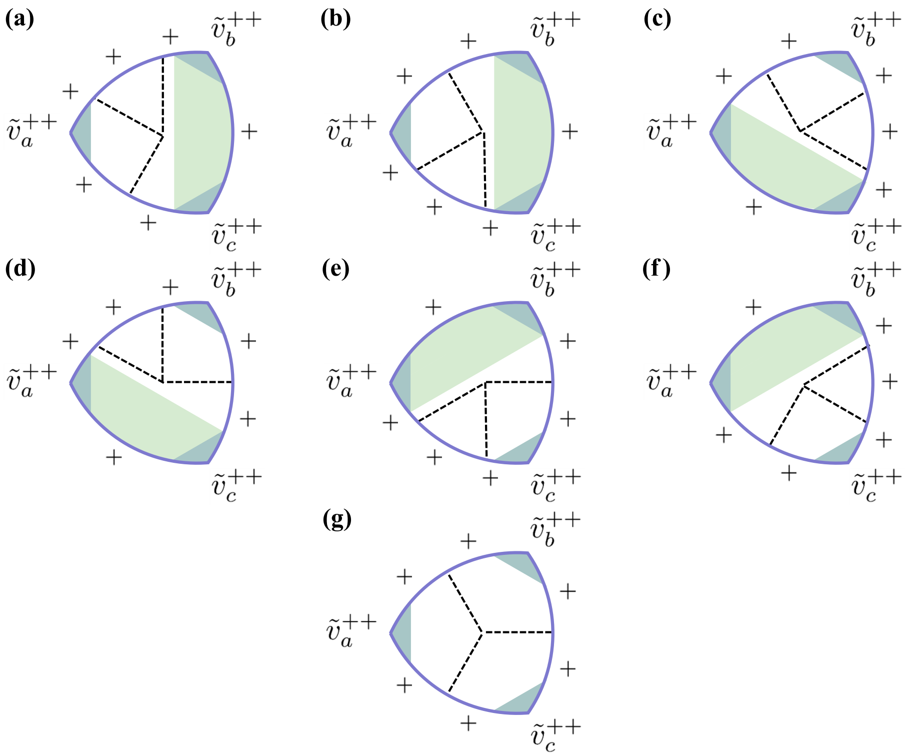

FIG. 9. Feynman diagrams of the extrinsic skew-scattering contribution to the nonlinear Hall effect.

Supplementary Fig. 9 (a)-(g) as $\tilde{\mathcal{D}}^{sk,2}_{(a)}(\omega)$-$\tilde{\mathcal{D}}^{sk,2}_{(g)}(\omega)$, we have

$$\begin{aligned}\mathcal{D}^{sk,2}_{(a)}(\omega) = &\frac{ie^3\hbar}{2\pi}\int[dk]\int[dk']\int[dk'']\int_{-\infty}^{\infty}d\varepsilon f'(\varepsilon)\tilde{v}^{++}_{a\boldsymbol{k}}\langle V^{++}_{\boldsymbol{k}\boldsymbol{k}''}V^{++}_{\boldsymbol{k}''\boldsymbol{k}'}V^{++}_{\boldsymbol{k}'\boldsymbol{k}}\rangle\\
&\times\Big[\mathcal{W}^{++}_{cb\boldsymbol{k}'}\tilde{G}^R_{+\boldsymbol{k}}(\varepsilon^+_1)\tilde{G}^R_{+\boldsymbol{k}''}(\varepsilon^+_1)\tilde{G}^R_{+\boldsymbol{k}'}(\varepsilon^+_1)\tilde{G}^R_{+\boldsymbol{k}'}(\varepsilon)\tilde{G}^A_{+\boldsymbol{k}'}(\varepsilon)\tilde{G}^A_{+\boldsymbol{k}}(\varepsilon)\\
&+\mathcal{W}^{++}_{bc\boldsymbol{k}'}\tilde{G}^R_{+\boldsymbol{k}}(\varepsilon)\tilde{G}^R_{+\boldsymbol{k}''}(\varepsilon)\tilde{G}^R_{+\boldsymbol{k}'}(\varepsilon)\tilde{G}^A_{+\boldsymbol{k}'}(\varepsilon)\tilde{G}^A_{+\boldsymbol{k}'}(\varepsilon^-_1)\tilde{G}^A_{+\boldsymbol{k}}(\varepsilon^-_1)\Big] + b\leftrightarrow c\\
\simeq &-\frac{2\pi^2e^3}{\hbar^2}\int[dk]\int[dk']\int[dk'']\frac{\tau^+_{\boldsymbol{k}}(\tau^+_{\boldsymbol{k}'})^2f'(\varepsilon^+_{\boldsymbol{k}})}{(1-i\omega\tau^+_{\boldsymbol{k}})(1-i\omega\tau^+_{\boldsymbol{k}'})}I^{sk,2}(\boldsymbol{k},\boldsymbol{k}',\boldsymbol{k}'')\tilde{v}^{++}_{a\boldsymbol{k}}\\
&\times\Big[\mathcal{W}^{++}_{cb\boldsymbol{k}'}\delta(\varepsilon^+_{\boldsymbol{k}} - \varepsilon^+_{\boldsymbol{k}''} + \hbar\omega) - \mathcal{W}^{++}_{bc\boldsymbol{k}'}\delta(\varepsilon^+_{\boldsymbol{k}} - \varepsilon^+_{\boldsymbol{k}''})\Big]\delta(\varepsilon^+_{\boldsymbol{k}} - \varepsilon^+_{\boldsymbol{k}'}) + b\leftrightarrow c,\end{aligned} \tag{129}$$

$$\mathcal{D}^{sk,2}_{(b)}(\omega) = \frac{ie^3\hbar}{2\pi}\int[dk]\int[dk']\int[dk'']\int_{-\infty}^{\infty}d\varepsilon f'(\varepsilon)\tilde{v}^{++}_{a\boldsymbol{k}}\langle V^{++}_{\boldsymbol{k}\boldsymbol{k}'}V^{++}_{\boldsymbol{k}'\boldsymbol{k}''}V^{++}_{\boldsymbol{k}''\boldsymbol{k}}\rangle$$
$$\times\Big[\mathcal{W}^{++}_{cb\boldsymbol{k}'}\tilde{G}^R_{+\boldsymbol{k}}(\varepsilon_1^+)\tilde{G}^R_{+\boldsymbol{k}'}(\varepsilon_1^+)\tilde{G}^R_{+\boldsymbol{k}'}(\varepsilon)\tilde{G}^A_{+\boldsymbol{k}'}(\varepsilon)\tilde{G}^A_{+\boldsymbol{k}''}(\varepsilon)\tilde{G}^A_{+\boldsymbol{k}}(\varepsilon)$$
$$+\mathcal{W}^{++}_{bc\boldsymbol{k}'}\tilde{G}^R_{+\boldsymbol{k}}(\varepsilon)\tilde{G}^R_{+\boldsymbol{k}'}(\varepsilon)\tilde{G}^A_{+\boldsymbol{k}'}(\varepsilon)\tilde{G}^A_{+\boldsymbol{k}'}(\varepsilon_1^-)\tilde{G}^A_{+\boldsymbol{k}''}(\varepsilon_1^-)\tilde{G}^A_{+\boldsymbol{k}}(\varepsilon_1^-)\Big] + b\leftrightarrow c$$
$$\simeq -\frac{2\pi^2e^3}{\hbar^2}\int[dk]\int[dk']\int[dk'']\frac{\tau^+_{\boldsymbol{k}}(\tau^+_{\boldsymbol{k}'})^2f'(\varepsilon^+_{\boldsymbol{k}})}{(1-i\omega\tau^+_{\boldsymbol{k}})(1-i\omega\tau^+_{\boldsymbol{k}'})}I^{sk,2}(\boldsymbol{k},\boldsymbol{k}',\boldsymbol{k}'')\tilde{v}^{++}_{a\boldsymbol{k}}$$
$$\Big[\mathcal{W}^{++}_{cb\boldsymbol{k}'}\delta(\varepsilon^+_{\boldsymbol{k}}-\varepsilon^+_{\boldsymbol{k}''}) - \mathcal{W}^{++}_{bc\boldsymbol{k}'}\delta(\varepsilon^+_{\boldsymbol{k}}-\varepsilon^+_{\boldsymbol{k}''}-\hbar\omega)\Big]\delta(\varepsilon^+_{\boldsymbol{k}}-\varepsilon^+_{\boldsymbol{k}'}) + b\leftrightarrow c, \tag{130}$$

$$\mathcal{D}^{sk,2}_{(c)}(\omega) = \frac{ie^3\hbar}{2\pi}\int[dk]\int[dk']\int[dk'']\int_{-\infty}^{\infty}d\varepsilon f'(\varepsilon)\langle V^{++}_{\boldsymbol{k}\boldsymbol{k}'}V^{++}_{\boldsymbol{k}'\boldsymbol{k}''}V^{++}_{\boldsymbol{k}''\boldsymbol{k}}\rangle$$
$$\times\Big[\tilde{v}^{++}_{a\boldsymbol{k}}v^{++}_{b\boldsymbol{k}'}\tilde{v}^{++}_{c\boldsymbol{k}}\tilde{G}^R_{+\boldsymbol{k}}(\varepsilon_1^+)\tilde{G}^R_{+\boldsymbol{k}'}(\varepsilon_1^+)\tilde{G}^R_{+\boldsymbol{k}'}(\varepsilon)\tilde{G}^R_{+\boldsymbol{k}''}(\varepsilon)\tilde{G}^R_{+\boldsymbol{k}}(\varepsilon)\tilde{G}^A_{+\boldsymbol{k}}(\varepsilon)$$
$$+\mathcal{W}_{ac\boldsymbol{k}}\tilde{v}^{++}_{b\boldsymbol{k}'}\tilde{G}^R_{+\boldsymbol{k}}(\varepsilon)\tilde{G}^R_{+\boldsymbol{k}'}(\varepsilon)\tilde{G}^A_{+\boldsymbol{k}'}(\varepsilon)\tilde{G}^A_{+\boldsymbol{k}''}(\varepsilon)\tilde{G}^A_{+\boldsymbol{k}}(\varepsilon)\tilde{G}^A_{+\boldsymbol{k}}(\varepsilon_1^-)\Big] + b\leftrightarrow c$$
$$\simeq -\frac{\pi^2e^3}{\hbar^2}\int[dk]\int[dk']\int[dk'']\frac{\tau^+_{\boldsymbol{k}'}(\tau^+_{\boldsymbol{k}})^2}{1-i\omega\tau^+_{\boldsymbol{k}}}f'(\varepsilon^+_{\boldsymbol{k}})I^{sk,2}(\boldsymbol{k},\boldsymbol{k}',\boldsymbol{k}'')$$
$$\times\Big[\frac{\tilde{v}^{++}_{a\boldsymbol{k}}v^{++}_{b\boldsymbol{k}'}\tilde{v}^{++}_{c\boldsymbol{k}}}{1-i\omega\tau^+_{\boldsymbol{k}'}} - 2\mathcal{W}_{ac\boldsymbol{k}}\tilde{v}^{++}_{b\boldsymbol{k}'}\Big]\delta(\varepsilon^+_{\boldsymbol{k}}-\varepsilon^+_{\boldsymbol{k}'})\delta(\varepsilon^+_{\boldsymbol{k}}-\varepsilon^+_{\boldsymbol{k}''}) + b\leftrightarrow c, \tag{131}$$

$$\mathcal{D}^{sk,2}_{(d)}(\omega) = \frac{ie^3\hbar}{2\pi}\int[dk]\int[dk']\int[dk'']\int_{-\infty}^{\infty}d\varepsilon f'(\varepsilon)\langle V^{++}_{\boldsymbol{k}\boldsymbol{k}''}V^{++}_{\boldsymbol{k}''\boldsymbol{k}'}V^{++}_{\boldsymbol{k}'\boldsymbol{k}}\rangle$$
$$\times\Big[\tilde{v}^{++}_{a\boldsymbol{k}}v^{++}_{b\boldsymbol{k}'}\tilde{v}^{++}_{c\boldsymbol{k}}\tilde{G}^R_{+\boldsymbol{k}}(\varepsilon_1^+)\tilde{G}^R_{+\boldsymbol{k}''}(\varepsilon_1^+)\tilde{G}^R_{+\boldsymbol{k}'}(\varepsilon_1^+)\tilde{G}^R_{+\boldsymbol{k}'}(\varepsilon)\tilde{G}^R_{+\boldsymbol{k}}(\varepsilon)\tilde{G}^A_{+\boldsymbol{k}}(\varepsilon)$$
$$+\mathcal{W}_{ac\boldsymbol{k}}\tilde{v}^{++}_{b\boldsymbol{k}'}\tilde{G}^R_{+\boldsymbol{k}}(\varepsilon)\tilde{G}^R_{+\boldsymbol{k}''}(\varepsilon)\tilde{G}^R_{+\boldsymbol{k}'}(\varepsilon)\tilde{G}^A_{+\boldsymbol{k}'}(\varepsilon)\tilde{G}^A_{+\boldsymbol{k}}(\varepsilon)\tilde{G}^A_{+\boldsymbol{k}}(\varepsilon_1^-)\Big] + b\leftrightarrow c$$
$$\simeq \frac{\pi^2e^3}{\hbar^2}\int[dk]\int[dk']\int[dk'']\frac{\tau^+_{\boldsymbol{k}'}(\tau^+_{\boldsymbol{k}})^2}{1-i\omega\tau^+_{\boldsymbol{k}}}f'(\varepsilon^+_{\boldsymbol{k}})I^{sk,2}(\boldsymbol{k},\boldsymbol{k}',\boldsymbol{k}'')$$
$$\times\Big[\frac{\tilde{v}^{++}_{a\boldsymbol{k}}v^{++}_{b\boldsymbol{k}'}\tilde{v}^{++}_{c\boldsymbol{k}}}{1-i\omega\tau^+_{\boldsymbol{k}'}}\delta(\varepsilon^+_{\boldsymbol{k}}-\varepsilon^+_{\boldsymbol{k}''}+\hbar\omega) + 2\mathcal{W}_{ac\boldsymbol{k}}\tilde{v}^{++}_{b\boldsymbol{k}'}\delta(\varepsilon^+_{\boldsymbol{k}}-\varepsilon^+_{\boldsymbol{k}''})\Big]\delta(\varepsilon^+_{\boldsymbol{k}}-\varepsilon^+_{\boldsymbol{k}'}) + b\leftrightarrow c, \tag{132}$$

$$\mathcal{D}^{sk,2}_{(e)}(\omega) = \frac{ie^3\hbar}{2\pi}\int[dk]\int[dk']\int[dk'']\int_{-\infty}^{\infty}d\varepsilon f'(\varepsilon)\langle V^{++}_{\boldsymbol{k}\boldsymbol{k}'}V^{++}_{\boldsymbol{k}'\boldsymbol{k}''}V^{++}_{\boldsymbol{k}''\boldsymbol{k}}\rangle$$
$$\times\Big[\mathcal{W}^{++}_{ab\boldsymbol{k}}\tilde{v}^{++}_{c\boldsymbol{k}'}\tilde{G}^R_{+\boldsymbol{k}}(\varepsilon_1^+)\tilde{G}^R_{+\boldsymbol{k}}(\varepsilon)\tilde{G}^R_{+\boldsymbol{k}'}(\varepsilon)\tilde{G}^A_{+\boldsymbol{k}'}(\varepsilon)\tilde{G}^A_{+\boldsymbol{k}''}(\varepsilon)\tilde{G}^A_{+\boldsymbol{k}}(\varepsilon)$$
$$+\tilde{v}^{++}_{a\boldsymbol{k}}\tilde{v}^{++}_{b\boldsymbol{k}}v^{++}_{c\boldsymbol{k}'}\tilde{G}^R_{+\boldsymbol{k}}(\varepsilon)\tilde{G}^A_{+\boldsymbol{k}}(\varepsilon)\tilde{G}^A_{+\boldsymbol{k}'}(\varepsilon)\tilde{G}^A_{+\boldsymbol{k}'}(\varepsilon_1^-)\tilde{G}^A_{+\boldsymbol{k}''}(\varepsilon_1^-)\tilde{G}^A_{+\boldsymbol{k}}(\varepsilon_1^-)\Big] + b\leftrightarrow c$$
$$\simeq -\frac{\pi^2e^3}{\hbar^2}\int[dk]\int[dk']\int[dk'']\frac{\tau^+_{\boldsymbol{k}'}(\tau^+_{\boldsymbol{k}})^2}{1-i\omega\tau^+_{\boldsymbol{k}}}f'(\varepsilon^+_{\boldsymbol{k}})I^{sk,2}(\boldsymbol{k},\boldsymbol{k}',\boldsymbol{k}'')$$
$$\times\Big[2\mathcal{W}^{++}_{ab\boldsymbol{k}}\tilde{v}^{++}_{c\boldsymbol{k}'}\delta(\varepsilon^+_{\boldsymbol{k}}-\varepsilon^+_{\boldsymbol{k}''}) + \frac{\tilde{v}^{++}_{a\boldsymbol{k}}\tilde{v}^{++}_{b\boldsymbol{k}}v^{++}_{c\boldsymbol{k}'}}{1-i\omega\tau^+_{\boldsymbol{k}'}}\delta(\varepsilon^+_{\boldsymbol{k}}-\varepsilon^+_{\boldsymbol{k}''}-\hbar\omega)\Big]\delta(\varepsilon^+_{\boldsymbol{k}}-\varepsilon^+_{\boldsymbol{k}'}) + b\leftrightarrow c, \tag{133}$$

$$\mathcal{D}^{sk,2}_{(f)}(\omega) = \frac{ie^3\hbar}{2\pi}\int[dk]\int[dk']\int[dk'']\int_{-\infty}^{\infty}d\varepsilon f'(\varepsilon)\langle V^{++}_{\boldsymbol{k}\boldsymbol{k}''}V^{++}_{\boldsymbol{k}''\boldsymbol{k}'}V^{++}_{\boldsymbol{k}'\boldsymbol{k}}\rangle$$
$$\times\Big[\mathcal{W}^{++}_{ab\boldsymbol{k}}\tilde{v}^{++}_{c\boldsymbol{k}'}\tilde{G}^R_{+\boldsymbol{k}}(\varepsilon_1^+)\tilde{G}^R_{+\boldsymbol{k}}(\varepsilon)\tilde{G}^R_{+\boldsymbol{k}''}(\varepsilon)\tilde{G}^R_{+\boldsymbol{k}'}(\varepsilon)\tilde{G}^A_{+\boldsymbol{k}'}(\varepsilon)\tilde{G}^A_{+\boldsymbol{k}}(\varepsilon)$$
$$+\tilde{v}^{++}_{a\boldsymbol{k}}\tilde{v}^{++}_{b\boldsymbol{k}}v^{++}_{c\boldsymbol{k}'}\tilde{G}^R_{+\boldsymbol{k}}(\varepsilon)\tilde{G}^A_{+\boldsymbol{k}}(\varepsilon)\tilde{G}^A_{+\boldsymbol{k}''}(\varepsilon)\tilde{G}^A_{+\boldsymbol{k}'}(\varepsilon)\tilde{G}^A_{+\boldsymbol{k}'}(\varepsilon_1^-)\tilde{G}^A_{+\boldsymbol{k}}(\varepsilon_1^-)\Big] + b\leftrightarrow c$$
$$\simeq -\frac{\pi^2e^3}{\hbar^2}\int[dk]\int[dk']\int[dk'']\frac{\tau^+_{\boldsymbol{k}'}(\tau^+_{\boldsymbol{k}})^2}{1-i\omega\tau^+_{\boldsymbol{k}}}f'(\varepsilon^+_{\boldsymbol{k}})I^{sk,2}(\boldsymbol{k},\boldsymbol{k}',\boldsymbol{k}'')$$
$$\times\Big[2\mathcal{W}^{++}_{ab\boldsymbol{k}}\tilde{v}^{++}_{c\boldsymbol{k}'} - \frac{\tilde{v}^{++}_{a\boldsymbol{k}}\tilde{v}^{++}_{b\boldsymbol{k}}v^{++}_{c\boldsymbol{k}'}}{1-i\omega\tau^+_{\boldsymbol{k}'}}\Big]\delta(\varepsilon^+_{\boldsymbol{k}}-\varepsilon^+_{\boldsymbol{k}'})\delta(\varepsilon^+_{\boldsymbol{k}}-\varepsilon^+_{\boldsymbol{k}''}) + b\leftrightarrow c, \tag{134}$$

$$\mathcal{D}^{sk,2}_{(g)}(\omega) = \frac{ie^3\hbar}{2\pi}\int[dk]\int[dk']\int[dk'']\int_{-\infty}^{\infty} d\varepsilon f'(\varepsilon)\tilde{v}^{++}_{a\boldsymbol{k}}\langle V^{++}_{\boldsymbol{k}\boldsymbol{k}'}V^{++}_{\boldsymbol{k}'\boldsymbol{k}''}V^{++}_{\boldsymbol{k}''\boldsymbol{k}}\rangle$$
$$\times\Big[v^{++}_{b\boldsymbol{k}'}\tilde{v}^{++}_{c\boldsymbol{k}''}\tilde{G}^R_{+\boldsymbol{k}}(\varepsilon_1^+)\tilde{G}^R_{+\boldsymbol{k}'}(\varepsilon_1^+)\tilde{G}^R_{+\boldsymbol{k}'}(\varepsilon)\tilde{G}^R_{+\boldsymbol{k}''}(\varepsilon)\tilde{G}^A_{+\boldsymbol{k}''}(\varepsilon)\tilde{G}^A_{+\boldsymbol{k}}(\varepsilon)$$
$$+\tilde{v}^{++}_{b\boldsymbol{k}'}v^{++}_{c\boldsymbol{k}''}\tilde{G}^R_{+\boldsymbol{k}}(\varepsilon)\tilde{G}^R_{+\boldsymbol{k}'}(\varepsilon)\tilde{G}^A_{+\boldsymbol{k}'}(\varepsilon)\tilde{G}^A_{+\boldsymbol{k}''}(\varepsilon)\tilde{G}^A_{+\boldsymbol{k}''}(\varepsilon_1^-)\tilde{G}^A_{+\boldsymbol{k}}(\varepsilon_1^-)\Big] + b\leftrightarrow c$$
$$\simeq \frac{2\pi^2e^3}{\hbar^2}\int[dk]\int[dk']\int[dk'']\frac{\tau^+_{\boldsymbol{k}}\tau^+_{\boldsymbol{k}'}\tau^+_{\boldsymbol{k}''}}{1-i\omega\tau^+_{\boldsymbol{k}}}f'(\varepsilon^+_{\boldsymbol{k}})I^{sk,2}(\boldsymbol{k},\boldsymbol{k}',\boldsymbol{k}'')\tilde{v}^{++}_{a\boldsymbol{k}}$$
$$\times\Big[\frac{v^{++}_{b\boldsymbol{k}'}\tilde{v}^{++}_{c\boldsymbol{k}''}}{1-i\omega\tau^+_{\boldsymbol{k}'}}+\frac{\tilde{v}^{++}_{b\boldsymbol{k}'}v^{++}_{c\boldsymbol{k}''}}{1-i\omega\tau^+_{\boldsymbol{k}''}}\Big]\delta(\varepsilon^+_{\boldsymbol{k}}-\varepsilon^+_{\boldsymbol{k}'})\delta(\varepsilon^+_{\boldsymbol{k}}-\varepsilon^+_{\boldsymbol{k}''}) + b\leftrightarrow c. \tag{135}$$

By combining all the relevant contributions in the above results, the extrinsic skew-scattering contribution can be found as

$$\Xi^{I\text{-}sk,2}_{abc} = \sum_{i=a}^{g}\frac{\partial\tilde{\mathcal{D}}^{sk,2}_{(i)}(\omega)}{\partial\omega}\Big|_{\omega=0}$$
$$= e^3\int[dk]\int[dk']\int[dk'']\tau^+_{\boldsymbol{k}}\tau^+_{\boldsymbol{k}'}f'(\varepsilon^+_{\boldsymbol{k}})\Big(\tau^+_{\boldsymbol{k}'}\tilde{v}^{++}_{a\boldsymbol{k}}\mathcal{W}^{++}_{bc\boldsymbol{k}'}-\frac{\tau^+_{\boldsymbol{k}}}{2}\tilde{v}^{++}_{a\boldsymbol{k}}\tilde{v}^{++}_{b\boldsymbol{k}}v^{++}_{c\boldsymbol{k}'}\Big)W^{sk,2}_{\boldsymbol{k}\boldsymbol{k}'\boldsymbol{k}''}\delta'(\varepsilon^+_{\boldsymbol{k}}-\varepsilon^+_{\boldsymbol{k}''}) + b\leftrightarrow c, \tag{136}$$

And the extrinsic skew-scattering contribution to χ^I_{abc} in the dc limit is

$$\chi^{I\text{-}sk,2}_{abc} = \frac{e^3}{4}\int[dk]\int[dk']\int[dk'']\tau^+_{\boldsymbol{k}}\tau^+_{\boldsymbol{k}'}f'(\varepsilon^+_{\boldsymbol{k}})\Big(\tau^+_{\boldsymbol{k}'}\tilde{v}^{++}_{a\boldsymbol{k}}\mathcal{W}^{++}_{bc\boldsymbol{k}'}-\frac{\tau^+_{\boldsymbol{k}}}{2}\tilde{v}^{++}_{a\boldsymbol{k}}\tilde{v}^{++}_{b\boldsymbol{k}}v^{++}_{c\boldsymbol{k}'}\Big)W^{sk,2}_{\boldsymbol{k}\boldsymbol{k}'\boldsymbol{k}''}\delta'(\varepsilon^+_{\boldsymbol{k}}-\varepsilon^+_{\boldsymbol{k}''}) + b\leftrightarrow c. \tag{137}$$

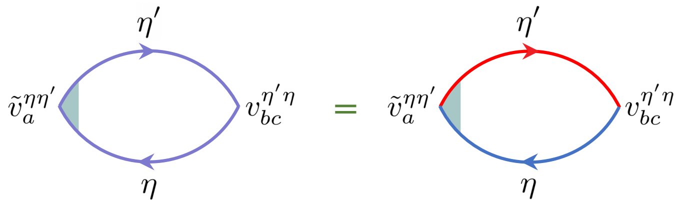

FIG. 10. Effective diagrammatic interpretation of the two-photon diagram. The purple lines represent the Matsubara Green's function. The red and blue lines represent the retarded and advanced Green's functions, respectively.

Supplementary Note 4. DIAGRAMMATICS: III. TWO-PHOTON DIAGRAMS

In this section, we turn to the calculation of the disorder averaged Fermi surface contribution Ξ^{II}_{abc} to the nonlinear Hall effect, which corresponds to the diagrams with two-photon vertex. According to the analysis in Sec. Supplementary Note 2 C, the effective diagrammatic interpretation of the two-photon diagrams are plotted in Supplementary Fig. 10, which effectively connects the original two-photon diagrams with Matsubara Green's functions to the effective diagrams with retarded (advanced) Green's functions. Different from the case in triangular diagrams, in the two-photon diagrams the ω-dependent part of the vertex correction has finite contribution. Following the discussion in Sec. Supplementary Note 3, we denote the ω-dependent modified vertex as $\tilde{v}^{\eta\eta}_a(\omega)$, and its leading contributions under ω-expansion read $\tilde{v}^{\eta\eta}_a(\omega)\simeq\tilde{v}^{\eta\eta}_a+i\omega\tau\delta\tilde{v}^{\eta\eta}_a$.

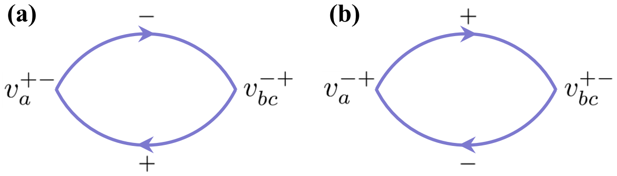

FIG. 11. Feynman diagrams of the two-photon intrinsic contribution.

A. Intrinsic contribution

The intrinsic two-photon diagrams are disorder-independent as shown in Supplementary Fig. 11. By denoting the diagrams in Supplementary Fig. 11 (a)-(b) as $\tilde{\mathcal{D}}^{in}_{(a)}(\omega)$-$\tilde{\mathcal{D}}^{in}_{(b)}(\omega)$, we have

$$\begin{aligned}\tilde{\mathcal{D}}^{in}_{(a)}(\omega) = &\frac{ie^3\hbar}{4\pi}\int[dk]\int_{-\infty}^{\infty}d\varepsilon f'(\varepsilon)v^{+-}_{a\boldsymbol{k}}v^{-+}_{bc\boldsymbol{k}}\Big[\tilde{G}^R_{-\boldsymbol{k}}(\varepsilon+\hbar\omega)\tilde{G}^A_{+\boldsymbol{k}}(\varepsilon)\\ &+\tilde{G}^R_{-\boldsymbol{k}}(\varepsilon)\tilde{G}^A_{+\boldsymbol{k}}(\varepsilon-\hbar\omega)\Big]+b\leftrightarrow c\\ \sim&\ (n_iV_0^2)^0,\end{aligned} \tag{138}$$

$$\begin{aligned}\tilde{\mathcal{D}}^{in}_{(b)}(\omega) = &\frac{ie^3\hbar}{4\pi}\int[dk]\int_{-\infty}^{\infty}d\varepsilon f'(\varepsilon)v^{-+}_{a\boldsymbol{k}}v^{+-}_{bc\boldsymbol{k}}\Big[\tilde{G}^R_{+\boldsymbol{k}}(\varepsilon+\hbar\omega)\tilde{G}^A_{-\boldsymbol{k}}(\varepsilon)\\ &+\tilde{G}^R_{+\boldsymbol{k}}(\varepsilon)\tilde{G}^A_{-\boldsymbol{k}}(\varepsilon-\hbar\omega)\Big]+b\leftrightarrow c\\ \sim&\ (n_iV_0^2)^0,\end{aligned} \tag{139}$$

This contribution is not of leading order in $n_iV_0^2$ because all the diagrams are disorder-independent. Thus, up to the leading order, we have $\Xi^{II\text{-}in}_{abc}=0$.

B. Side-jump contribution

Similar with triangular diagrams, the two-photon side-jump diagrams can also be classified into two categories: the anomalous velocity and anomalous distribution induced side-jump diagrams.

1. *Anomalous velocity induced side-jump contribution*

The diagrams corresponding to the anomalous velocity induced side-jump contribution with two-photon vertex are shown in Supplementary Figs. 12. By denoting the diagrams in Supplementary Fig. 12 (a)-(d) as $\tilde{\mathcal{D}}^{sj,1}_{(a)}(\omega)$-$\tilde{\mathcal{D}}^{sj,1}_{(d)}(\omega)$,

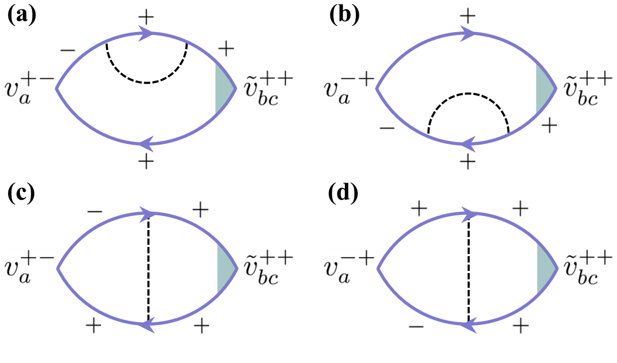

FIG. 12. Feynman diagrams of the two-photon anomalous velocity induced side-jump contribution.

we have

$$\tilde{\mathcal{D}}^{sj,1}_{(a)}(\omega) = \frac{ie^3\hbar}{4\pi}\int[dk]\int[dk']\int_{-\infty}^{\infty} d\varepsilon f'(\varepsilon) v^{+-}_{a\boldsymbol{k}}\tilde{v}^{++}_{bc\boldsymbol{k}}(\omega)\langle V^{-+}_{\boldsymbol{k}\boldsymbol{k}'}V^{++}_{\boldsymbol{k}'\boldsymbol{k}}\rangle_{dis}\Big[\tilde{G}^R_{-\boldsymbol{k}}(\varepsilon_1^+)\tilde{G}^R_{+\boldsymbol{k}'}(\varepsilon_1^+)\tilde{G}^R_{+\boldsymbol{k}}(\varepsilon_1^+)\tilde{G}^A_{+\boldsymbol{k}}(\varepsilon)$$
$$+\tilde{G}^R_{-\boldsymbol{k}}(\varepsilon)\tilde{G}^R_{+\boldsymbol{k}'}(\varepsilon)\tilde{G}^R_{+\boldsymbol{k}}(\varepsilon)\tilde{G}^A_{+\boldsymbol{k}}(\varepsilon_1^-)\Big]$$
$$\simeq \frac{i\pi e^3}{2}\int[dk]\int[dk']\frac{\tau^+_{\boldsymbol{k}}}{1-i\omega\tau^+_{\boldsymbol{k}}}f'(\varepsilon^+_{\boldsymbol{k}})I^{sj}_a(\boldsymbol{k},\boldsymbol{k}')\Big[\frac{\delta(\varepsilon^+_{\boldsymbol{k}}-\varepsilon^+_{\boldsymbol{k}'}+\hbar\omega)}{2h_{\boldsymbol{k}}+\hbar\omega}$$
$$+\frac{\delta(\varepsilon^+_{\boldsymbol{k}}-\varepsilon^+_{\boldsymbol{k}'})}{2h_{\boldsymbol{k}}}\Big]\tilde{v}^{++}_{bc\boldsymbol{k}}(\omega) + b\leftrightarrow c, \tag{140}$$

$$\tilde{\mathcal{D}}^{sj,1}_{(b)}(\omega) = \frac{ie^3\hbar}{4\pi}\int[dk]\int[dk']\int_{-\infty}^{\infty} d\varepsilon f'(\varepsilon) v^{-+}_{a\boldsymbol{k}}\tilde{v}^{++}_{bc\boldsymbol{k}}(\omega)\langle V^{++}_{\boldsymbol{k}\boldsymbol{k}'}V^{+-}_{\boldsymbol{k}'\boldsymbol{k}}\rangle_{dis}\Big[\tilde{G}^R_{+\boldsymbol{k}}(\varepsilon_1^+)\tilde{G}^A_{+\boldsymbol{k}}(\varepsilon)\tilde{G}^A_{+\boldsymbol{k}'}(\varepsilon)\tilde{G}^A_{-\boldsymbol{k}}(\varepsilon)$$
$$+\tilde{G}^R_{+\boldsymbol{k}}(\varepsilon)\tilde{G}^A_{+\boldsymbol{k}}(\varepsilon_1^-)\tilde{G}^A_{+\boldsymbol{k}'}(\varepsilon_1^-)\tilde{G}^A_{-\boldsymbol{k}}(\varepsilon_1^-)\Big]$$
$$\simeq \frac{i\pi e^3}{2}\int[dk]\int[dk']\frac{\tau^+_{\boldsymbol{k}}}{1-i\omega\tau^+_{\boldsymbol{k}}}f'(\varepsilon^+_{\boldsymbol{k}})I^{sj}_a(\boldsymbol{k},\boldsymbol{k}')\Big[\frac{\delta(\varepsilon^+_{\boldsymbol{k}}-\varepsilon^+_{\boldsymbol{k}'})}{2h_{\boldsymbol{k}}}$$
$$+\frac{\delta(\varepsilon^+_{\boldsymbol{k}}-\varepsilon^+_{\boldsymbol{k}'}-\hbar\omega)}{2h_{\boldsymbol{k}}-\hbar\omega}\Big]\tilde{v}^{++}_{bc\boldsymbol{k}}(\omega) + b\leftrightarrow c, \tag{141}$$

$$\tilde{\mathcal{D}}^{sj,1}_{(c)}(\omega) = \frac{ie^3\hbar}{4\pi}\int[dk]\int[dk']\int_{-\infty}^{\infty} d\varepsilon f'(\varepsilon) v^{+-}_{a\boldsymbol{k}'}\tilde{v}^{++}_{bc\boldsymbol{k}}(\omega)\langle V^{-+}_{\boldsymbol{k}'\boldsymbol{k}}V^{++}_{\boldsymbol{k}\boldsymbol{k}'}\rangle_{dis}\Big[\tilde{G}^R_{-\boldsymbol{k}'}(\varepsilon_1^+)\tilde{G}^R_{+\boldsymbol{k}}(\varepsilon_1^+)\tilde{G}^A_{+\boldsymbol{k}}(\varepsilon)\tilde{G}^A_{+\boldsymbol{k}'}(\varepsilon)$$
$$+\tilde{G}^R_{-\boldsymbol{k}'}(\varepsilon)\tilde{G}^R_{+\boldsymbol{k}}(\varepsilon)\tilde{G}^A_{+\boldsymbol{k}}(\varepsilon_1^-)\tilde{G}^A_{+\boldsymbol{k}'}(\varepsilon_1^-)\Big]$$
$$\simeq -\frac{i\pi e^3}{2}\int[dk]\int[dk']\frac{\tau^+_{\boldsymbol{k}}}{1-i\omega\tau^+_{\boldsymbol{k}}}f'(\varepsilon^+_{\boldsymbol{k}})I^{sj}_a(\boldsymbol{k}',\boldsymbol{k})\Big[\frac{\delta(\varepsilon^+_{\boldsymbol{k}}-\varepsilon^+_{\boldsymbol{k}'})}{2h_{\boldsymbol{k}'}+\hbar\omega}$$
$$+\frac{\delta(\varepsilon^+_{\boldsymbol{k}}-\varepsilon^+_{\boldsymbol{k}'}-\hbar\omega)}{2h_{\boldsymbol{k}'}}\Big]\tilde{v}^{++}_{bc\boldsymbol{k}}(\omega) + b\leftrightarrow c, \tag{142}$$

$$\tilde{\mathcal{D}}^{sj,1}_{(d)}(\omega) = \frac{ie^3\hbar}{4\pi}\int[dk]\int[dk']\int_{-\infty}^{\infty} d\varepsilon f'(\varepsilon) v^{-+}_{a\boldsymbol{k}'}\tilde{v}^{++}_{bc\boldsymbol{k}}(\omega)\langle V^{++}_{\boldsymbol{k}'\boldsymbol{k}}V^{+-}_{\boldsymbol{k}\boldsymbol{k}'}\rangle_{dis}\Big[\tilde{G}^R_{+\boldsymbol{k}'}(\varepsilon_1^+)\tilde{G}^R_{+\boldsymbol{k}}(\varepsilon_1^+)\tilde{G}^A_{+\boldsymbol{k}}(\varepsilon)\tilde{G}^A_{-\boldsymbol{k}'}(\varepsilon)$$
$$+\tilde{G}^R_{+\boldsymbol{k}'}(\varepsilon)\tilde{G}^R_{+\boldsymbol{k}}(\varepsilon)\tilde{G}^A_{+\boldsymbol{k}}(\varepsilon_1^-)\tilde{G}^A_{-\boldsymbol{k}'}(\varepsilon_1^-)\Big]$$
$$\simeq -\frac{i\pi e^3}{2}\int[dk]\int[dk']\frac{\tau^+_{\boldsymbol{k}}}{1-i\omega\tau^+_{\boldsymbol{k}}}f'(\varepsilon^+_{\boldsymbol{k}})I^{sj}_a(\boldsymbol{k}',\boldsymbol{k})\Big[\frac{\delta(\varepsilon^+_{\boldsymbol{k}}-\varepsilon^+_{\boldsymbol{k}'}+\hbar\omega)}{2h_{\boldsymbol{k}'}}$$
$$+\frac{\delta(\varepsilon^+_{\boldsymbol{k}}-\varepsilon^+_{\boldsymbol{k}'})}{2h_{\boldsymbol{k}'}-\hbar\omega}\Big]\tilde{v}^{++}_{bc\boldsymbol{k}}(\omega) + b\leftrightarrow c. \tag{143}$$

In the dc limit, the anomalous velocity induced side-jump contribution up to the leading order in $n_iV_0^2$ can be obtained as

$$\Xi^{II\text{-}sj,1}_{abc} = \sum_{i=a}^{d}\frac{\partial\tilde{\mathcal{D}}^{sj,1}_{(i)}(\omega)}{\partial\omega}\Big|_{\omega=0}$$
$$= -e^3\int[dk]\int[dk](\tau^+_{\boldsymbol{k}})^2 v^{sj}_{a\boldsymbol{k}}(\tilde{v}^{++}_{bc\boldsymbol{k}}+\delta\tilde{v}^{++}_{bc\boldsymbol{k}})f'(\varepsilon^+_{\boldsymbol{k}}) + b\leftrightarrow c, \tag{144}$$

And the anomalous velocity induced side-jump contribution to χ^{II}_{abc} in the dc limit is

$$\chi^{II\text{-}sj,1}_{abc} = -\frac{e^3}{4}\int[dk]\int[dk](\tau^+_{\boldsymbol{k}})^2 v^{sj}_{a\boldsymbol{k}}(\tilde{v}^{++}_{bc\boldsymbol{k}}+\delta\tilde{v}^{++}_{bc\boldsymbol{k}})f'(\varepsilon^+_{\boldsymbol{k}}) + b\leftrightarrow c. \tag{145}$$

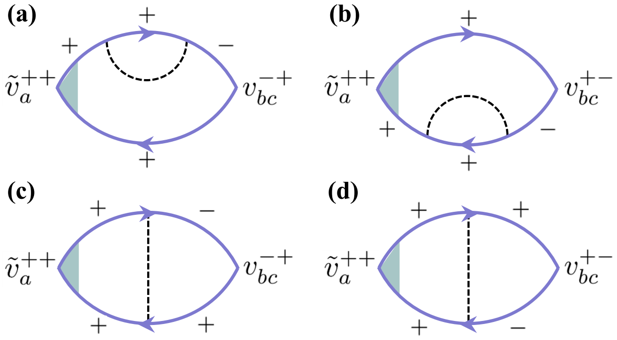

FIG. 13. Feynman diagrams of the two-photon anomalous distribution induced side-jump contribution.

2. Anomalous distribution induced side-jump contribution

The diagrams corresponding to the anomalous distribution induced side-jump contribution with two-photon vertex are shown in Supplementary Figs. 13. By denoting the diagrams in Supplementary Fig. 13 (a)-(d) as $\tilde{\mathcal{D}}^{sj,2}_{(a)}(\omega)$-$\tilde{\mathcal{D}}^{sj,2}_{(d)}(\omega)$, we have

$$\begin{aligned}\tilde{\mathcal{D}}^{sj,2}_{(a)}(\omega) =& \frac{ie^3\hbar}{4\pi}\int[dk]\int[dk']\int_{-\infty}^{\infty} d\varepsilon f'(\varepsilon)\tilde{v}^{++}_{a\boldsymbol{k}}(\omega)v^{-+}_{bc\boldsymbol{k}}\langle V^{++}_{\boldsymbol{k}\boldsymbol{k}'}V^{+-}_{\boldsymbol{k}'\boldsymbol{k}}\rangle_{dis}\Big[\tilde{G}^R_{+\boldsymbol{k}}(\varepsilon_1^+)\tilde{G}^R_{+\boldsymbol{k}'}(\varepsilon_1^+)\tilde{G}^R_{-\boldsymbol{k}}(\varepsilon_1^+)\tilde{G}^A_{+\boldsymbol{k}}(\varepsilon)\\ &+\tilde{G}^R_{+\boldsymbol{k}}(\varepsilon)\tilde{G}^R_{+\boldsymbol{k}'}(\varepsilon)\tilde{G}^R_{-\boldsymbol{k}}(\varepsilon)\tilde{G}^A_{+\boldsymbol{k}}(\varepsilon_1^-)\Big]\\ \simeq& -\frac{i\pi e^3}{2}\int[dk]\int[dk']\frac{\tau^+_{\boldsymbol{k}}}{1-i\omega\tau^+_{\boldsymbol{k}}}f'(\varepsilon^+_{\boldsymbol{k}})\tilde{v}^{++}_{a\boldsymbol{k}}(\omega)I^{sj}_{bc}(\boldsymbol{k},\boldsymbol{k}')\Big[\frac{\delta(\varepsilon^+_{\boldsymbol{k}}-\varepsilon^+_{\boldsymbol{k}'}+\hbar\omega)}{2h_{\boldsymbol{k}}+\hbar\omega}\\ &+\frac{\delta(\varepsilon^+_{\boldsymbol{k}}-\varepsilon^+_{\boldsymbol{k}'})}{2h_{\boldsymbol{k}}}\Big]+b\leftrightarrow c,\end{aligned} \tag{146}$$

$$\begin{aligned}\tilde{\mathcal{D}}^{sj,2}_{(b)}(\omega) =& \frac{ie^3\hbar}{4\pi}\int[dk]\int[dk']\int_{-\infty}^{\infty} d\varepsilon f'(\varepsilon)\tilde{v}^{++}_{a\boldsymbol{k}}(\omega)v^{+-}_{bc\boldsymbol{k}}\langle V^{-+}_{\boldsymbol{k}\boldsymbol{k}'}V^{++}_{\boldsymbol{k}'\boldsymbol{k}}\rangle_{dis}\Big[\tilde{G}^R_{+\boldsymbol{k}}(\varepsilon_1^+)\tilde{G}^A_{-\boldsymbol{k}}(\varepsilon)\tilde{G}^A_{+\boldsymbol{k}'}(\varepsilon)\tilde{G}^A_{+\boldsymbol{k}}(\varepsilon)\\ &+\tilde{G}^R_{+\boldsymbol{k}}(\varepsilon)\tilde{G}^A_{-\boldsymbol{k}}(\varepsilon_1^-)\tilde{G}^A_{+\boldsymbol{k}'}(\varepsilon_1^-)\tilde{G}^A_{+\boldsymbol{k}}(\varepsilon_1^-)\Big]\\ \simeq& -\frac{i\pi e^3}{2}\int[dk]\int[dk']\frac{\tau^+_{\boldsymbol{k}}}{1-i\omega\tau^+_{\boldsymbol{k}}}f'(\varepsilon^+_{\boldsymbol{k}})\tilde{v}^{++}_{a\boldsymbol{k}}(\omega)I^{sj}_{bc}(\boldsymbol{k},\boldsymbol{k}')\Big[\frac{\delta(\varepsilon^+_{\boldsymbol{k}}-\varepsilon^+_{\boldsymbol{k}'})}{2h_{\boldsymbol{k}}}\\ &+\frac{\delta(\varepsilon^+_{\boldsymbol{k}}-\varepsilon^+_{\boldsymbol{k}'}-\hbar\omega)}{2h_{\boldsymbol{k}}-\hbar\omega}\Big]+b\leftrightarrow c,\end{aligned} \tag{147}$$

$$\begin{aligned}\tilde{\mathcal{D}}^{sj,2}_{(c)}(\omega) =& \frac{ie^3\hbar}{4\pi}\int[dk]\int[dk']\int_{-\infty}^{\infty} d\varepsilon f'(\varepsilon)\tilde{v}^{++}_{a\boldsymbol{k}}(\omega)v^{-+}_{bc\boldsymbol{k}'}\langle V^{+-}_{\boldsymbol{k}\boldsymbol{k}'}V^{++}_{\boldsymbol{k}'\boldsymbol{k}}\rangle_{dis}\Big[\tilde{G}^R_{+\boldsymbol{k}}(\varepsilon_1^+)\tilde{G}^R_{-\boldsymbol{k}'}(\varepsilon_1^+)\tilde{G}^A_{+\boldsymbol{k}'}(\varepsilon)\tilde{G}^A_{+\boldsymbol{k}}(\varepsilon)\\ &+\tilde{G}^R_{+\boldsymbol{k}}(\varepsilon)\tilde{G}^R_{-\boldsymbol{k}'}(\varepsilon)\tilde{G}^A_{+\boldsymbol{k}'}(\varepsilon_1^-)\tilde{G}^A_{+\boldsymbol{k}}(\varepsilon_1^-)\Big]\\ \simeq& \frac{i\pi e^3}{2}\int[dk]\int[dk']\frac{\tau^+_{\boldsymbol{k}}}{1-i\omega\tau^+_{\boldsymbol{k}}}f'(\varepsilon^+_{\boldsymbol{k}})\tilde{v}^{++}_{a\boldsymbol{k}}(\omega)I^{sj}_{bc}(\boldsymbol{k}',\boldsymbol{k})\Big[\frac{\delta(\varepsilon^+_{\boldsymbol{k}}-\varepsilon^+_{\boldsymbol{k}'})}{2h_{\boldsymbol{k}'}+\hbar\omega}\\ &+\frac{\delta(\varepsilon^+_{\boldsymbol{k}}-\varepsilon^+_{\boldsymbol{k}'}-\hbar\omega)}{2h_{\boldsymbol{k}'}}\Big]+b\leftrightarrow c,\end{aligned} \tag{148}$$

$$\tilde{\mathcal{D}}^{sj,2}_{(d)}(\omega)=\frac{ie^3\hbar}{4\pi}\int[dk]\int[dk']\int_{-\infty}^{\infty}d\varepsilon f'(\varepsilon)\tilde{v}^{++}_{a\boldsymbol{k}}(\omega)v^{+-}_{bc\boldsymbol{k}'}\langle V^{++}_{\boldsymbol{k}\boldsymbol{k}'}V^{-+}_{\boldsymbol{k}'\boldsymbol{k}}\rangle_{dis}\Big[\tilde{G}^R_{+\boldsymbol{k}}(\varepsilon_1^+)\tilde{G}^R_{+\boldsymbol{k}'}(\varepsilon_1^+)\tilde{G}^A_{-\boldsymbol{k}'}(\varepsilon)\tilde{G}^A_{+\boldsymbol{k}}(\varepsilon)$$
$$+\tilde{G}^R_{+\boldsymbol{k}}(\varepsilon)\tilde{G}^R_{+\boldsymbol{k}'}(\varepsilon)\tilde{G}^A_{-\boldsymbol{k}'}(\varepsilon_1^-)\tilde{G}^A_{+\boldsymbol{k}}(\varepsilon_1^-)\Big]$$
$$\simeq\frac{i\pi e^3}{2}\int[dk]\int[dk']\frac{\tau^+_{\boldsymbol{k}}}{1-i\omega\tau^+_{\boldsymbol{k}}}f'(\varepsilon^+_{\boldsymbol{k}})\tilde{v}^{++}_{a\boldsymbol{k}}(\omega)I^{sj}_{bc}(\boldsymbol{k}',\boldsymbol{k})\Big[\frac{\delta(\varepsilon^+_{\boldsymbol{k}}-\varepsilon^+_{\boldsymbol{k}'}+\hbar\omega)}{2h_{\boldsymbol{k}'}}$$
$$+\frac{\delta(\varepsilon^+_{\boldsymbol{k}}-\varepsilon^+_{\boldsymbol{k}'})}{2h_{\boldsymbol{k}'}-\hbar\omega}\Big]+b\leftrightarrow c. \tag{149}$$

In the dc limit, the anomalous distribution induced side-jump contribution up to the leading order in $n_iV_0^2$ can be obtained as

$$\Xi^{II\text{-}sj,2}_{abc}=\sum_{i=a}^{d}\frac{\partial\tilde{\mathcal{D}}^{sj,2}_{(i)}(\omega)}{\partial\omega}\Big|_{\omega=0}$$
$$=e^3\int[dk]\int[dk](\tau^+_{\boldsymbol{k}})^2(\tilde{v}^{++}_{a\boldsymbol{k}}+\delta\tilde{v}^{++}_{a\boldsymbol{k}})v^{sj}_{bc\boldsymbol{k}}f'(\varepsilon^+_{\boldsymbol{k}})+b\leftrightarrow c, \tag{150}$$

And the anomalous distribution induced side-jump contribution to χ^{II}_{abc} in the dc limit is

$$\chi^{II\text{-}sj,2}_{abc}=\frac{e^3}{4}\int[dk]\int[dk](\tau^+_{\boldsymbol{k}})^2(\tilde{v}^{++}_{a\boldsymbol{k}}+\delta\tilde{v}^{++}_{a\boldsymbol{k}})v^{sj}_{bc\boldsymbol{k}}f'(\varepsilon^+_{\boldsymbol{k}})+b\leftrightarrow c. \tag{151}$$

C. Skew-scattering contribution

Similar with triangular diagrams, the two-photon skew-scattering diagrams can also be classified into two categories: the intrinsic and extrinsic skew-scattering diagrams.

1. *Intrinsic skew-scattering contribution*

The diagrams corresponding to the intrinsic skew-scattering contribution with two-photon vertex are shown in Supplementary Figs. 14. By denoting the diagrams in Supplementary Fig. 14 (a)-(f) as $\tilde{\mathcal{D}}^{sk,1}_{(a)}(\omega)$-$\tilde{\mathcal{D}}^{sk,1}_{(f)}(\omega)$, we have

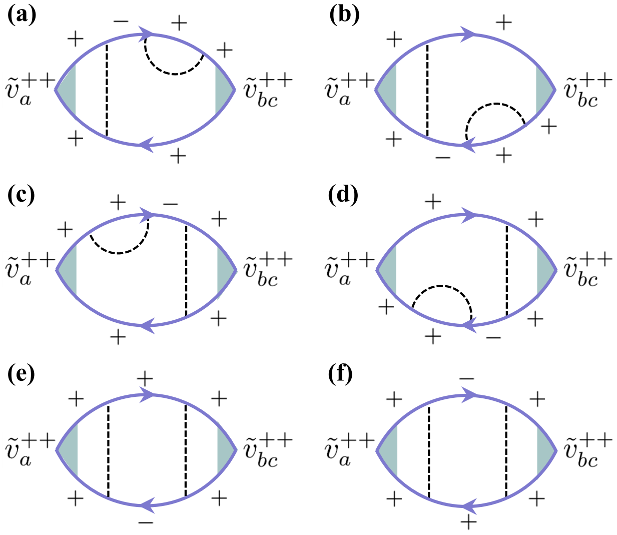

FIG. 14. Feynman diagrams of the two-photon intrinsic skew-scattering contribution.

$$\begin{aligned}
\tilde{\mathcal{D}}^{sk,1}_{(a)}(\omega) =& \frac{ie^3\hbar}{4\pi}\int[dk]\int[dk']\int[dk'']\int_{-\infty}^{\infty}d\varepsilon f'(\varepsilon)\tilde{v}^{++}_{a\boldsymbol{k}}(\omega)\tilde{v}^{++}_{bc\boldsymbol{k}'}(\omega)\langle V^{+-}_{\boldsymbol{k}\boldsymbol{k}'}V^{++}_{\boldsymbol{k}'\boldsymbol{k}}\rangle_{dis}\langle V^{-+}_{\boldsymbol{k}'\boldsymbol{k}''}V^{++}_{\boldsymbol{k}''\boldsymbol{k}'}\rangle_{dis}\\
&\times\Big[\tilde{G}^R_{+\boldsymbol{k}}(\varepsilon_1^+)\tilde{G}^R_{-\boldsymbol{k}'}(\varepsilon_1^+)\tilde{G}^R_{+\boldsymbol{k}''}(\varepsilon_1^+)\tilde{G}^R_{+\boldsymbol{k}'}(\varepsilon_1^+)\tilde{G}^A_{+\boldsymbol{k}'}(\varepsilon)\tilde{G}^A_{+\boldsymbol{k}}(\varepsilon)\\
&+\tilde{G}^R_{+\boldsymbol{k}}(\varepsilon)\tilde{G}^R_{-\boldsymbol{k}'}(\varepsilon)\tilde{G}^R_{+\boldsymbol{k}''}(\varepsilon)\tilde{G}^R_{+\boldsymbol{k}'}(\varepsilon)\tilde{G}^A_{+\boldsymbol{k}'}(\varepsilon_1^-)\tilde{G}^A_{+\boldsymbol{k}}(\varepsilon_1^-)\Big]\\
\simeq& \frac{i\pi^2e^3}{\hbar}\int[dk]\int[dk']\int[dk'']\frac{\tau^+_{\boldsymbol{k}}\tau^+_{\boldsymbol{k}'}f'(\varepsilon^+_{\boldsymbol{k}})}{(1-i\omega\tau^+_{\boldsymbol{k}})(1-i\omega\tau^+_{\boldsymbol{k}'})}\tilde{v}^{++}_{a\boldsymbol{k}}(\omega)\tilde{v}^{++}_{bc\boldsymbol{k}'}(\omega)I^{sk,1}(\boldsymbol{k}',\boldsymbol{k}'',\boldsymbol{k})\\
&\times\Big[\frac{\delta(\varepsilon^+_{\boldsymbol{k}}-\varepsilon^+_{\boldsymbol{k}''}+\hbar\omega)}{2h_{\boldsymbol{k}'}+\hbar\omega}+\frac{\delta(\varepsilon^+_{\boldsymbol{k}}-\varepsilon^+_{\boldsymbol{k}''})}{2h_{\boldsymbol{k}'}}\Big]\delta(\varepsilon^+_{\boldsymbol{k}}-\varepsilon^+_{\boldsymbol{k}'})+b\leftrightarrow c,
\end{aligned}\tag{152}$$

$$\begin{aligned}
\tilde{\mathcal{D}}^{sk,1}_{(b)}(\omega) =& \frac{ie^3\hbar}{4\pi}\int[dk]\int[dk']\int[dk'']\int_{-\infty}^{\infty}d\varepsilon f'(\varepsilon)\tilde{v}^{++}_{a\boldsymbol{k}}(\omega)\tilde{v}^{++}_{bc\boldsymbol{k}'}(\omega)\langle V^{++}_{\boldsymbol{k}\boldsymbol{k}'}V^{-+}_{\boldsymbol{k}'\boldsymbol{k}}\rangle_{dis}\langle V^{++}_{\boldsymbol{k}'\boldsymbol{k}''}V^{+-}_{\boldsymbol{k}''\boldsymbol{k}'}\rangle_{dis}\\
&\times\Big[\tilde{G}^R_{+\boldsymbol{k}}(\varepsilon_1^+)\tilde{G}^R_{+\boldsymbol{k}'}(\varepsilon_1^+)\tilde{G}^A_{+\boldsymbol{k}'}(\varepsilon)\tilde{G}^A_{+\boldsymbol{k}''}(\varepsilon)\tilde{G}^A_{-\boldsymbol{k}'}(\varepsilon)\tilde{G}^A_{+\boldsymbol{k}}(\varepsilon)\\
&+\tilde{G}^R_{+\boldsymbol{k}}(\varepsilon)\tilde{G}^R_{+\boldsymbol{k}'}(\varepsilon)\tilde{G}^A_{+\boldsymbol{k}'}(\varepsilon_1^-)\tilde{G}^A_{+\boldsymbol{k}''}(\varepsilon_1^-)\tilde{G}^A_{-\boldsymbol{k}'}(\varepsilon_1^-)\tilde{G}^A_{+\boldsymbol{k}}(\varepsilon_1^-)\Big]\\
\simeq& \frac{i\pi^2e^3}{\hbar}\int[dk]\int[dk']\int[dk'']\frac{\tau^+_{\boldsymbol{k}}\tau^+_{\boldsymbol{k}'}f'(\varepsilon^+_{\boldsymbol{k}})}{(1-i\omega\tau^+_{\boldsymbol{k}})(1-i\omega\tau^+_{\boldsymbol{k}'})}\tilde{v}^{++}_{a\boldsymbol{k}}(\omega)\tilde{v}^{++}_{bc\boldsymbol{k}'}(\omega)I^{sk,1}(\boldsymbol{k}',\boldsymbol{k}'',\boldsymbol{k})\\
&\times\Big[\frac{\delta(\varepsilon^+_{\boldsymbol{k}}-\varepsilon^+_{\boldsymbol{k}''})}{2h_{\boldsymbol{k}'}}+\frac{\delta(\varepsilon^+_{\boldsymbol{k}}-\varepsilon^+_{\boldsymbol{k}''}-\hbar\omega)}{2h_{\boldsymbol{k}'}-\hbar\omega}\Big]\delta(\varepsilon^+_{\boldsymbol{k}}-\varepsilon^+_{\boldsymbol{k}'})+b\leftrightarrow c,
\end{aligned}\tag{153}$$

$$\begin{aligned}
\tilde{\mathcal{D}}^{sk,1}_{(c)}(\omega) =& \frac{ie^3\hbar}{4\pi}\int[dk]\int[dk']\int[dk'']\int_{-\infty}^{\infty}d\varepsilon f'(\varepsilon)\tilde{v}^{++}_{a\boldsymbol{k}}(\omega)\tilde{v}^{++}_{bc\boldsymbol{k}'}(\omega)\langle V^{++}_{\boldsymbol{k}\boldsymbol{k}''}V^{+-}_{\boldsymbol{k}''\boldsymbol{k}}\rangle_{dis}\langle V^{-+}_{\boldsymbol{k}\boldsymbol{k}'}V^{++}_{\boldsymbol{k}'\boldsymbol{k}}\rangle_{dis}\\
&\times\Big[\tilde{G}^R_{+\boldsymbol{k}}(\varepsilon_1^+)\tilde{G}^R_{+\boldsymbol{k}''}(\varepsilon_1^+)\tilde{G}^R_{-\boldsymbol{k}}(\varepsilon_1^+)\tilde{G}^R_{+\boldsymbol{k}'}(\varepsilon_1^+)\tilde{G}^A_{+\boldsymbol{k}'}(\varepsilon)\tilde{G}^A_{+\boldsymbol{k}}(\varepsilon)\\
&+\tilde{G}^R_{+\boldsymbol{k}}(\varepsilon)\tilde{G}^R_{+\boldsymbol{k}''}(\varepsilon)\tilde{G}^R_{-\boldsymbol{k}}(\varepsilon)\tilde{G}^R_{+\boldsymbol{k}'}(\varepsilon)\tilde{G}^A_{+\boldsymbol{k}'}(\varepsilon_1^-)\tilde{G}^A_{+\boldsymbol{k}}(\varepsilon_1^-)\Big]\\
\simeq& \frac{i\pi^2e^3}{\hbar}\int[dk]\int[dk']\int[dk'']\frac{\tau^+_{\boldsymbol{k}}\tau^+_{\boldsymbol{k}'}f'(\varepsilon^+_{\boldsymbol{k}})}{(1-i\omega\tau^+_{\boldsymbol{k}})(1-i\omega\tau^+_{\boldsymbol{k}'})}\tilde{v}^{++}_{a\boldsymbol{k}}(\omega)\tilde{v}^{++}_{bc\boldsymbol{k}'}(\omega)I^{sk,1}(\boldsymbol{k},\boldsymbol{k}',\boldsymbol{k}'')\\
&\times\Big[\frac{\delta(\varepsilon^+_{\boldsymbol{k}}-\varepsilon^+_{\boldsymbol{k}''}+\hbar\omega)}{2h_{\boldsymbol{k}}+\hbar\omega}+\frac{\delta(\varepsilon^+_{\boldsymbol{k}}-\varepsilon^+_{\boldsymbol{k}''})}{2h_{\boldsymbol{k}}}\Big]\delta(\varepsilon^+_{\boldsymbol{k}}-\varepsilon^+_{\boldsymbol{k}'})+b\leftrightarrow c,
\end{aligned}\tag{154}$$

$$\begin{aligned}
\tilde{\mathcal{D}}^{sk,1}_{(d)}(\omega) =& \frac{ie^3\hbar}{4\pi}\int[dk]\int[dk']\int[dk'']\int_{-\infty}^{\infty}d\varepsilon f'(\varepsilon)\tilde{v}^{++}_{a\boldsymbol{k}}(\omega)\tilde{v}^{++}_{bc\boldsymbol{k}'}(\omega)\langle V^{++}_{\boldsymbol{k}\boldsymbol{k}'}V^{+-}_{\boldsymbol{k}'\boldsymbol{k}}\rangle_{dis}\langle V^{-+}_{\boldsymbol{k}\boldsymbol{k}''}V^{++}_{\boldsymbol{k}''\boldsymbol{k}}\rangle_{dis}\\
&\times\Big[\tilde{G}^R_{+\boldsymbol{k}}(\varepsilon_1^+)\tilde{G}^R_{+\boldsymbol{k}'}(\varepsilon_1^+)\tilde{G}^A_{+\boldsymbol{k}'}(\varepsilon)\tilde{G}^A_{-\boldsymbol{k}}(\varepsilon)\tilde{G}^A_{+\boldsymbol{k}''}(\varepsilon)\tilde{G}^A_{+\boldsymbol{k}}(\varepsilon)\\
&+\tilde{G}^R_{+\boldsymbol{k}}(\varepsilon)\tilde{G}^R_{+\boldsymbol{k}'}(\varepsilon)\tilde{G}^A_{+\boldsymbol{k}'}(\varepsilon_1^-)\tilde{G}^A_{-\boldsymbol{k}}(\varepsilon_1^-)\tilde{G}^A_{+\boldsymbol{k}''}(\varepsilon_1^-)\tilde{G}^A_{+\boldsymbol{k}}(\varepsilon_1^-)\Big]\\
\simeq& \frac{i\pi^2e^3}{\hbar}\int[dk]\int[dk']\int[dk'']\frac{\tau^+_{\boldsymbol{k}}\tau^+_{\boldsymbol{k}'}f'(\varepsilon^+_{\boldsymbol{k}})}{(1-i\omega\tau^+_{\boldsymbol{k}})(1-i\omega\tau^+_{\boldsymbol{k}'})}\tilde{v}^{++}_{a\boldsymbol{k}}(\omega)\tilde{v}^{++}_{bc\boldsymbol{k}'}(\omega)I^{sk,1}(\boldsymbol{k},\boldsymbol{k}',\boldsymbol{k}'')\\
&\times\Big[\frac{\delta(\varepsilon^+_{\boldsymbol{k}}-\varepsilon^+_{\boldsymbol{k}''})}{2h_{\boldsymbol{k}}}+\frac{\delta(\varepsilon^+_{\boldsymbol{k}}-\varepsilon^+_{\boldsymbol{k}''}-\hbar\omega)}{2h_{\boldsymbol{k}}-\hbar\omega}\Big]\delta(\varepsilon^+_{\boldsymbol{k}}-\varepsilon^+_{\boldsymbol{k}'})+b\leftrightarrow c,
\end{aligned}\tag{155}$$

$$\begin{aligned}
\tilde{\mathcal{D}}^{sk,1}_{(e)}(\omega) =& \frac{ie^3\hbar}{4\pi}\int[dk]\int[dk']\int[dk'']\int_{-\infty}^{\infty}d\varepsilon f'(\varepsilon)\tilde{v}^{++}_{a\boldsymbol{k}}(\omega)\tilde{v}^{++}_{bc\boldsymbol{k}'}(\omega)\langle V^{++}_{\boldsymbol{k}\boldsymbol{k}''}V^{-+}_{\boldsymbol{k}''\boldsymbol{k}}\rangle_{dis}\langle V^{++}_{\boldsymbol{k}''\boldsymbol{k}'}V^{+-}_{\boldsymbol{k}'\boldsymbol{k}''}\rangle_{dis}\\
&\times\Big[\tilde{G}^R_{+\boldsymbol{k}}(\varepsilon_1^+)\tilde{G}^R_{+\boldsymbol{k}''}(\varepsilon_1^+)\tilde{G}^R_{+\boldsymbol{k}'}(\varepsilon_1^+)\tilde{G}^A_{+\boldsymbol{k}'}(\varepsilon)\tilde{G}^A_{-\boldsymbol{k}''}(\varepsilon)\tilde{G}^A_{+\boldsymbol{k}}(\varepsilon)\\
&+\tilde{G}^R_{+\boldsymbol{k}}(\varepsilon)\tilde{G}^R_{+\boldsymbol{k}''}(\varepsilon)\tilde{G}^R_{+\boldsymbol{k}'}(\varepsilon)\tilde{G}^A_{+\boldsymbol{k}'}(\varepsilon_1^-)\tilde{G}^A_{-\boldsymbol{k}''}(\varepsilon_1^-)\tilde{G}^A_{+\boldsymbol{k}}(\varepsilon_1^-)\Big]\\
\simeq& \frac{i\pi^2e^3}{\hbar}\int[dk]\int[dk']\int[dk'']\frac{\tau^+_{\boldsymbol{k}}\tau^+_{\boldsymbol{k}'}f'(\varepsilon^+_{\boldsymbol{k}})}{(1-i\omega\tau^+_{\boldsymbol{k}})(1-i\omega\tau^+_{\boldsymbol{k}'})}\tilde{v}^{++}_{a\boldsymbol{k}}(\omega)\tilde{v}^{++}_{bc\boldsymbol{k}'}(\omega)I^{sk,1}(\boldsymbol{k}'',\boldsymbol{k},\boldsymbol{k}')\\
&\times\Big[\frac{\delta(\varepsilon^+_{\boldsymbol{k}}-\varepsilon^+_{\boldsymbol{k}''}+\hbar\omega)}{2h_{\boldsymbol{k}''}}+\frac{\delta(\varepsilon^+_{\boldsymbol{k}}-\varepsilon^+_{\boldsymbol{k}''})}{2h_{\boldsymbol{k}''}-\hbar\omega}\Big]\delta(\varepsilon^+_{\boldsymbol{k}}-\varepsilon^+_{\boldsymbol{k}'})+b\leftrightarrow c,
\end{aligned}\tag{156}$$

$$\tilde{\mathcal{D}}^{sk,1}_{(f)}(\omega) = \frac{ie^3\hbar}{4\pi}\int[dk]\int[dk']\int[dk'']\int_{-\infty}^{\infty} d\varepsilon f'(\varepsilon)\tilde{v}^{++}_{a\boldsymbol{k}}(\omega)\tilde{v}^{++}_{bc\boldsymbol{k}'}(\omega)\langle V^{+-}_{\boldsymbol{k}\boldsymbol{k}''}V^{++}_{\boldsymbol{k}''\boldsymbol{k}}\rangle_{dis}\langle V^{-+}_{\boldsymbol{k}''\boldsymbol{k}'}V^{++}_{\boldsymbol{k}'\boldsymbol{k}''}\rangle_{dis}$$
$$\times\Big[\tilde{G}^R_{+\boldsymbol{k}}(\varepsilon_1^+)\tilde{G}^R_{-\boldsymbol{k}''}(\varepsilon_1^+)\tilde{G}^R_{+\boldsymbol{k}'}(\varepsilon_1^+)\tilde{G}^A_{+\boldsymbol{k}'}(\varepsilon)\tilde{G}^A_{+\boldsymbol{k}''}(\varepsilon)\tilde{G}^A_{+\boldsymbol{k}}(\varepsilon)$$
$$+\tilde{G}^R_{+\boldsymbol{k}}(\varepsilon)\tilde{G}^R_{-\boldsymbol{k}''}(\varepsilon)\tilde{G}^R_{+\boldsymbol{k}'}(\varepsilon)\tilde{G}^A_{+\boldsymbol{k}'}(\varepsilon_1^-)\tilde{G}^A_{+\boldsymbol{k}''}(\varepsilon_1^-)\tilde{G}^A_{+\boldsymbol{k}}(\varepsilon_1^-)\Big]$$
$$\simeq \frac{i\pi^2e^3}{\hbar}\int[dk]\int[dk']\int[dk'']\frac{\tau^+_{\boldsymbol{k}}\tau^+_{\boldsymbol{k}'}f'(\varepsilon^+_{\boldsymbol{k}})}{(1-i\omega\tau^+_{\boldsymbol{k}})(1-i\omega\tau^+_{\boldsymbol{k}'})}\tilde{v}^{++}_{a\boldsymbol{k}}(\omega)\tilde{v}^{++}_{bc\boldsymbol{k}'}(\omega)I^{sk,1}(\boldsymbol{k}'',\boldsymbol{k},\boldsymbol{k}')$$
$$\times\Big[\frac{\delta(\varepsilon^+_{\boldsymbol{k}}-\varepsilon^+_{\boldsymbol{k}''})}{2h_{\boldsymbol{k}''}+\hbar\omega}+\frac{\delta(\varepsilon^+_{\boldsymbol{k}}-\varepsilon^+_{\boldsymbol{k}''}-\hbar\omega)}{2h_{\boldsymbol{k}''}}\Big]\delta(\varepsilon^+_{\boldsymbol{k}}-\varepsilon^+_{\boldsymbol{k}'})+b\leftrightarrow c. \tag{157}$$

In the dc limit, the intrinsic skew-scattering contributions up to the leading order in $n_iV_0^2$ can be obtained as

$$\Xi^{II\text{-}sk,1}_{abc} = \sum_{i=a}^{f}\frac{\partial\tilde{\mathcal{D}}^{sk,1}_{(i)}(\omega)}{\partial\omega}\Big|_{\omega=0}$$
$$= e^3\int[dk]\int[dk']\tau^+_{\boldsymbol{k}}\tau^+_{\boldsymbol{k}'}\varpi^{sk,1}_{\boldsymbol{k}\boldsymbol{k}'}\Big[(\tau^+_{\boldsymbol{k}}+\tau^+_{\boldsymbol{k}'})\tilde{v}^{++}_{a\boldsymbol{k}'}\tilde{v}^{++}_{bc\boldsymbol{k}}+\tau^+_{\boldsymbol{k}'}\delta\tilde{v}^{++}_{a\boldsymbol{k}'}\tilde{v}^{++}_{bc\boldsymbol{k}}$$
$$+\tau^+_{\boldsymbol{k}}\tilde{v}^{++}_{a\boldsymbol{k}'}\delta\tilde{v}^{++}_{bc\boldsymbol{k}}\Big]f'(\varepsilon^+_{\boldsymbol{k}})\delta(\varepsilon^+_{\boldsymbol{k}}-\varepsilon^+_{\boldsymbol{k}'})+b\leftrightarrow c, \tag{158}$$

And the intrinsic skew-scattering contribution to χ^{II}_{abc} in the dc limit is

$$\chi^{II\text{-}sk,1}_{abc} = \frac{e^3}{4}\int[dk]\int[dk']\tau^+_{\boldsymbol{k}}\tau^+_{\boldsymbol{k}'}\varpi^{sk,1}_{\boldsymbol{k}\boldsymbol{k}'}\Big[(\tau^+_{\boldsymbol{k}}+\tau^+_{\boldsymbol{k}'})\tilde{v}^{++}_{a\boldsymbol{k}'}\tilde{v}^{++}_{bc\boldsymbol{k}}+\tau^+_{\boldsymbol{k}'}\delta\tilde{v}^{++}_{a\boldsymbol{k}'}\tilde{v}^{++}_{bc\boldsymbol{k}}$$
$$+\tau^+_{\boldsymbol{k}}\tilde{v}^{++}_{a\boldsymbol{k}'}\delta\tilde{v}^{++}_{bc\boldsymbol{k}}\Big]f'(\varepsilon^+_{\boldsymbol{k}})\delta(\varepsilon^+_{\boldsymbol{k}}-\varepsilon^+_{\boldsymbol{k}'})+b\leftrightarrow c. \tag{159}$$

2. *Extrinsic skew-scattering contribution*

The diagrams corresponding to the extrinsic skew-scattering contribution with two-photon vertex are shown in Supplementary Figs. 15. By denoting the diagrams in Supplementary Fig. 15 (a)-(b) as $\tilde{\mathcal{D}}^{sk,2}_{(a)}(\omega)$-$\tilde{\mathcal{D}}^{sk,2}_{(b)}(\omega)$, we have

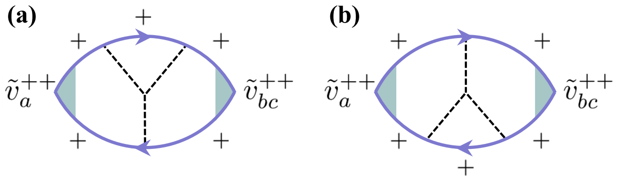

FIG. 15. Feynman diagrams of the two-photon extrinsic skew-scattering contribution.

$$\tilde{\mathcal{D}}^{sk,2}_{(a)}(\omega) = \frac{ie^3\hbar}{4\pi}\int[dk]\int[dk']\int[dk'']\int_{-\infty}^{\infty} d\varepsilon f'(\varepsilon)\tilde{v}^{++}_{a\boldsymbol{k}}\tilde{v}^{++}_{bc\boldsymbol{k}'}\langle V^{++}_{\boldsymbol{k}\boldsymbol{k}''}V^{++}_{\boldsymbol{k}''\boldsymbol{k}'}V^{++}_{\boldsymbol{k}'\boldsymbol{k}}\rangle_{dis}$$
$$\times\Big[\tilde{G}^R_{+\boldsymbol{k}}(\varepsilon_1^+)\tilde{G}^R_{+\boldsymbol{k}''}(\varepsilon_1^+)\tilde{G}^R_{+\boldsymbol{k}'}(\varepsilon_1^+)\tilde{G}^A_{+\boldsymbol{k}'}(\varepsilon)\tilde{G}^A_{+\boldsymbol{k}}(\varepsilon)+\tilde{G}^R_{+\boldsymbol{k}}(\varepsilon)\tilde{G}^R_{+\boldsymbol{k}''}(\varepsilon)\tilde{G}^R_{+\boldsymbol{k}'}(\varepsilon)\tilde{G}^A_{+\boldsymbol{k}'}(\varepsilon_1^-)\tilde{G}^A_{+\boldsymbol{k}}(\varepsilon_1^-)\Big]$$
$$\simeq -\frac{i\pi^2e^3}{\hbar}\int[dk]\int[dk']\int[dk'']\frac{\tau^+_{\boldsymbol{k}}\tau^+_{\boldsymbol{k}'}f'(\varepsilon^+_{\boldsymbol{k}})}{(1-i\omega\tau^+_{\boldsymbol{k}})(1-i\omega\tau^+_{\boldsymbol{k}'})}\tilde{v}^{++}_{a\boldsymbol{k}}\tilde{v}^{++}_{bc\boldsymbol{k}'}I^{sk,2}(\boldsymbol{k},\boldsymbol{k}',\boldsymbol{k}'')$$
$$\times\Big[\delta(\varepsilon^+_{\boldsymbol{k}}-\varepsilon^+_{\boldsymbol{k}''}+\hbar\omega)+\delta(\varepsilon^+_{\boldsymbol{k}}-\varepsilon^+_{\boldsymbol{k}''})\Big]\delta(\varepsilon^+_{\boldsymbol{k}}-\varepsilon^+_{\boldsymbol{k}'})+b\leftrightarrow c, \tag{160}$$

$$\tilde{\mathcal{D}}^{sk,2}_{(b)}(\omega) = \frac{ie^3\hbar}{4\pi}\int[dk]\int[dk']\int[dk'']\int_{-\infty}^{\infty}d\varepsilon f'(\varepsilon)\tilde{v}^{++}_{a\boldsymbol{k}}\tilde{v}^{++}_{bc\boldsymbol{k}'}\langle V^{++}_{\boldsymbol{k}\boldsymbol{k}'}V^{++}_{\boldsymbol{k}'\boldsymbol{k}''}V^{++}_{\boldsymbol{k}''\boldsymbol{k}}\rangle_{dis}$$
$$\times\Big[\tilde{G}^R_{+\boldsymbol{k}}(\varepsilon_1^+)\tilde{G}^R_{+\boldsymbol{k}'}(\varepsilon_1^+)\tilde{G}^A_{+\boldsymbol{k}'}(\varepsilon)\tilde{G}^A_{+\boldsymbol{k}''}(\varepsilon)\tilde{G}^A_{+\boldsymbol{k}}(\varepsilon)+\tilde{G}^R_{+\boldsymbol{k}}(\varepsilon)\tilde{G}^R_{+\boldsymbol{k}'}(\varepsilon)\tilde{G}^A_{+\boldsymbol{k}'}(\varepsilon_1^-)\tilde{G}^A_{+\boldsymbol{k}''}(\varepsilon_1^-)\tilde{G}^A_{+\boldsymbol{k}}(\varepsilon_1^-)\Big]$$
$$\simeq -\frac{i\pi^2e^3}{\hbar}\int[dk]\int[dk']\int[dk'']\frac{\tau^+_{\boldsymbol{k}}\tau^+_{\boldsymbol{k}'}f'(\varepsilon^+_{\boldsymbol{k}})}{(1-i\omega\tau^+_{\boldsymbol{k}})(1-i\omega\tau^+_{\boldsymbol{k}'})}\tilde{v}^{++}_{a\boldsymbol{k}}\tilde{v}^{++}_{bc\boldsymbol{k}'}I^{sk,2}(\boldsymbol{k},\boldsymbol{k}',\boldsymbol{k}'')$$
$$\times\Big[\delta(\varepsilon^+_{\boldsymbol{k}}-\varepsilon^+_{\boldsymbol{k}''}-\hbar\omega)+\delta(\varepsilon^+_{\boldsymbol{k}}-\varepsilon^+_{\boldsymbol{k}''})\Big]\delta(\varepsilon^+_{\boldsymbol{k}}-\varepsilon^+_{\boldsymbol{k}'})+b\leftrightarrow c. \tag{161}$$

In the dc limit, the extrinsic skew-scattering contributions up to the leading order in $n_iV_0^2$ can be obtained as

$$\Xi^{II\text{-}sk,2}_{abc} = \sum_{i=a}^{b}\frac{\partial\tilde{\mathcal{D}}^{sk,2}_{(i)}(\omega)}{\partial\omega}\Big|_{\omega=0}$$
$$= e^3\int[dk]\int[dk']\tau^+_{\boldsymbol{k}}\tau^+_{\boldsymbol{k}'}\varpi^{sk,2}_{\boldsymbol{k}\boldsymbol{k}'}\Big[(\tau^+_{\boldsymbol{k}}+\tau^+_{\boldsymbol{k}'})\tilde{v}^{++}_{a\boldsymbol{k}'}\tilde{v}^{++}_{bc\boldsymbol{k}}+\tau^+_{\boldsymbol{k}'}\delta\tilde{v}^{++}_{a\boldsymbol{k}'}\tilde{v}^{++}_{bc\boldsymbol{k}}$$
$$+\tau^+_{\boldsymbol{k}}\tilde{v}^{++}_{a\boldsymbol{k}'}\delta\tilde{v}^{++}_{bc\boldsymbol{k}}\Big]f'(\varepsilon^+_{\boldsymbol{k}})\delta(\varepsilon^+_{\boldsymbol{k}}-\varepsilon^+_{\boldsymbol{k}'})+b\leftrightarrow c, \tag{162}$$

And the extrinsic skew-scattering contribution to χ^{II}_{abc} in the dc limit is

$$\chi^{II\text{-}sk,2}_{abc} = \frac{e^3}{4}\int[dk]\int[dk']\tau^+_{\boldsymbol{k}}\tau^+_{\boldsymbol{k}'}\varpi^{sk,2}_{\boldsymbol{k}\boldsymbol{k}'}\Big[(\tau^+_{\boldsymbol{k}}+\tau^+_{\boldsymbol{k}'})\tilde{v}^{++}_{a\boldsymbol{k}'}\tilde{v}^{++}_{bc\boldsymbol{k}}+\tau^+_{\boldsymbol{k}'}\delta\tilde{v}^{++}_{a\boldsymbol{k}'}\tilde{v}^{++}_{bc\boldsymbol{k}}$$
$$+\tau^+_{\boldsymbol{k}}\tilde{v}^{++}_{a\boldsymbol{k}'}\delta\tilde{v}^{++}_{bc\boldsymbol{k}}\Big]f'(\varepsilon^+_{\boldsymbol{k}})\delta(\varepsilon^+_{\boldsymbol{k}}-\varepsilon^+_{\boldsymbol{k}'})+b\leftrightarrow c. \tag{163}$$

Supplementary Note 5. DIAGRAMMATICS: IV. REMARKS ON THE METHODOLOGY

In this section we turn to the discussions on the diagrammatic methodology. The discussion is mainly focused on the interpretation of the effective diagrammatics present in previous sections, including the role of the multi-photon processes and the results of the original intrinsic triangular diagrams as a comparison.

A. Role of the multi-photon processes

According to the calculation presented in Sec. Supplementary Note 2 B, the necessary conditions for the establishment of final expressions Supplementary Eq. (49)-(52) in the dc limit is that all the coupling vertices ($\hat{v}_a$, $\hat{v}_{ab}$, $\hat{v}_{abc}$) should be finite. However, there are many models that have vanishing multi-photon coupling vertices, such as the Dirac and Weyl fermion models, whose effective Hamiltonians only contain the linear $\boldsymbol{k}$-dependent terms. In these cases, one have $\hat{v}_{ab} = \hat{v}_{abc} = 0$, thus only the triangular diagrams in Fig.2 [(g) and (h)] have nonvanishing contributions. Then, it seems that one only needs to calculate the triangular diagrams, and the calculations presented in Sec. Supplementary Note 2 B become problematic and unnecessary. However, as we will show in the next subsection, a direct calculation of the triangular diagrams would lead to unphysical results in the dc limit. While a calculation of the same model from the effective diagrammatics obtained in Sec. Supplementary Note 2 B lead to physical results (see Sec. Supplementary Note 3 A) that are consistent with the semiclassical picture [1]. These facts indicate a rather puzzling conclusion that the multi-photon processes can not be ignored even in systems without multi-photon coupling vertices. The key to understand the problem is that the multi-photon coupling vertices always exist in Bloch bands. And the linear $\boldsymbol{k}$-dependent Hamiltonian is only a low energy effective description. Thus, a correct interpretation of the effective diagrammatic theory is one should first include the multi-photon coupling vertices to obtain the general expression in the dc limit, and then turn to the low energy effective Hamiltonian for detailed calculation as we have done in previous sections. Then, we can conclude that the multi-photon processes can not be ignored even in systems with linear $\boldsymbol{k}$-dependent low energy effective Hamiltonian. In other words, a correct starting point of an investigation concerning the nonlinear dc response should be Supplementary Eq. (49)-(52).

B. Results of the original intrinsic triangular diagrams

In this subsection we present the calculation of the original triangular diagrams. The "original" means we calculate the triangular diagrams directly instead of using the effective diagrammatics. To keep the discussion general and

simple, we adopt the multi-band Hamiltonian Supplementary Eq. (12) and only consider the intrinsic contribution, whose semiclassical results are well known [1]. The corresponding diagrams are shown in Supplementary Fig. 16-18. The contribution of diagram Supplementary Fig. 16 (a) can be written as

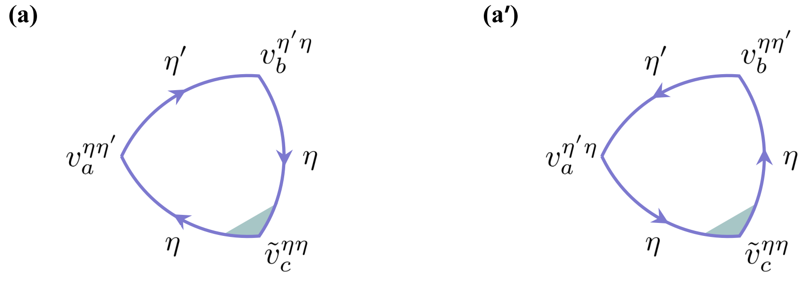

FIG. 16. Original triangular diagrams of the intrinsic contribution to the nonlinear Hall effect.

$$
\begin{aligned}
\mathcal{D}^{in}_{(a)}(\omega_b,\omega_c) =& \frac{e^3}{\beta\omega_b\omega_c}\int[dk]\sum_{\eta'\neq\eta}\sum_n v^{\eta\eta'}_{a\boldsymbol{k}}(i\omega_n, i\omega_n+i\nu_b+i\nu_c)\tilde{\mathcal{G}}_{\eta'\boldsymbol{k}}(i\omega_n+i\nu_b+i\nu_c)\\
&\times v^{\eta'\eta}_{b\boldsymbol{k}}(i\omega_n+i\nu_b+i\nu_c, i\omega_n+i\nu_c)\tilde{\mathcal{G}}_{\eta\boldsymbol{k}}(i\omega_n+i\nu_c)\tilde{v}^{\eta\eta}_{c\boldsymbol{k}}(i\omega_n+i\nu_c, i\omega_n)\tilde{\mathcal{G}}_{\eta\boldsymbol{k}}(i\omega_n)\\
\simeq& \frac{ie^3}{2\pi\omega_b\omega_c}\int[dk]\sum_{\eta'\neq\eta}\int_{-\infty}^{\infty}d\varepsilon f(\varepsilon)\Big\{v^{\eta\eta'}_{a\boldsymbol{k}}v^{\eta'\eta}_{b\boldsymbol{k}}\tilde{v}^{\eta\eta}_{c\boldsymbol{k}}\Big[\tilde{G}^R_{\eta'\boldsymbol{k}}(\varepsilon+\hbar\omega_b)\tilde{G}^R_{\eta\boldsymbol{k}}(\varepsilon)\tilde{G}^A_{\eta\boldsymbol{k}}(\varepsilon-\hbar\omega_c)\\
&-\tilde{G}^R_{\eta'\boldsymbol{k}}(\varepsilon+\hbar\omega_b+\hbar\omega_c)\tilde{G}^R_{\eta\boldsymbol{k}}(\varepsilon+\hbar\omega_c)\tilde{G}^A_{\eta\boldsymbol{k}}(\varepsilon)\Big]+v^{\eta\eta'}_{a\boldsymbol{k}}v^{\eta'\eta}_{b\boldsymbol{k}}v^{\eta\eta}_{c\boldsymbol{k}}\Big[\tilde{G}^R_{\eta'\boldsymbol{k}}(\varepsilon)\tilde{G}^A_{\eta\boldsymbol{k}}(\varepsilon-\hbar\omega_b)\tilde{G}^A_{\eta\boldsymbol{k}}(\varepsilon-\hbar\omega_b-\hbar\omega_c)\\
&-\tilde{G}^R_{\eta'\boldsymbol{k}}(\varepsilon+\hbar\omega_b)\tilde{G}^A_{\eta\boldsymbol{k}}(\varepsilon)\tilde{G}^A_{\eta\boldsymbol{k}}(\varepsilon-\hbar\omega_c)\Big]\Big\}\\
=& \frac{ie^3}{2\pi\omega_b\omega_c}\int[dk]\sum_{\eta'\neq\eta}\int_{-\infty}^{\infty}d\varepsilon\Big\{\Big[f(\varepsilon)-f(\varepsilon-\hbar\omega_c)\Big]v^{\eta\eta'}_{a\boldsymbol{k}}v^{\eta'\eta}_{b\boldsymbol{k}}\tilde{v}^{\eta\eta}_{c\boldsymbol{k}}\tilde{G}^R_{\eta'\boldsymbol{k}}(\varepsilon+\hbar\omega_b)\tilde{G}^R_{\eta\boldsymbol{k}}(\varepsilon)\tilde{G}^A_{\eta\boldsymbol{k}}(\varepsilon-\hbar\omega_c)\\
&+\Big[f(\varepsilon+\hbar\omega_b)-f(\varepsilon)\Big]v^{\eta\eta'}_{a\boldsymbol{k}}v^{\eta'\eta}_{b\boldsymbol{k}}v^{\eta\eta}_{c\boldsymbol{k}}\tilde{G}^R_{\eta'\boldsymbol{k}}(\varepsilon+\hbar\omega_b)\tilde{G}^A_{\eta\boldsymbol{k}}(\varepsilon)\tilde{G}^A_{\eta\boldsymbol{k}}(\varepsilon-\hbar\omega_c)\Big\}\\
\simeq& \frac{ie^3}{\hbar\omega_b\omega_c}\int[dk]\sum_{\eta'\neq\eta}\frac{\tau^\eta_{\boldsymbol{k}}}{1-i\omega_c\tau^\eta_{\boldsymbol{k}}}\frac{f(\varepsilon^\eta_{\boldsymbol{k}})-f(\varepsilon^\eta_{\boldsymbol{k}}-\hbar\omega_c)}{\varepsilon^\eta_{\boldsymbol{k}}-\varepsilon^{\eta'}_{\boldsymbol{k}}+\hbar\omega_b}v^{\eta\eta'}_{a\boldsymbol{k}}v^{\eta'\eta}_{b\boldsymbol{k}}\tilde{v}^{\eta\eta}_{c\boldsymbol{k}},
\end{aligned}
\tag{164}
$$

where we have set $i\nu_{a,b,c}\to\hbar\omega_{a,b,c}+i\delta$. In the last step, we have neglected the $\tilde{G}^R_{\eta'}\tilde{G}^A_\eta\tilde{G}^A_\eta$ term, because it is of the form $\tilde{G}^A_\eta\tilde{G}^A_\eta$ for the occupied η-band. Similarly, the contribution of diagram Supplementary Fig. 16 (a') can be written as

$$
\begin{aligned}
\mathcal{D}^{in}_{(a')}(\omega_b,\omega_c) \simeq& \frac{ie^3}{2\pi\omega_b\omega_c}\int[dk]\sum_{\eta'\neq\eta}\int_{-\infty}^{\infty}d\varepsilon f(\varepsilon)\Big\{v^{\eta'\eta}_{a\boldsymbol{k}}v^{\eta\eta}_{c\boldsymbol{k}}v^{\eta\eta'}_{b\boldsymbol{k}}\Big[\tilde{G}^R_{\eta\boldsymbol{k}}(\varepsilon+\hbar\omega_c)\tilde{G}^R_{\eta\boldsymbol{k}}(\varepsilon)\tilde{G}^A_{\eta'\boldsymbol{k}}(\varepsilon-\hbar\omega_b)\\
&-\tilde{G}^R_{\eta\boldsymbol{k}}(\varepsilon+\hbar\omega_b+\hbar\omega_c)\tilde{G}^R_{\eta\boldsymbol{k}}(\varepsilon+\hbar\omega_b)\tilde{G}^A_{\eta'\boldsymbol{k}}(\varepsilon)\Big]+v^{\eta'\eta}_{a\boldsymbol{k}}\tilde{v}^{\eta\eta}_{c\boldsymbol{k}}v^{\eta\eta'}_{b\boldsymbol{k}}\Big[\tilde{G}^R_{\eta\boldsymbol{k}}(\varepsilon)\tilde{G}^A_{\eta\boldsymbol{k}}(\varepsilon-\hbar\omega_c)\tilde{G}^A_{\eta'\boldsymbol{k}}(\varepsilon-\hbar\omega_b-\hbar\omega_c)\\
&-\tilde{G}^R_{\eta\boldsymbol{k}}(\varepsilon+\hbar\omega_c)\tilde{G}^A_{\eta\boldsymbol{k}}(\varepsilon)\tilde{G}^A_{\eta'\boldsymbol{k}}(\varepsilon-\hbar\omega_b)\Big]\Big\}\\
=& \frac{ie^3}{2\pi\omega_b\omega_c}\int[dk]\sum_{\eta'\neq\eta}\int_{-\infty}^{\infty}d\varepsilon\Big\{\Big[f(\varepsilon)-f(\varepsilon-\hbar\omega_b)\Big]v^{\eta'\eta}_{a\boldsymbol{k}}v^{\eta\eta'}_{b\boldsymbol{k}}v^{\eta\eta}_{c\boldsymbol{k}}\tilde{G}^R_{\eta\boldsymbol{k}}(\varepsilon+\hbar\omega_c)\tilde{G}^R_{\eta\boldsymbol{k}}(\varepsilon)\tilde{G}^A_{\eta'\boldsymbol{k}}(\varepsilon-\hbar\omega_b)\\
&+\Big[f(\varepsilon+\hbar\omega_c)-f(\varepsilon)\Big]v^{\eta'\eta}_{a\boldsymbol{k}}v^{\eta\eta'}_{b\boldsymbol{k}}\tilde{v}^{\eta\eta}_{c\boldsymbol{k}}\tilde{G}^R_{\eta\boldsymbol{k}}(\varepsilon+\hbar\omega_c)\tilde{G}^A_{\eta\boldsymbol{k}}(\varepsilon)\tilde{G}^A_{\eta'\boldsymbol{k}}(\varepsilon-\hbar\omega_b)\Big\}\\
\simeq& \frac{ie^3}{\hbar\omega_b\omega_c}\int[dk]\sum_{\eta'\neq\eta}\frac{\tau^\eta_{\boldsymbol{k}}}{1-i\omega_c\tau^\eta_{\boldsymbol{k}}}\frac{f(\varepsilon^\eta_{\boldsymbol{k}}+\hbar\omega_c)-f(\varepsilon^\eta_{\boldsymbol{k}})}{\varepsilon^\eta_{\boldsymbol{k}}-\varepsilon^{\eta'}_{\boldsymbol{k}}-\hbar\omega_b}v^{\eta'\eta}_{a\boldsymbol{k}}v^{\eta\eta'}_{b\boldsymbol{k}}\tilde{v}^{\eta\eta}_{c\boldsymbol{k}}.
\end{aligned}
\tag{165}
$$

The contribution of diagram Supplementary Fig. 17 (a) can be written as

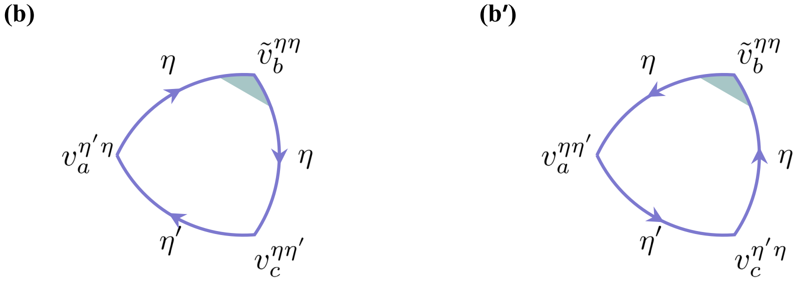

FIG. 17. Original triangular diagrams of the intrinsic contribution to the nonlinear Hall effect.

$$\begin{aligned}
\mathcal{D}^{in}_{(b)}(\omega_b,\omega_c) \simeq{}& \frac{ie^3}{2\pi\omega_b\omega_c}\int[dk]\sum_{\eta'\neq\eta}\int_{-\infty}^{\infty}d\varepsilon f(\varepsilon)\Big\{v^{\eta'\eta}_{a\boldsymbol{k}}v^{\eta\eta}_{b\boldsymbol{k}}v^{\eta\eta'}_{c\boldsymbol{k}}\Big[\tilde{G}^R_{\eta\boldsymbol{k}}(\varepsilon+\hbar\omega_b)\tilde{G}^R_{\eta\boldsymbol{k}}(\varepsilon)\tilde{G}^A_{\eta'\boldsymbol{k}}(\varepsilon-\hbar\omega_c)\\
&-\tilde{G}^R_{\eta\boldsymbol{k}}(\varepsilon+\hbar\omega_b+\hbar\omega_c)\tilde{G}^R_{\eta\boldsymbol{k}}(\varepsilon+\hbar\omega_c)\tilde{G}^A_{\eta'\boldsymbol{k}}(\varepsilon)\Big]+v^{\eta'\eta}_{a\boldsymbol{k}}\tilde{v}^{\eta\eta}_{b\boldsymbol{k}}v^{\eta\eta'}_{c\boldsymbol{k}}\Big[\tilde{G}^R_{\eta\boldsymbol{k}}(\varepsilon)\tilde{G}^A_{\eta\boldsymbol{k}}(\varepsilon-\hbar\omega_b)\tilde{G}^A_{\eta'\boldsymbol{k}}(\varepsilon-\hbar\omega_b-\hbar\omega_c)\\
&-\tilde{G}^R_{\eta\boldsymbol{k}}(\varepsilon+\hbar\omega_b)\tilde{G}^A_{\eta\boldsymbol{k}}(\varepsilon)\tilde{G}^A_{\eta'\boldsymbol{k}}(\varepsilon-\hbar\omega_c)\Big]\Big\}\\
={}& \frac{ie^3}{2\pi\omega_b\omega_c}\int[dk]\sum_{\eta'\neq\eta}\int_{-\infty}^{\infty}d\varepsilon\Big\{\Big[f(\varepsilon)-f(\varepsilon-\hbar\omega_c)\Big]v^{\eta\eta}_{a\boldsymbol{k}}v^{\eta'\eta}_{b\boldsymbol{k}}v^{\eta\eta'}_{c\boldsymbol{k}}\,\tilde{G}^R_{\eta\boldsymbol{k}}(\varepsilon+\hbar\omega_b)\tilde{G}^R_{\eta\boldsymbol{k}}(\varepsilon)\tilde{G}^A_{\eta'\boldsymbol{k}}(\varepsilon-\hbar\omega_c)\\
&+\Big[f(\varepsilon+\hbar\omega_b)-f(\varepsilon)\Big]v^{\eta'\eta}_{a\boldsymbol{k}}\tilde{v}^{\eta\eta}_{b\boldsymbol{k}}v^{\eta\eta'}_{c\boldsymbol{k}}\,\tilde{G}^R_{\eta\boldsymbol{k}}(\varepsilon+\hbar\omega_b)\tilde{G}^A_{\eta\boldsymbol{k}}(\varepsilon)\tilde{G}^A_{\eta'\boldsymbol{k}}(\varepsilon-\hbar\omega_c)\Big\}\\
\simeq{}& \frac{ie^3}{\hbar\omega_b\omega_c}\int[dk]\sum_{\eta'\neq\eta}\frac{\tau^\eta_{\boldsymbol{k}}}{1-i\omega_b\tau^\eta_{\boldsymbol{k}}}\frac{f(\varepsilon^\eta_{\boldsymbol{k}}+\hbar\omega_b)-f(\varepsilon^\eta_{\boldsymbol{k}})}{\varepsilon^\eta_{\boldsymbol{k}}-\varepsilon^{\eta'}_{\boldsymbol{k}}-\hbar\omega_c}v^{\eta'\eta}_{a\boldsymbol{k}}\tilde{v}^{\eta\eta}_{b\boldsymbol{k}}v^{\eta\eta'}_{c\boldsymbol{k}}.
\end{aligned}\tag{166}$$

The contribution of diagram Supplementary Fig. 17 (a’) can be written as

$$\begin{aligned}
\mathcal{D}^{in}_{(b')}(\omega_b,\omega_c) \simeq{}& \frac{ie^3}{2\pi\omega_b\omega_c}\int[dk]\sum_{\eta'\neq\eta}\int_{-\infty}^{\infty}d\varepsilon f(\varepsilon)\Big\{v^{\eta\eta'}_{a\boldsymbol{k}}v^{\eta'\eta}_{c\boldsymbol{k}}\tilde{v}^{\eta\eta}_{b\boldsymbol{k}}\Big[\tilde{G}^R_{\eta'\boldsymbol{k}}(\varepsilon+\hbar\omega_c)\tilde{G}^R_{\eta\boldsymbol{k}}(\varepsilon)\tilde{G}^A_{\eta\boldsymbol{k}}(\varepsilon-\hbar\omega_b)\\
&-\tilde{G}^R_{\eta'\boldsymbol{k}}(\varepsilon+\hbar\omega_b+\hbar\omega_c)\tilde{G}^R_{\eta\boldsymbol{k}}(\varepsilon+\hbar\omega_b)\tilde{G}^A_{\eta\boldsymbol{k}}(\varepsilon)\Big]+v^{\eta\eta'}_{a\boldsymbol{k}}v^{\eta'\eta}_{c\boldsymbol{k}}v^{\eta\eta}_{b\boldsymbol{k}}\Big[\tilde{G}^R_{\eta'\boldsymbol{k}}(\varepsilon)\tilde{G}^A_{\eta\boldsymbol{k}}(\varepsilon-\hbar\omega_c)\tilde{G}^A_{\eta\boldsymbol{k}}(\varepsilon-\hbar\omega_b-\hbar\omega_c)\\
&-\tilde{G}^R_{\eta'\boldsymbol{k}}(\varepsilon+\hbar\omega_c)\tilde{G}^A_{\eta\boldsymbol{k}}(\varepsilon)\tilde{G}^A_{\eta\boldsymbol{k}}(\varepsilon-\hbar\omega_b)\Big]\Big\}\\
={}& \frac{ie^3}{2\pi\omega_b\omega_c}\int[dk]\sum_{\eta'\neq\eta}\int_{-\infty}^{\infty}d\varepsilon\Big\{\Big[f(\varepsilon)-f(\varepsilon-\hbar\omega_b)\Big]v^{\eta\eta'}_{a\boldsymbol{k}}\tilde{v}^{\eta\eta}_{b\boldsymbol{k}}v^{\eta'\eta}_{c\boldsymbol{k}}\tilde{G}^R_{\eta'\boldsymbol{k}}(\varepsilon+\hbar\omega_c)\tilde{G}^R_{\eta\boldsymbol{k}}(\varepsilon)\tilde{G}^A_{\eta\boldsymbol{k}}(\varepsilon-\hbar\omega_b)\\
&+\Big[f(\varepsilon+\hbar\omega_c)-f(\varepsilon)\Big]v^{\eta\eta'}_{a\boldsymbol{k}}v^{\eta\eta}_{b\boldsymbol{k}}v^{\eta'\eta}_{c\boldsymbol{k}}\tilde{G}^R_{\eta'\boldsymbol{k}}(\varepsilon+\hbar\omega_c)\tilde{G}^A_{\eta\boldsymbol{k}}(\varepsilon)\tilde{G}^A_{\eta\boldsymbol{k}}(\varepsilon-\hbar\omega_b)\Big\}\\
\simeq{}& \frac{ie^3}{\hbar\omega_b\omega_c}\int[dk]\sum_{\eta'\neq\eta}\frac{\tau^\eta_{\boldsymbol{k}}}{1-i\omega_b\tau^\eta_{\boldsymbol{k}}}\frac{f(\varepsilon^\eta_{\boldsymbol{k}})-f(\varepsilon^\eta_{\boldsymbol{k}}-\hbar\omega_b)}{\varepsilon^\eta_{\boldsymbol{k}}-\varepsilon^{\eta'}_{\boldsymbol{k}}+\hbar\omega_c}v^{\eta\eta'}_{a\boldsymbol{k}}\tilde{v}^{\eta\eta}_{b\boldsymbol{k}}v^{\eta'\eta}_{c\boldsymbol{k}}.
\end{aligned}\tag{167}$$

The contribution of diagram Supplementary Fig. 18 (c) can be written as

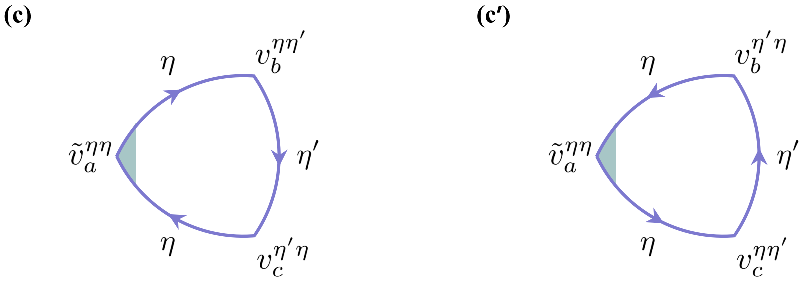

FIG. 18. Original triangular diagrams of the intrinsic contribution to the nonlinear Hall effect.

$$\mathcal{D}^{in}_{(c)}(\omega_b,\omega_c) \simeq \frac{ie^3}{2\pi\omega_b\omega_c}\int[dk]\sum_{\eta'\neq\eta}\int_{-\infty}^{\infty}d\varepsilon f(\varepsilon)\Big\{\tilde{v}^{\eta\eta}_{a\boldsymbol{k}}v^{\eta\eta'}_{b\boldsymbol{k}}v^{\eta'\eta}_{c\boldsymbol{k}}\Big[\tilde{G}^R_{\eta\boldsymbol{k}}(\varepsilon+\hbar\omega_b)\tilde{G}^R_{\eta'\boldsymbol{k}}(\varepsilon)\tilde{G}^A_{\eta\boldsymbol{k}}(\varepsilon-\hbar\omega_c)$$
$$-\tilde{G}^R_{\eta\boldsymbol{k}}(\varepsilon+\hbar\omega_b+\hbar\omega_c)\tilde{G}^R_{\eta'\boldsymbol{k}}(\varepsilon+\hbar\omega_c)\tilde{G}^A_{\eta\boldsymbol{k}}(\varepsilon)\Big]+\tilde{v}^{\eta\eta}_{a\boldsymbol{k}}v^{\eta\eta'}_{b\boldsymbol{k}}v^{\eta'\eta}_{c\boldsymbol{k}}\Big[\tilde{G}^R_{\eta\boldsymbol{k}}(\varepsilon)\tilde{G}^A_{\eta'\boldsymbol{k}}(\varepsilon-\hbar\omega_b)\tilde{G}^A_{\eta\boldsymbol{k}}(\varepsilon-\hbar\omega_b-\hbar\omega_c)$$
$$-\tilde{G}^R_{\eta\boldsymbol{k}}(\varepsilon+\hbar\omega_b)\tilde{G}^A_{\eta'\boldsymbol{k}}(\varepsilon)\tilde{G}^A_{\eta\boldsymbol{k}}(\varepsilon-\hbar\omega_c)\Big]\Big\}$$
$$=\frac{ie^3}{2\pi\omega_b\omega_c}\int[dk]\sum_{\eta'\neq\eta}\int_{-\infty}^{\infty}d\varepsilon\Big\{\Big[f(\varepsilon)-f(\varepsilon-\hbar\omega_c)\Big]\tilde{v}^{\eta\eta}_{a\boldsymbol{k}}v^{\eta\eta'}_{b\boldsymbol{k}}v^{\eta'\eta}_{c\boldsymbol{k}}\tilde{G}^R_{\eta\boldsymbol{k}}(\varepsilon+\hbar\omega_b)\tilde{G}^R_{\eta'\boldsymbol{k}}(\varepsilon)\tilde{G}^A_{\eta\boldsymbol{k}}(\varepsilon-\hbar\omega_c)$$
$$+\Big[f(\varepsilon+\hbar\omega_b)-f(\varepsilon)\Big]\tilde{v}^{\eta\eta}_{a\boldsymbol{k}}v^{\eta\eta'}_{b\boldsymbol{k}}v^{\eta'\eta}_{c\boldsymbol{k}}\tilde{G}^R_{\eta\boldsymbol{k}}(\varepsilon+\hbar\omega_b)\tilde{G}^A_{\eta'\boldsymbol{k}}(\varepsilon)\tilde{G}^A_{\eta\boldsymbol{k}}(\varepsilon-\hbar\omega_c)\Big\}$$
$$\simeq\frac{ie^3}{\hbar\omega_b\omega_c}\int[dk]\sum_{\eta'\neq\eta}\frac{\tau^\eta_{\boldsymbol{k}}}{1-i(\omega_b+\omega_c)\tau^\eta_{\boldsymbol{k}}}\frac{f(\varepsilon^\eta_{\boldsymbol{k}}+\hbar\omega_b)-f(\varepsilon^\eta_{\boldsymbol{k}}-\hbar\omega_c)}{\varepsilon^\eta_{\boldsymbol{k}}-\varepsilon^{\eta'}_{\boldsymbol{k}}}\tilde{v}^{\eta\eta}_{a\boldsymbol{k}}v^{\eta\eta'}_{b\boldsymbol{k}}v^{\eta'\eta}_{c\boldsymbol{k}}. \tag{168}$$

The contribution of diagram Supplementary Fig. 18 (c') can be written as

$$\mathcal{D}^{in}_{(c')}(\omega_b,\omega_c) \simeq \frac{ie^3}{2\pi\omega_b\omega_c}\int[dk]\sum_{\eta'\neq\eta}\int_{-\infty}^{\infty}d\varepsilon f(\varepsilon)\Big\{\tilde{v}^{\eta\eta}_{a\boldsymbol{k}}v^{\eta\eta'}_{c\boldsymbol{k}}v^{\eta'\eta}_{b\boldsymbol{k}}\Big[\tilde{G}^R_{\eta\boldsymbol{k}}(\varepsilon+\hbar\omega_c)\tilde{G}^R_{\eta'\boldsymbol{k}}(\varepsilon)\tilde{G}^A_{\eta\boldsymbol{k}}(\varepsilon-\hbar\omega_b)$$
$$-\tilde{G}^R_{\eta\boldsymbol{k}}(\varepsilon+\hbar\omega_b+\hbar\omega_c)\tilde{G}^R_{\eta'\boldsymbol{k}}(\varepsilon+\hbar\omega_b)\tilde{G}^A_{\eta\boldsymbol{k}}(\varepsilon)\Big]+\tilde{v}^{\eta\eta}_{a\boldsymbol{k}}v^{\eta\eta'}_{c\boldsymbol{k}}v^{\eta'\eta}_{b\boldsymbol{k}}\Big[\tilde{G}^R_{\eta\boldsymbol{k}}(\varepsilon)\tilde{G}^A_{\eta'\boldsymbol{k}}(\varepsilon-\hbar\omega_c)\tilde{G}^A_{\eta\boldsymbol{k}}(\varepsilon-\hbar\omega_b-\hbar\omega_c)$$
$$-\tilde{G}^R_{\eta\boldsymbol{k}}(\varepsilon+\hbar\omega_c)\tilde{G}^A_{\eta'\boldsymbol{k}}(\varepsilon)\tilde{G}^A_{\eta\boldsymbol{k}}(\varepsilon-\hbar\omega_b)\Big]\Big\}$$
$$=\frac{ie^3}{2\pi\omega_b\omega_c}\int[dk]\sum_{\eta'\neq\eta}\int_{-\infty}^{\infty}d\varepsilon\Big\{\Big[f(\varepsilon)-f(\varepsilon-\hbar\omega_b)\Big]\tilde{v}^{\eta\eta}_{a\boldsymbol{k}}v^{\eta'\eta}_{b\boldsymbol{k}}v^{\eta\eta'}_{c\boldsymbol{k}}\tilde{G}^R_{\eta\boldsymbol{k}}(\varepsilon+\hbar\omega_c)\tilde{G}^R_{\eta'\boldsymbol{k}}(\varepsilon)\tilde{G}^A_{\eta\boldsymbol{k}}(\varepsilon-\hbar\omega_b)$$
$$+\Big[f(\varepsilon+\hbar\omega_c)-f(\varepsilon)\Big]\tilde{v}^{\eta\eta}_{a\boldsymbol{k}}v^{\eta'\eta}_{b\boldsymbol{k}}v^{\eta\eta'}_{c\boldsymbol{k}}\tilde{G}^R_{\eta\boldsymbol{k}}(\varepsilon+\hbar\omega_c)\tilde{G}^A_{\eta'\boldsymbol{k}}(\varepsilon)\tilde{G}^A_{\eta\boldsymbol{k}}(\varepsilon-\hbar\omega_b)\Big\}$$
$$\simeq\frac{ie^3}{\hbar\omega_b\omega_c}\int[dk]\sum_{\eta'\neq\eta}\frac{\tau^\eta_{\boldsymbol{k}}}{1-i(\omega_b+\omega_c)\tau^\eta_{\boldsymbol{k}}}\frac{f(\varepsilon^\eta_{\boldsymbol{k}}+\hbar\omega_c)-f(\varepsilon^\eta_{\boldsymbol{k}}-\hbar\omega_b)}{\varepsilon^\eta_{\boldsymbol{k}}-\varepsilon^{\eta'}_{\boldsymbol{k}}}\tilde{v}^{\eta\eta}_{a\boldsymbol{k}}v^{\eta'\eta}_{b\boldsymbol{k}}v^{\eta\eta'}_{c\boldsymbol{k}}. \tag{169}$$

As only the real part of each diagram contributes to the conductivity in the diffusive limit ($\omega\tau \ll 1$), the combination of diagrams $\mathcal{D}^{in}_{(a)}(\omega_b,\omega_c)$ and $\mathcal{D}^{in}_{(a')}(\omega_b,\omega_c)$ can be approximately written as

$$\mathcal{D}^{in}_{(a)}(\omega_b,\omega_c)+\mathcal{D}^{in}_{(a')}(\omega_b,\omega_c)\simeq\frac{e^3}{\hbar\omega_b\omega_c}\int[dk]\sum_{\eta'\neq\eta}\frac{\tau^\eta_{\boldsymbol{k}}\tilde{v}^{\eta\eta}_{c\boldsymbol{k}}\mathrm{Im}(v^{\eta\eta'}_{a\boldsymbol{k}}v^{\eta'\eta}_{b\boldsymbol{k}})}{1-i\omega_c\tau^\eta_{\boldsymbol{k}}}\Big[\frac{f(\varepsilon^\eta_{\boldsymbol{k}}+\hbar\omega_c)-f(\varepsilon^\eta_{\boldsymbol{k}})}{\varepsilon^\eta_{\boldsymbol{k}}-\varepsilon^{\eta'}_{\boldsymbol{k}}-\hbar\omega_b}-\frac{f(\varepsilon^\eta_{\boldsymbol{k}})-f(\varepsilon^\eta_{\boldsymbol{k}}-\hbar\omega_c)}{\varepsilon^\eta_{\boldsymbol{k}}-\varepsilon^{\eta'}_{\boldsymbol{k}}+\hbar\omega_b}\Big]$$
$$\simeq\frac{e^3}{\hbar\omega_b\omega_c}\int[dk]\sum_{\eta'\neq\eta}\frac{\tau^\eta_{\boldsymbol{k}}\tilde{v}^{\eta\eta}_{c\boldsymbol{k}}\mathrm{Im}(v^{\eta\eta'}_{a\boldsymbol{k}}v^{\eta'\eta}_{b\boldsymbol{k}})}{1-i\omega_c\tau^\eta_{\boldsymbol{k}}}\Big[\hbar\omega_b\frac{f(\varepsilon^\eta_{\boldsymbol{k}}+\hbar\omega_c)-f(\varepsilon^\eta_{\boldsymbol{k}}-\hbar\omega_c)}{(\varepsilon^\eta_{\boldsymbol{k}}-\varepsilon^{\eta'}_{\boldsymbol{k}})^2}$$
$$+\frac{f(\varepsilon^\eta_{\boldsymbol{k}}+\hbar\omega_c)+f(\varepsilon^\eta_{\boldsymbol{k}}-\hbar\omega_c)-2f(\varepsilon^\eta_{\boldsymbol{k}})}{\varepsilon^\eta_{\boldsymbol{k}}-\varepsilon^{\eta'}_{\boldsymbol{k}}}\Big]$$
$$\simeq -e^2\int[dk]\frac{\tau^\eta_{\boldsymbol{k}}\tilde{v}^{\eta\eta}_{c\boldsymbol{k}}}{1-i\omega_c\tau^\eta_{\boldsymbol{k}}}\Big[\frac{e}{\hbar}\frac{f(\varepsilon^\eta_{\boldsymbol{k}}+\hbar\omega_c)-f(\varepsilon^\eta_{\boldsymbol{k}}-\hbar\omega_c)}{2\hbar\omega_c}\Omega^{ab}_{\eta\boldsymbol{k}}$$
$$+\frac{\omega_c}{\omega_b}\frac{f(\varepsilon^\eta_{\boldsymbol{k}}+\hbar\omega_c)+f(\varepsilon^\eta_{\boldsymbol{k}}-\hbar\omega_c)-2f(\varepsilon^\eta_{\boldsymbol{k}})}{(\hbar\omega_c)^2}m^{ab}_{\eta\boldsymbol{k}}\Big], \tag{170}$$

where we have used the multi-band expressions of the Berry curvature $\Omega^{ab}_{\eta\boldsymbol{k}}$ and orbital moment $m^{ab}_{\eta\boldsymbol{k}}$ [12]

$$\Omega^{ab}_{\eta\boldsymbol{k}}=\varepsilon^{abc}\Omega^c_{\eta\boldsymbol{k}}=-2\sum_{\eta'\neq\eta}\frac{\mathrm{Im}[\langle u^\eta_{\boldsymbol{k}}|\partial\hat{\mathcal{H}}_0/\partial k_a|u^{\eta'}_{\boldsymbol{k}}\rangle\langle u^{\eta'}_{\boldsymbol{k}}|\partial\hat{\mathcal{H}}_0/\partial k_b|u^\eta_{\boldsymbol{k}}\rangle]}{(\varepsilon^\eta_{\boldsymbol{k}}-\varepsilon^{\eta'}_{\boldsymbol{k}})^2}=-2\hbar^2\sum_{\eta'\neq\eta}\frac{\mathrm{Im}(v^{\eta\eta'}_{a\boldsymbol{k}}v^{\eta'\eta}_{b\boldsymbol{k}})}{(\varepsilon^\eta_{\boldsymbol{k}}-\varepsilon^{\eta'}_{\boldsymbol{k}})^2}, \tag{171}$$

$$m^{ab}_{\eta\boldsymbol{k}}=\varepsilon^{abc}m^c_{\eta\boldsymbol{k}}=-\frac{e}{\hbar}\sum_{\eta'\neq\eta}\frac{\mathrm{Im}[\langle u^\eta_{\boldsymbol{k}}|\partial\hat{\mathcal{H}}_0/\partial k_a|u^{\eta'}_{\boldsymbol{k}}\rangle\langle u^{\eta'}_{\boldsymbol{k}}|\partial\hat{\mathcal{H}}_0/\partial k_b|u^\eta_{\boldsymbol{k}}\rangle]}{\varepsilon^\eta_{\boldsymbol{k}}-\varepsilon^{\eta'}_{\boldsymbol{k}}}=-e\hbar\sum_{\eta'\neq\eta}\frac{\mathrm{Im}(v^{\eta\eta'}_{a\boldsymbol{k}}v^{\eta'\eta}_{b\boldsymbol{k}})}{\varepsilon^\eta_{\boldsymbol{k}}-\varepsilon^{\eta'}_{\boldsymbol{k}}}. \tag{172}$$

Similarly, the combination of diagrams $\mathcal{D}^{in}_{(b)}(\omega_b,\omega_c)$ and $\mathcal{D}^{in}_{(b')}(\omega_b,\omega_c)$ can be approximately written as

$$\mathcal{D}^{in}_{(b)}(\omega_b,\omega_c)+\mathcal{D}^{in}_{(b')}(\omega_b,\omega_c)\simeq -e^2\int[dk]\frac{\tau^{\eta}_{\boldsymbol{k}}\tilde{v}^{\eta\eta}_{b\boldsymbol{k}}}{1-i\omega_b\tau^{\eta}_{\boldsymbol{k}}}\Big[\frac{e}{\hbar}\frac{f(\varepsilon^{\eta}_{\boldsymbol{k}}+\hbar\omega_b)-f(\varepsilon^{\eta}_{\boldsymbol{k}}-\hbar\omega_b)}{2\hbar\omega_b}\Omega^{ac}_{\eta\boldsymbol{k}} + \frac{\omega_b}{\omega_c}\frac{f(\varepsilon^{\eta}_{\boldsymbol{k}}+\hbar\omega_b)+f(\varepsilon^{\eta}_{\boldsymbol{k}}-\hbar\omega_b)-2f(\varepsilon^{\eta}_{\boldsymbol{k}})}{(\hbar\omega_b)^2}m^{ac}_{\eta\boldsymbol{k}}\Big]. \tag{173}$$

While the combination of diagrams $\mathcal{D}^{in}_{(c)}(\omega_b,\omega_c)$ and $\mathcal{D}^{in}_{(c')}(\omega_b,\omega_c)$ is different, which can be approximately written as

$$\begin{aligned}\mathcal{D}^{in}_{(c)}(\omega_b,\omega_c)+\mathcal{D}^{in}_{(c')}(\omega_b,\omega_c)&\simeq \frac{e^3}{\hbar\omega_b\omega_c}\int[dk]\frac{\tau^{\eta}_{\boldsymbol{k}}\tilde{v}^{\eta\eta}_{a\boldsymbol{k}}\mathrm{Im}(v^{\eta\eta'}_{b\boldsymbol{k}}v^{\eta'\eta}_{c\boldsymbol{k}})}{1-i(\omega_b+\omega_c)\tau^{\eta}_{\boldsymbol{k}}}\Big[\frac{f(\varepsilon^{\eta}_{\boldsymbol{k}}+\hbar\omega_c)-f(\varepsilon^{\eta}_{\boldsymbol{k}}-\hbar\omega_b)}{\varepsilon^{\eta}_{\boldsymbol{k}}-\varepsilon^{\eta'}_{\boldsymbol{k}}} - \frac{f(\varepsilon^{\eta}_{\boldsymbol{k}}+\hbar\omega_b)-f(\varepsilon^{\eta}_{\boldsymbol{k}}-\hbar\omega_c)}{\varepsilon^{\eta}_{\boldsymbol{k}}-\varepsilon^{\eta'}_{\boldsymbol{k}}}\Big]\\ &\simeq -\frac{e^2}{\hbar^2\omega_b\omega_c}\int[dk]\frac{\tau^{\eta}_{\boldsymbol{k}}\tilde{v}^{\eta\eta}_{a\boldsymbol{k}}m^{bc}_{\eta\boldsymbol{k}}}{1-i(\omega_b+\omega_c)\tau^{\eta}_{\boldsymbol{k}}}\Big[f(\varepsilon^{\eta}_{\boldsymbol{k}}+\hbar\omega_c)+f(\varepsilon^{\eta}_{\boldsymbol{k}}-\hbar\omega_c) -f(\varepsilon^{\eta}_{\boldsymbol{k}}+\hbar\omega_b)-f(\varepsilon^{\eta}_{\boldsymbol{k}}-\hbar\omega_b)\Big].\end{aligned} \tag{174}$$

Considering the special case $\omega_b=\omega_c=\omega$, we have

$$\mathcal{D}^{in}_{(a)}(\omega,\omega)+\mathcal{D}^{in}_{(a')}(\omega,\omega)\simeq -e^2\int[dk]\frac{\tau^{\eta}_{\boldsymbol{k}}\tilde{v}^{\eta\eta}_{c\boldsymbol{k}}}{1-i\omega\tau^{\eta}_{\boldsymbol{k}}}\Big[\frac{e}{\hbar}\frac{f(\varepsilon^{\eta}_{\boldsymbol{k}}+\hbar\omega)-f(\varepsilon^{\eta}_{\boldsymbol{k}}-\hbar\omega)}{2\hbar\omega}\Omega^{ab}_{\eta\boldsymbol{k}} + \frac{f(\varepsilon^{\eta}_{\boldsymbol{k}}+\hbar\omega)+f(\varepsilon^{\eta}_{\boldsymbol{k}}-\hbar\omega)-2f(\varepsilon^{\eta}_{\boldsymbol{k}})}{(\hbar\omega)^2}m^{ab}_{\eta\boldsymbol{k}}\Big], \tag{175}$$

$$\mathcal{D}^{in}_{(b)}(\omega,\omega)+\mathcal{D}^{in}_{(b')}(\omega,\omega)\simeq -e^2\int[dk]\frac{\tau^{\eta}_{\boldsymbol{k}}\tilde{v}^{\eta\eta}_{c\boldsymbol{k}}}{1-i\omega\tau^{\eta}_{\boldsymbol{k}}}\Big[\frac{e}{\hbar}\frac{f(\varepsilon^{\eta}_{\boldsymbol{k}}+\hbar\omega)-f(\varepsilon^{\eta}_{\boldsymbol{k}}-\hbar\omega)}{2\hbar\omega}\Omega^{ac}_{\eta\boldsymbol{k}} + \frac{f(\varepsilon^{\eta}_{\boldsymbol{k}}+\hbar\omega)+f(\varepsilon^{\eta}_{\boldsymbol{k}}-\hbar\omega)-2f(\varepsilon^{\eta}_{\boldsymbol{k}})}{(\hbar\omega)^2}m^{ac}_{\eta\boldsymbol{k}}\Big], \tag{176}$$

$$\mathcal{D}^{in}_{(c)}(\omega,\omega)+\mathcal{D}^{in}_{(c')}(\omega,\omega)\simeq 0, \tag{177}$$

and the combination of the above six diagrams can be denoted as

$$\begin{aligned}\Xi^{in}_{abc}(\omega,\omega)&=\sum_{i=a}^{c}\Big[\mathcal{D}^{in}_{(i)}(\omega,\omega)+\mathcal{D}^{in}_{(i')}(\omega,\omega)\Big]\\ &\simeq -e^2\int[dk]\frac{\tau^{\eta}_{\boldsymbol{k}}\tilde{v}^{\eta\eta}_{c\boldsymbol{k}}}{1-i\omega\tau^{\eta}_{\boldsymbol{k}}}\Big[\frac{e}{\hbar}\frac{f(\varepsilon^{\eta}_{\boldsymbol{k}}+\hbar\omega)-f(\varepsilon^{\eta}_{\boldsymbol{k}}-\hbar\omega)}{2\hbar\omega}\Omega^{ab}_{\eta\boldsymbol{k}} + \frac{f(\varepsilon^{\eta}_{\boldsymbol{k}}+\hbar\omega)+f(\varepsilon^{\eta}_{\boldsymbol{k}}-\hbar\omega)-2f(\varepsilon^{\eta}_{\boldsymbol{k}})}{(\hbar\omega)^2}m^{ab}_{\eta\boldsymbol{k}}\Big]+b\leftrightarrow c.\end{aligned} \tag{178}$$

Thus the intrinsic contributions to the double- and zero-frequency nonlinear Hall conductivities in the dc limit are

$$\lim_{\omega\to 0}\chi^{in}_{abc}(\omega,\omega)\simeq -\frac{e^2}{4}\int[dk]\tau^{\eta}_{\boldsymbol{k}}\tilde{v}^{\eta\eta}_{c\boldsymbol{k}}\Big[\frac{e}{\hbar}f'(\varepsilon^{\eta}_{\boldsymbol{k}})\Omega^{ab}_{\eta\boldsymbol{k}}+f''(\varepsilon^{\eta}_{\boldsymbol{k}})m^{ab}_{\eta\boldsymbol{k}}\Big]+b\leftrightarrow c, \tag{179}$$

$$\lim_{\omega\to 0}\xi^{in}_{abc}(\omega,\omega)\simeq -\frac{e^2}{4}\int[dk]\tau^{\eta}_{\boldsymbol{k}}\tilde{v}^{\eta\eta}_{c\boldsymbol{k}}\Big[\frac{e}{\hbar}f'(\varepsilon^{\eta}_{\boldsymbol{k}})\Omega^{ab}_{\eta\boldsymbol{k}}-f''(\varepsilon^{\eta}_{\boldsymbol{k}})m^{ab}_{\eta\boldsymbol{k}}\Big]+b\leftrightarrow c, \tag{180}$$

where $f''(\varepsilon)\equiv\partial^2 f(\varepsilon)/\partial\varepsilon^2$. These results lead to two problems:

1. Compared with the semiclassical results, there are extra f''-terms in the intrinsic contribution in Supplementary Eq. (179) and (180), which in the multi-band formalism has to be interpreted as the orbital moment contribution. However, in the Boltzmann calculation, the orbital moment should not appear in the absence of the magnetic field [12].

2. The f''-terms in Supplementary Eq. (179) and Supplementary Eq. (180) have sign difference. For $\lim_{\omega\to 0}\chi^{in}_{abc}(\omega,\omega)$ and $\lim_{\omega\to 0}\xi^{in}_{abc}(\omega,\omega)$, the Berry curvature related f'-terms share the same sign while the f''-terms have opposite signs. This sign difference can be roughly interpreted as follows: both f' and f'' are defined according to one frequency ω

$$f'(\varepsilon)=\lim_{\omega\to 0}\frac{f(\varepsilon+\omega)-f(\varepsilon)}{\omega}=\lim_{\omega\to 0}\frac{f(\varepsilon)-f(\varepsilon-\omega)}{\omega},\qquad f''(\varepsilon)=\lim_{\omega\to 0}\frac{f(\varepsilon+\omega)+f(\varepsilon-\omega)-2f(\varepsilon)}{\omega^2}, \tag{181}$$

thus, for a quadratic nonlinear response function with two frequency ω_b and ω_c, we usually have

$$\Xi(\omega_b,\omega_c) \sim \lim_{\omega_{b,c}\to 0}\left[A(\boldsymbol{k})\frac{f(\varepsilon+\omega_c)-f(\varepsilon)}{\omega_c}+B(\boldsymbol{k})\frac{\omega_c}{\omega_b}\frac{f(\varepsilon+\omega_c)+f(\varepsilon-\omega_c)-2f(\varepsilon)}{\omega_c^2}\right], \tag{182}$$

where $A(\boldsymbol{k})$ and $B(\boldsymbol{k})$ are general functions of $\boldsymbol{k}$. The sign difference comes from the ω_c/ω_b factor, which changes sign when one of the frequency changes its sign [such as the cases in $\Xi(\omega_b,-\omega_c)$ and $\Xi(-\omega_b,\omega_c)$]. However, in the Boltzmann calculation, the $\omega\to 0$ limit is irrelevant with the $\partial_{\boldsymbol{k}}$ operation, so there is no sign difference and we always have $\lim_{\omega\to 0}\chi^{in}_{abc}(\omega,\omega)=\lim_{\omega\to 0}\xi^{in}_{abc}(\omega,\omega)$.

Supplementary Note 6. DIAGRAMMATICS: V. GENERAL ASPECTS OF THE DIAGRAMMATIC THEORY

In addition to the calculation convenience and more accurate description of the quantum effect, another powerful point of the diagrammatic theory is that one can directly draw some general conclusions from the diagrams without detailed calculation. In this section, we discuss some general aspects of the diagrammatic theory. Still, we consider the general two-band model with the Fermi level intersecting the upper (+) band.

A. Intrinsic triangular diagrams

The intrinsic diagrams are characterised by two off-diagonal velocities, which consequently yields one Green's function of the occupied lower band (−) on the triangular edge. The rotation of the triangular diagram indicates the total number of the intrinsic diagrams is 3 as summarised in Supplementary Fig. 5. Although there are clockwise and anticlockwise counterparts for each triangular diagram, we treat them as one diagram since we do not need to calculate them explicitly in the effective diagrammatics.

B. Side-jump triangular diagrams

The anomalous distribution induced side-jump diagrams are characterized with off-diagonal velocity on the input vertex, and are summarised in Supplementary Fig. 7. We first focus on the anomalous velocity induced side-jump diagrams, which include relatively fewer diagrams. According to Supplementary Fig. 6, the total number of the anomalous velocity induced side-jump diagrams is 6. These diagrams are characterised by one off-diagonal velocity on the output vertex (a), which consequently yields an off-diagonal scattering event $\langle V^{-+}_{\boldsymbol{k}\boldsymbol{k}'}V^{++}_{\boldsymbol{k}'\boldsymbol{k}}\rangle$. As these two quantities always appear together, the characteristic quantity of the side-jump contribution is usually referred to as $v^{+-}_{a\boldsymbol{k}}\langle V^{-+}_{\boldsymbol{k}\boldsymbol{k}'}V^{++}_{\boldsymbol{k}'\boldsymbol{k}}\rangle$. From Supplementary Fig. 6 (a)-(d), we can see that one main effect of this side-jump characteristic quantity is to turn the bare off-diagonal velocity into a modified diagonal one. This observation indicates that we can define a new velocity that is associated with the side-jump contribution, the so-called side-jump velocity $\boldsymbol{v}^{sj}$. Its definition within the diagrammatic representation is shown in Supplementary Fig. 19 (a).

For the general two-band model, the corresponding expression of the side-jump velocity is Supplementary Eq. (191). One can easily show that this expression is consistent with previous semiclassical results [4]. Note that the edge corrections in Supplementary Fig. 6 (a)-(d) can be absorbed into the disorder-induced modification to the side-jump velocity. That is, we can eliminate the edge correction by defining the modified side-jump velocity $\tilde{\boldsymbol{v}}^{sj}$. Thus, in our quantum diagrammatic theory, the relevant quantity is not the bare side-jump velocity shown in Supplementary Fig. 19 (a) but the disorder modified one shown in Supplementary Fig. 19 (b). This is distinct from the case in linear response theory, in which the diffuson can always be absorbed by the other velocity operator, thus no edge correction is present.

In addition to the side-jump velocity, there are two more anomalous velocity induced side-jump diagrams that do not have semiclassical correspondence [Supplementary Fig. 6 (e) and (f)]. In these diagrams, the off-diagonal scattering event $\langle V^{-+}_{\boldsymbol{k}\boldsymbol{k}'}V^{++}_{\boldsymbol{k}'\boldsymbol{k}}\rangle$ tends to turn the diagonal velocity into an off-diagonal one. Following the same logic as the side-jump velocity, we define a modified off-diagonal velocity as shown in Supplementary Fig. 20. As we shall see, the modified off-diagonal velocity also exists in skew-scattering diagrams, and thus it is a common feature of our quantum nonlinear theory.

Note that the anomalous distribution induced side-jump diagrams can be obtained via a three-fold rotation of the anomalous velocity induced ones, and thus the total number is $6\times 2=12$. Then, based on the modified side-jump and off-diagonal velocity, all the side-jump diagrams can be recasted into a more simplified form as shown in Supplementary Fig. 21. In this form, the Green's functions are arranged in the simplest manner, thus the general

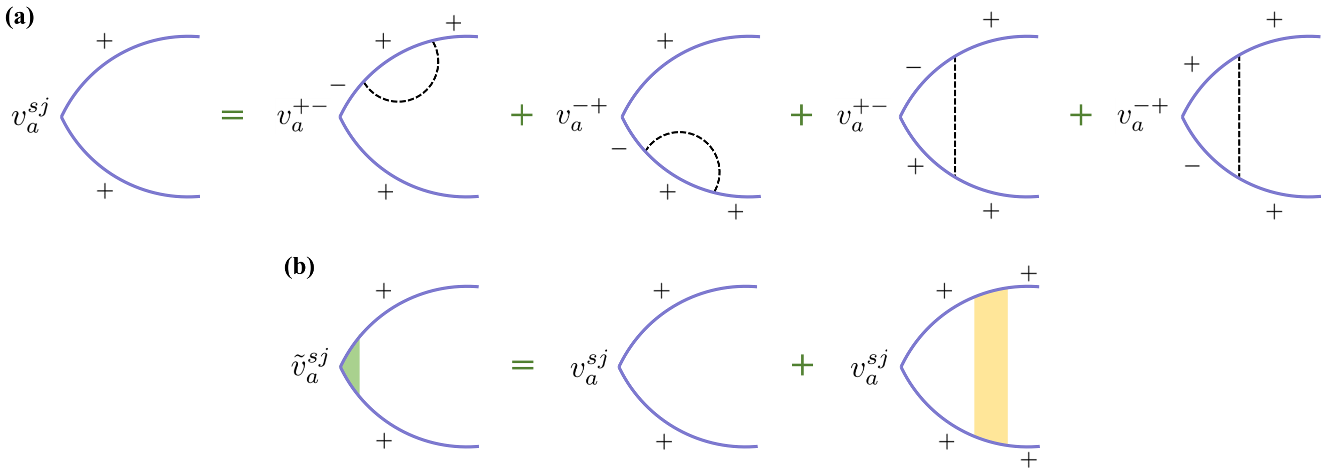

FIG. 19. Diagrammatic representation of the side-jump velocity and its disorder-induced modification.

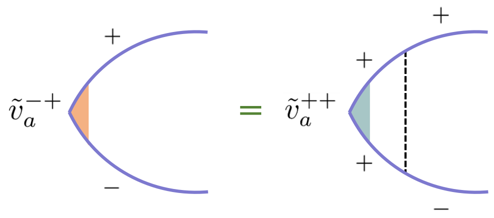

FIG. 20. Diagrammatic representation of the modified off-diagonal velocity.

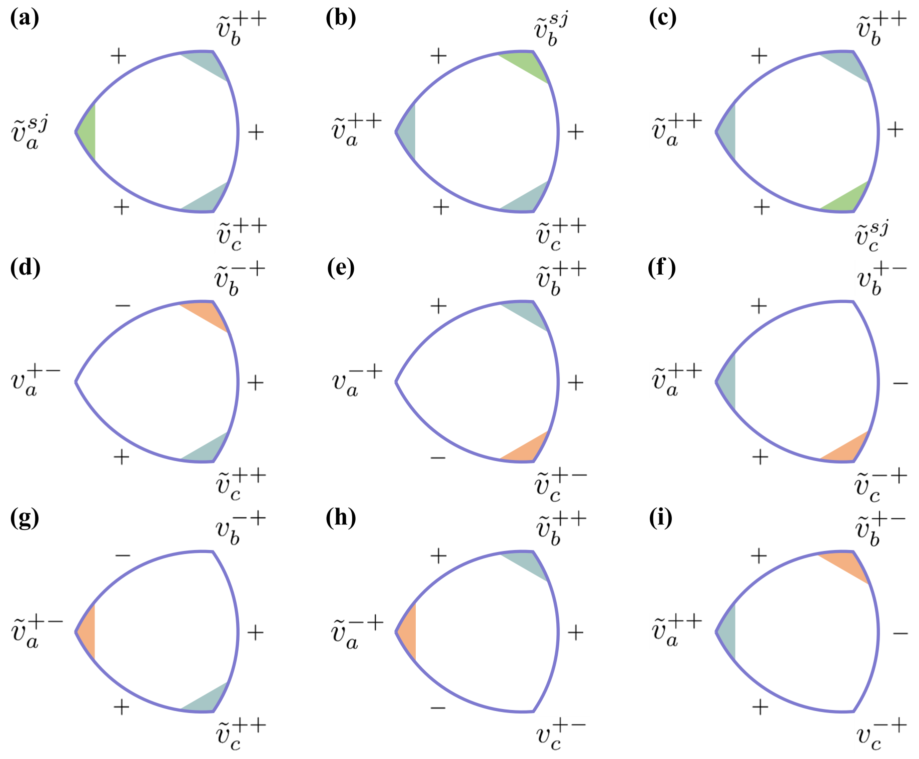

FIG. 21. Feynman diagrams of the side-jump contribution after the vertex redefinition.

features of the side-jump diagrams become more transparent. As shown in Supplementary Fig. 21, there are only two types of diagrams after the vertex redefinition, one is the diagonal triangular diagrams and the other is the modified off-diagonal diagrams.

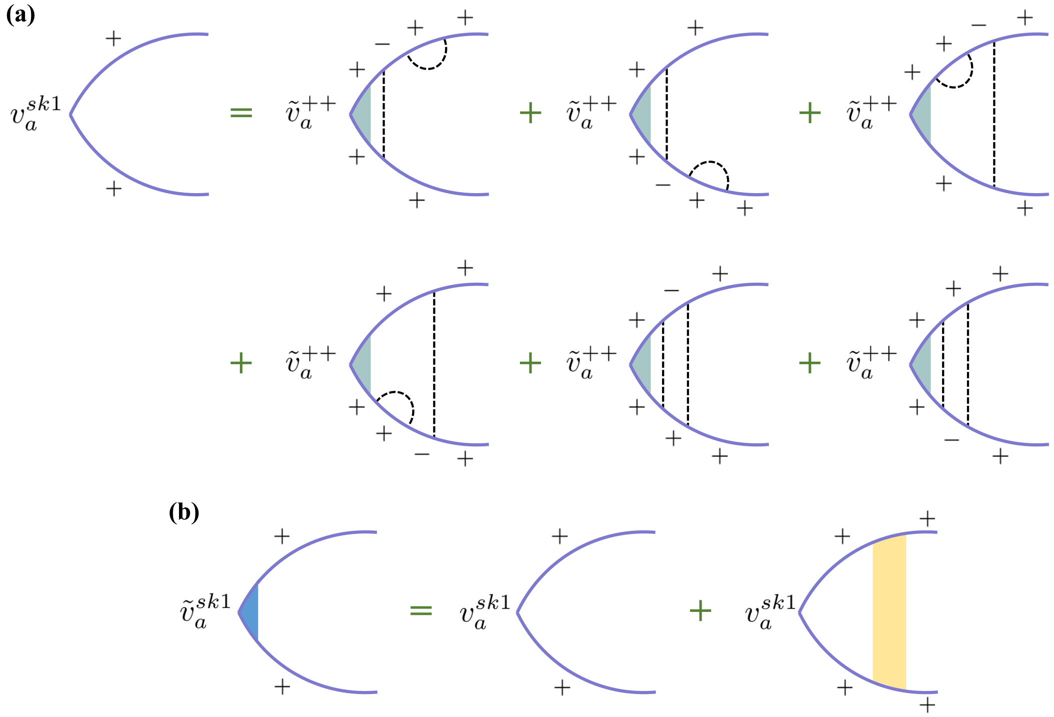

FIG. 22. Diagrammatic representation of the intrinsic skew-scattering velocity and its disorder-induced modification.

C. Skew-scattering triangular diagrams

The skew-scattering contribution corresponds to the diagrams with no off-diagonal velocity, thus only diagonal velocities are relevant. The skew-scattering contribution can be classified into two categories as intrinsic (summarised in Supplementary Fig. 8) and extrinsic (summarised in Supplementary Fig. 9) skew-scattering according to their characteristic scattering processes [4]. The first one is from the leading asymmetric scattering contribution due to the Gaussian disorder. Following the previous work [4], here we adopt the non-crossing approximation, the resultant diagrams are then featured by the scattering processes $\langle V^{-+}_{\boldsymbol{kk'}} V^{++}_{\boldsymbol{k'k}} \rangle \langle V^{+-}_{\boldsymbol{k''k}} V^{++}_{\boldsymbol{kk''}} \rangle$. Thus, the total number of the intrinsic skew-scattering diagrams is 21 as shown in Supplementary Fig. 8. The second one is from the leading asymmetric scattering contribution due to the non-Gaussian disorder, and is featured by the scattering processes $\langle V^{++}_{\boldsymbol{kk'}} V^{++}_{\boldsymbol{k'k''}} V^{++}_{\boldsymbol{k''k}} \rangle$ with the total number of diagrams being 7 as shown in Supplementary Fig. 9.

We first address the intrinsic skew-scattering diagrams. Following the same logic as the side-jump velocity, to simplify the intrinsic skew-scattering diagrams we need an intrinsic skew-scattering velocity. By examining the diagrams in Supplementary Fig. 8 we obtain the correct definition of the intrinsic skew-scattering velocity as shown in Supplementary Fig. 22.

Similar to the side-jump velocity, the vertex correction of the skew-scattering velocity can also absorb the edge correction. We also need the modified off-diagonal velocity defined in Supplementary Fig. 20. The resultant intrinsic skew-scattering diagrams after the vertex redefinition are shown in Supplementary Fig. 23. Again, similar to the side-jump case, there are only two types of diagrams after the vertex redefinition. As a matter of fact, this "coincidence" is actually a consequence of the non-crossing approximation.

The extrinsic skew-scattering contains relatively fewer diagrams. However, to simplify them, we need to define an extrinsic skew-scattering velocity as shown in Supplementary Fig. 24. This velocity can also absorb the edge correction in the diagrams. Thus, the extrinsic skew-scattering diagrams after the vertex redefinition can be obtained accordingly, and the resultant diagrams are shown in Supplementary Fig. 25. The diagram Supplementary Fig. 25 (d) is special, as it can not be considered as a vertex modification. This diagram actually belongs to a general type of Feynman diagram with indecomposable self-energy $\Sigma_{\boldsymbol{kk'k''}} = W^{sk2}_{\boldsymbol{kk'k''}}$ as shown in Supplementary Fig. 26.

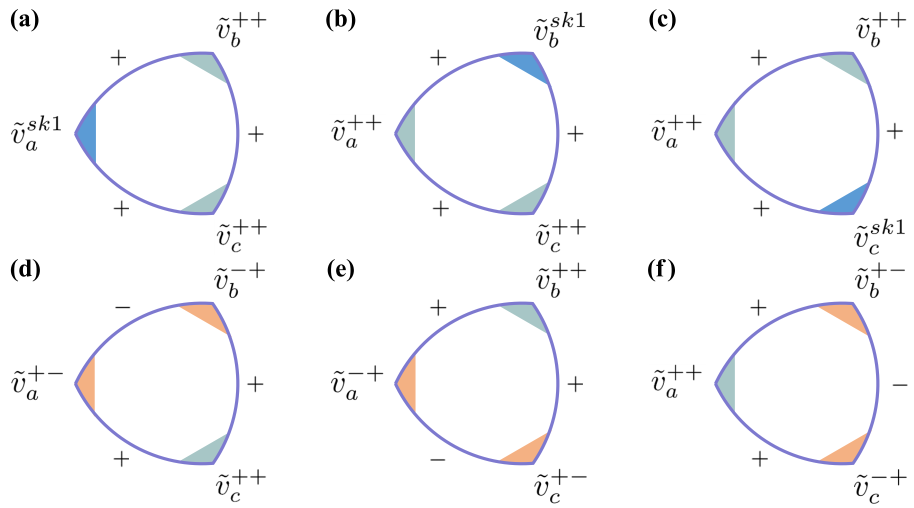

FIG. 23. Feynman diagrams of the intrinsic skew-scattering contribution after the vertex redefinition.

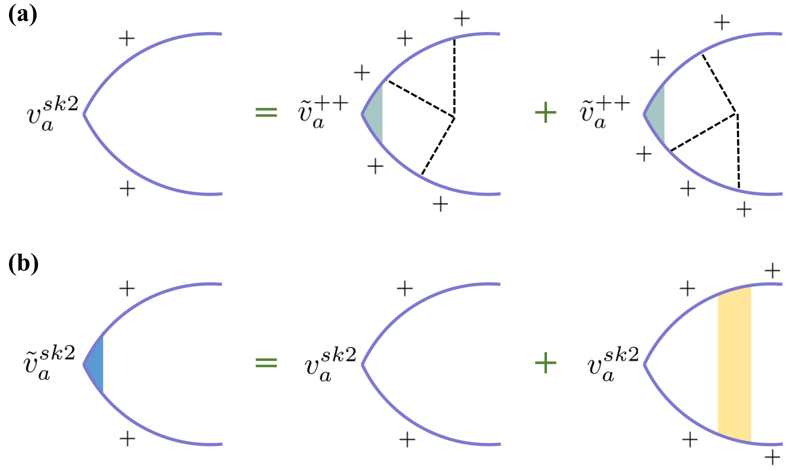

FIG. 24. Diagrammatic representation of the extrinsic skew-scattering velocity and its disorder-induced modification.

D. Two-photon diagrams

According to the analysis in Sec. Supplementary Note 4, the two-photon processes can only contribute to the nonlinear Hall conductivity (up to the leading order in $n_i V_0^2$) via the side-jump and skew-scattering mechanisms. After the vertex redefinition, the two-photon side-jump and skew-scattering diagrams can also be simplified as shown in Supplementary Fig. 27. As these diagrams are very similar with the linear response diagrams, the results are relatively simple.

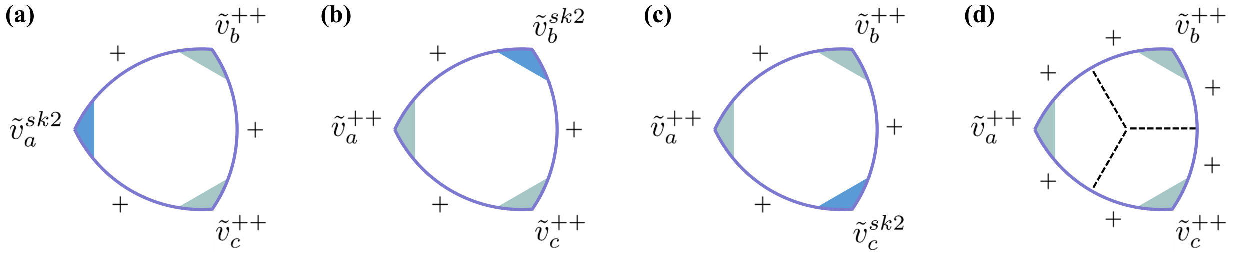

FIG. 25. Feynman diagrams of the extrinsic skew-scattering contribution after the vertex redefinition.

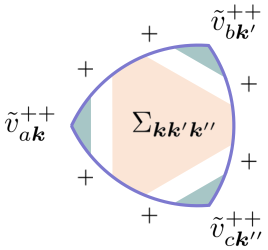

FIG. 26. A general Feynman diagram with indecomposable self-energy $\Sigma_{\boldsymbol{k}\boldsymbol{k}'\boldsymbol{k}''}$.

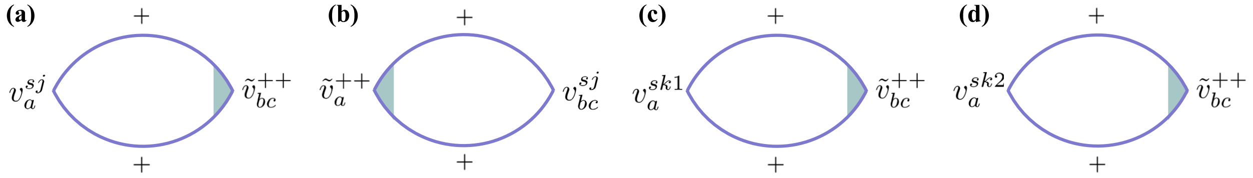

FIG. 27. Feynman diagrams of the two-photon side-jump [(a) and (b)], intrinsic skew-scattering (c), and extrinsic skew-scattering (d) contributions after the vertex redefinition.

Supplementary Note 7. PHYSICAL QUANTITIES AND USEFUL IDENTITIES

A. Intrinsic related quantities

In the calculation of the intrinsic contribution to the anomalous and nonlinear Hall conductivities, the relevant inter-band coupling processes are $v^{+-}_{a\boldsymbol{k}}v^{-+}_{b\boldsymbol{k}}$ and $v^{-+}_{a\boldsymbol{k}}v^{+-}_{b\boldsymbol{k}}$. Noting that we have $v^{+-}_{a\boldsymbol{k}}v^{-+}_{b\boldsymbol{k}} = (v^{-+}_{a\boldsymbol{k}}v^{+-}_{b\boldsymbol{k}})^*$ and the Fermi level is assumed in the upper band, thus the relevant physical quantity for the nonlinear transverse response in time-reversal symmetric systems can be defined as

$$
\begin{aligned}
I^{in}_{ab}(\boldsymbol{k}) &\equiv \mathrm{Im}(v^{+-}_{a\boldsymbol{k}}v^{-+}_{b\boldsymbol{k}}) \\
&= \frac{1}{\hbar^2 h_{\boldsymbol{k}}}\Big(h_{z\boldsymbol{k}}\partial^a_{\boldsymbol{k}}h_{x\boldsymbol{k}}\partial^b_{\boldsymbol{k}}h_{y\boldsymbol{k}} - h_{y\boldsymbol{k}}\partial^a_{\boldsymbol{k}}h_{x\boldsymbol{k}}\partial^b_{\boldsymbol{k}}h_{z\boldsymbol{k}} - h_{z\boldsymbol{k}}\partial^a_{\boldsymbol{k}}h_{y\boldsymbol{k}}\partial^b_{\boldsymbol{k}}h_{x\boldsymbol{k}} \\
&\quad + h_{x\boldsymbol{k}}\partial^a_{\boldsymbol{k}}h_{y\boldsymbol{k}}\partial^b_{\boldsymbol{k}}h_{z\boldsymbol{k}} + h_{y\boldsymbol{k}}\partial^a_{\boldsymbol{k}}h_{z\boldsymbol{k}}\partial^b_{\boldsymbol{k}}h_{x\boldsymbol{k}} - h_{x\boldsymbol{k}}\partial^a_{\boldsymbol{k}}h_{z\boldsymbol{k}}\partial^b_{\boldsymbol{k}}h_{y\boldsymbol{k}}\Big) \\
&= \frac{1}{\hbar^2 h_{\boldsymbol{k}}}\varepsilon^{efg}h_{e\boldsymbol{k}}\partial^a_{\boldsymbol{k}}h_{f\boldsymbol{k}}\partial^b_{\boldsymbol{k}}h_{g\boldsymbol{k}}.
\end{aligned}
\tag{183}
$$

Compared with Supplementary Eq. (71), we have

$$
I^{in}_{ab}(\boldsymbol{k}) = \mp 2\Omega^{ab}_{\pm}h^2_{\boldsymbol{k}}/\hbar^2. \tag{184}
$$

B. Side-jump related quantities

In the calculation of the 1st kind side-jump contribution to the anomalous and nonlinear Hall conductivities, the relevant scattering processes are

$$
\langle V^{-+}_{\boldsymbol{k}\boldsymbol{k}'}V^{++}_{\boldsymbol{k}'\boldsymbol{k}}\rangle = \frac{n_i V_0^2}{2}\Big[\sin\theta_{\boldsymbol{k}}\cos\theta_{\boldsymbol{k}'} - \cos\theta_{\boldsymbol{k}}\sin\theta_{\boldsymbol{k}'}\cos(\phi_{\boldsymbol{k}}-\phi_{\boldsymbol{k}'}) + i\sin\theta_{\boldsymbol{k}'}\sin(\phi_{\boldsymbol{k}}-\phi_{\boldsymbol{k}'})\Big], \tag{185}
$$

$$
\langle V^{++}_{\boldsymbol{k}\boldsymbol{k}'}V^{+-}_{\boldsymbol{k}'\boldsymbol{k}}\rangle = \frac{n_i V_0^2}{2}\Big[\sin\theta_{\boldsymbol{k}}\cos\theta_{\boldsymbol{k}'} - \cos\theta_{\boldsymbol{k}}\sin\theta_{\boldsymbol{k}'}\cos(\phi_{\boldsymbol{k}}-\phi_{\boldsymbol{k}'}) - i\sin\theta_{\boldsymbol{k}'}\sin(\phi_{\boldsymbol{k}}-\phi_{\boldsymbol{k}'})\Big]. \tag{186}
$$

By definition, we have $\langle V^{-+}_{\boldsymbol{k}\boldsymbol{k}'} V^{++}_{\boldsymbol{k}'\boldsymbol{k}} \rangle = \langle V^{++}_{\boldsymbol{k}\boldsymbol{k}'} V^{+-}_{\boldsymbol{k}'\boldsymbol{k}} \rangle^*$. Thus, the relevant physical quantity for the nonlinear transverse response in time-reversal symmetric systems can be defined as

$$\begin{aligned} I^{sj}_a(\boldsymbol{k},\boldsymbol{k}') &\equiv \mathrm{Im}[v^{+-}_{a\boldsymbol{k}} \langle V^{-+}_{\boldsymbol{k}\boldsymbol{k}'} V^{++}_{\boldsymbol{k}'\boldsymbol{k}} \rangle] \\ &= \frac{n_i V_0^2 h_{\boldsymbol{k}}}{2\hbar} \Big\{ \sin\theta_{\boldsymbol{k}} \big[\sin\theta_{\boldsymbol{k}} \cos\theta_{\boldsymbol{k}'} - \cos\theta_{\boldsymbol{k}} \sin\theta_{\boldsymbol{k}'} \cos(\phi_{\boldsymbol{k}} - \phi_{\boldsymbol{k}'}) \big] \partial^a_{\boldsymbol{k}} \phi_{\boldsymbol{k}} \\ &\quad - \sin\theta_{\boldsymbol{k}'} \sin(\phi_{\boldsymbol{k}} - \phi_{\boldsymbol{k}'}) \partial^a_{\boldsymbol{k}} \theta_{\boldsymbol{k}} \Big\}. \end{aligned} \tag{187}$$

Also, note that the side-jump velocity of the upper band to the leading order is defined as

$$v^{sj}_{a\boldsymbol{k}} = \int [dk'] \varpi^{(2)}_{\boldsymbol{k}\boldsymbol{k}'} \delta r^a_{\boldsymbol{k}'\boldsymbol{k}}, \tag{188}$$

with the coordinate shift

$$\delta r^a_{\boldsymbol{k}\boldsymbol{k}'} = \langle u^+_{\boldsymbol{k}} | i\partial^a_{\boldsymbol{k}} u^+_{\boldsymbol{k}} \rangle - \langle u^+_{\boldsymbol{k}'} | i\partial^a_{\boldsymbol{k}'} u^+_{\boldsymbol{k}'} \rangle - (\partial^a_{\boldsymbol{k}} + \partial^a_{\boldsymbol{k}'}) \arg\big[\langle u^+_{\boldsymbol{k}} | u^+_{\boldsymbol{k}'} \rangle\big], \tag{189}$$

and the leading Gaussian scattering rate

$$\begin{aligned} \varpi^{(2)}_{\boldsymbol{k}\boldsymbol{k}'} &= \frac{2\pi}{\hbar} \langle V^{++}_{\boldsymbol{k}'\boldsymbol{k}} V^{++}_{\boldsymbol{k}\boldsymbol{k}'} \rangle \delta(\varepsilon^+_{\boldsymbol{k}} - \varepsilon^+_{\boldsymbol{k}'}) \\ &= \frac{\pi n_i V_0^2}{\hbar} \big[1 + \cos\theta_{\boldsymbol{k}} \cos\theta_{\boldsymbol{k}'} + \sin\theta_{\boldsymbol{k}} \sin\theta_{\boldsymbol{k}'} \cos(\phi_{\boldsymbol{k}'} - \phi_{\boldsymbol{k}}) \big] \delta(\varepsilon^+_{\boldsymbol{k}} - \varepsilon^+_{\boldsymbol{k}'}). \end{aligned} \tag{190}$$

Following the similar steps in our previous work [4], one can prove that

$$v^{sj}_{a\boldsymbol{k}} = \pi \int [dk'] \Big[\frac{I^{sj}_a(\boldsymbol{k},\boldsymbol{k}')}{h_{\boldsymbol{k}}} - \frac{I^{sj}_a(\boldsymbol{k}',\boldsymbol{k})}{h_{\boldsymbol{k}'}} \Big] \delta(\varepsilon^+_{\boldsymbol{k}} - \varepsilon^+_{\boldsymbol{k}'}). \tag{191}$$

Except for the side-jump velocity, the nonlinear response also contains side-jump related quantities that have no obvious semiclassical correspondence. Thus, for convenience, we define that

$$V^{sj,0}_{a\boldsymbol{k}\boldsymbol{k}'} \equiv 2\pi \Big[\frac{I^{sj}_a(\boldsymbol{k},\boldsymbol{k}')}{\varepsilon^+_{\boldsymbol{k}} - \varepsilon^-_{-\boldsymbol{k}}} - \frac{I^{sj}_a(\boldsymbol{k}',\boldsymbol{k})}{\varepsilon^+_{\boldsymbol{k}} - \varepsilon^-_{-\boldsymbol{k}'}} \Big], \tag{192}$$

$$V^{sj,1}_{a\boldsymbol{k}\boldsymbol{k}'} \equiv \frac{\pi}{2} \Big[\frac{I^{sj}_a(\boldsymbol{k},\boldsymbol{k}')}{h^2_{\boldsymbol{k}}} - \frac{I^{sj}_a(\boldsymbol{k}',\boldsymbol{k})}{h^2_{\boldsymbol{k}'}} \Big], \tag{193}$$

$$V^{sj,2}_{a\boldsymbol{k}\boldsymbol{k}'} \equiv \frac{\pi}{2} \Big[\frac{I^{sj}_a(\boldsymbol{k},\boldsymbol{k}')}{h^2_{\boldsymbol{k}}} + \frac{I^{sj}_a(\boldsymbol{k}',\boldsymbol{k})}{h^2_{\boldsymbol{k}'}} \Big], \tag{194}$$

which indicates that

$$v^{sj}_{a\boldsymbol{k}} = \int [dk'] V^{sj,0}_{a\boldsymbol{k}\boldsymbol{k}'} \delta(\varepsilon^+_{\boldsymbol{k}} - \varepsilon^+_{\boldsymbol{k}'}). \tag{195}$$

To include the two-photon processes into the side-jump contribution, we also need to define the tensor generalization of the side-jump velocity as

$$v^{sj}_{ab\boldsymbol{k}} = \pi \int [dk'] \Big[\frac{I^{sj}_{ab}(\boldsymbol{k},\boldsymbol{k}')}{h_{\boldsymbol{k}}} - \frac{I^{sj}_{ab}(\boldsymbol{k}',\boldsymbol{k})}{h_{\boldsymbol{k}'}} \Big] \delta(\varepsilon^+_{\boldsymbol{k}} - \varepsilon^+_{\boldsymbol{k}'}), \tag{196}$$

where $I^{sj}_{ab}(\boldsymbol{k},\boldsymbol{k}') \equiv \mathrm{Im}[v^{+-}_{ab\boldsymbol{k}} \langle V^{-+}_{\boldsymbol{k}\boldsymbol{k}'} V^{++}_{\boldsymbol{k}'\boldsymbol{k}} \rangle]$.

C. Skew-scattering related quantities

In the calculation of the intrinsic skew-scattering contribution within the non-crossing approximation to the anomalous and nonlinear Hall conductivities, the relevant scattering processes are something like $\langle V^{-+}_{\boldsymbol{k}\boldsymbol{k}'} V^{++}_{\boldsymbol{k}'\boldsymbol{k}} \rangle \langle V^{+-}_{\boldsymbol{k}''\boldsymbol{k}} V^{++}_{\boldsymbol{k}\boldsymbol{k}''} \rangle$ and $\langle V^{++}_{\boldsymbol{k}\boldsymbol{k}'} V^{++}_{\boldsymbol{k}'\boldsymbol{k}''} V^{++}_{\boldsymbol{k}''\boldsymbol{k}} \rangle$ [4]. And the relevant physical quantities for the nonlinear transverse response in time-reversal symmetric systems can be defined as

$$I^{sk,1}(\boldsymbol{k},\boldsymbol{k}',\boldsymbol{k}'') \equiv \mathrm{Im}[\langle V^{-+}_{\boldsymbol{k}\boldsymbol{k}'} V^{++}_{\boldsymbol{k}'\boldsymbol{k}} \rangle \langle V^{+-}_{\boldsymbol{k}''\boldsymbol{k}} V^{++}_{\boldsymbol{k}\boldsymbol{k}''} \rangle], \tag{197}$$

$$I^{sk,2}(\boldsymbol{k},\boldsymbol{k}',\boldsymbol{k}'') \equiv \mathrm{Im}[\langle V^{++}_{\boldsymbol{k}\boldsymbol{k}'} V^{++}_{\boldsymbol{k}'\boldsymbol{k}''} V^{++}_{\boldsymbol{k}''\boldsymbol{k}} \rangle]. \tag{198}$$

Also, note that

$$[\langle V^{-+}_{\boldsymbol{kk}'} V^{++}_{\boldsymbol{k}'\boldsymbol{k}}\rangle\langle V^{+-}_{\boldsymbol{k}''\boldsymbol{k}} V^{++}_{\boldsymbol{kk}''}\rangle]^* = \langle V^{-+}_{\boldsymbol{kk}''} V^{++}_{\boldsymbol{k}''\boldsymbol{k}}\rangle\langle V^{+-}_{\boldsymbol{k}'\boldsymbol{k}} V^{++}_{\boldsymbol{kk}'}\rangle, \tag{199}$$

and

$$[\langle V^{++}_{\boldsymbol{kk}'} V^{++}_{\boldsymbol{k}'\boldsymbol{k}''} V^{++}_{\boldsymbol{k}''\boldsymbol{k}}\rangle]^* = \langle V^{++}_{\boldsymbol{kk}''} V^{++}_{\boldsymbol{k}''\boldsymbol{k}'} V^{++}_{\boldsymbol{k}'\boldsymbol{k}}\rangle, \tag{200}$$

thus we have

$$I^{sk,1}(\boldsymbol{k},\boldsymbol{k}',\boldsymbol{k}'') = -I^{sk,1}(\boldsymbol{k},\boldsymbol{k}'',\boldsymbol{k}'), \tag{201}$$
$$I^{sk,2}(\boldsymbol{k},\boldsymbol{k}',\boldsymbol{k}'') = -I^{sk,2}(\boldsymbol{k},\boldsymbol{k}'',\boldsymbol{k}'), \tag{202}$$

Similarly, the related scattering processes can be written as

$$\mathrm{Im}[\langle V^{-+}_{\boldsymbol{k}'\boldsymbol{k}''} V^{++}_{\boldsymbol{k}''\boldsymbol{k}'}\rangle\langle V^{+-}_{\boldsymbol{kk}'} V^{++}_{\boldsymbol{k}'\boldsymbol{k}}\rangle] = I^{sk,1}(\boldsymbol{k}',\boldsymbol{k}'',\boldsymbol{k}), \tag{203}$$
$$\mathrm{Im}[\langle V^{-+}_{\boldsymbol{k}'\boldsymbol{k}} V^{++}_{\boldsymbol{kk}'}\rangle\langle V^{+-}_{\boldsymbol{k}''\boldsymbol{k}'} V^{++}_{\boldsymbol{k}'\boldsymbol{k}''}\rangle] = -I^{sk,1}(\boldsymbol{k}',\boldsymbol{k}'',\boldsymbol{k}), \tag{204}$$
$$\mathrm{Im}[\langle V^{-+}_{\boldsymbol{k}''\boldsymbol{k}} V^{++}_{\boldsymbol{kk}''}\rangle\langle V^{+-}_{\boldsymbol{k}'\boldsymbol{k}''} V^{++}_{\boldsymbol{k}''\boldsymbol{k}'}\rangle] = I^{sk,1}(\boldsymbol{k}'',\boldsymbol{k},\boldsymbol{k}'), \tag{205}$$
$$\mathrm{Im}[\langle V^{-+}_{\boldsymbol{k}''\boldsymbol{k}'} V^{++}_{\boldsymbol{k}'\boldsymbol{k}''}\rangle\langle V^{+-}_{\boldsymbol{kk}''} V^{++}_{\boldsymbol{k}''\boldsymbol{k}}\rangle] = -I^{sk,1}(\boldsymbol{k}'',\boldsymbol{k},\boldsymbol{k}'). \tag{206}$$

Note that the relevant quantity

$$\begin{aligned}\varpi^{sk,1}_{\boldsymbol{kk}'} &= \frac{2\pi^2}{\hbar}\int[dk'']\Big[\frac{I^{sk,1}(\boldsymbol{k}',\boldsymbol{k}'',\boldsymbol{k})}{h_{\boldsymbol{k}'}} + \frac{I^{sk,1}(\boldsymbol{k},\boldsymbol{k}',\boldsymbol{k}'')}{h_{\boldsymbol{k}}} + \frac{I^{sk,1}(\boldsymbol{k}'',\boldsymbol{k},\boldsymbol{k}')}{h_{\boldsymbol{k}''}}\Big]\delta(\varepsilon^+_{\boldsymbol{k}} - \varepsilon^+_{\boldsymbol{k}'})\delta(\varepsilon^+_{\boldsymbol{k}} - \varepsilon^+_{\boldsymbol{k}''})\\
&= \frac{\pi^2 n_i^2 V_0^4}{2\hbar}\int[dk'']\Big(\frac{1}{h_{\boldsymbol{k}}} + \frac{1}{h_{\boldsymbol{k}'}} + \frac{1}{h_{\boldsymbol{k}''}}\Big)\Big[\sin\theta_{\boldsymbol{k}}\sin\theta_{\boldsymbol{k}'}\cos\theta_{\boldsymbol{k}''}\sin(\phi_{\boldsymbol{k}} - \phi_{\boldsymbol{k}'}) + \sin\theta_{\boldsymbol{k}'}\sin\theta_{\boldsymbol{k}''}\cos\theta_{\boldsymbol{k}}\sin(\phi_{\boldsymbol{k}'} - \phi_{\boldsymbol{k}''})\\
&\quad + \sin\theta_{\boldsymbol{k}}\sin\theta_{\boldsymbol{k}''}\cos\theta_{\boldsymbol{k}'}\sin(\phi_{\boldsymbol{k}''} - \phi_{\boldsymbol{k}})\Big]\delta(\varepsilon^+_{\boldsymbol{k}} - \varepsilon^+_{\boldsymbol{k}''})\delta(\varepsilon^+_{\boldsymbol{k}} - \varepsilon^+_{\boldsymbol{k}'})\\
&= \varpi^{(4a)}_{\boldsymbol{kk}'},\end{aligned} \tag{207}$$

$$\begin{aligned}\varpi^{sk,2}_{\boldsymbol{kk}'} &= -\frac{4\pi^2}{\hbar}\int[dk'']I_2^{sk,2}(\boldsymbol{k},\boldsymbol{k}',\boldsymbol{k}'')\delta(\varepsilon^+_{\boldsymbol{k}} - \varepsilon^+_{\boldsymbol{k}''})\delta(\varepsilon^+_{\boldsymbol{k}} - \varepsilon^+_{\boldsymbol{k}'})\\
&= \frac{\pi^2 n_i V_1^3}{\hbar}\int[dk'']\Big[\sin\theta_{\boldsymbol{k}}\sin\theta_{\boldsymbol{k}'}\cos\theta_{\boldsymbol{k}''}\sin(\phi_{\boldsymbol{k}} - \phi_{\boldsymbol{k}'}) + \sin\theta_{\boldsymbol{k}'}\sin\theta_{\boldsymbol{k}''}\cos\theta_{\boldsymbol{k}}\sin(\phi_{\boldsymbol{k}'} - \phi_{\boldsymbol{k}''})\\
&\quad + \sin\theta_{\boldsymbol{k}}\sin\theta_{\boldsymbol{k}''}\cos\theta_{\boldsymbol{k}'}\sin(\phi_{\boldsymbol{k}''} - \phi_{\boldsymbol{k}})\Big]\delta(\varepsilon^+_{\boldsymbol{k}} - \varepsilon^+_{\boldsymbol{k}''})\delta(\varepsilon^+_{\boldsymbol{k}} - \varepsilon^+_{\boldsymbol{k}'}),\\
&= \varpi^{(3a)}_{\boldsymbol{kk}'},\end{aligned} \tag{208}$$

where $\varpi^{(4a)}_{\boldsymbol{kk}'}$ is the leading Gaussian skew-scattering rate and $\varpi^{(3a)}_{\boldsymbol{kk}'}$ is the leading non-Gaussian skew-scattering rate.

The nonlinear response also contains skew-scattering related quantities that have no obvious semiclassical correspondence. Thus, for convenience, we define that

$$W^{sk,1\alpha}_{\boldsymbol{kk}'\boldsymbol{k}''} = \frac{4\pi^2}{\hbar}\Big[\frac{I^{sk,1}(\boldsymbol{k},\boldsymbol{k}',\boldsymbol{k}'')}{\varepsilon^+_{\boldsymbol{k}} - \varepsilon^-_{-\boldsymbol{k}}} + \frac{I^{sk,1}(\boldsymbol{k}',\boldsymbol{k}'',\boldsymbol{k})}{\varepsilon^+_{\boldsymbol{k}} - \varepsilon^-_{-\boldsymbol{k}'}} + \frac{I^{sk,1}(\boldsymbol{k}'',\boldsymbol{k},\boldsymbol{k}')}{\varepsilon^+_{\boldsymbol{k}} - \varepsilon^-_{-\boldsymbol{k}''}}\Big], \tag{209}$$

$$W^{sk,1\beta}_{\boldsymbol{kk}'\boldsymbol{k}''} = \frac{4\pi^2}{\hbar}\Big[\frac{I^{sk,1}(\boldsymbol{k},\boldsymbol{k}',\boldsymbol{k}'')}{(\varepsilon^+_{\boldsymbol{k}} - \varepsilon^-_{-\boldsymbol{k}})^2} + \frac{I^{sk,1}(\boldsymbol{k}',\boldsymbol{k}'',\boldsymbol{k})}{(\varepsilon^+_{\boldsymbol{k}} - \varepsilon^-_{-\boldsymbol{k}'})^2} + \frac{I^{sk,1}(\boldsymbol{k}'',\boldsymbol{k},\boldsymbol{k}')}{(\varepsilon^+_{\boldsymbol{k}} - \varepsilon^-_{-\boldsymbol{k}''})^2}\Big], \tag{210}$$

$$W^{sk,1\gamma}_{\boldsymbol{kk}'\boldsymbol{k}''} = \frac{4\pi^2}{\hbar}\Big[\frac{I^{sk,1}(\boldsymbol{k},\boldsymbol{k}',\boldsymbol{k}'')}{(\varepsilon^+_{\boldsymbol{k}} - \varepsilon^-_{-\boldsymbol{k}})^2} + \frac{I^{sk,1}(\boldsymbol{k}',\boldsymbol{k}'',\boldsymbol{k})}{(\varepsilon^+_{\boldsymbol{k}} - \varepsilon^-_{-\boldsymbol{k}'})^2} - \frac{I^{sk,1}(\boldsymbol{k}'',\boldsymbol{k},\boldsymbol{k}')}{(\varepsilon^+_{\boldsymbol{k}} - \varepsilon^-_{-\boldsymbol{k}''})^2}\Big], \tag{211}$$

$$W^{sk,2}_{\boldsymbol{kk}'\boldsymbol{k}''} = -\frac{4\pi^2}{\hbar}I^{sk,2}(\boldsymbol{k},\boldsymbol{k}',\boldsymbol{k}''), \tag{212}$$

which indicates that

$$\varpi^{sk,1}_{\boldsymbol{kk}'} = \int[dk'']W^{sk,1\alpha}_{\boldsymbol{kk}'\boldsymbol{k}''}\delta(\varepsilon^+_{\boldsymbol{k}} - \varepsilon^+_{\boldsymbol{k}''})\delta(\varepsilon^+_{\boldsymbol{k}} - \varepsilon^+_{\boldsymbol{k}'}), \tag{213}$$

$$\varpi^{sk,2}_{\boldsymbol{kk}'} = \int[dk'']W^{sk,2}_{\boldsymbol{kk}'\boldsymbol{k}''}\delta(\varepsilon^+_{\boldsymbol{k}} - \varepsilon^+_{\boldsymbol{k}''})\delta(\varepsilon^+_{\boldsymbol{k}} - \varepsilon^+_{\boldsymbol{k}'}). \tag{214}$$

D. Identities about the Green's functions

A common approximation of the Green's functions' product in the weak-disorder limit is

$$\tilde{G}_l^R(\varepsilon)\tilde{G}_l^A(\varepsilon) \simeq \frac{2\pi}{\hbar}\tau_l\delta(\varepsilon-\varepsilon_l), \tag{215}$$

where $l=(\eta,\boldsymbol{k})$ is a combined label. Here we consider a general products of the form $[\tilde{G}_l^R(\varepsilon)]^{m+1}[\tilde{G}_l^A(\varepsilon)]^{n+1}$ with $m,n\in\mathbb{Z}$ and $m,n\geq 0$. Based on the identity with $m=n=0$, one can estimate that $[\tilde{G}_l^R(\varepsilon)]^{m+1}[\tilde{G}_l^A(\varepsilon)]^{n+1}\propto\delta(\varepsilon-\varepsilon_l)$. Thus, we can assume the general expression as

$$[\tilde{G}_l^R(\varepsilon)]^{m+1}[\tilde{G}_l^A(\varepsilon)]^{n+1} = P_l(m,n)\delta(\varepsilon-\varepsilon_l), \tag{216}$$

where $P_l(m,n)$ is a l-related polynomial of m and n. By integrating over ε on both sides of the above equation, we obtain that

$$\begin{aligned} P_l(m,n) &= \int_{-\infty}^{\infty} d\varepsilon \frac{1}{(\varepsilon-\varepsilon_l+i\Gamma_l)^{m+1}(\varepsilon-\varepsilon_l-i\Gamma_l)^{n+1}} \\ &= 2\pi i^{n-m}\frac{(m+n)!}{m!n!}\Big(\frac{\tau_l}{\hbar}\Big)^{n+m+1}. \end{aligned} \tag{217}$$

Thus, we have that

$$[\tilde{G}_l^R(\varepsilon)]^{m+1}[\tilde{G}_l^A(\varepsilon)]^{n+1} \simeq 2\pi i^{n-m}\frac{(m+n)!}{m!n!}\Big(\frac{\tau_l}{\hbar}\Big)^{n+m+1}\delta(\varepsilon-\varepsilon_l), \tag{218}$$

which reproduces the simplest identity at $m=n=0$.

Now we turn to evaluate the products such as $\tilde{G}_l^R(\varepsilon)\tilde{G}_l^A(\varepsilon-\hbar\omega_\alpha)$, $\tilde{G}_l^R(\varepsilon+\hbar\omega_\alpha)\tilde{G}_l^A(\varepsilon)$ and $\tilde{G}_l^R(\varepsilon+\hbar\omega_\alpha)\tilde{G}_l^A(\varepsilon-\hbar\omega_\beta)$, where $\alpha,\beta\in(a,b,c,\cdots)$. Based on the identities we have obtained, we can obtain that

$$\begin{aligned} \tilde{G}_l^R(\varepsilon)\tilde{G}_l^A(\varepsilon-\hbar\omega_\alpha) &= \frac{1}{(\varepsilon-\varepsilon_l+i\Gamma_l)(\varepsilon-\varepsilon_l-\hbar\omega_\alpha-i\Gamma_l)} \\ &= \tilde{G}_l^R(\varepsilon)\Big\{\tilde{G}_l^A(\varepsilon)+\hbar\omega_\alpha[\tilde{G}_l^A(\varepsilon)]^2+\hbar^2\omega_\alpha^2[\tilde{G}_l^A(\varepsilon)]^3+\hbar^3\omega_\alpha^3[\tilde{G}_l^A(\varepsilon)]^4+\cdots\Big\} \\ &\simeq \frac{2\pi}{\hbar}\tau_l\Big[1+i\omega_\alpha\tau_l+i^2\omega_\alpha^2\tau_l^2+i^3\omega_\alpha^3\tau_l^3+\cdots\Big]\delta(\varepsilon-\varepsilon_l) \\ &= \frac{2\pi}{\hbar}\frac{\tau_l}{1-i\omega_\alpha\tau_l}\delta(\varepsilon-\varepsilon_l), \end{aligned} \tag{219}$$

$$\tilde{G}_l^R(\varepsilon+\hbar\omega_\alpha)\tilde{G}_l^A(\varepsilon) = \frac{2\pi}{\hbar}\frac{\tau_l}{1-i\omega_\alpha\tau_l}\delta(\varepsilon-\varepsilon_l), \tag{220}$$

and similarly

$$\begin{aligned} \tilde{G}_l^R(\varepsilon+\hbar\omega_\alpha)\tilde{G}_l^A(\varepsilon-\hbar\omega_\beta) &= \frac{1}{(\varepsilon-\varepsilon_l+\hbar\omega_\alpha+i\Gamma_l)(\varepsilon-\varepsilon_l-\hbar\omega_\beta-i\Gamma_l)} \\ &= \Big\{\tilde{G}_l^R(\varepsilon)-\hbar\omega_\alpha[\tilde{G}_l^R(\varepsilon)]^2+\hbar^2\omega_\alpha^2[\tilde{G}_l^R(\varepsilon)]^3-\hbar^3\omega_\alpha^3[\tilde{G}_l^R(\varepsilon)]^4+\cdots\Big\} \\ &\quad\times\Big\{\tilde{G}_l^A(\varepsilon)+\hbar\omega_\beta[\tilde{G}_l^A(\varepsilon)]^2+\hbar^2\omega_\beta^2[\tilde{G}_l^A(\varepsilon)]^3+\hbar^3\omega_\beta^3[\tilde{G}_l^A(\varepsilon)]^4+\cdots\Big\} \\ &\simeq \frac{2\pi}{\hbar}\tau_l\Big[1+i(\omega_\alpha+\omega_\beta)\tau_l+i^2(\omega_\alpha+\omega_\beta)^2\tau_l^2+i^3(\omega_\alpha+\omega_\beta)^3\tau_l^3+\cdots\Big]\delta(\varepsilon-\varepsilon_l) \\ &= \frac{2\pi}{\hbar}\frac{\tau_l}{1-i(\omega_\alpha+\omega_\beta)\tau_l}\delta(\varepsilon-\varepsilon_l), \end{aligned} \tag{221}$$

where we have assumed $\hbar\omega_\alpha\ll\varepsilon$. A further generalization is the multiple products such as $\tilde{G}_l^R(\varepsilon+\hbar\omega_\alpha)\tilde{G}_l^R(\varepsilon)\tilde{G}_l^A(\varepsilon-$

$\hbar\omega_\beta)$ and $\tilde{G}_l^R(\varepsilon+\hbar\omega_\alpha)\tilde{G}_l^A(\varepsilon)\tilde{G}_l^A(\varepsilon-\hbar\omega_\beta)$ with $\alpha,\beta\in(a,b,c)$, for which we have

$$\begin{aligned}\tilde{G}_l^R(\varepsilon+\hbar\omega_\alpha)\tilde{G}_l^R(\varepsilon)\tilde{G}_l^A(\varepsilon-\hbar\omega_\beta) &= \frac{1}{(\varepsilon-\varepsilon_l+\hbar\omega_\alpha+i\Gamma_l)(\varepsilon-\varepsilon_l+i\Gamma_l)(\varepsilon-\varepsilon_l-\hbar\omega_\beta-i\Gamma_l)}\\ &= \Big\{\tilde{G}_l^R(\varepsilon)-\hbar\omega_\alpha[\tilde{G}_l^R(\varepsilon)]^2+\hbar^2\omega_\alpha^2[\tilde{G}_l^R(\varepsilon)]^3-\hbar^3\omega_\alpha^3[\tilde{G}_l^R(\varepsilon)]^4+\cdots\Big\}\tilde{G}_l^R(\varepsilon)\\ &\quad\times\Big\{\tilde{G}_l^A(\varepsilon)+\hbar\omega_\beta[\tilde{G}_l^A(\varepsilon)]^2+\hbar^2\omega_\beta^2[\tilde{G}_l^A(\varepsilon)]^3+\hbar^3\omega_\beta^3[\tilde{G}_l^A(\varepsilon)]^4+\cdots\Big\}\\ &\simeq -\frac{i2\pi}{\hbar^2}\tau_l^2\Big[1+i(\omega_\alpha+\omega_\beta)\tau_l+i^2(\omega_\alpha+\omega_\beta)^2\tau_l^2+i^3(\omega_\alpha+\omega_\beta)^3\tau_l^3+\cdots\Big]\\ &\quad\times\Big[1+i\omega_\beta\tau_l+i^2\omega_\beta^2\tau_l^2+i^3\omega_\beta^3\tau_l^3+\cdots\Big]\delta(\varepsilon-\varepsilon_l)\\ &= -\frac{i2\pi}{\hbar^2}\frac{\tau_l^2}{(1-i\omega_\beta\tau_l)[1-i(\omega_\alpha+\omega_\beta)\tau_l]}\delta(\varepsilon-\varepsilon_l),\end{aligned}\tag{222}$$

and similarly

$$\tilde{G}_l^R(\varepsilon+\hbar\omega_\alpha)\tilde{G}_l^A(\varepsilon)\tilde{G}_l^A(\varepsilon-\hbar\omega_\beta)=\frac{i2\pi}{\hbar^2}\frac{\tau_l^2}{(1-i\omega_\alpha\tau_l)[1-i(\omega_\alpha+\omega_\beta)\tau_l]}\delta(\varepsilon-\varepsilon_l).\tag{223}$$

Supplementary Note 8. 2D TILTED DIRAC MODEL

Now we apply the above diagrams to the 2D tilted massive Dirac model to calculate the nonlinear Hall conductivity in the presence of disorder scattering. The model gives the symmetry-allowed minimal description of the nonlinear Hall effect and can serve as a building block in realistic band structures [2, 13]

$$\hat{\mathcal{H}}_d = tk_x+v(k_x\sigma_x+k_y\sigma_y)+m\sigma_z,\tag{224}$$

where t, v, and m are model parameters. t tilts the Dirac cone along the x direction. The time reversal of the above model gives

$$\hat{\mathcal{T}}:\ \hat{\mathcal{H}}_d(t,v,m)\Rightarrow\hat{\mathcal{H}}_d(-t,v,-m),\tag{225}$$

which represents another Dirac fermion with opposite mass and tilt. For the disorder part, we also consider the model of randomly located δ-function scatters with both Gaussian and non-Gaussian correlations (see Sec. Supplementary Note 2 D). Because the Hamiltonian of the 2D tilted Dirac model has only linear $\boldsymbol{k}$-dependent terms, thus only triangular diagrams contribute to its nonlinear Hall conductivity. And we do not need to calculate the ω-dependent part of the vertex and edge corrections either.

A. Born approximation

1. Self-energy

The effect of disorder on the retarded and advanced Green's functions is captured by the Born approximation as

$$G_l^{R/A}(\varepsilon)\to\tilde{G}_l^{R/A}(\varepsilon)=\frac{1}{\varepsilon-\varepsilon_l\pm i\Gamma_l}.\tag{226}$$

Here $G_l^R(\varepsilon)$ and $G_l^A(\varepsilon)$ are the disorder-free Green's functions in the chiral Bloch eigenstate basis. $\tilde{G}_l^R(\varepsilon)$ and $\tilde{G}_l^A(\varepsilon)$ are the disorder-averaged Green's functions with

$$\begin{aligned}\Gamma_l &= \mp\mathrm{Im}\big(\Sigma_l^{R/A}\big)\\ &= \mp\mathrm{Im}\sum_{l'}\frac{\langle V_{ll'}V_{l'l}\rangle}{\varepsilon-\varepsilon_{l'}\pm i\delta}\\ &= \pi\sum_{l'}\langle V_{ll'}V_{l'l}\rangle\delta(\varepsilon-\varepsilon_{l'})\end{aligned}\tag{227}$$

with $V_{ll'} = \langle l|\hat{V}_{imp}|l'\rangle$. Assuming that $t < v$ and the Fermi level lies in the upper band, we have

$$\begin{aligned}
\Gamma^+_{\boldsymbol{k}} &= \frac{\pi n_i V_0^2}{2}\int \frac{d^2k'}{(2\pi)^2}\big[1+\cos\theta\cos\theta'+\sin\theta\sin\theta'\cos(\phi'-\phi)\big]\delta(\varepsilon-\varepsilon^+_{\boldsymbol{k}'}) \\
&\simeq \frac{n_i V_0^2}{8\pi}\int_0^\infty k'dk'\int_0^{2\pi} d\phi'\big[1+\cos\theta\cos\theta'+\sin\theta\sin\theta'\cos(\phi'-\phi)\big]\Big[\delta(\varepsilon-\varepsilon^0_{\boldsymbol{k}'})+tk'\cos\phi'\delta'(\varepsilon-\varepsilon^0_{\boldsymbol{k}'})\Big] \\
&= \Gamma_0+\Gamma_x\sin\theta\cos\phi+\Gamma_z\cos\theta, \qquad (228)\\
\Gamma^-_{\boldsymbol{k}} &= \frac{\pi n_i V_0^2}{2}\int \frac{d^2k'}{(2\pi)^2}\big[1-\cos\theta\cos\theta'-\sin\theta\sin\theta'\cos(\phi'-\phi)\big]\delta(\varepsilon-\varepsilon^+_{\boldsymbol{k}'}) \\
&\simeq \frac{n_i V_0^2}{8\pi}\int_0^\infty k'dk'\int_0^{2\pi} d\phi'\big[1-\cos\theta\cos\theta'-\sin\theta\sin\theta'\cos(\phi'-\phi)\big]\Big[\delta(\varepsilon-\varepsilon^0_{\boldsymbol{k}'})+tk'\cos\phi'\delta'(\varepsilon-\varepsilon^0_{\boldsymbol{k}'})\Big] \\
&= \Gamma_0-\Gamma_x\sin\theta\cos\phi-\Gamma_z\cos\theta, \qquad (229)
\end{aligned}$$

with

$$\Gamma_0 = \frac{n_i V_0^2}{4v^2}\varepsilon, \qquad \Gamma_x = -\frac{t}{v}\Gamma_0, \qquad \Gamma_z = \frac{m}{\varepsilon}\Gamma_0, \qquad (230)$$

where we have considered the $t \ll v$ limit and expanded the results up to the linear order in t. Also note that, for a general function $F(t)$, we have

$$\lim_{t\to 0} F(t) \simeq F(0)+tF'(0), \qquad \lim_{t\to 0}\frac{1}{F(t)} \simeq \frac{1}{F(0)} - t\frac{F'(0)}{F^2(0)} \qquad (231)$$

with $F'(t) = \partial F(t)/\partial t$. Thus the corresponding scattering time $\tau^\pm_{\boldsymbol{k}}$ up to the linear order in t can be found as

$$\begin{aligned}
\tau^+_{\boldsymbol{k}} &= \frac{2\hbar v^2}{n_i V_0^2}\Big[\frac{1}{\varepsilon+m\cos\theta}+\frac{t}{v}\frac{\varepsilon\sin\theta\cos\phi}{(\varepsilon+m\cos\theta)^2}\Big], \qquad (232)\\
\tau^-_{\boldsymbol{k}} &= \frac{2\hbar v^2}{n_i V_0^2}\Big[\frac{1}{\varepsilon-m\cos\theta}-\frac{t}{v}\frac{\varepsilon\sin\theta\cos\phi}{(\varepsilon-m\cos\theta)^2}\Big]. \qquad (233)
\end{aligned}$$

It is worth noting that the elastic scattering time has an angular dependence that is proportional to $\cos\phi$.

2. *Vertex correction*

The effect of disorder on the retarded-advanced vertex in the chiral basis is captured by the ladder diagrams. The diagonal component is given as

$$\begin{aligned}
\tilde{v}^{++}_{a\boldsymbol{k}} &= v^{++}_{a\boldsymbol{k}} + \int [dk']\langle V^{++}_{\boldsymbol{k}\boldsymbol{k}'}V^{++}_{\boldsymbol{k}'\boldsymbol{k}}\rangle \tilde{G}^R_{+\boldsymbol{k}'}(\varepsilon^+_1)\tilde{v}^{++}_{a\boldsymbol{k}'}\tilde{G}^A_{+\boldsymbol{k}'}(\varepsilon) \\
&= v^{++}_{a\boldsymbol{k}} + \frac{2\pi}{\hbar}\int [dk']\frac{\tau^+_{\boldsymbol{k}'}}{1-i\omega\tau^+_{\boldsymbol{k}'}}\langle V^{++}_{\boldsymbol{k}\boldsymbol{k}'}V^{++}_{\boldsymbol{k}'\boldsymbol{k}}\rangle \tilde{v}^{++}_{a\boldsymbol{k}'}\delta(\varepsilon-\varepsilon^+_{\boldsymbol{k}'}), \qquad (234)
\end{aligned}$$

which is also ω-dependent. For the 2D tilted Dirac model with the Fermi level in the upper band, by multiplying $\delta(\varepsilon-\varepsilon^+_{\boldsymbol{k}})$ on both sides, the above equation can be rewritten as

$$\tilde{v}^{++}_{a\boldsymbol{k}} = v^{++}_{a\boldsymbol{k}} + \frac{2\pi}{\hbar}\int [dk']\frac{\tau^+_{\boldsymbol{k}'}}{1-i\omega\tau^+_{\boldsymbol{k}'}}\langle V^{++}_{\boldsymbol{k}\boldsymbol{k}'}V^{++}_{\boldsymbol{k}'\boldsymbol{k}}\rangle \tilde{v}^{++}_{a\boldsymbol{k}'}\delta(\varepsilon^+_{\boldsymbol{k}}-\varepsilon^+_{\boldsymbol{k}'}). \qquad (235)$$

Note that the bare diagonal velocity of the the upper band is anisotropic. Consider the $t \ll v$ limit and decompose the diagonal velocities into the t-dependent and t-independent part, we have

$$\begin{aligned}
v^{++}_{a\boldsymbol{k}} &= \frac{v}{\hbar}\Big[I^{(0)}_{a\boldsymbol{k}}+\frac{t}{v}I^{(1)}_{a\boldsymbol{k}}\Big], \qquad (236)\\
\tilde{v}^{++}_{a\boldsymbol{k}} &= \frac{v}{\hbar}\Big[J^{(0)}_{a\boldsymbol{k}}+\frac{t}{v}J^{(1)}_{a\boldsymbol{k}}\Big], \qquad (237)
\end{aligned}$$

where $I^{(i)}_{\boldsymbol{k}}$ and $J^{(i)}_{\boldsymbol{k}}$ are t-independent functions. Accordingly, the equation of $J^{(0)}_{a\boldsymbol{k}}$ can be found as

$$J^{(0)}_{a\boldsymbol{k}}(\omega) = I^{(0)}_{a\boldsymbol{k}} + \frac{2\pi}{\hbar}\int [dk']\frac{\tau^0_{\boldsymbol{k}'}}{1-i\omega\tau^0_{\boldsymbol{k}'}}\langle V^{++}_{\boldsymbol{k}\boldsymbol{k}'}V^{++}_{\boldsymbol{k}'\boldsymbol{k}}\rangle J^{(0)}_{a\boldsymbol{k}'}(\omega)\delta(\varepsilon^0_{\boldsymbol{k}}-\varepsilon^0_{\boldsymbol{k}'}). \qquad (238)$$

For $v^{++}_{x\boldsymbol{k}}$, we have $I^{(0)}_{x\boldsymbol{k}} = \sin\theta\cos\phi$ and $I^{(1)}_{x\boldsymbol{k}} = 1$, thus the above equation can be solved as

$$J^{(0)}_{x\boldsymbol{k}}(\omega) = \frac{2(1 - i\omega\tau^0_{\boldsymbol{k}})(1 + \cos^2\theta)}{1 + 3\cos^2\theta - i2\omega\tau^0_{\boldsymbol{k}}(1 + \cos^2\theta)} \sin\theta\cos\phi. \tag{239}$$

According to the expression of $J^{(0)}_{x\boldsymbol{k}}$, we can obtain the equation of $J^{(1)}_{x\boldsymbol{k}}$ as

$$J^{(1)}_{x\boldsymbol{k}}(\omega) = \alpha_{x\boldsymbol{k}}(\omega) + \beta_{x\boldsymbol{k}}(\omega)\cos^2\phi + \frac{2\pi}{\hbar}\int [dk'] \frac{\tau^0_{\boldsymbol{k}'}}{1 - i\omega\tau^0_{\boldsymbol{k}'}} \langle V^{++}_{\boldsymbol{k}\boldsymbol{k}'} V^{++}_{\boldsymbol{k}'\boldsymbol{k}}\rangle J^{(1)}_{x\boldsymbol{k}'}(\omega)\delta(\varepsilon^0_{\boldsymbol{k}} - \varepsilon^0_{\boldsymbol{k}'}). \tag{240}$$

Here $\alpha_{x\boldsymbol{k}'}$ and $\beta_{x\boldsymbol{k}'}$ are functions of k and ω, which are finite at zero frequency as

$$\alpha_{x\boldsymbol{k}}(0) = -\frac{4\sin^2\theta\cos^2\theta}{(1 + 3\cos^2\theta)^2}, \qquad \beta_{x\boldsymbol{k}}(0) = \frac{8\sin^2\theta\cos^2\theta}{(1 + 3\cos^2\theta)^2}. \tag{241}$$

The above equation can be solved as

$$J^{(1)}_{x\boldsymbol{k}}(\omega) = \beta_{x\boldsymbol{k}}(\omega)\cos^2\phi - \frac{2(1 - i\omega\tau^0_{\boldsymbol{k}})\alpha_{x\boldsymbol{k}}(\omega) + \beta_{x\boldsymbol{k}}(\omega)}{i2\omega\tau^0_{\boldsymbol{k}}}, \tag{242}$$

whose real part is finite in the zero-frequency limit as

$$\lim_{\omega\to 0} \mathrm{Re}[J^{(1)}_{x\boldsymbol{k}}(\omega)] = 1 + \frac{\sin^2\theta\cos^2\theta}{1 + 3\cos^2\theta} + \frac{8\sin^2\theta\cos^2\theta\cos^2\phi}{(1 + 3\cos^2\theta)^2}. \tag{243}$$

Thus, we obtain the modified diagonal velocity along the x direction in the dc limit as

$$\tilde{v}^{++}_{x\boldsymbol{k}} = \frac{v}{\hbar}\Big\{\frac{2(1 + \cos^2\theta)}{1 + 3\cos^2\theta}\sin\theta\cos\phi + \frac{t}{v}\Big[1 + \frac{\sin^2\theta\cos^2\theta}{1 + 3\cos^2\theta} + \frac{8\sin^2\theta\cos^2\theta\cos^2\phi}{(1 + 3\cos^2\theta)^2}\Big]\Big\}. \tag{244}$$

For $v^{++}_{y\boldsymbol{k}}$, we have $I^{(0)}_{y\boldsymbol{k}} = \sin\theta\sin\phi$ and $I^{(1)}_{y\boldsymbol{k}} = 0$, thus the first equation can be solved as

$$J^{(0)}_{y\boldsymbol{k}}(\omega) = \frac{2(1 - i\omega\tau^0_{\boldsymbol{k}})(1 + \cos^2\theta)}{1 + 3\cos^2\theta - i2\omega\tau^0_{\boldsymbol{k}}(1 + \cos^2\theta)} \sin\theta\sin\phi, \tag{245}$$

and the second equation then becomes

$$J^{(1)}_{y\boldsymbol{k}}(\omega) = \alpha_{y\boldsymbol{k}}(\omega) + \beta_{y\boldsymbol{k}}(\omega)\sin\phi\cos\phi + \frac{2\pi}{\hbar}\int [dk'] \frac{\tau^0_{\boldsymbol{k}'}}{1 - i\omega\tau^0_{\boldsymbol{k}'}} \langle V^{++}_{\boldsymbol{k}\boldsymbol{k}'} V^{++}_{\boldsymbol{k}'\boldsymbol{k}}\rangle J^{(1)}_{y\boldsymbol{k}'}\delta(\varepsilon^0_{\boldsymbol{k}} - \varepsilon^0_{\boldsymbol{k}'}), \tag{246}$$

where

$$\alpha_{y\boldsymbol{k}}(0) = 0, \qquad \beta_{y\boldsymbol{k}}(0) = \frac{8\sin^2\theta\cos^2\theta}{(1 + 3\cos^2\theta)^2}. \tag{247}$$

The above equation can be solved as

$$J^{(1)}_{y\boldsymbol{k}} = \beta_{y\boldsymbol{k}}(\omega)\sin\phi\cos\phi, \tag{248}$$

whose real part is finite in the dc limit as

$$\lim_{\omega\to 0} \mathrm{Re}[J^{(1)}_{y\boldsymbol{k}}(\omega)] = \frac{8\sin^2\theta\cos^2\theta\sin\phi\cos\phi}{(1 + 3\cos^2\theta)^2}. \tag{249}$$

Thus, the modified diagonal velocity along the y direction is

$$\tilde{v}^{++}_{y\boldsymbol{k}} = \frac{v}{\hbar}\Big[\frac{2(1 + \cos^2\theta)}{1 + 3\cos^2\theta}\sin\theta\sin\phi + \frac{t}{v}\frac{8\sin^2\theta\cos^2\theta\sin\phi\cos\phi}{(1 + 3\cos^2\theta)^2}\Big]. \tag{250}$$

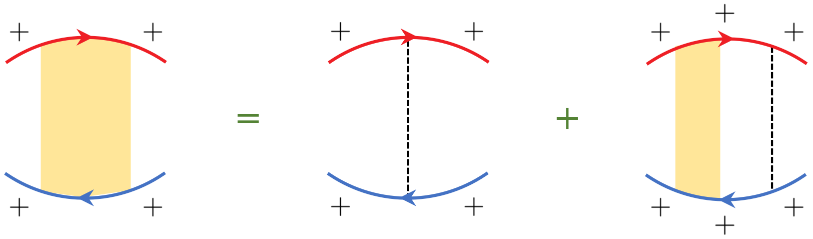

FIG. 28. Diagrammatic representation of the diffuson equation. The red and blue lines represent the retarded and advanced Green's functions, respectively.

3. Edge corrections

In the chiral basis, there are two types of corrections: vertex and edge corrections. To consider these two corrections in a unified frame, we consider the corrections in terms of diffuson. According to Supplementary Fig. 28, the diffuson $\mathcal{D}_{\boldsymbol{k}\boldsymbol{k}'}(\varepsilon,\omega)$ satisfies the equation

$$\mathcal{D}_{\boldsymbol{k}\boldsymbol{k}'}(\varepsilon,\omega)=\langle V^{++}_{\boldsymbol{k}\boldsymbol{k}'}V^{++}_{\boldsymbol{k}'\boldsymbol{k}}\rangle+\int[dk'']\mathcal{D}_{\boldsymbol{k}\boldsymbol{k}''}(\varepsilon,\omega)\langle V^{++}_{\boldsymbol{k}'',\boldsymbol{k}'}V^{++}_{\boldsymbol{k}',\boldsymbol{k}''}\rangle\tilde{G}^R_{+\boldsymbol{k}''}(\varepsilon^+_1)\tilde{G}^A_{+\boldsymbol{k}''}(\varepsilon). \tag{251}$$

Multiply $\delta(\varepsilon-\varepsilon^+_{\boldsymbol{k}'})$ on both sides of the above equation, we obtain that

$$\begin{aligned}\mathcal{D}_{\boldsymbol{k}\boldsymbol{k}'}(\varepsilon^+_{\boldsymbol{k}'},\omega)=&\frac{n_iV_0^2}{2}\left[1+\cos\theta\cos\theta'+\sin\theta\sin\theta'\cos(\phi'-\phi)\right]\\&+\frac{1}{2\pi\hbar}\int d^2k''\frac{\tau^+_{\boldsymbol{k}''}}{1-i\omega\tau^+_{\boldsymbol{k}''}}\langle V^{++}_{\boldsymbol{k}''\boldsymbol{k}'}V^{++}_{\boldsymbol{k}'\boldsymbol{k}''}\rangle\mathcal{D}_{\boldsymbol{k}\boldsymbol{k}''}(\varepsilon^+_{\boldsymbol{k}''},\omega)\delta(\varepsilon^+_{\boldsymbol{k}'}-\varepsilon^+_{\boldsymbol{k}''}).\end{aligned} \tag{252}$$

According to the k-dependence, the diffuson can be decomposed into four parts as

$$\mathcal{D}_{\boldsymbol{k}\boldsymbol{k}'}(\varepsilon^+_{\boldsymbol{k}'},\omega)=\frac{n_iV_0^2}{2}\left[\tilde{\mathcal{D}}_{0\boldsymbol{k}'}(\omega)+\tilde{\mathcal{D}}_{x\boldsymbol{k}'}(\omega)\sin\theta\cos\phi+\tilde{\mathcal{D}}_{y\boldsymbol{k}'}(\omega)\sin\theta\sin\phi+\tilde{\mathcal{D}}_{z\boldsymbol{k}'}(\omega)\cos\theta\right]. \tag{253}$$

Considering the $t\ll v$ limit, we can decompose $\tilde{\mathcal{D}}_{\mu\boldsymbol{k}}(\omega)$ into the t-dependent and t-independent parts as

$$\tilde{\mathcal{D}}_{\mu\boldsymbol{k}'}(\omega)=\tilde{\mathcal{D}}^{(0)}_{\mu\boldsymbol{k}'}(\omega)+\frac{t}{v}\tilde{\mathcal{D}}^{(1)}_{\mu\boldsymbol{k}'}(\omega). \tag{254}$$

Accordingly, the equations of $\tilde{\mathcal{D}}^{(0)}_{\mu\boldsymbol{k}'}$ can be written as

$$\tilde{\mathcal{D}}^{(0)}_{0\boldsymbol{k}'}(\omega)=1+\frac{1}{2\pi\hbar}\int d^2k''\frac{\tau^0_{\boldsymbol{k}''}}{1-i\omega\tau^0_{\boldsymbol{k}''}}\langle V^{++}_{\boldsymbol{k}''\boldsymbol{k}'}V^{++}_{\boldsymbol{k}'\boldsymbol{k}''}\rangle\tilde{\mathcal{D}}^{(0)}_{0\boldsymbol{k}''}(\omega)\delta(\varepsilon^0_{\boldsymbol{k}'}-\varepsilon^0_{\boldsymbol{k}''}), \tag{255}$$

$$\tilde{\mathcal{D}}^{(0)}_{x\boldsymbol{k}'}(\omega)=\sin\theta'\cos\phi'+\frac{1}{2\pi\hbar}\int d^2k''\frac{\tau^0_{\boldsymbol{k}''}}{1-i\omega\tau^0_{\boldsymbol{k}''}}\langle V^{++}_{\boldsymbol{k}''\boldsymbol{k}'}V^{++}_{\boldsymbol{k}'\boldsymbol{k}''}\rangle\tilde{\mathcal{D}}^{(0)}_{x\boldsymbol{k}''}(\omega)\delta(\varepsilon^0_{\boldsymbol{k}'}-\varepsilon^0_{\boldsymbol{k}''}), \tag{256}$$

$$\tilde{\mathcal{D}}^{(0)}_{y\boldsymbol{k}'}(\omega)=\sin\theta'\sin\phi'+\frac{1}{2\pi\hbar}\int d^2k''\frac{\tau^0_{\boldsymbol{k}''}}{1-i\omega\tau^0_{\boldsymbol{k}''}}\langle V^{++}_{\boldsymbol{k}''\boldsymbol{k}'}V^{++}_{\boldsymbol{k}'\boldsymbol{k}''}\rangle\tilde{\mathcal{D}}^{(0)}_{y\boldsymbol{k}''}(\omega)\delta(\varepsilon^0_{\boldsymbol{k}'}-\varepsilon^0_{\boldsymbol{k}''}), \tag{257}$$

$$\tilde{\mathcal{D}}^{(0)}_{z\boldsymbol{k}'}(\omega)=\cos\theta'+\frac{1}{2\pi\hbar}\int d^2k''\frac{\tau^0_{\boldsymbol{k}''}}{1-i\omega\tau^0_{\boldsymbol{k}''}}\langle V^{++}_{\boldsymbol{k}''\boldsymbol{k}'}V^{++}_{\boldsymbol{k}'\boldsymbol{k}''}\rangle\tilde{\mathcal{D}}^{(0)}_{z\boldsymbol{k}''}(\omega)\delta(\varepsilon^0_{\boldsymbol{k}'}-\varepsilon^0_{\boldsymbol{k}''}). \tag{258}$$

which can be solved as

$$\tilde{\mathcal{D}}^{(0)}_{0\boldsymbol{k}'}(\omega)=-\frac{1-i\omega\tau^0_{\boldsymbol{k}'}}{i\omega\tau^0_{\boldsymbol{k}'}}, \tag{259}$$

$$\tilde{\mathcal{D}}^{(0)}_{x\boldsymbol{k}'}(\omega)=\frac{2(1-i\omega\tau^0_{\boldsymbol{k}'})(1+\cos^2\theta')}{1+3\cos^2\theta'-i2\omega\tau^0_{\boldsymbol{k}'}(1+\cos^2\theta')}\sin\theta'\cos\phi', \tag{260}$$

$$\tilde{\mathcal{D}}^{(0)}_{y\boldsymbol{k}'}(\omega)=\frac{2(1-i\omega\tau^0_{\boldsymbol{k}'})(1+\cos^2\theta')}{1+3\cos^2\theta'-i2\omega\tau^0_{\boldsymbol{k}'}(1+\cos^2\theta')}\sin\theta'\sin\phi', \tag{261}$$

$$\tilde{\mathcal{D}}^{(0)}_{z\boldsymbol{k}'}(\omega)=-\frac{1-i\omega\tau^0_{\boldsymbol{k}'}}{i\omega\tau^0_{\boldsymbol{k}'}}\cos\theta'. \tag{262}$$

It is worth noting that the singular ω-dependence in $\tilde{\mathcal{D}}^{(0)}_{0\boldsymbol{k}'}(\omega)$ and $\tilde{\mathcal{D}}^{(0)}_{z\boldsymbol{k}'}(\omega)$ as $\omega\tau\to 0$ is actually a consequence of the uniform limit ($\boldsymbol{q}\to 0$). As we shall see, the nonlinear conductance remains finite in the dc limit.

Based on these results, the equations of $\tilde{\mathcal{D}}^{(1)}_{\mu\boldsymbol{k}'}(\omega)$ can be found as

$$\tilde{\mathcal{D}}^{(1)}_{0\boldsymbol{k}'}(\omega)=\frac{1}{2\pi\hbar}\int d^2k''\frac{\tau^0_{\boldsymbol{k}''}}{1-i\omega\tau^0_{\boldsymbol{k}''}}\langle V^{++}_{\boldsymbol{k}''\boldsymbol{k}'}V^{++}_{\boldsymbol{k}'\boldsymbol{k}''}\rangle\tilde{\mathcal{D}}^{(1)}_{0\boldsymbol{k}''}(\omega)\delta(\varepsilon^0_{\boldsymbol{k}'}-\varepsilon^0_{\boldsymbol{k}''}),\tag{263}$$

$$\tilde{\mathcal{D}}^{(1)}_{x\boldsymbol{k}'}(\omega)=A_{x\boldsymbol{k}'}(\omega)+B_{x\boldsymbol{k}'}(\omega)\cos^2\phi'+\frac{1}{2\pi\hbar}\int d^2k''\frac{\tau^0_{\boldsymbol{k}''}}{1-i\omega\tau^0_{\boldsymbol{k}''}}\langle V^{++}_{\boldsymbol{k}''\boldsymbol{k}'}V^{++}_{\boldsymbol{k}'\boldsymbol{k}''}\rangle\tilde{\mathcal{D}}^{(1)}_{x\boldsymbol{k}''}(\omega)\delta(\varepsilon^0_{\boldsymbol{k}'}-\varepsilon^0_{\boldsymbol{k}''}),\tag{264}$$

$$\tilde{\mathcal{D}}^{(1)}_{y\boldsymbol{k}'}(\omega)=C_{x\boldsymbol{k}'}(\omega)\sin\phi'\cos\phi'+\frac{1}{2\pi\hbar}\int d^2k''\frac{\tau^0_{\boldsymbol{k}''}}{1-i\omega\tau^0_{\boldsymbol{k}''}}\langle V^{++}_{\boldsymbol{k}''\boldsymbol{k}'}V^{++}_{\boldsymbol{k}'\boldsymbol{k}''}\rangle\tilde{\mathcal{D}}^{(1)}_{y\boldsymbol{k}''}(\omega)\delta(\varepsilon^0_{\boldsymbol{k}'}-\varepsilon^0_{\boldsymbol{k}''}),\tag{265}$$

$$\tilde{\mathcal{D}}^{(1)}_{z\boldsymbol{k}'}(\omega)=\frac{\sin\theta'\cos\theta'(1+3\cos^2\theta')}{i2\omega\tau^0_{\boldsymbol{k}'}(1+\cos^2\theta')}\cos\phi'+\frac{1}{2\pi\hbar}\int d^2k''\frac{\tau^0_{\boldsymbol{k}''}}{1-i\omega\tau^0_{\boldsymbol{k}''}}\langle V^{++}_{\boldsymbol{k}''\boldsymbol{k}'}V^{++}_{\boldsymbol{k}'\boldsymbol{k}''}\rangle\tilde{\mathcal{D}}^{(1)}_{z\boldsymbol{k}''}(\omega)\delta(\varepsilon^0_{\boldsymbol{k}'}-\varepsilon^0_{\boldsymbol{k}''}).\tag{266}$$

Here $A_{x\boldsymbol{k}'}$, $B_{x\boldsymbol{k}'}$ and $C_{x\boldsymbol{k}'}$ are functions of k' and ω, which are finite in the zero-frequency limit as

$$A_{x\boldsymbol{k}'}(0)=-\frac{1+10\cos^2\theta'+5\cos^4\theta'}{(1+3\cos^2\theta')^2},\tag{267}$$

$$B_{x\boldsymbol{k}'}(0)=C_{x\boldsymbol{k}'}(0)=\frac{8\sin^2\theta'\cos^2\theta'}{(1+3\cos^2\theta')^2}.\tag{268}$$

Accordingly, the equations of $\tilde{\mathcal{D}}^{(1)}_{\mu\boldsymbol{k}'}$ can be solved as

$$\tilde{\mathcal{D}}^{(1)}_{0\boldsymbol{k}'}(\omega)=0,\tag{269}$$

$$\tilde{\mathcal{D}}^{(1)}_{x\boldsymbol{k}'}(\omega)=B_{x\boldsymbol{k}'}(\omega)\cos^2\phi'-\frac{2(1-i\omega\tau^0_{\boldsymbol{k}'})A_{x\boldsymbol{k}'}(\omega)+B_{x\boldsymbol{k}'}(\omega)}{i2\omega\tau^0_{\boldsymbol{k}'}},\tag{270}$$

$$\tilde{\mathcal{D}}^{(1)}_{y\boldsymbol{k}'}(\omega)=C_{x\boldsymbol{k}'}(\omega)\sin\phi'\cos\phi',\tag{271}$$

$$\tilde{\mathcal{D}}^{(1)}_{z\boldsymbol{k}'}(\omega)=\frac{1-i\omega\tau^0_{\boldsymbol{k}'}}{i\omega\tau^0_{\boldsymbol{k}'}}\frac{\sin\theta'\cos\theta'(1+3\cos^2\theta')}{1+3\cos^2\theta'-i2\omega\tau^0_{\boldsymbol{k}'}(1+\cos^2\theta')}\cos\phi'.\tag{272}$$

Thus, we obtain the $\tilde{\mathcal{D}}_{\mu\boldsymbol{k}'}(\omega)$ up to the linear order in t as

$$\tilde{\mathcal{D}}_{0\boldsymbol{k}'}(\omega)=-\frac{1-i\omega\tau^0_{\boldsymbol{k}'}}{i\omega\tau^0_{\boldsymbol{k}'}},\tag{273}$$

$$\begin{aligned}\tilde{\mathcal{D}}_{x\boldsymbol{k}'}(\omega)=&\frac{2(1-i\omega\tau^0_{\boldsymbol{k}'})(1+\cos^2\theta')}{1+3\cos^2\theta'-i2\omega\tau^0_{\boldsymbol{k}'}(1+\cos^2\theta')}\sin\theta'\cos\phi'\\&+\frac{t}{v}\Big[B_{x\boldsymbol{k}'}(\omega)\cos^2\phi'-\frac{2(1-i\omega\tau^0_{\boldsymbol{k}'})A_{x\boldsymbol{k}'}(\omega)+B_{x\boldsymbol{k}'}(\omega)}{i2\omega\tau^0_{\boldsymbol{k}'}}\Big],\end{aligned}\tag{274}$$

$$\tilde{\mathcal{D}}_{y\boldsymbol{k}'}(\omega)=\frac{2(1-i\omega\tau^0_{\boldsymbol{k}'})(1+\cos^2\theta')}{1+3\cos^2\theta'-i2\omega\tau^0_{\boldsymbol{k}'}(1+\cos^2\theta')}\sin\theta'\sin\phi'+\frac{t}{v}C_{x\boldsymbol{k}'}(\omega)\sin\phi'\cos\phi',\tag{275}$$

$$\tilde{\mathcal{D}}_{z\boldsymbol{k}'}(\omega)=-\frac{1-i\omega\tau^0_{\boldsymbol{k}'}}{i\omega\tau^0_{\boldsymbol{k}'}}\cos\theta'\Big[1-\frac{t}{v}\frac{1+3\cos^2\theta'}{1+3\cos^2\theta'-i2\omega\tau^0_{\boldsymbol{k}'}(1+\cos^2\theta')}\sin\theta'\cos\phi'\Big].\tag{276}$$

The above results contain all the information about the vertex and edge corrections of the system.

The relation between the vertex corrections and the diffuson is shown in Supplementary Fig. 29, which indicates that

$$\tilde{v}^{++}_{a\boldsymbol{k}}=v^{++}_{a\boldsymbol{k}}+\int[dk']v^{++}_{a\boldsymbol{k}'}\mathcal{D}_{\boldsymbol{k}\boldsymbol{k}'}(\varepsilon,\omega)\tilde{G}^R_{+\boldsymbol{k}'}(\varepsilon^+_1)\tilde{G}^A_{+\boldsymbol{k}'}(\varepsilon).\tag{277}$$

Based on the diffuson results, the vertex correction can be found as

$$\tilde{v}^{++}_{a\boldsymbol{k}}=v^{++}_{a\boldsymbol{k}}+\frac{\pi n_iV_0^2}{\hbar}\int[dk']\frac{\tau^+_{\boldsymbol{k}'}v^{++}_{a\boldsymbol{k}'}}{1-i\omega\tau^+_{\boldsymbol{k}'}}\big[\tilde{\mathcal{D}}_{0\boldsymbol{k}'}+\tilde{\mathcal{D}}_{x\boldsymbol{k}'}\sin\theta\cos\phi+\tilde{\mathcal{D}}_{y\boldsymbol{k}'}\sin\theta\sin\phi+\tilde{\mathcal{D}}_{z\boldsymbol{k}'}\cos\theta\big]\delta(\varepsilon^+_{\boldsymbol{k}}-\varepsilon^+_{\boldsymbol{k}'}),\tag{278}$$

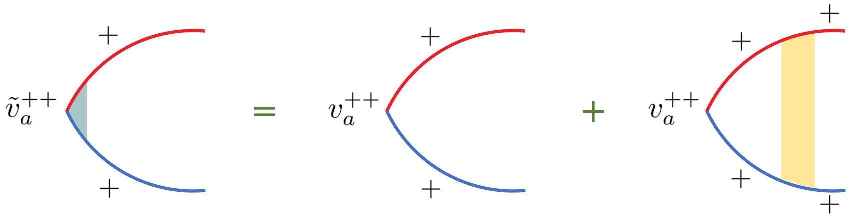

FIG. 29. Diagram representation of the vertex correction equations.

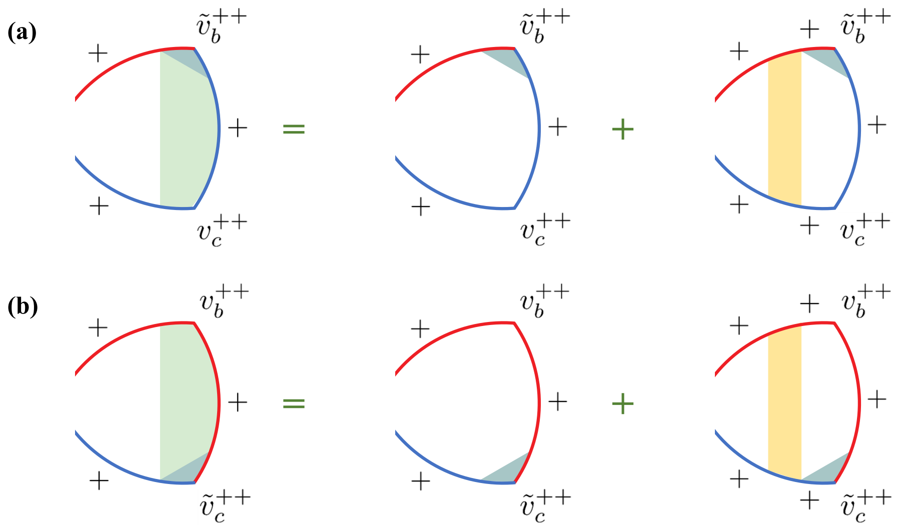

FIG. 30. Diagrammatic representation of the edge vertex correction equations.

where we have multiplied $\delta(\varepsilon - \varepsilon_{\boldsymbol{k}}^{+})$ on both sides of the equation. For the two-dimensional tilted Dirac model, the vertex correction can be calculated as

$$\tilde{v}_{x\boldsymbol{k}}^{++} = \frac{v}{\hbar}\Big\{\frac{2(1+\cos^2\theta)}{1+3\cos^2\theta}\sin\theta\cos\phi + \frac{t}{v}\Big[1 + \frac{\sin^2\theta\cos^2\theta}{1+3\cos^2\theta} + \frac{8\sin^2\theta\cos^2\theta\cos^2\phi}{(1+3\cos^2\theta)^2}\Big]\Big\}, \tag{279}$$

$$\tilde{v}_{y\boldsymbol{k}}^{++} = \frac{v}{\hbar}\Big[\frac{2(1+\cos^2\theta)}{1+3\cos^2\theta}\sin\theta\sin\phi + \frac{t}{v}\frac{8\sin^2\theta\cos^2\theta\sin\phi\cos\phi}{(1+3\cos^2\theta)^2}\Big], \tag{280}$$

where we have neglected the imaginary part of the vertex correction and considered the dc limit. The above results are consistent with the previous ones [Supplementary Eq. (244) and (250)].

The relation between the edge corrections and the diffuson is shown in Supplementary Fig. 30. Here we only need to consider the case shown in Supplementary Fig. 30 (a)

$$\tilde{w}_{bc\boldsymbol{k}}^{++} = \tilde{v}_{b\boldsymbol{k}}^{++} v_{c\boldsymbol{k}}^{++} \tilde{G}_{+\boldsymbol{k}}^{A}(\varepsilon) + \int [dk'] \tilde{v}_{b\boldsymbol{k}'}^{++} v_{c\boldsymbol{k}'}^{++} \mathcal{D}_{\boldsymbol{k}\boldsymbol{k}'}(\varepsilon,\omega) \tilde{G}_{+\boldsymbol{k}'}^{R}(\varepsilon_1^{+}) \tilde{G}_{+\boldsymbol{k}'}^{A}(\varepsilon) \tilde{G}_{+\boldsymbol{k}'}^{A}(\varepsilon), \tag{281}$$

where $\tilde{w}_{bc\boldsymbol{k}}^{++}$ represents the corresponding corrected edge functions and the case shown in Supplementary Fig. 30 (b) with $\tilde{w}_{cb\boldsymbol{k}}^{++}$ is the $b \leftrightarrow c$ counterpart of $\tilde{w}_{bc\boldsymbol{k}}^{++}$. We can multiply $\tilde{G}_{+\boldsymbol{k}}^{R}(\varepsilon_1^{+})\tilde{G}_{+\boldsymbol{k}}^{A}(\varepsilon)$ on both sides of the equations and define that

$$\tilde{G}_{+\boldsymbol{k}}^{R}(\varepsilon_1^{+}) \tilde{w}_{bc\boldsymbol{k}}^{++}(\varepsilon) \tilde{G}_{+\boldsymbol{k}}^{A}(\varepsilon) \equiv \frac{i2\pi}{\hbar^2} \frac{(\tau_{\boldsymbol{k}}^{+})^2}{1 - i\omega\tau_{\boldsymbol{k}}^{+}} \mathcal{W}_{bc\boldsymbol{k}}^{++} \delta(\varepsilon - \varepsilon_{\boldsymbol{k}}^{+}). \tag{282}$$

Then, the edge correction equation can be written as

$$\begin{aligned} \mathcal{W}_{bc\boldsymbol{k}}^{++} = \tilde{v}_{b\boldsymbol{k}}^{++} v_{c\boldsymbol{k}}^{++} + \frac{\pi n_i V_0^2}{\hbar \tau_{\boldsymbol{k}}^{+}} \int [dk'] \frac{(\tau_{\boldsymbol{k}'}^{+})^2}{1 - i\omega\tau_{\boldsymbol{k}'}^{+}} \tilde{v}_{b\boldsymbol{k}'}^{++} v_{c\boldsymbol{k}'}^{++} \\ \times \big[\tilde{\mathcal{D}}_{0\boldsymbol{k}'} + \tilde{\mathcal{D}}_{x\boldsymbol{k}'}\sin\theta\cos\phi + \tilde{\mathcal{D}}_{y\boldsymbol{k}'}\sin\theta\sin\phi + \tilde{\mathcal{D}}_{z\boldsymbol{k}'}\cos\theta\big] \delta(\varepsilon_{\boldsymbol{k}}^{+} - \varepsilon_{\boldsymbol{k}'}^{+}). \end{aligned} \tag{283}$$

In order to calculate the nonlinear conductivity χ_{yxx}, we need the edge corrected function $\mathcal{W}^{++}_{xx\boldsymbol{k}}$. By solving the above equation, we can obtain that

$$\mathcal{W}^{++}_{xx\boldsymbol{k}} = \frac{v^2}{\hbar^2}\Big\{\frac{\sin^2\theta(1+\cos^2\theta)}{(1+3\cos^2\theta)^2}\Big[2(1+3\cos^2\theta)\cos^2\phi - \sin^2\theta\Big] + \frac{t}{v}\frac{\sin\theta\cos\phi}{(1+3\cos^2\theta)^3}$$
$$\times\Big[128 - 288\sin^2\theta + 232\sin^4\theta - 60\sin^6\theta - 3\sin^8\theta + 8(1+3\cos^2\theta)\sin^2\theta\cos^2\theta\cos^2\phi\Big]\Big\}, \tag{284}$$

where we have also neglected the imaginary parts and considered the dc limit. The above calculation shows that the poles structure is unchanged in the edge correction, and thus one can simply identify

$$\tilde{w}^{++}_{bc\boldsymbol{k}}(\varepsilon) = \mathcal{W}^{++}_{bc\boldsymbol{k}}\tilde{G}^A_{+\boldsymbol{k}}(\varepsilon), \tag{285}$$
$$\tilde{w}^{++}_{cb\boldsymbol{k}}(\varepsilon) = \mathcal{W}^{++}_{cb\boldsymbol{k}}\tilde{G}^A_{+\boldsymbol{k}}(\varepsilon), \tag{286}$$

to account for the edge correction.

B. Nonlinear Hall conductivity

1. *Nonlinear Hall conductivity of the 2D tilted Dirac model*

According to the results obtained in Sec. Supplementary Note 3, the non-zero contributions to the nonlinear Hall conductivity χ_{yxx} in time-reversal symmetric systems can be summarized as

$$\chi^{in}_{yxx} = \frac{e^3}{2\hbar}\int[dk]\tau^+_{\boldsymbol{k}}f'(\varepsilon^+_{\boldsymbol{k}})\Omega^+_{z\boldsymbol{k}}\tilde{v}^{++}_{x\boldsymbol{k}}, \tag{287}$$

$$\chi^{sj,1}_{yxx} = \frac{e^3}{2}\int[dk]\int[dk']\tau^+_{\boldsymbol{k}}f'(\varepsilon^+_{\boldsymbol{k}})\Big\{\tau^+_{\boldsymbol{k}}\mathcal{W}^{++}_{xx\boldsymbol{k}}\Big[V^{sj,0}_{y\boldsymbol{k}\boldsymbol{k}'}\delta'(\varepsilon^+_{\boldsymbol{k}}-\varepsilon^+_{\boldsymbol{k}'}) - V^{sj,1}_{y\boldsymbol{k}\boldsymbol{k}'}\delta(\varepsilon^+_{\boldsymbol{k}}-\varepsilon^+_{\boldsymbol{k}'})\Big]$$
$$-\frac{1}{2}\tau^+_{\boldsymbol{k}'}\tilde{v}^{++}_{x\boldsymbol{k}}v^{++}_{x\boldsymbol{k}'}(V^{sj,1}_{y\boldsymbol{k}\boldsymbol{k}'}+V^{sj,2}_{y\boldsymbol{k}\boldsymbol{k}'})\delta(\varepsilon^+_{\boldsymbol{k}}-\varepsilon^+_{\boldsymbol{k}'})\Big\}, \tag{288}$$

$$\chi^{sj,2}_{yxx} = -\frac{e^3}{2}\int[dk]\int[dk']\tau^+_{\boldsymbol{k}}f'(\varepsilon^+_{\boldsymbol{k}})\Big\{\tau^+_{\boldsymbol{k}}\tilde{v}^{++}_{y\boldsymbol{k}}\tilde{v}^{++}_{x\boldsymbol{k}}\Big[V^{sj,0}_{x\boldsymbol{k}\boldsymbol{k}'}\delta'(\varepsilon^+_{\boldsymbol{k}}-\varepsilon^+_{\boldsymbol{k}'}) - V^{sj,2}_{x\boldsymbol{k}\boldsymbol{k}'}\delta(\varepsilon^+_{\boldsymbol{k}}-\varepsilon^+_{\boldsymbol{k}'})\Big]$$
$$+\tau^+_{\boldsymbol{k}'}\tilde{v}^{++}_{y\boldsymbol{k}'}\tilde{v}^{++}_{x\boldsymbol{k}}(V^{sj,1}_{x\boldsymbol{k}\boldsymbol{k}'}+V^{sj,2}_{x\boldsymbol{k}\boldsymbol{k}'})\delta(\varepsilon^+_{\boldsymbol{k}}-\varepsilon^+_{\boldsymbol{k}'})\Big\}, \tag{289}$$

$$\chi^{sk,1}_{yxx} = \frac{e^3}{2}\int[dk]\int[dk']\int[dk'']\tau^+_{\boldsymbol{k}}\tau^+_{\boldsymbol{k}'}f'(\varepsilon^+_{\boldsymbol{k}})\Big[\Big(\tau^+_{\boldsymbol{k}'}\tilde{v}^{++}_{y\boldsymbol{k}}\mathcal{W}^{++}_{xx\boldsymbol{k}'} - \frac{\tau^+_{\boldsymbol{k}}}{2}\tilde{v}^{++}_{y\boldsymbol{k}}\tilde{v}^{++}_{x\boldsymbol{k}}v^{++}_{x\boldsymbol{k}'}\Big)W^{sk,1\alpha}_{\boldsymbol{k}\boldsymbol{k}'\boldsymbol{k}''}\delta'(\varepsilon^+_{\boldsymbol{k}}-\varepsilon^+_{\boldsymbol{k}''})$$
$$-\Big(\tau^+_{\boldsymbol{k}'}\tilde{v}^{++}_{y\boldsymbol{k}}\mathcal{W}^{++}_{xx\boldsymbol{k}'}W^{sk,1\beta}_{\boldsymbol{k}\boldsymbol{k}'\boldsymbol{k}''} - \frac{\tau^+_{\boldsymbol{k}}}{2}\tilde{v}^{++}_{y\boldsymbol{k}}\tilde{v}^{++}_{x\boldsymbol{k}}v^{++}_{x\boldsymbol{k}'}W^{sk,1\gamma}_{\boldsymbol{k}\boldsymbol{k}'\boldsymbol{k}''}\Big)\delta(\varepsilon^+_{\boldsymbol{k}}-\varepsilon^+_{\boldsymbol{k}''})\Big]\delta(\varepsilon^+_{\boldsymbol{k}}-\varepsilon^+_{\boldsymbol{k}'}), \tag{290}$$

$$\chi^{sk,2}_{yxx} = \frac{e^3}{2}\int[dk]\int[dk']\int[dk'']\tau^+_{\boldsymbol{k}}\tau^+_{\boldsymbol{k}'}f'(\varepsilon^+_{\boldsymbol{k}})\Big(\tau^+_{\boldsymbol{k}'}\tilde{v}^{++}_{y\boldsymbol{k}}\mathcal{W}^{++}_{xx\boldsymbol{k}'} - \frac{\tau^+_{\boldsymbol{k}}}{2}\tilde{v}^{++}_{y\boldsymbol{k}}\tilde{v}^{++}_{x\boldsymbol{k}}v^{++}_{x\boldsymbol{k}'}\Big)W^{sk,2}_{\boldsymbol{k}\boldsymbol{k}'\boldsymbol{k}''}\delta'(\varepsilon^+_{\boldsymbol{k}}-\varepsilon^+_{\boldsymbol{k}''}). \tag{291}$$

Supplementary Note 9. SYMMETRY ASPECTS OF THE NONLINEAR RESPONSE

According to our previous diagrammatic analysis, the disorder-induced extrinsic contribution to the quadratic nonlinear conductivity is a rank-three tensor with the constraint $\chi^{ex}_{abc} = \chi^{ex}_{acb}$, while the Berry curvature dipole related intrinsic contribution has extra antisymmetric properties under the label exchanges $a \leftrightarrow b$ or $a \leftrightarrow c$ as described by

$$\chi^{ex}_{abc} = T_{abc} + T_{acb}, \tag{292}$$
$$\chi^{in}_{abc} = \varepsilon_{abd}T_{cd} + \varepsilon_{acd}T_{bd}, \tag{293}$$

where T_{ab} and T_{abc} are general rank-two and rank-three tensors, respectively. The extrinsic response tensor χ^{ex}_{abc} has more nonzero elements than χ^{in}_{abc}. More importantly, the different symmetry properties under the exchange of labels would impose very different constraints on the elements of the nonlinear response tensor.

For a 2D system, we only have two directions, x and y, and thus the quadratic response can only be measured with the input electric fields E_x^2, E_y^2, E_xE_y, and E_yE_x. Due to the $b \leftrightarrow c$ symmetry, we can arrange the response tensor

into the matrix form in 2D as

$$\begin{pmatrix} J_x \\ J_y \end{pmatrix} = \begin{pmatrix} \chi_{xxx} & \chi_{xxy} & \chi_{xyy} \\ \chi_{yxx} & \chi_{yyx} & \chi_{yyy} \end{pmatrix} \begin{pmatrix} E_x^2 \\ 2E_xE_y \\ E_y^2 \end{pmatrix}, \tag{294}$$

which includes 6 independent elements. As the intrinsic contribution also has $b \leftrightarrow c$ symmetry, from now on we neglect the superscript ex in the general discussion. While for the intrinsic contribution, due to the extra $a \leftrightarrow b$ or $a \leftrightarrow c$ symmetries, we have

$$\begin{pmatrix} J_x \\ J_y \end{pmatrix} = \begin{pmatrix} 0 & -\chi_{yxx}^{in} & \chi_{xyy}^{in} \\ \chi_{yxx}^{in} & -\chi_{xyy}^{in} & 0 \end{pmatrix} \begin{pmatrix} E_x^2 \\ 2E_xE_y \\ E_y^2 \end{pmatrix}, \tag{295}$$

which includes 2 independent elements.

For a 3D system, we only have three directions, x, y and z, the quadratic response can only be measured with the input electric fields E_x^2, E_y^2, E_z^2, E_xE_y, E_yE_x, E_xE_z, E_zE_x, E_zE_y, and E_yE_z. Due to the $b \leftrightarrow c$ symmetry, we can arrange the response tensor into the matrix form in 3D as

$$\begin{pmatrix} J_x \\ J_y \\ J_z \end{pmatrix} = \begin{pmatrix} \chi_{xxx} & \chi_{xxy} & \chi_{xyy} & \chi_{xxz} & \chi_{xyz} & \chi_{xzz} \\ \chi_{yxx} & \chi_{yyx} & \chi_{yyy} & \chi_{yzx} & \chi_{yyz} & \chi_{yzz} \\ \chi_{zxx} & \chi_{zxy} & \chi_{zyy} & \chi_{zzx} & \chi_{zzy} & \chi_{zzz} \end{pmatrix} \begin{pmatrix} E_x^2 \\ 2E_xE_y \\ E_y^2 \\ 2E_xE_z \\ 2E_yE_z \\ E_z^2 \end{pmatrix}, \tag{296}$$

which includes 18 independent elements. While for the intrinsic contribution, due to the $a \leftrightarrow b$ or $a \leftrightarrow c$ symmetries, we have

$$\begin{pmatrix} J_x \\ J_y \\ J_z \end{pmatrix} = \begin{pmatrix} 0 & -\chi_{yxx}^{in} & \chi_{xyy}^{in} & -\chi_{zxx}^{in} & \chi_{xyz}^{in} & \chi_{xzz}^{in} \\ \chi_{yxx}^{in} & -\chi_{xyy}^{in} & 0 & \chi_{yzx}^{in} & -\chi_{zyy}^{in} & \chi_{yzz}^{in} \\ \chi_{zxx}^{in} & \chi_{zxy}^{in} & \chi_{zyy}^{in} & -\chi_{xzz}^{in} & -\chi_{yzz}^{in} & 0 \end{pmatrix} \begin{pmatrix} E_x^2 \\ 2E_xE_y \\ E_y^2 \\ 2E_xE_z \\ 2E_yE_z \\ E_z^2 \end{pmatrix}, \tag{297}$$

which includes 8 independent elements due to the constraint $\chi_{xyz}^{in} + \chi_{yzx}^{in} + \chi_{zxy}^{in} = 0$. It is worth noting that the response tensor in 2D is just a subspace of the 3D case in our arrangement of the electric field basis.

In this section, we consider the constraint imposed by the space symmetry of the system. We first consider some specific symmetry operations including inversion ($\mathcal{I}$), mirror-reflection ($\mathcal{M}$) and n-fold rotation ($\mathcal{C}_n$) symmetry. Then, for a complete consideration, we explicitly check the 32 point groups and determine the allowed form of the susceptibility tensor for each group.

A. $\mathcal{I}$-symmetry

As both charge current $\boldsymbol{J}$ and electric field $\boldsymbol{E}$ are odd under inversion symmetry, the expression of the current then transforms as

$$\mathcal{I}: \quad J_a = -\chi_{abc}E_bE_c \tag{298}$$

under $\mathcal{I}$ operation. Thus, for a $\mathcal{I}$-symmetric system, we should have

$$\chi_{abc} = -\chi_{abc}, \tag{299}$$

which indicates a vanishing nonlinear response. Thus, we have the conclusion that the nonlinear Hall current can only appear in $\mathcal{I}$-broken systems. It is worth noting that this conclusion is independent of the mechanisms, and thus it is a real generic property of the quadratic nonlinear response.

B. $\mathcal{M}$-symmetry

Different from the $\mathcal{I}$-symmetry, a mirror-reflection operation only changes the sign of a vector with specific orientation, such as

$$\mathcal{M}_a: \quad (V_a, V_b, V_c, \cdots) \Rightarrow (-V_a, V_b, V_c, \cdots), \tag{300}$$

where $\boldsymbol{V} = (V_a, V_b, V_c, \cdots)$ represents a general vector. As the 2D response tensor is the subspace of the 3D one, here we only consider the effect of the $\mathcal{M}$-symmetry in 3D systems. Also, because $\mathcal{M}$-symmetry only changes the sign of the elements, the only constraint it can impose is to make certain elements vanish. Thus, considering $\mathcal{M}_x$, $\mathcal{M}_y$ and $\mathcal{M}_z$ explicitly, we have

$$\mathcal{M}_x: \quad \chi = \begin{pmatrix} 0 & \chi_{xxy} & 0 & \chi_{xxz} & 0 & 0 \\ \chi_{yxx} & 0 & \chi_{yyy} & 0 & \chi_{yyz} & \chi_{yzz} \\ \chi_{zxx} & 0 & \chi_{zyy} & 0 & \chi_{zzy} & \chi_{zzz} \end{pmatrix}, \tag{301}$$

$$\mathcal{M}_y: \quad \chi = \begin{pmatrix} \chi_{xxx} & 0 & \chi_{xyy} & \chi_{xxz} & 0 & \chi_{xzz} \\ 0 & \chi_{yyx} & 0 & 0 & \chi_{yyz} & 0 \\ \chi_{zxx} & 0 & \chi_{zyy} & \chi_{zzx} & 0 & \chi_{zzz} \end{pmatrix} \tag{302}$$

$$\mathcal{M}_z: \quad \chi = \begin{pmatrix} \chi_{xxx} & \chi_{xxy} & \chi_{xyy} & 0 & 0 & \chi_{xzz} \\ \chi_{yxx} & \chi_{yyx} & \chi_{yyy} & 0 & 0 & \chi_{yzz} \\ 0 & 0 & 0 & \chi_{zzx} & \chi_{zzy} & 0 \end{pmatrix} \tag{303}$$

The above relations indicate an important conclusion that for systems with $\mathcal{M}_a$-symmetry (the mirror plane is the b-c plane) all the response elements with odd numbers of subscript a vanish. According to our results, a single mirror plane would eliminate 8 elements.

C. $\mathcal{C}_n$-symmetry

A more complex case is the n-fold rotation symmetry, which can be performed via the special orthogonal matrix

$$\hat{C}_n = \begin{pmatrix} \cos\theta_n & \sin\theta_n & 0 \\ -\sin\theta_n & \cos\theta_n & 0 \\ 0 & 0 & 1 \end{pmatrix} \tag{304}$$

with $\theta_n \in (2\pi/n, 4\pi/n, \cdots, 2\pi)$ around a certain axis. Assuming a $\mathcal{C}_n$ operation around the z-axis, the current and electric field transform as

$$\begin{pmatrix} J_x \\ J_y \\ J_z \end{pmatrix} \Rightarrow \begin{pmatrix} \cos\theta_n & \sin\theta_n & 0 \\ -\sin\theta_n & \cos\theta_n & 0 \\ 0 & 0 & 1 \end{pmatrix} \begin{pmatrix} J_x \\ J_y \\ J_z \end{pmatrix}, \tag{305}$$

$$\begin{pmatrix} E_x \\ E_y \\ E_z \end{pmatrix} \Rightarrow \begin{pmatrix} \cos\theta_n & \sin\theta_n & 0 \\ -\sin\theta_n & \cos\theta_n & 0 \\ 0 & 0 & 1 \end{pmatrix} \begin{pmatrix} E_x \\ E_y \\ E_z \end{pmatrix}. \tag{306}$$

For a 3D system under a $\mathcal{C}_n$ operation, the above new basis of the electric field transforms as

$$\begin{pmatrix} E_x^2 \\ 2E_xE_y \\ E_y^2 \\ 2E_xE_z \\ 2E_yE_z \\ E_z^2 \end{pmatrix} \Rightarrow \begin{pmatrix} \cos^2\theta_n & \cos\theta_n\sin\theta_n & \sin^2\theta_n & 0 & 0 & 0 \\ -2\cos\theta_n\sin\theta_n & \cos^2\theta_n - \sin^2\theta_n & 2\cos\theta_n\sin\theta_n & 0 & 0 & 0 \\ \sin^2\theta_n & -\cos\theta_n\sin\theta_n & \cos^2\theta_n & 0 & 0 & 0 \\ 0 & 0 & 0 & \cos\theta_n & \sin\theta_n & 0 \\ 0 & 0 & 0 & -\sin\theta_n & \cos\theta_n & 0 \\ 0 & 0 & 0 & 0 & 0 & 1 \end{pmatrix} \begin{pmatrix} E_x^2 \\ 2E_xE_y \\ E_y^2 \\ 2E_xE_z \\ 2E_yE_z \\ E_z^2 \end{pmatrix}, \tag{307}$$

where the transformation matrix can be rewritten as

$$\begin{pmatrix} \hat{C}'_n & 0 \\ 0 & \hat{C}_n \end{pmatrix} \tag{308}$$

with

$$\hat{C}'_{n,\alpha} = \begin{pmatrix} \cos^2\theta_n & \cos\theta_n\sin\theta_n & \sin^2\theta_n \\ -2\cos\theta_n\sin\theta_n & \cos^2\theta_n - \sin^2\theta_n & 2\cos\theta_n\sin\theta_n \\ \sin^2\theta_n & -\cos\theta_n\sin\theta_n & \cos^2\theta_n \end{pmatrix}. \tag{309}$$

Accordingly, we can decompose the response function χ into two parts as

$$\chi = (\chi_\alpha, \chi_\beta) \tag{310}$$

with

$$\chi_\alpha = \begin{pmatrix} \chi_{xxx} & \chi_{xxy} & \chi_{xyy} \\ \chi_{yxx} & \chi_{yyx} & \chi_{yyy} \\ \chi_{zxx} & \chi_{zxy} & \chi_{zyy} \end{pmatrix}, \qquad \chi_\beta = \begin{pmatrix} \chi_{xxz} & \chi_{xyz} & \chi_{xzz} \\ \chi_{yzx} & \chi_{yyz} & \chi_{yzz} \\ \chi_{zzx} & \chi_{zzy} & \chi_{zzz} \end{pmatrix}, \tag{311}$$

which transform under the $\mathcal{C}_n$-rotation as

$$\begin{aligned} \chi_\alpha &\Rightarrow \mathcal{C}_n\chi_\alpha\mathcal{C}'^{-1}_n \\ &= \begin{pmatrix} \cos\theta_n & \sin\theta_n & 0 \\ -\sin\theta_n & \cos\theta_n & 0 \\ 0 & 0 & 1 \end{pmatrix} \begin{pmatrix} \chi_{xxx} & \chi_{xxy} & \chi_{xyy} \\ \chi_{yxx} & \chi_{yyx} & \chi_{yyy} \\ \chi_{zxx} & \chi_{zxy} & \chi_{zyy} \end{pmatrix} \begin{pmatrix} \cos^2\theta_n & -\cos\theta_n\sin\theta_n & \sin^2\theta_n \\ 2\cos\theta_n\sin\theta_n & \cos^2\theta_n - \sin^2\theta_n & -2\cos\theta_n\sin\theta_n \\ \sin^2\theta_n & \cos\theta_n\sin\theta_n & \cos^2\theta_n \end{pmatrix}, \end{aligned} \tag{312}$$

$$\begin{aligned} \chi_\beta &\Rightarrow \mathcal{C}_n\chi_\beta\mathcal{C}^{-1}_n \\ &= \begin{pmatrix} \cos\theta_n & \sin\theta_n & 0 \\ -\sin\theta_n & \cos\theta_n & 0 \\ 0 & 0 & 1 \end{pmatrix} \begin{pmatrix} \chi_{xxz} & \chi_{xyz} & \chi_{xzz} \\ \chi_{yzx} & \chi_{yyz} & \chi_{yzz} \\ \chi_{zzx} & \chi_{zzy} & \chi_{zzz} \end{pmatrix} \begin{pmatrix} \cos\theta_n & -\sin\theta_n & 0 \\ \sin\theta_n & \cos\theta_n & 0 \\ 0 & 0 & 1 \end{pmatrix}. \end{aligned} \tag{313}$$

By identifying the elements before and after the above transformation, we can obtain a series of coupled equations, which represents the constraint imposed by the $\mathcal{C}_n$-rotation symmetry.

Before performing detailed calculations, there are some general considerations to note. The $\mathcal{C}_n$-rotation symmetry can be classified into two categories as $\mathcal{C}_{2n}$-rotation and $\mathcal{C}_{2n+1}$-rotation symmetries. Now we consider them explicitly and the rotation axis is assumed as z.

1. $\mathcal{C}_{2n}$-symmetry

For a $\mathcal{C}_{2n}$-symmetric system, we always have an effective 2D inversion symmetry within the xy-plane, thus all of the response elements with odd number x and y subscripts vanish according to our previous analysis. That is

$$\chi = (\chi_\alpha, \chi_\beta) \tag{314}$$

with

$$\chi_\alpha = \begin{pmatrix} 0 & 0 & 0 \\ 0 & 0 & 0 \\ \chi_{zxx} & \chi_{zxy} & \chi_{zyy} \end{pmatrix}, \qquad \chi_\beta = \begin{pmatrix} \chi_{xxz} & \chi_{xyz} & 0 \\ \chi_{yzx} & \chi_{yyz} & 0 \\ 0 & 0 & \chi_{zzz} \end{pmatrix}, \tag{315}$$

which also transform like Supplementary Eq. (312) and (313).

In all the 32 point groups, the allowed $\mathcal{C}_{2n}$-symmetries are $\mathcal{C}_2$, $\mathcal{C}_4$, and $\mathcal{C}_6$. The $\mathcal{C}_2$-symmetry is nothing but the effective 2D inversion symmetry within the xy-plane, which has been included in the form of χ as

$$\mathcal{C}_2: \quad \chi = \begin{pmatrix} 0 & 0 & 0 & \chi_{xxz} & \chi_{xyz} & 0 \\ 0 & 0 & 0 & \chi_{yzx} & \chi_{yyz} & 0 \\ \chi_{zxx} & \chi_{zxy} & \chi_{zyy} & 0 & 0 & \chi_{zzz} \end{pmatrix}. \tag{316}$$

For the $\mathcal{C}_4$-symmetry, we can obtain the constraints as

$$\begin{pmatrix} 0 & 0 & 0 \\ 0 & 0 & 0 \\ \chi_{zxx} & \chi_{zxy} & \chi_{zyy} \end{pmatrix} = \begin{pmatrix} 0 & 0 & 0 \\ 0 & 0 & 0 \\ \chi_{zyy} & -\chi_{zxy} & \chi_{zxx} \end{pmatrix}, \tag{317}$$

$$\begin{pmatrix} \chi_{xxz} & \chi_{xyz} & 0 \\ \chi_{yzx} & \chi_{yyz} & 0 \\ 0 & 0 & \chi_{zzz} \end{pmatrix} = \begin{pmatrix} \chi_{yyz} & -\chi_{yzx} & 0 \\ -\chi_{xyz} & \chi_{xxz} & 0 \\ 0 & 0 & \chi_{zzz} \end{pmatrix}. \tag{318}$$

Thus, we have

$$\mathcal{C}_4: \quad \chi = \begin{pmatrix} 0 & 0 & 0 & \chi_{xxz} & \chi_{xyz} & 0 \\ 0 & 0 & 0 & -\chi_{xyz} & \chi_{xxz} & 0 \\ \chi_{zxx} & 0 & \chi_{zxx} & 0 & 0 & \chi_{zzz} \end{pmatrix}. \tag{319}$$

For the $\mathcal{C}_6$-symmetry, we can obtain the constraints as

$$\begin{pmatrix} 0 & 0 & 0 \\ 0 & 0 & 0 \\ \chi_{zxx} & \chi_{zxy} & \chi_{zyy} \end{pmatrix} = \begin{pmatrix} 0 & 0 & 0 \\ 0 & 0 & 0 \\ \chi_{zyy} & 0 & \chi_{zxx} \end{pmatrix}, \tag{320}$$

$$\begin{pmatrix} \chi_{xxz} & \chi_{xyz} & 0 \\ \chi_{yzx} & \chi_{yyz} & 0 \\ 0 & 0 & \chi_{zzz} \end{pmatrix} = \begin{pmatrix} \chi_{yyz} & -\chi_{yzx} & 0 \\ -\chi_{xyz} & \chi_{xxz} & 0 \\ 0 & 0 & \chi_{zzz} \end{pmatrix}. \tag{321}$$

Thus, we have

$$\mathcal{C}_6: \quad \chi = \begin{pmatrix} 0 & 0 & 0 & \chi_{xxz} & \chi_{xyz} & 0 \\ 0 & 0 & 0 & -\chi_{xyz} & \chi_{xxz} & 0 \\ \chi_{zxx} & 0 & \chi_{zxx} & 0 & 0 & \chi_{zzz} \end{pmatrix}. \tag{322}$$

2. $\mathcal{C}_{2n+1}$-symmetry

In all of the 32 point groups, the only allowed $\mathcal{C}_{2n+1}$-symmetry is $\mathcal{C}_3$. Thus, here we only need to consider the $\mathcal{C}_3$-symmetry, which imposes the constraints

$$\begin{pmatrix} \chi_{xxx} & \chi_{xxy} & \chi_{xyy} \\ \chi_{yxx} & \chi_{yyx} & \chi_{yyy} \\ \chi_{zxx} & \chi_{zxy} & \chi_{zyy} \end{pmatrix} = \begin{pmatrix} \chi_{xxx} & -\chi_{yyy} & -\chi_{xxx} \\ -\chi_{yyy} & -\chi_{xxx} & \chi_{yyy} \\ \chi_{zxx} & 0 & \chi_{zxx} \end{pmatrix}, \tag{323}$$

$$\begin{pmatrix} \chi_{xxz} & \chi_{xyz} & \chi_{xzz} \\ \chi_{yzx} & \chi_{yyz} & \chi_{yzz} \\ \chi_{zzx} & \chi_{zzy} & \chi_{zzz} \end{pmatrix} = \begin{pmatrix} -\chi_{xxx} & \chi_{xyz} & 0 \\ -\chi_{xyz} & -\chi_{xxx} & 0 \\ 0 & 0 & \chi_{zzz} \end{pmatrix}. \tag{324}$$

Thus, we have

$$\mathcal{C}_3: \quad \chi = \begin{pmatrix} \chi_{xxx} & -\chi_{yyy} & -\chi_{xxx} & -\chi_{xxx} & \chi_{xyz} & 0 \\ -\chi_{yyy} & -\chi_{xxx} & \chi_{yyy} & -\chi_{xyz} & -\chi_{xxx} & 0 \\ \chi_{zxx} & 0 & \chi_{zxx} & 0 & 0 & \chi_{zzz} \end{pmatrix}. \tag{325}$$

D. 32 Point groups

A systematic way of discussing the effect of symmetry is based on the point symmetry, which includes 32 point groups in 7 classes. As the nonlinear Hall current can only appear in $\mathcal{I}$-broken systems, we only need to consider 21 of the 32 point groups that lack an inversion center. We use the red color to emphasize the elements that exist in χ^{ex}

but vanish in χ^{in}. These elements are only contributed by the disorder effects, and thus are irrelevant to the Berry curvature.

---

[1] I. Sodemann and L. Fu, "Quantum nonlinear Hall effect induced by Berry curvature dipole in time-reversal invariant materials", Phys. Rev. Lett. **115**, 216806 (2015).
[2] Q. Ma, *et al.*, "Observation of the nonlinear Hall effect under time-reversal-symmetric conditions", Nature **565**, 337 (2019).
[3] K. Kang, T. Li, E. Sohn, J. Shan, and K. F. Mak, "Observation of the nonlinear anomalous Hall effect in few-layer WTe_2", Nature Mater. **18**, 324 (2019).
[4] Z. Z. Du, C. M. Wang, S. Li, H.-Z. Lu, and X. C. Xie, "Disorder-induced nonlinear Hall effect with time-reversal symmetry", Nature Commun. **10**, 3047 (2019).
[5] C. Xiao, Z. Z. Du, and Q. Niu, "Theory of nonlinear Hall effects: Modified semiclassics from quantum kinetics", Phys. Rev. B **100**, 165422 (2019).
[6] S. Nandy and I. Sodemann, "Symmetry and quantum kinetics of the nonlinear Hall effect", Phys. Rev. B **100**, 195117 (2019).
[7] D. E. Parker, T. Morimoto, J. Orenstein, and J. E. Moore, "Diagrammatic approach to nonlinear optical response with application to Weyl semimetals", Phys. Rev. B **99**, 045121 (2019).
[8] G. D. Mahan, *Many-Particle Physics* (Plenum Press, 1990).
[9] N. Sinitsyn, A. MacDonald, T. Jungwirth, V. Dugaev, and J. Sinova, "Anomalous Hall effect in a two-dimensional Dirac band: The link between the Kubo-Streda formula and the semiclassical Boltzmann equation approach", Phys. Rev. B **75**, 045315 (2007).
[10] N. Nagaosa, J. Sinova, S. Onoda, A. H. MacDonald, and N. P. Ong, "Anomalous Hall effect", Rev. Mod. Phys. **82**, 1539 (2010).
[11] N. Sinitsyn, "Semiclassical theories of the anomalous Hall effect", J. Phys.: Condens. Matter **20**, 023201 (2008).
[12] D. Xiao, M. C. Chang, and Q. Niu, "Berry phase effects on electronic properties", Rev. Mod. Phys. **82**, 1959 (2010).
[13] Z. Z. Du, C. M. Wang, H.-Z. Lu, and X. C. Xie, "Band signatures for strong nonlinear Hall effect in bilayer WTe_2", Phys. Rev. Lett. **121**, 266601 (2018).

TABLE 2. **Nonzero nonlinear Hall response elements in 32 point groups (3D).** The matrices are defined in Supplementary Eq. (296). The elements that exist in χ^{ex} but vanish in χ^{in} are highlighted in red.

Classes	Groups	Extrinsic contribution	Intrinsic contribution
Triclinic	C_1	$\begin{pmatrix} {\color{red}\chi^{ex}_{xxx}} & \chi^{ex}_{xxy} & \chi^{ex}_{xyy} & \chi^{ex}_{xxz} & \chi^{ex}_{xyz} & \chi^{ex}_{xzz} \\ \chi^{ex}_{yxx} & \chi^{ex}_{yyx} & {\color{red}\chi^{ex}_{yyy}} & \chi^{ex}_{yzx} & \chi^{ex}_{yyz} & \chi^{ex}_{yzz} \\ \chi^{ex}_{zxx} & \chi^{ex}_{zxy} & \chi^{ex}_{zyy} & \chi^{ex}_{zzx} & \chi^{ex}_{zzy} & {\color{red}\chi^{ex}_{zzz}} \end{pmatrix}$	$\begin{pmatrix} 0 & -\chi^{in}_{yxx} & \chi^{in}_{xyy} & -\chi^{in}_{zxx} & \chi^{in}_{xyz} & \chi^{in}_{xzz} \\ \chi^{in}_{yxx} & -\chi^{in}_{xyy} & 0 & \chi^{in}_{yzx} & -\chi^{in}_{zyy} & \chi^{in}_{yzz} \\ \chi^{in}_{zxx} & -\chi^{in}_{xyz} - \chi^{in}_{yzx} & \chi^{in}_{zyy} & -\chi^{in}_{xzz} & -\chi^{in}_{yzz} & 0 \end{pmatrix}$
Monoclinic	C_{1v}	$\begin{pmatrix} 0 & \chi^{ex}_{xxy} & 0 & \chi^{ex}_{xxz} & 0 & 0 \\ \chi^{ex}_{yxx} & 0 & {\color{red}\chi^{ex}_{yyy}} & 0 & \chi^{ex}_{yyz} & \chi^{ex}_{yzz} \\ \chi^{ex}_{zxx} & 0 & \chi^{ex}_{zyy} & 0 & \chi^{ex}_{zzy} & {\color{red}\chi^{ex}_{zzz}} \end{pmatrix}$	$\begin{pmatrix} 0 & -\chi^{in}_{yxx} & 0 & -\chi^{in}_{zxx} & 0 & 0 \\ \chi^{in}_{yxx} & 0 & 0 & 0 & -\chi^{in}_{zyy} & \chi^{in}_{yzz} \\ \chi^{in}_{zxx} & 0 & \chi^{in}_{zyy} & 0 & -\chi^{in}_{yzz} & 0 \end{pmatrix}$
Monoclinic	C_2	$\begin{pmatrix} 0 & 0 & 0 & \chi^{ex}_{xxz} & \chi^{ex}_{xyz} & 0 \\ 0 & 0 & 0 & \chi^{ex}_{yzx} & \chi^{ex}_{yyz} & 0 \\ \chi^{ex}_{zxx} & \chi^{ex}_{zxy} & \chi^{ex}_{zyy} & 0 & 0 & {\color{red}\chi^{ex}_{zzz}} \end{pmatrix}$	$\begin{pmatrix} 0 & 0 & 0 & -\chi^{in}_{zxx} & \chi^{in}_{xyz} & 0 \\ 0 & 0 & 0 & \chi^{in}_{yzx} & -\chi^{in}_{zyy} & 0 \\ \chi^{in}_{zxx} & -\chi^{in}_{xyz} - \chi^{in}_{yzx} & \chi^{in}_{zyy} & 0 & 0 & 0 \end{pmatrix}$
Orthorhombic	C_{2v}	$\begin{pmatrix} 0 & 0 & 0 & \chi^{ex}_{xxz} & 0 & 0 \\ 0 & 0 & 0 & 0 & \chi^{ex}_{yyz} & 0 \\ \chi^{ex}_{zxx} & 0 & \chi^{ex}_{zyy} & 0 & 0 & {\color{red}\chi^{ex}_{zzz}} \end{pmatrix}$	$\begin{pmatrix} 0 & 0 & 0 & -\chi^{in}_{zxx} & 0 & 0 \\ 0 & 0 & 0 & 0 & -\chi^{in}_{zyy} & 0 \\ \chi^{in}_{zxx} & 0 & \chi^{in}_{zyy} & 0 & 0 & 0 \end{pmatrix}$
Orthorhombic	D_2	$\begin{pmatrix} 0 & 0 & 0 & 0 & \chi^{ex}_{xyz} & 0 \\ 0 & 0 & 0 & \chi^{ex}_{yzx} & 0 & 0 \\ 0 & \chi^{ex}_{zxy} & 0 & 0 & 0 & 0 \end{pmatrix}$	$\begin{pmatrix} 0 & 0 & 0 & 0 & \chi^{in}_{xyz} & 0 \\ 0 & 0 & 0 & \chi^{in}_{yzx} & 0 & 0 \\ 0 & -\chi^{in}_{xyz} - \chi^{in}_{yzx} & 0 & 0 & 0 & 0 \end{pmatrix}$
Tetragonal	C_4	$\begin{pmatrix} 0 & 0 & 0 & \chi^{ex}_{xxz} & \chi^{ex}_{xyz} & 0 \\ 0 & 0 & 0 & -\chi^{ex}_{xyz} & \chi^{ex}_{xxz} & 0 \\ \chi^{ex}_{zxx} & 0 & \chi^{ex}_{zxx} & 0 & 0 & {\color{red}\chi^{ex}_{zzz}} \end{pmatrix}$	$\begin{pmatrix} 0 & 0 & 0 & -\chi^{in}_{zxx} & \chi^{in}_{xyz} & 0 \\ 0 & 0 & 0 & -\chi^{in}_{xyz} & -\chi^{in}_{zxx} & 0 \\ \chi^{in}_{zxx} & 0 & \chi^{in}_{zxx} & 0 & 0 & 0 \end{pmatrix}$
Tetragonal	C_{4v}	$\begin{pmatrix} 0 & 0 & 0 & \chi^{ex}_{xxz} & 0 & 0 \\ 0 & 0 & 0 & 0 & \chi^{ex}_{xxz} & 0 \\ \chi^{ex}_{zxx} & 0 & \chi^{ex}_{zxx} & 0 & 0 & {\color{red}\chi^{ex}_{zzz}} \end{pmatrix}$	$\begin{pmatrix} 0 & 0 & 0 & -\chi^{in}_{zxx} & 0 & 0 \\ 0 & 0 & 0 & 0 & -\chi^{in}_{zxx} & 0 \\ \chi^{in}_{zxx} & 0 & \chi^{in}_{zxx} & 0 & 0 & 0 \end{pmatrix}$
Tetragonal	D_4	$\begin{pmatrix} 0 & 0 & 0 & 0 & \chi^{ex}_{xyz} & 0 \\ 0 & 0 & 0 & -\chi^{ex}_{xyz} & 0 & 0 \\ 0 & 0 & 0 & 0 & 0 & 0 \end{pmatrix}$	$\begin{pmatrix} 0 & 0 & 0 & 0 & \chi^{in}_{xyz} & 0 \\ 0 & 0 & 0 & -\chi^{in}_{xyz} & 0 & 0 \\ 0 & 0 & 0 & 0 & 0 & 0 \end{pmatrix}$
Tetragonal	S_4	$\begin{pmatrix} 0 & 0 & 0 & \chi^{ex}_{xxz} & \chi^{ex}_{xyz} & 0 \\ 0 & 0 & 0 & \chi^{ex}_{xyz} & -\chi^{ex}_{xxz} & 0 \\ \chi^{ex}_{zxx} & \chi^{ex}_{zxy} & -\chi^{ex}_{zxx} & 0 & 0 & 0 \end{pmatrix}$	$\begin{pmatrix} 0 & 0 & 0 & -\chi^{in}_{zxx} & \chi^{in}_{xyz} & 0 \\ 0 & 0 & 0 & \chi^{in}_{xyz} & \chi^{in}_{zxx} & 0 \\ \chi^{in}_{zxx} & -2\chi^{in}_{xyz} & -\chi^{in}_{zxx} & 0 & 0 & 0 \end{pmatrix}$
Tetragonal	D_{2d}	$\begin{pmatrix} 0 & 0 & 0 & 0 & \chi^{ex}_{xyz} & 0 \\ 0 & 0 & 0 & \chi^{ex}_{xyz} & 0 & 0 \\ 0 & \chi^{ex}_{zxy} & 0 & 0 & 0 & 0 \end{pmatrix}$	$\begin{pmatrix} 0 & 0 & 0 & 0 & \chi^{in}_{xyz} & 0 \\ 0 & 0 & 0 & \chi^{in}_{xyz} & 0 & 0 \\ 0 & -2\chi^{in}_{xyz} & 0 & 0 & 0 & 0 \end{pmatrix}$

The C_n axis is assumed in order as the z-, x- and y-axis, the mirror plane σ_v is assumed in order as the yz- and zx-plane and the mirror plane σ_h is assumed as the xy-plane.

Classes	Groups	Extrinsic contribution	Intrinsic contribution
Cubic	T	$\begin{pmatrix} 0 & 0 & 0 & 0 & \chi^{ex}_{xyz} & 0 \\ 0 & 0 & 0 & \chi^{ex}_{xyz} & 0 & 0 \\ 0 & \chi^{ex}_{xyz} & 0 & 0 & 0 & 0 \end{pmatrix}$	$\begin{pmatrix} 0 & 0 & 0 & 0 & 0 & 0 \\ 0 & 0 & 0 & 0 & 0 & 0 \\ 0 & 0 & 0 & 0 & 0 & 0 \end{pmatrix}$
Cubic	T_d	$\begin{pmatrix} 0 & 0 & 0 & 0 & \chi^{ex}_{xyz} & 0 \\ 0 & 0 & 0 & \chi^{ex}_{xyz} & 0 & 0 \\ 0 & \chi^{ex}_{xyz} & 0 & 0 & 0 & 0 \end{pmatrix}$	$\begin{pmatrix} 0 & 0 & 0 & 0 & 0 & 0 \\ 0 & 0 & 0 & 0 & 0 & 0 \\ 0 & 0 & 0 & 0 & 0 & 0 \end{pmatrix}$
Trigonal	C_3	$\begin{pmatrix} \chi^{ex}_{xxx} & -\chi^{ex}_{yyy} & -\chi^{ex}_{xxx} & -\chi^{ex}_{xxx} & \chi^{ex}_{xyz} & 0 \\ -\chi^{ex}_{yyy} & -\chi^{ex}_{xxx} & \chi^{ex}_{yyy} & -\chi^{ex}_{xyz} & -\chi^{ex}_{xxx} & 0 \\ \chi^{ex}_{zxx} & 0 & \chi^{ex}_{zxx} & 0 & 0 & \chi^{ex}_{zzz} \end{pmatrix}$	$\begin{pmatrix} 0 & 0 & 0 & 0 & \chi^{in}_{xyz} & 0 \\ 0 & 0 & 0 & -\chi^{in}_{xyz} & 0 & 0 \\ \chi^{in}_{zxx} & 0 & \chi^{in}_{zxx} & 0 & 0 & 0 \end{pmatrix}$
Trigonal	C_{3v}	$\begin{pmatrix} 0 & -\chi^{ex}_{yyy} & 0 & 0 & 0 & 0 \\ -\chi^{ex}_{yyy} & 0 & \chi^{ex}_{yyy} & 0 & 0 & 0 \\ \chi^{ex}_{zxx} & 0 & \chi^{ex}_{zxx} & 0 & 0 & \chi^{ex}_{zzz} \end{pmatrix}$	$\begin{pmatrix} 0 & 0 & 0 & 0 & 0 & 0 \\ 0 & 0 & 0 & 0 & 0 & 0 \\ \chi^{in}_{zxx} & 0 & \chi^{in}_{zxx} & 0 & 0 & 0 \end{pmatrix}$
Trigonal	D_3	$\begin{pmatrix} \chi^{ex}_{xxx} & 0 & -\chi^{ex}_{xxx} & 0 & \chi^{ex}_{xyz} & 0 \\ 0 & -\chi^{ex}_{xxx} & 0 & -\chi^{ex}_{xyz} & 0 & 0 \\ 0 & 0 & 0 & 0 & 0 & 0 \end{pmatrix}$	$\begin{pmatrix} 0 & 0 & 0 & 0 & \chi^{in}_{xyz} & 0 \\ 0 & 0 & 0 & -\chi^{in}_{xyz} & 0 & 0 \\ 0 & 0 & 0 & 0 & 0 & 0 \end{pmatrix}$
Hexagonal	C_6	$\begin{pmatrix} 0 & 0 & 0 & \chi^{ex}_{xxz} & \chi^{ex}_{xyz} & 0 \\ 0 & 0 & 0 & -\chi^{ex}_{xyz} & \chi^{ex}_{xxz} & 0 \\ \chi^{ex}_{zxx} & 0 & \chi^{ex}_{zxx} & 0 & 0 & \chi^{ex}_{zzz} \end{pmatrix}$	$\begin{pmatrix} 0 & 0 & 0 & -\chi^{in}_{zxx} & \chi^{in}_{xyz} & 0 \\ 0 & 0 & 0 & -\chi^{in}_{xyz} & -\chi^{in}_{zxx} & 0 \\ \chi^{in}_{zxx} & 0 & \chi^{in}_{zxx} & 0 & 0 & 0 \end{pmatrix}$
Hexagonal	C_{6v}	$\begin{pmatrix} 0 & 0 & 0 & \chi^{ex}_{xxz} & 0 & 0 \\ 0 & 0 & 0 & 0 & \chi^{ex}_{xxz} & 0 \\ \chi^{ex}_{zxx} & 0 & \chi^{ex}_{zxx} & 0 & 0 & \chi^{ex}_{zzz} \end{pmatrix}$	$\begin{pmatrix} 0 & 0 & 0 & -\chi^{in}_{zxx} & 0 & 0 \\ 0 & 0 & 0 & 0 & -\chi^{in}_{zxx} & 0 \\ \chi^{in}_{zxx} & 0 & \chi^{in}_{zxx} & 0 & 0 & 0 \end{pmatrix}$
Hexagonal	D_6	$\begin{pmatrix} 0 & 0 & 0 & 0 & \chi^{ex}_{xyz} & 0 \\ 0 & 0 & 0 & -\chi^{ex}_{xyz} & 0 & 0 \\ 0 & 0 & 0 & 0 & 0 & 0 \end{pmatrix}$	$\begin{pmatrix} 0 & 0 & 0 & 0 & \chi^{in}_{xyz} & 0 \\ 0 & 0 & 0 & -\chi^{in}_{xyz} & 0 & 0 \\ 0 & 0 & 0 & 0 & 0 & 0 \end{pmatrix}$
Hexagonal	C_{3h}	$\begin{pmatrix} \chi^{ex}_{xxx} & -\chi^{ex}_{yyy} & -\chi^{ex}_{xxx} & 0 & 0 & 0 \\ -\chi^{ex}_{yyy} & -\chi^{ex}_{xxx} & \chi^{ex}_{yyy} & 0 & 0 & 0 \\ 0 & 0 & 0 & 0 & 0 & 0 \end{pmatrix}$	$\begin{pmatrix} 0 & 0 & 0 & 0 & 0 & 0 \\ 0 & 0 & 0 & 0 & 0 & 0 \\ 0 & 0 & 0 & 0 & 0 & 0 \end{pmatrix}$
Hexagonal	D_{3h}	$\begin{pmatrix} 0 & -\chi^{ex}_{yyy} & 0 & 0 & 0 & 0 \\ -\chi^{ex}_{yyy} & 0 & \chi^{ex}_{yyy} & 0 & 0 & 0 \\ 0 & 0 & 0 & 0 & 0 & 0 \end{pmatrix}$	$\begin{pmatrix} 0 & 0 & 0 & 0 & 0 & 0 \\ 0 & 0 & 0 & 0 & 0 & 0 \\ 0 & 0 & 0 & 0 & 0 & 0 \end{pmatrix}$

The C_n axis is assumed in order as the z-, x- and y-axis, the mirror plane σ_v is assumed in order as the yz- and zx-plane and the mirror plane σ_h is assumed as the xy-plane.
